# Weakly supervised artificial intelligence for multi-cancer detection of lymph node metastasis on whole slide images

**DOI:** 10.1038/s41467-026-76410-w

**Published:** 2026-08-14

**Authors:** Lili Sun, Shuilian Yao, Qi Jia, Yanmei Zhu

**Affiliations:** 1 Department of Pathology, Cancer Hospital of Dalian University of Technology (Liaoning Cancer Hospital and Institute, Cancer Hospital of China Medical University), Shenyang, China; 2 School of Software Technology, DUT-RU International School of Information Science & Engineering, Dalian University of Technology, Dalian, China

**Keywords:** Machine learning, Imaging, Cancer imaging, Metastasis

## Abstract

The accurate identification of lymph node metastasis is critical for cancer diagnosis/treatment but remains time-consuming and error-prone for pathologists. We develop MambaMIL+HiLA-MIL, a multiple instance learning model combining Vision Mamba with high-low attention separation mechanism. This model is compared against six baselines under four feature extractors (ResNet, UNI, Virchow, and GigaPath). Ten-fold cross-validation is employed for model evaluation. Our model significantly outperforms all baselines. It exhibits strong performance in detection of isolated tumor cells and micro-metastasis, while maintaining high performance for negative and macro-metastasis cases. The model still demonstrates good stability in detecting lymph node metastasis across multi-cancer and various individual cancer types. UNI and GigaPath yield significantly better performance than ResNet and Virchow. Here, we show that MambaMIL+HiLA-MIL is a multi-cancer four-class lymph node metastasis model, demonstrating high efficiency and robustness across multiple centers and various feature extractors, offering a reliable tool for clinical lymph node metastasis classification.

## Abstract (web summary)

The identification of lymph node metastasis remains time-consuming and error-prone for pathologists. Here, the authors develop MambaMIL+HiLA-MIL, a multiple instance learning model that allows the accurate detection of lymph node metastasis, particularly isolated tumor cells and micro-metastasis across multi-cancer and various individual cancer types from histopathology images.

## Introduction

Assessment of lymph node metastasis (LNM) is important for determining the stage of tumor-node-metastasis (TNM) and selecting an appropriate treatment strategy to ensure optimal outcomes for cancer patients. Based on the presence or absence of LNM, the number of cancer cells, and the size of the tumor foci within the metastatic lymph nodes, LNM is classified as macro-metastasis (>2 mm), micro-metastasis (>0.2–≤2 mm), isolated tumor cells (ITCs, ≤200 scattered tumor cells or tumor clusters ≤0.2 mm), or no metastasis^1^. Accurate classification of LNM is necessary to improve the accuracy of TNM staging and predict patient prognosis^2,3^. In clinical practice, pathological assessment is conventionally the gold standard for cancer diagnosis. However, accurate histopathological interpretation of LNM is not only time-consuming and labor-intensive for pathologists but also prone to false negatives^4^.

With the advancement of artificial intelligence (AI) and digital pathology, deep learning techniques have been developed to assess whole slide images (WSIs) of lymph nodes for the detection of LNM in various types of cancers. Most previous studies have been conducted with fully supervised settings. Undoubtedly, the Cancer Metastases in Lymph Nodes Challenge 2016 (CAMELYON16) was a milestone in the use of AI for pathological assessment. Although a breakthrough in the capability for automati×c detection of LNM was achieved, with algorithm performance reaching the level of diagnosis by a pathologist, detection of ITCs was regrettably excluded^5^. In 2017, the CAMELYON17 was launched. As compared to the CAMELYON16, the most critical change to the CAMELYON17 was the inclusion of ITCs, the smallest metastatic type, in the dataset. All algorithms performed well for identification of negative results and macro-metastasis, while performance varied for detection of micro-metastasis and all performed poorly for ITCs, with accuracy rates below 40% (range, 0–34.3%)^6^. In addition to breast cancer, which was the earliest and most extensive application^7–9^, fully supervised deep learning techniques have also gradually been applied for LNM detection in other cancer types^10–22^. Similar to the CAMELYON16, studies that achieved both high sensitivity and specificity (> 0.90) did not include ITCs for precise four-class classification of LNM^16,19–21^, indicating that the technical bottleneck of poor ITCs detection performance of the CAMELYON17 remains unresolved.

The fully supervised deep learning methods used in the aforementioned studies relied on manual annotations by pathologists, but were labor-intensive and prone to annotation bias. In recent years, weakly supervised deep learning algorithms have emerged, including multiple instance learning (MIL), which has been widely applied for WSI classification tasks in pathology^23–28^. Building on this progress, attention-based deep multiple instance learning (AB-MIL) algorithms^23^ and MIL-ResNet34-RNN algorithms^24^ have been developed, which not only improve classification performance but also provide interpretability of the models. However, to achieve ideal results, thousands of WSIs with slide-level labels are required. In 2021, Lu et al. reported a clustering-constrained attention multiple-instance learning (CLAM) algorithm to solve general multi-classification problems. The CLAM model has two variants: single-branch attention (CLAM-SB) and multi-branch attention (CLAM-MB)^25^. The CLAM model, trained on a dataset of several hundred WSIs combined from CAMELYON16 and CAMELYON17, can achieve an area under the receiver operating characteristic (ROC) curve (AUC) > 0.9 with the test set. However, regrettably, this study did not perform precise four-class classification of LNM. Subsequently, Li et al. proposed a dual-stream multiple instance learning (DSMIL) model, which also did not include precise LNM four-class classification^26^. Tan et al. introduced the DT-DSMIL model, which is based on a deformable transformer and DSMIL for detection of LNM in colorectal cancer. The model achieved good performance for lymph nodes with macro-metastasis and micro-metastasis, but identification of ITCs was relatively poor, with an AUC of only 0.8120 and accuracy of 27.3%^27^. The AB-MIL, CLAM-SB, CLAM-MB, and DSMIL models are based on convolutional neural networks (CNNs). However, CNNs lack global context awareness and the application of these weakly supervised deep learning algorithms for detection of LNM and classification tasks remains challenging. In 2021, Shao et al. reported a transformer-based correlated multiple instance learning (TransMIL) algorithm, which can better utilize the information between instances, reduce uncertainty, and effectively handle imbalanced/balanced and binary/multi- classification problems^28^. However, to date, there have been no reports of the TransMIL weakly supervised algorithm applied for LNM detection tasks.

The performance of the algorithm largely depends on the adopted feature extraction method. Traditional WSI feature extraction generally adopts CNNs pre-trained on ImageNet, among which ResNet50 serves as the most frequently used backbone. Benefiting from its outstanding performance in natural image classification and strong inductive bias capability, ResNet50 has long been widely utilized for feature extraction in pathological images^29^. Nevertheless, significant domain discrepancies exist between natural and pathological images, restricting the generalization of ImageNet-pre-trained models in pathological tasks. To address the limitations of conventional CNNs, large-scale self-supervised learning-based foundation models for computational pathology have emerged. UNI is pre-trained on over 100,000 H&E-stained whole-slide images, possessing capabilities such as resolution-agnostic classification, few-shot prototype classification, and generalized classification for 108 cancer subtypes^30^. GigaPath (Prov-GigaPath) is a whole-slide pathology foundation model developed by Xu et al. Targeting the unique computational challenge of gigapixel whole-slide images containing tens of thousands of image patches in digital pathology, GigaPath adopts a novel vision transformer architecture and leverages the LongNet approach to model ultra-large contextual ranges^31^. Virchow is a large-scale digital pathology foundation model proposed by Vorontsov et al. Its feature embedding achieves superior cancer detection performance across all cancer types, including rare malignancies^32^. Nevertheless, up to the present, comprehensive performance comparisons among the aforementioned four feature extraction backbones for LNM classification remain unreported.

In this work, we propose a MIL model with high-low attention separation (HiLA), named MambaMIL + HiLA-MIL, based on the Vision Mamba as the fundamental architecture of the MIL model (MambaMIL)^33^ and the incorporation of a HiLA mechanism. Our model significantly outperforms all baselines across the four feature extractors. It exhibits good performance in detection of ITCs and micro-metastasis, while maintaining high performance for negative and macro-metastasis cases. The model still demonstrates good stability in detecting LNM across multi-cancer and various individual cancer types. Comparative analysis of the four feature extractors demonstrates that UNI and GigaPath yielded significantly better performance than ResNet and Virchow. In conclusion, MambaMIL + HiLA-MIL is a multi-cancer four-class LNM model, demonstrating high efficiency and robustness across multiple centers and various feature extractors, particularly for ITCs detection, offering a reliable tool for clinical LNM classification.

## Results

### Comparison with baseline models

On the internal NIMM dataset, the performance metrics of the six baseline models (AB-MIL, CLAM-SB, CLAM-MB, DSMIL, TransMIL, and MambaMIL) and the proposed MambaMIL + HiLA-MIL model for the overall four-class classification and each LNM category level (negative, ITCs, micro-metastasis, and macro-metastasis) base on four feature extractors (ResNet, UNI, Virchow, and GigaPath) are detailed in Table 1. Across all four feature extractors, the proposed MambaMIL + HiLA-MIL model outperformed all six baseline models across all evaluation metrics, with most performance improvements being statistically significant, thus achieving state-of-the-art performance in overall four-class classification.

**Table 1:** Evaluation metrics of overall four-class classification and each LNM category across four feature extractors on NIMM dataset

ResNet												
Methods	Overall											
	AUC	*P*-value	Accuracy	*P*-value	Precision	*P*-value	Recall	*P*-value	Specificity	*P*-value	F1-Score	*P*-value
ABMIL	0.9637 ± 0.0170	0.0001 (***↓)	0.8822 ± 0.0411	<0.0001 (***↓)	0.7885 ± 0.0925	0.0196 (*↓)	0.7541 ± 0.0750	0.0184 (*↓)	0.9601 ± 0.0145	0.0552 (ns↓)	0.7470 ± 0.0691	0.0208 (*↓)
CLAM-SB	0.9617 ± 0.0172	<0.0001 (***↓)	0.8898 ± 0.0255	0.0142 (*↓)	0.8097 ± 0.0727	0.3308 (ns↓)	0.7556 ± 0.0601	0.0286 (*↓)	0.9609 ± 0.0111	0.1517 (ns↓)	0.7538 ± 0.0440	0.1192 (ns↓)
CLAM-MB	0.9613 ± 0.0146	0.0031 (**↓)	0.8883 ± 0.0298	0.0059 (**↓)	0.7650 ± 0.0668	0.0639 (ns↓)	0.7484 ± 0.0446	0.0193 (*↓)	0.9616 ± 0.0110	0.2037 (ns↓)	0.7376 ± 0.0439	0.0340 (*↓)
DSMIL	0.9613 ± 0.0130	<0.0001 (***↓)	0.8595 ± 0.0409	<0.0001 (***↓)	0.7266 ± 0.1036	<0.0001 (***↓)	0.7207 ± 0.0694	<0.0001 (***↓)	0.9539 ± 0.0108	0.0001 (***↓)	0.7002 ± 0.0790	<0.0001 (***↓)
TransMIL	0.9587 ± 0.0130	<0.0001 (***↓)	0.8565 ± 0.0531	<0.0001 (***↓)	0.7682 ± 0.0707	0.0004 (***↓)	0.7160 ± 0.0732	0.0001 (***↓)	0.9493 ± 0.0208	<0.0001 (***↓)	0.7093 ± 0.0560	0.0002 (***↓)
MambaMIL	0.9588 ± 0.0207	<0.0001 (***↓)	0.8762 ± 0.0320	<0.0001 (***↓)	0.7797 ± 0.0846	0.0321 (*↓)	0.7479 ± 0.0710	0.0241 (*↓)	0.9562 ± 0.0135	0.0097 (**↓)	0.7310 ± 0.0585	0.0214 (*↓)
MambaMIL + HiLA-MIL (Ours)	0.9700 ± 0.0140	N/A	0.9019 ± 0.0318	N/A	0.8193 ± 0.0661	N/A	0.8029 ± 0.0518	N/A	0.9657 ± 0.0111	N/A	0.7993 ± 0.0547	N/A
Methods	Negative											
	AUC	*P*-value	Accuracy	*P*-value	Precision	*P*-value	Recall	*P*-value	Specificity	*P*-value	F1-Score	*P*-value
ABMIL	0.9959 ± 0.0128	1.0000 (ns↓)	0.9940 ± 0.0105	0.5007 (ns↓)	0.9958 ± 0.0132	0.5007 (ns↓)	0.9870 ± 0.0293	<0.0001 (***↓)	0.9977 ± 0.0072	1.0000 (ns↓)	0.9911 ± 0.0157	0.5007 (ns↓)
CLAM-SB	0.9941 ± 0.0140	<0.0001 (***↓)	0.9925 ± 0.0107	0.3712 (ns↓)	1.0000 ± 0.0000	0.9384 (ns↑)	0.9783 ± 0.0307	<0.0001 (***↓)	1.0000 ± 0.0000	1.0000 (ns↑)	0.9888 ± 0.0160	0.1218 (ns↓)
CLAM-MB	0.9938 ± 0.0123	0.4906 (ns↓)	0.9940 ± 0.0105	0.6222 (ns↓)	1.0000 ± 0.0000	1.0000 (ns↑)	0.9826 ± 0.0304	<0.0001 (***↓)	1.0000 ± 0.0000	1.0000 (ns↑)	0.9910 ± 0.0158	0.1219 (ns↓)
DSMIL	0.9968 ± 0.0067	0.5019 (ns↑)	0.9880 ± 0.0170	0.0309 (*↓)	0.9885 ± 0.0365	<0.0001 (***↓)	0.9783 ± 0.0369	<0.0001 (***↓)	0.9932 ± 0.0216	<0.0001 (***↓)	0.9826 ± 0.0243	<0.0001 (***↓)
TransMIL	0.9936 ± 0.0135	0.0004 (***↓)	0.9652 ± 0.0594	<0.0001 (***↓)	0.9480 ± 0.1148	<0.0001 (***↓)	0.9739 ± 0.0367	<0.0001 (***↓)	0.9605 ± 0.0950	<0.0001 (***↓)	0.9562 ± 0.0663	<0.0001 (***↓)
MambaMIL	0.9924 ± 0.0162	0.2346 (ns↓)	0.9895 ± 0.0159	0.1149 (ns↓)	1.0000 ± 0.0000	0.9861 (ns↑)	0.9696 ± 0.0461	<0.0001 (***↓)	1.0000 ± 0.0000	1.0000 (ns↑)	0.9840 ± 0.0244	0.0145 (*↓)
MambaMIL + HiLA-MIL (Ours)	0.9960 ± 0.0125	N/A	0.9970 ± 0.0063	N/A	0.9958 ± 0.0132	N/A	0.9957 ± 0.0137	N/A	0.9977 ± 0.0072	N/A	0.9957 ± 0.0092	N/A
Methods	ITC											
	AUC	*P*-value	Accuracy	*P*-value	Precision	*P*-value	Recall	*P*-value	Specificity	*P*-value	F1-Score	*P*-value
ABMIL	0.9478 ± 0.0303	0.0005 (***↓)	0.9426 ± 0.0293	0.0615 (ns↓)	0.6231 ± 0.2542	0.0683 (ns↓)	0.5250 ± 0.2751	0.0000 (***↓)	0.9695 ± 0.0289	0.3874 (ns↓)	0.5131 ± 0.1839	0.0049 (**↓)
CLAM-SB	0.9490 ± 0.0318	0.0064 (**↓)	0.9502 ± 0.0121	0.5200 (ns↓)	0.6921 ± 0.2390	0.7560 (ns↑)	0.5250 ± 0.2751	0.0000 (***↓)	0.9775 ± 0.0188	0.6107 (ns↑)	0.5301 ± 0.1577	0.0608 (ns↓)
CLAM-MB	0.9486 ± 0.0256	0.0054 (**↓)	0.9396 ± 0.0140	0.0436 (*↓)	0.5278 ± 0.1774	0.0397 (*↓)	0.5250 ± 0.2993	0.0039 (**↓)	0.9663 ± 0.0244	0.2080 (ns↓)	0.4840 ± 0.1569	0.0069 (**↓)
DSMIL	0.9520 ± 0.0230	0.0009 (***↓)	0.9229 ± 0.0401	0.0000 (***↓)	0.4818 ± 0.2297	0.0000 (***↓)	0.6000 ± 0.2415	0.0307 (*↓)	0.9436 ± 0.0465	0.0000 (***↓)	0.4947 ± 0.1499	0.0000 (***↓)
TransMIL	0.9502 ± 0.0280	0.0850 (ns↓)	0.9411 ± 0.0219	0.0423 (*↓)	0.6302 ± 0.2769	0.0380 (*↓)	0.5250 ± 0.2751	0.0036 (**↓)	0.9678 ± 0.0304	0.2249 (ns↓)	0.5030 ± 0.1423	0.0078 (**↓)
MambaMIL	0.9522 ± 0.0355	0.0415 (*↓)	0.9411 ± 0.0149	0.0652 (ns↓)	0.5810 ± 0.2358	0.0355 (*↓)	0.6000 ± 0.3162	0.1110 (ns↓)	0.9630 ± 0.0228	0.1827 (ns↓)	0.5207 ± 0.1537	0.0587 (ns↓)
MambaMIL + HiLA-MIL (Ours)	0.9643 ± 0.0240	N/A	0.9562 ± 0.0181	N/A	0.6462 ± 0.1640	N/A	0.7000 ± 0.1581	N/A	0.9727 ± 0.0171	N/A	0.6607 ± 0.1278	N/A
Methods	Micro											
	AUC	*P*-value	Accuracy	*P*-value	Precision	*P*-value	Recall	*P*-value	Specificity	*P*-value	F1-Score	*P*-value
ABMIL	0.9311 ± 0.0261	0.0000 (***↓)	0.8958 ± 0.0303	0.0698 (ns↓)	0.6110 ± 0.1771	0.0105 (*↓)	0.5722 ± 0.2493	1.0000 (ns↑)	0.9414 ± 0.0356	0.0029 (**↓)	0.5564 ± 0.1568	0.3069 (ns↓)
CLAM-SB	0.9271 ± 0.0251	0.0007 (***↓)	0.9003 ± 0.0227	0.2918 (ns↓)	0.6333 ± 0.1524	0.1456 (ns↓)	0.5611 ± 0.2342	1.0000 (ns↓)	0.9483 ± 0.0257	0.1877 (ns↓)	0.5621 ± 0.1373	0.4595 (ns↓)
CLAM-MB	0.9267 ± 0.0295	0.0545 (ns↓)	0.9004 ± 0.0317	0.2633 (ns↓)	0.6111 ± 0.1369	0.1934 (ns↓)	0.5250 ± 0.2737	0.2842 (ns ↓ )	0.9534 ± 0.0282	0.4855 (ns↓)	0.5354 ± 0.2046	0.2436 (ns↓)
DSMIL	0.9176 ± 0.0237	0.0000 (***↓)	0.8761 ± 0.0369	0.0000 (***↓)	0.5097 ± 0.2699	0.0000 (***↓)	0.3722 ± 0.2620	0.0000 (***↓)	0.9466 ± 0.0298	0.0985 (ns↓)	0.3961 ± 0.2336	0.0000 (***↓)
TransMIL	0.9155 ± 0.0280	0.0000 (***↓)	0.8867 ± 0.0305	0.0078 (**↓)	0.5802 ± 0.2033	0.0077 (**↓)	0.4458 ± 0.2382	0.0106 (*↓)	0.9483 ± 0.0293	0.1641 (ns↓)	0.4682 ± 0.1890	0.0058 (**↓)
MambaMIL	0.9187 ± 0.0342	0.0000 (***↓)	0.8958 ± 0.0259	0.1240 (ns↓)	0.6354 ± 0.1916	0.1355 (ns↓)	0.4736 ± 0.2586	0.0534 (ns↓)	0.9552 ± 0.0327	0.6514 (ns↓)	0.4963 ± 0.1956	0.0601 (ns↓)
MambaMIL + HiLA-MIL (Ours)	0.9398 ± 0.0260	N/A	0.9124 ± 0.0300	N/A	0.7102 ± 0.1895	N/A	0.5708 ± 0.2013	N/A	0.9603 ± 0.0282	N/A	0.6065 ± 0.1402	N/A
Methods	Macro											
	AUC	AUC Perm P	Accuracy	*P*-value	Precision	*P*-value	Recall	*P*-value	Specificity	*P*-value	F1-Score	*P*-value
ABMIL	0.9801 ± 0.0164	0.0684 (ns↑)	0.9320 ± 0.0364	0.4613 (ns↓)	0.9243 ± 0.0471	0.8178 (ns↓)	0.9323 ± 0.0467	0.2226 (ns↓)	0.9320 ± 0.0429	1.0000 (ns↑)	0.9276 ± 0.0392	0.2619 (ns↓)
CLAM-SB	0.9767 ± 0.0171	0.0230 (*↓)	0.9366 ± 0.0309	1.0000 (ns↓)	0.9135 ± 0.0509	0.2361 (ns↓)	0.9581 ± 0.0431	0.3397 (ns↑)	0.9179 ± 0.0512	0.0926 (ns↓)	0.9340 ± 0.0318	0.9759 (ns↓)
CLAM-MB	0.9761 ± 0.0174	0.1803 (ns↓)	0.9427 ± 0.0263	0.6695 (ns↑)	0.9211 ± 0.0433	0.7413 (ns↓)	0.9612 ± 0.0296	0.0381 (*↑)	0.9265 ± 0.0426	0.3883 (ns↓)	0.9401 ± 0.0274	0.4148 (ns↑)
DSMIL	0.9788 ± 0.0163	0.0682 (ns↓)	0.9320 ± 0.0191	0.5075 (ns↓)	0.9262 ± 0.0472	0.8318 (ns↑)	0.9323 ± 0.0442	0.3870 (ns↓)	0.9321 ± 0.0447	1.0000 (ns↑)	0.9276 ± 0.0202	0.3584 (ns↓)
TransMIL	0.9753 ± 0.0194	0.0002 (***↓)	0.9199 ± 0.0572	0.0165 (*↓)	0.9143 ± 0.0478	0.1875 (ns↓)	0.9194 ± 0.1345	0.0216 (*↓)	0.9207 ± 0.0516	0.1777 (ns↓)	0.9099 ± 0.0797	0.0195 (*↓)
MambaMIL	0.9721 ± 0.0238	0.0002 (***↓)	0.9261 ± 0.0287	0.1639 (ns↓)	0.9024 ± 0.0543	0.0423 (*↓)	0.9484 ± 0.0461	1.0000 (ns↑)	0.9067 ± 0.0568	0.0199 (*↓)	0.9232 ± 0.0297	0.1493 (ns↓)
MambaMIL + HiLA-MIL (Ours)	0.9800 ± 0.0171	N/A	0.9381 ± 0.0279	N/A	0.9249 ± 0.0354	N/A	0.9452 ± 0.0431	N/A	0.9320 ± 0.0334	N/A	0.9343 ± 0.0303	N/A
UNI												
Methods	Overall											
	AUC	*P*-value	Accuracy	*P*-value	Precision	*P*-value	Recall	*P*-value	Specificity	*P*-value	F1-Score	*P*-value
ABMIL	0.9636 ± 0.0173	0.1030 (ns↓)	0.9049 ± 0.0264	<0.0001 (***↓)	0.7887 ± 0.0784	0.0010 (**↓)	0.7912 ± 0.0444	0.0015 (**↓)	0.9706 ± 0.0082	0.0167 (*↓)	0.7721 ± 0.0484	0.0015 (**↓)
CLAM-SB	0.9623 ± 0.0154	0.0160 (*↓)	0.9079 ± 0.0267	<0.0001 (***↓)	0.7932 ± 0.0796	0.0028 (**↓)	0.7900 ± 0.0440	0.0009 (***↓)	0.9709 ± 0.0083	0.0183 (*↓)	0.7740 ± 0.0494	0.0020 (**↓)
CLAM-MB	0.9631 ± 0.0143	0.0937 (ns↓)	0.9064 ± 0.0221	<0.0001 (***↓)	0.7964 ± 0.0794	0.0017 (**↓)	0.7862 ± 0.0424	0.0008 (***↓)	0.9702 ± 0.0065	0.0172 (*↓)	0.7638 ± 0.0521	0.0012 (**↓)
DSMIL	0.9617 ± 0.0149	0.0333 (*↓)	0.9079 ± 0.0298	<0.0001 (***↓)	0.8000 ± 0.0767	0.0008 (***↓)	0.7916 ± 0.0576	0.0010 (**↓)	0.9715 ± 0.0085	0.0437 (*↓)	0.7806 ± 0.0541	0.0005 (***↓)
TransMIL	0.9699 ± 0.0112	0.3551 (ns↓)	0.9124 ± 0.0264	0.0002 (***↓)	0.8146 ± 0.0727	0.0075 (**↓)	0.7963 ± 0.0544	0.0019 (**↓)	0.9720 ± 0.0078	0.0695 (ns↓)	0.7881 ± 0.0555	0.0032 (**↓)
MambaMIL	0.9634 ± 0.0122	0.0979 (ns↓)	0.9094 ± 0.0189	<0.0001 (***↓)	0.8106 ± 0.0524	0.0201 (*↓)	0.8027 ± 0.0489	0.0082 (**↓)	0.9707 ± 0.0059	0.0293 (*↓)	0.7892 ± 0.0491	0.0114 (*↓)
MambaMIL + HiLA-MIL (Ours)	0.9710 ± 0.0105	N/A	0.9290±0.0256	N/A	0.8722 ± 0.0646	N/A	0.8576 ± 0.0501	N/A	0.9772 ± 0.0072	N/A	0.8455 ± 0.0470	N/A
Methods	Negative											
	AUC	*P*-value	Accuracy	*P*-value	Precision	*P*-value	Recall	P-value	Specificity	*P*-value	F1-Score	*P*-value
ABMIL	0.9999 ± 0.0003	<0.0001 (***↓)	0.9970 ± 0.0094	0.4969 (ns↓)	0.9957 ± 0.0137	0.4969 (ns↓)	0.9957 ± 0.0137	1.0000 (ns↓)	0.9977 ± 0.0072	1.0000 (ns↓)	0.9957 ± 0.0137	0.4969 (ns↓)
CLAM-SB	0.9997 ± 0.0007	0.9796 (ns↓)	0.9940 ± 0.0126	0.1221 (ns↓)	0.9957 ± 0.0137	0.1221 (ns↓)	0.9870 ± 0.0293	<0.0001 (***↓)	0.9977 ± 0.0072	1.0000 (ns↓)	0.9911 ± 0.0188	0.1221 (ns↓)
CLAM-MB	1.0000 ± 0.0000	1.0000 (ns↓)	0.9985 ± 0.0048	1.0000 (ns↓)	0.9958 ± 0.0132	1.0000 (ns↓)	1.0000 ± 0.0000	1.0000 (ns↓)	0.9977 ± 0.0074	1.0000 (ns↓)	0.9979 ± 0.0067	1.0000 (ns↓)
DSMIL	1.0000 ± 0.0000	0.0751 (ns↓)	0.9985 ± 0.0048	1.0000 (ns↓)	0.9958 ± 0.0132	1.0000 (ns↓)	1.0000 ± 0.0000	1.0000 (ns↓)	0.9977 ± 0.0074	1.0000 (ns↓)	0.9979 ± 0.0067	1.0000 (ns↓)
TransMIL	1.0000 ± 0.0000	1.0000 (ns↓)	1.0000 ± 0.0000	1.0000 (ns↓)	1.0000 ± 0.0000	1.0000 (ns↓)	1.0000 ± 0.0000	1.0000 (ns↓)	1.0000 ± 0.0000	1.0000 (ns↓)	1.0000 ± 0.0000	1.0000 (ns↓)
MambaMIL	1.0000 ± 0.0000	<0.0001 (***↓)	0.9970 ± 0.0064	0.4978 (ns↓)	0.9958 ± 0.0132	0.4978 (ns↓)	0.9957 ± 0.0137	1.0000 (ns↓)	0.9977 ± 0.0074	1.0000 (ns↓)	0.9957 ± 0.0092	0.4978 (ns↓)
MambaMIL + HiLA-MIL (Ours)	1.0000 ± 0.0000	N/A	1.0000 ± 0.0000	N/A	1.0000 ± 0.0000	N/A	1.0000 ± 0.0000	N/A	1.0000 ± 0.0000	N/A	1.0000 ± 0.0000	N/A
Methods	ITC											
	AUC	*P*-value	Accuracy	*P*-value	Precision	*P*-value	Recall	*P*-value	Specificity	*P*-value	F1-Score	*P*-value
ABMIL	0.9523 ± 0.0295	0.0620 (ns↓)	0.9426 ± 0.0140	0.0006 (***↓)	0.5171 ± 0.2512	0.0034 (**↓)	0.5750 ± 0.2899	<0.0001 (***↓)	0.9663 ± 0.0221	0.0634 (ns ↓ )	0.5065 ± 0.2066	0.0002 (***↓)
CLAM-SB	0.9497 ± 0.0300	0.0041 (**↓)	0.9426 ± 0.0140	0.0006 (***↓)	0.5171 ± 0.2512	0.0034 (**↓)	0.5750 ± 0.2899	<0.0001 (***↓)	0.9663 ± 0.0221	0.0634 (ns↓)	0.5065 ± 0.2066	0.0002 (***↓)
CLAM-MB	0.9488 ± 0.0315	0.0281 (*↓)	0.9411 ± 0.0151	0.0005 (***↓)	0.5116 ± 0.2522	0.0016 (**↓)	0.6000 ± 0.3162	<0.0001 (***↓)	0.9630 ± 0.0264	0.0911 (ns↓)	0.5081 ± 0.2074	0.0002 (***↓)
DSMIL	0.9467 ± 0.0275	0.0030 (**↓)	0.9411 ± 0.0182	<0.0001 (***↓)	0.5521 ± 0.2017	0.0002 (***↓)	0.6000 ± 0.2415	<0.0001 (***↓)	0.9630 ± 0.0187	0.0366 (*↓)	0.5382 ± 0.1492	<0.0001 (***↓)
TransMIL	0.9556 ± 0.0186	0.0586 (ns↓)	0.9456 ± 0.0178	0.0001 (***↓)	0.5950 ± 0.1981	0.0012 (**↓)	0.6000 ± 0.2415	<0.0001 (***↓)	0.9679 ± 0.0214	0.0303 (*↓)	0.5579 ± 0.1485	0.0001 (***↓)
MambaMIL	0.9628 ± 0.0155	0.3057 (ns↓)	0.9486 ± 0.0127	0.0169 (*↓)	0.5671 ± 0.0903	0.0526 (ns↓)	0.6500 ± 0.2415	0.0169 (*↓)	0.9679 ± 0.0131	0.1364 (ns↓)	0.5881 ± 0.1377	0.0191 (*↓)
MambaMIL + HiLA-MIL (Ours)	0.9630 ± 0.0199	N/A	0.9623 ± 0.0178	N/A	0.7395 ± 0.2062	N/A	0.7750 ± 0.2486	N/A	0.9743 ± 0.0241	N/A	0.7072 ± 0.1106	N/A
Methods	Micro											
	AUC	*P*-value	Accuracy	*P*-value	Precision	*P*-value	Recall	*P*-value	Specificity	*P*-value	F1-Score	*P*-value
ABMIL	0.9185 ± 0.0534	0.2509 (ns↓)	0.9094 ± 0.0222	0.0058 (**↓)	0.6739 ± 0.1550	0.0072 (**↓)	0.6458 ± 0.1996	0.1803 (ns↓)	0.9466 ± 0.0330	0.0078 (**↓)	0.6290 ± 0.0967	0.0219 (*↓)
CLAM-SB	0.9156 ± 0.0491	0.1550 (ns↓)	0.9140 ± 0.0211	0.0479 (*↓)	0.6974 ± 0.1454	0.0443 (*↓)	0.6333 ± 0.1962	0.0948 (ns↓)	0.9534 ± 0.0305	0.0617 (ns ↓ )	0.6360 ± 0.0980	0.0308 (*↓)
CLAM-MB	0.9197 ± 0.0484	0.3459 (ns)	0.9109 ± 0.0180	0.0263 (*↓)	0.7195 ± 0.1650	0.0454 (*↓)	0.5833 ± 0.2538	0.0212 (*↓)	0.9569 ± 0.0307	0.1410 (ns↓)	0.5899 ± 0.1595	0.0162 (*↓)
DSMIL	0.9171 ± 0.0539	0.2878 (ns↓)	0.9109 ± 0.0271	0.0194 (*↓)	0.6843 ± 0.1604	0.0154 (*↓)	0.6083 ± 0.1669	0.0472 (*↓)	0.9534 ± 0.0294	0.0349 (*↓)	0.6239 ± 0.1022	0.0138 (*↓)
TransMIL	0.9399 ± 0.0287	0.8372 (ns↑)	0.9154 ± 0.0227	0.0693 (ns↓)	0.7048 ± 0.1527	0.0861 (ns↓)	0.6208 ± 0.1919	0.0562 (ns↓)	0.9569 ± 0.0247	0.1400 (ns↓)	0.6334 ± 0.1245	0.0380 (*↓)
MambaMIL	0.9082 ± 0.0426	0.1207 (ns↓)	0.9139 ± 0.0227	0.0544 (ns↓)	0.7228 ± 0.1733	0.0717 (ns↓)	0.6069 ± 0.2058	0.0484 (*↓)	0.9569 ± 0.0296	0.1432 (ns↓)	0.6168 ± 0.1578	0.0285 (*↓)
MambaMIL + HiLA-MIL (Ours)	0.9355 ± 0.0312	N/A	0.9320 ± 0.0216	N/A	0.7839 ± 0.1702	N/A	0.6972 ± 0.1722	N/A	0.9655 ± 0.0304	N/A	0.7140 ± 0.0878	N/A
Methods	Macro											
	AUC	*P*-value	Accuracy	*P*-value	Precision	*P*-value	Recall	*P*-value	Specificity	*P*-value	F1-Score	*P*-value
ABMIL	0.9838 ± 0.0102	0.9412 (ns↓)	0.9608 ± 0.0226	0.8029 (ns↓)	0.9680 ± 0.0289	0.8275 (ns↑)	0.9484 ± 0.0486	0.5444 (ns↓)	0.9717 ± 0.0264	0.3692 (ns↑)	0.9571 ± 0.0254	0.5770 (ns↓)
CLAM-SB	0.9840 ± 0.0110	0.7825 (ns↓)	0.9653 ± 0.0201	0.5793 (ns↑)	0.9625 ± 0.0315	0.9092 (ns↓)	0.9645 ± 0.0442	0.2856 (ns↑)	0.9661 ± 0.0289	1.0000 (ns↓)	0.9626 ± 0.0224	0.7487 (ns↑)
CLAM-MB	0.9840 ± 0.0112	0.7142 (ns↓)	0.9623 ± 0.0203	1.0000 (ns↓)	0.9588 ± 0.0245	0.5106 (ns↓)	0.9613 ± 0.0451	0.5059 (ns↑)	0.9633 ± 0.0230	0.7255 (ns↓)	0.9593 ± 0.0230	0.9588 (ns↓)
DSMIL	0.9830 ± 0.0142	0.4993 (ns↓)	0.9653 ± 0.0201	0.5983 (ns↑)	0.9678 ± 0.0203	0.7944 (ns↑)	0.9581 ± 0.0431	1.0000 (ns↑)	0.9717 ± 0.0191	0.4482 (ns↑)	0.9623 ± 0.0225	0.7603 (ns↑)
TransMIL	0.9840 ± 0.0097	0.9853 (ns↓)	0.9638 ± 0.0161	1.0000 (ns↑)	0.9588 ± 0.0202	0.5505 (ns↓)	0.9645 ± 0.0386	0.3434 (ns↑)	0.9632 ± 0.0192	0.7247 (ns↓)	0.9611 ± 0.0182	0.8396 (ns↑)
MambaMIL	0.9825 ± 0.0089	0.8250 (ns↓)	0.9592 ± 0.0102	0.6500 (ns↓)	0.9566 ± 0.0320	0.2897 (ns↓)	0.9581 ± 0.0374	1.0000 (ns↓)	0.9603 ± 0.0304	0.4987 (ns↓)	0.9563 ± 0.0112	0.6195 (ns↓)
MambaMIL + HiLA-MIL (Ours)	0.9857 ± 0.0089	N/A	0.9638 ± 0.0162	N/A	0.9655 ± 0.0272	N/A	0.9581 ± 0.0457	N/A	0.9689 ± 0.0247	N/A	0.9607 ± 0.0184	N/A
GigaPath												
Methods	Overall											
	AUC	*P*-value	Accuracy	*P*-value	Precision	*P*-value	Recall	*P*-value	Specificity	*P*-value	F1-Score	*P*-value
ABMIL	0.9641 ± 0.0156	0.0234 (*↓)	0.8974 ± 0.0386	<0.0001 (***↓)	0.7911 ± 0.0837	0.0001 (***↓)	0.8051 ± 0.0668	0.0001 (***↓)	0.9676 ± 0.0109	0.0002 (***↓)	0.7789 ± 0.0855	0.0001 (***↓)
CLAM-SB	0.9659 ± 0.0163	0.0276 (*↓)	0.9095 ± 0.0306	<0.0001 (***↓)	0.8077 ± 0.0694	0.0132 (*↓)	0.8087 ± 0.0639	0.0034 (**↓)	0.9707 ± 0.0098	0.0094 (**↓)	0.8010 ± 0.0651	0.0115 (*↓)
CLAM-MB	0.9615 ± 0.0173	0.0030 (**↓)	0.9095 ± 0.0290	<0.0001 (***↓)	0.8047 ± 0.0690	0.0108 (*↓)	0.8067 ± 0.0657	0.0013 (**↓)	0.9707 ± 0.0093	0.0106 (*↓)	0.7987 ± 0.0661	0.0077 (**↓)
DSMIL	0.9645 ± 0.0156	0.0938 (ns↓)	0.9080 ± 0.0326	<0.0001 (***↓)	0.8196 ± 0.0747	0.0140 (*↓)	0.8183 ± 0.0550	0.0046 (**↓)	0.9701 ± 0.0106	0.0013 (**↓)	0.8053 ± 0.0648	0.0197 (*↓)
TransMIL	0.9640 ± 0.0180	0.0145 (*↓)	0.9064 ± 0.0367	<0.0001 (***↓)	0.8003 ± 0.1003	0.0472 (*↓)	0.8349 ± 0.0598	0.0929 (ns↓)	0.9693 ± 0.0119	0.0042 (**↓)	0.8038 ± 0.0845	0.0825 (ns↓)
MambaMIL	0.9651 ± 0.0166	0.0436 (*↓)	0.9170 ± 0.0311	0.0002 (***↓)	0.8364 ± 0.0646	0.0962 (ns↓)	0.8467 ± 0.0465	0.1581 (ns↓)	0.9736 ± 0.0093	0.0603 (ns↓)	0.8215 ± 0.0642	0.1356 (ns↓)
MambaMIL+HiLA-MIL (Ours)	0.9759 ± 0.0114	N/A	0.9291 ± 0.0307	N/A	0.8522 ± 0.0599	N/A	0.8678 ± 0.0560	N/A	0.9774 ± 0.0095	N/A	0.8468 ± 0.0610	N/A
Methods	Negative											
	AUC	*P*-value	Accuracy	*P*-value	Precision	*P*-value	Recall	*P*-value	Specificity	*P*-value	F1-Score	*P*-value
ABMIL	0.9989 ± 0.0025	0.2520 (ns↓)	0.9924 ± 0.0107	0.1226 (ns↓)	0.9958 ± 0.0132	0.1226 (ns↓)	0.9826 ± 0.0304	<0.0001 (***↓)	0.9977 ± 0.0074	1.0000 (ns↓)	0.9889 ± 0.0159	0.1226 (ns↓)
CLAM-SB	0.9985 ± 0.0032	0.2956 (ns↓)	0.9940 ± 0.0106	0.2508 (ns↓)	1.0000 ± 0.0000	1.0000 (ns↓)	0.9826 ± 0.0304	<0.0001 (***↓)	1.0000 ± 0.0000	1.0000 (ns↓)	0.9910 ± 0.0158	<0.0001 (***↓)
CLAM-MB	0.9969 ± 0.0056	0.2385 (ns↓)	0.9939 ± 0.0106	0.2484 (ns↓)	1.0000 ± 0.0000	1.0000 (ns↓)	0.9826 ± 0.0304	<0.0001 (***↓)	1.0000 ± 0.0000	1.0000 (ns↓)	0.9910 ± 0.0158	<0.0001 (***↓)
DSMIL	0.9992 ± 0.0020	0.6847 (ns↓)	0.9924 ± 0.0107	0.1250 (ns↓)	0.9920 ± 0.0253	0.1250 (ns↓)	0.9870 ± 0.0210	<0.0001 (***↓)	0.9953 ± 0.0147	0.4964 (ns↓)	0.9892 ± 0.0151	<0.0001 (***↓)
TransMIL	0.9984 ± 0.0019	0.0484 (*↓)	0.9910 ± 0.0105	<0.0001 (***↓)	1.0000 ± 0.0000	1.0000 (ns↓)	0.9739 ± 0.0304	<0.0001 (***↓)	1.0000 ± 0.0000	1.0000 (ns↓)	0.9866 ± 0.0158	<0.0001 (***↓)
MambaMIL	0.9994 ± 0.0019	0.4989 (ns↓)	0.9955 ± 0.0073	0.5006 (ns↓)	0.9958 ± 0.0132	0.5006 (ns↓)	0.9913 ± 0.0183	<0.0001 (***↓)	0.9977 ± 0.0072	1.0000 (ns↓)	0.9934 ± 0.0106	0.5006 (ns↓)
MambaMIL+HiLA-MIL (Ours)	1.0000 ± 0.0000	N/A	0.9985 ± 0.0048	N/A	1.0000 ± 0.0000	N/A	0.9957 ± 0.0137	N/A	1.0000 ± 0.0000	N/A	0.9978 ± 0.0070	N/A
Methods	ITC											
	AUC	*P*-value	Accuracy	*P*-value	Precision	*P*-value	Recall	*P*-value	Specificity	*P*-value	F1-Score	*P*-value
ABMIL	0.9513 ± 0.0272	0.0412 (*↓)	0.9396 ± 0.0327	0.0003 (***↓)	0.5657 ± 0.2062	0.0023 (**↓)	0.7750 ± 0.1845	<0.0001 (***↓)	0.9502 ± 0.0398	0.0374 (*↓)	0.6246 ± 0.1397	0.0003 (***↓)
CLAM-SB	0.9562 ± 0.0194	0.0899 (ns↓)	0.9472 ± 0.0227	0.1206 (ns↓)	0.5824 ± 0.1915	0.1525 (ns↓)	0.6750 ± 0.1687	<0.0001 (***↓)	0.9647 ± 0.0211	1.0000 (ns↑)	0.6119 ± 0.1504	0.0096 (**↓)
CLAM-MB	0.9469 ± 0.0326	0.0547 (ns↓)	0.9472 ± 0.0227	0.0942 (ns↓)	0.5824 ± 0.1915	0.1116 (ns↓)	0.6750 ± 0.1687	<0.0001 (***↓)	0.9647 ± 0.0211	1.0000 (ns↑)	0.6119 ± 0.1504	0.0092 (**↓)
DSMIL	0.9569 ± 0.0224	0.3805 (ns↓)	0.9517 ± 0.0211	0.2584 (ns↓)	0.6161 ± 0.1821	0.5711 (ns↓)	0.7250 ± 0.1845	<0.0001 (***↓)	0.9663 ± 0.0233	0.7894 (ns↑)	0.6483 ± 0.1374	0.1400 (ns↓)
TransMIL	0.9609 ± 0.0202	0.1952 (ns↓)	0.9547 ± 0.0201	0.8160 (ns↓)	0.6399 ± 0.1734	0.7790 (ns↑)	0.8250 ± 0.2058	0.2173 (ns↓)	0.9631 ± 0.0273	1.0000 (ns↑)	0.6915 ± 0.1132	0.5968 (ns↓)
MambaMIL	0.9630 ± 0.0148	0.4860 (ns↓)	0.9532 ± 0.0230	0.2681 (ns↓)	0.6356 ± 0.1871	0.3678 (ns↑)	0.8500 ± 0.1748	0.2463 (ns↓)	0.9599 ± 0.0305	0.3449 (ns↓)	0.6982 ± 0.1098	0.3267 (ns↓)
MambaMIL + HiLA-MIL (Ours)	0.9707 ± 0.0149	N/A	0.9578 ± 0.0220	N/A	0.6289 ± 0.1413	N/A	0.8750 ± 0.1318	N/A	0.9631 ± 0.0213	N/A	0.7226 ± 0.1177	N/A
Methods	Micro											
	AUC	*P*-value	Accuracy	*P*-value	Precision	*P*-value	Recall	*P*-value	Specificity	*P*-value	F1-Score	*P*-value
ABMIL	0.9168 ± 0.0413	0.0763 (ns↓)	0.9064 ± 0.0352	0.0012 (**↓)	0.6471 ± 0.1491	0.0028 (**↓)	0.5111 ± 0.2511	0.0039 (**↓)	0.9621 ± 0.0241	0.0501 (ns↓)	0.5493 ± 0.2134	0.0010 (**↓)
CLAM-SB	0.9249 ± 0.0418	0.1465 (ns↓)	0.9170 ± 0.0291	0.0152 (*↓)	0.6956 ± 0.1165	0.0113 (*↓)	0.6125 ± 0.1519	0.3915 (ns↓)	0.9603 ± 0.0216	0.0064 (**↓)	0.6431 ± 0.1214	0.0656 (ns↓)
CLAM-MB	0.9234 ± 0.0387	0.0789 (ns↓)	0.9155 ± 0.0265	0.0204 (*↓)	0.6839 ± 0.1022	0.0060 (**↓)	0.6014 ± 0.1604	0.2317 (ns↓)	0.9603 ± 0.0164	0.0054 (**↓)	0.6320 ± 0.1231	0.0311 (*↓)
DSMIL	0.9185 ± 0.0409	0.2037 (ns↓)	0.9155 ± 0.0346	0.0127 (*↓)	0.7150 ± 0.1841	0.0018 (**↓)	0.6097 ± 0.2059	0.3421 (ns↓)	0.9586 ± 0.0295	0.0009 (***↓)	0.6306 ± 0.1617	0.0283 (*↓)
TransMIL	0.9146 ± 0.0500	0.1034 (ns↓)	0.9155 ± 0.0349	0.0292 (*↓)	0.6141 ± 0.2501	0.0091 (**↓)	0.5889 ± 0.2985	0.2096 (ns↓)	0.9621 ± 0.0241	0.0342 (*↓)	0.5893 ± 0.2653	0.0444 (*↓)
MambaMIL	0.9119 ± 0.0463	0.0698 (ns↓)	0.9230 ± 0.0279	0.0972 (ns↓)	0.7528 ± 0.1378	0.0361 (*↓)	0.5875 ± 0.2224	0.1482 (ns↓)	0.9707 ± 0.0164	0.0291 (*↓)	0.6352 ± 0.1657	0.0760 (ns↓)
MambaMIL + HiLA-MIL (Ours)	0.9427 ± 0.0302	N/A	0.9351 ± 0.0254	N/A	0.8151 ± 0.1301	N/A	0.6361 ± 0.1566	N/A	0.9776 ± 0.0164	N/A	0.7024 ± 0.1225	N/A
Methods	Macro											
	AUC	*P*-value	Accuracy	*P*-value	Precision	*P*-value	Recall	*P*-value	Specificity	*P*-value	F1-Score	*P*-value
ABMIL	0.9895 ± 0.0096	0.8953 (ns↓)	0.9563 ± 0.0239	0.0218 (*↓)	0.9555 ± 0.0293	0.1117 (ns↓)	0.9516 ± 0.0487	0.2900 (ns↓)	0.9604 ± 0.0274	0.3822 (ns↓)	0.9527 ± 0.0268	0.0789 (ns↓)
CLAM-SB	0.9842 ± 0.0106	0.2009 (ns↓)	0.9608 ± 0.0237	0.1804 (ns↓)	0.9527 ± 0.0302	0.1514 (ns↓)	0.9645 ± 0.0355	1.0000 (ns↓)	0.9576 ± 0.0275	0.2929 (ns↓)	0.9582 ± 0.0255	0.4077 (ns↓)
CLAM-MB	0.9790 ± 0.0108	0.0005 (***↓)	0.9623 ± 0.0215	0.5805 (ns↓)	0.9527 ± 0.0259	0.1851 (ns↓)	0.9677 ± 0.0340	1.0000 (ns↑)	0.9577 ± 0.0236	0.2908 (ns↓)	0.9598 ± 0.0232	0.5065 (ns↓)
DSMIL	0.9835 ± 0.0091	0.2222 (ns↓)	0.9563 ± 0.0278	0.0116 (*↓)	0.9553 ± 0.0334	0.0981 (ns↓)	0.9516 ± 0.0409	0.2209 (ns↓)	0.9603 ± 0.0307	0.3709 (ns↓)	0.9530 ± 0.0303	0.0512 (ns↓)
TransMIL	0.9822 ± 0.0094	0.0036 (**↓)	0.9517 ± 0.0223	0.0018 (**↓)	0.9472 ± 0.0372	0.0070 (**↓)	0.9516 ± 0.0510	0.2136 (ns↓)	0.9521 ± 0.0348	0.0690 (ns↓)	0.9480 ± 0.0250	0.0044 (**↓)
MambaMIL	0.9859 ± 0.0084	0.1589 (ns↓)	0.9622 ± 0.0228	0.3780 (ns↓)	0.9615 ± 0.0247	0.4443 (ns↓)	0.9581 ± 0.0431	<0.0001 (***↓)	0.9660 ± 0.0224	1.0000 (ns↓)	0.9592 ± 0.0253	0.0634 (ns↓)
MambaMIL + HiLA-MIL (Ours)	0.9900 ± 0.0090	N/A	0.9668 ± 0.0185	N/A	0.9649 ± 0.0230	N/A	0.9645 ± 0.0355	N/A	0.9689 ± 0.0209	N/A	0.9642 ± 0.0204	N/A
Virchow												
Methods	Overall											
	AUC	*P*-value	Accuracy	*P*-value	Precision	*P*-value	Recall	*P*-value	Specificity	*P*-value	F1-Score	*P*-value
ABMIL	0.9559 ± 0.0127	<0.0001 (***↓)	0.8687 ± 0.0330	<0.0001 (***↓)	0.6805 ± 0.0664	<0.0001 (***↓)	0.6875 ± 0.0676	<0.0001 (***↓)	0.9592 ± 0.0091	0.0020 (**↓)	0.6760 ± 0.0620	<0.0001 (***↓)
CLAM-SB	0.9561 ± 0.0177	<0.0001 (***↓)	0.8732 ± 0.0347	<0.0001 (***↓)	0.6891 ± 0.0732	<0.0001 (***↓)	0.6939 ± 0.0653	<0.0001 (***↓)	0.9605 ± 0.0121	0.0056 (**↓)	0.6853 ± 0.0657	<0.0001 (***↓)
CLAM-MB	0.9621 ± 0.0127	0.0032 (**↓)	0.8853 ± 0.0350	<0.0001 (***↓)	0.7075 ± 0.0710	0.0002 (***↓)	0.7200 ± 0.0619	0.0004 (***↓)	0.9649 ± 0.0111	0.0550 (ns↓)	0.7067 ± 0.0632	0.0004 (***↓)
DSMIL	0.9591 ± 0.0117	0.0002 (***↓)	0.8732 ± 0.0319	<0.0001 (***↓)	0.7019 ± 0.0678	<0.0001 (***↓)	0.7013 ± 0.0684	<0.0001 (***↓)	0.9593 ± 0.0137	0.0015 (**↓)	0.6915 ± 0.0643	<0.0001 (***↓)
TransMIL	0.9555 ± 0.0220	0.0004 (***↓)	0.8838 ± 0.0253	<0.0001 (***↓)	0.7303 ± 0.0819	0.0009 (***↓)	0.7162 ± 0.0645	0.0003 (***↓)	0.9629 ± 0.0090	0.0120 (*↓)	0.7131 ± 0.0639	0.0005 (***↓)
MambaMIL	0.9601 ± 0.0133	0.0157 (*↓)	0.8913 ± 0.0362	0.0001 (***↓)	0.7733 ± 0.0728	0.0443 (*↓)	0.7545 ± 0.0561	0.0458 (*↓)	0.9650 ± 0.0110	0.0708 (ns↓)	0.7512 ± 0.0614	0.0419 (*↓)
MambaMIL + HiLA-MIL (Ours)	0.9695 ± 0.0131	N/A	0.9125 ± 0.0337	N/A	0.8149 ± 0.0763	N/A	0.8076 ± 0.0792	N/A	0.9714 ± 0.0122	N/A	0.7995 ± 0.0704	N/A
Methods	Negative											
	AUC	*P*-value	Accuracy	*P*-value	Precision	*P*-value	Recall	*P*-value	Specificity	*P*-value	F1-Score	*P*-value
ABMIL	0.9987 ± 0.0037	0.9929 (ns↑)	0.9970 ± 0.0063	0.3769 (ns↑)	0.9917 ± 0.0176	0.1225 (ns↑)	1.0000 ± 0.0000	1.0000 (ns↑)	0.9954 ± 0.0097	0.6249 (ns↑)	0.9957 ± 0.0090	0.0600 (ns↑
CLAM-SB	0.9984 ± 0.0051	0.7647 (ns↑)	0.9985 ± 0.0048	0.2166 (ns↑)	1.0000 ± 0.0000	0.0323 (*↑)	0.9957 ± 0.0137	1.0000 (ns↓)	1.0000 ± 0.0000	0.1280 (ns↑)	0.9978 ± 0.0070	0.1525 (ns↑)
CLAM-MB	1.0000 ± 0.0000	0.9208 (ns↑)	0.9970 ± 0.0063	0.2475 (ns↑)	0.9958 ± 0.0132	<0.0001 (***↓)	0.9957 ± 0.0137	1.0000 (ns↓)	0.9977 ± 0.0074	<0.0001 (***↓)	0.9957 ± 0.0092	<0.0001 (***↓)
DSMIL	0.9985 ± 0.0048	0.9311 (ns↑)	0.9970 ± 0.0064	0.3715 (ns↑)	0.9958 ± 0.0132	0.0627 (ns↑)	0.9957 ± 0.0137	1.0000 (ns↓)	0.9977 ± 0.0074	<0.0001 (***↓)	0.9957 ± 0.0092	0.2488 (ns↑)
TransMIL	0.9999 ± 0.0003	0.9257 (ns↑)	0.9955 ± 0.0073	0.6876 (ns↑)	0.9917 ± 0.0176	0.2463 (ns↑)	0.9957 ± 0.0137	1.0000 (ns↓)	0.9953 ± 0.0098	0.6178 (ns↑)	0.9935 ± 0.0104	0.6568 (ns↑)
MambaMIL	0.9951 ± 0.0113	0.3533 (ns↓)	0.9879 ± 0.0284	0.5549 (ns↓)	0.9751 ± 0.0653	0.4339 (ns↓)	0.9957 ± 0.0137	1.0000 (ns↓)	0.9837 ± 0.0439	0.5177 (ns↓)	0.9841 ± 0.0361	0.3481 (ns↓)
MambaMIL + HiLA-MIL (Ours)	0.9978 ± 0.0069	N/A	0.9924 ± 0.0192	N/A	0.9852 ± 0.0468	N/A	0.9957 ± 0.0137	N/A	0.9907 ± 0.0294	N/A	0.9898 ± 0.0255	N/A
Methods	ITC											
	AUC	*P*-value	Accuracy	*P*-value	Precision	*P*-value	Recall	*P*-value	Specificity	*P*-value	F1-Score	*P*-value
ABMIL	0.9229 ± 0.0340	<0.0001 (***↓)	0.9109 ± 0.0217	<0.0001 (***↓)	0.2400 ± 0.1725	<0.0001 (***↓)	0.3000 ± 0.2297	<0.0001 (***↓)	0.9502 ± 0.0220	0.0467 (*↓)	0.2644 ± 0.1928	<0.0001 (***↓)
CLAM-SB	0.9181 ± 0.0524	0.0003 (***↓)	0.9124 ± 0.0198	0.0001 (***↓)	0.2483 ± 0.1751	0.0001 (***↓)	0.3000 ± 0.2297	<0.0001 (***↓)	0.9518 ± 0.0201	0.0873 (ns↓)	0.2680 ± 0.1928	<0.0001 (***↓)
CLAM-MB	0.9283 ± 0.0201	0.0004 (***↓)	0.9185 ± 0.0189	0.0006 (***↓)	0.2712 ± 0.1846	0.0002 (***↓)	0.3250 ± 0.2648	<0.0001 (***↓)	0.9566 ± 0.0187	0.1904 (ns↓)	0.2915 ± 0.2131	<0.0001 (***↓)
DSMIL	0.9317 ± 0.0196	0.0016 (**↓)	0.9169 ± 0.0192	0.0001 (***↓)	0.3062 ± 0.1691	0.0001 (***↓)	0.3750 ± 0.2700	0.0001 (***↓)	0.9517 ± 0.0241	0.0544 (ns↓)	0.3229 ± 0.1894	0.0001 (***↓)
TransMIL	0.9247 ± 0.0423	0.0256 (*↓)	0.9291 ± 0.0187	0.0184 (*↓)	0.4000 ± 0.2600	0.0059 (**↓)	0.3250 ± 0.2058	<0.0001 (***↓)	0.9679 ± 0.0151	1.0000 (ns↓)	0.3457 ± 0.1944	0.0002 (***↓)
MambaMIL	0.9394 ± 0.0314	0.2551 (ns↓)	0.9335 ± 0.0259	0.1235 (ns↓)	0.5119 ± 0.2201	0.0964 (ns↓)	0.5250 ± 0.2189	0.0656 (ns↓)	0.9598 ± 0.0307	0.4150 (ns↓)	0.4844 ± 0.1629	0.0610 (ns↓)
MambaMIL + HiLA-MIL (Ours)	0.9510 ± 0.0207	N/A	0.9487 ± 0.0190	N/A	0.5783 ± 0.1796	N/A	0.6500 ± 0.2934	N/A	0.9679 ± 0.0212	N/A	0.5857 ± 0.1873	N/A
Methods	Micro											
	AUC	*P*-value	Accuracy	*P*-value	Precision	*P*-value	Recall	*P*-value	Specificity	*P*-value	F1-Score	*P*-value
ABMIL	0.9146 ± 0.0229	<0.0001 (***↓)	0.8792 ± 0.0331	0.0002 (***↓)	0.5337 ± 0.1523	0.0002 (***↓)	0.5111 ± 0.2230	0.0735 (ns↓)	0.9310 ± 0.0398	0.0007 (***↓)	0.4982 ± 0.1451	0.0035 (**↓)
CLAM-SB	0.9241 ± 0.0236	0.0008 (***↓)	0.8838 ± 0.0358	0.0010 (**↓)	0.5537 ± 0.1637	<0.0001 (***↓)	0.5347 ± 0.1926	0.1679 (ns↓)	0.9328 ± 0.0402	0.0003 (***↓)	0.5273 ± 0.1383	0.0173 (*↓)
CLAM-MB	0.9323 ± 0.0283	0.0945 (ns↓)	0.8959 ± 0.0359	0.0228 (*↓)	0.5977 ± 0.1439	0.0029 (**↓)	0.6111 ± 0.2158	0.6900 (ns↓)	0.9362 ± 0.0364	0.0009 (***↓)	0.5837 ± 0.1449	0.1271 (ns↓)
DSMIL	0.9213 ± 0.0305	0.0011 (**↓)	0.8853 ± 0.0293	0.0008 (***↓)	0.5614 ± 0.1486	0.0007 (***↓)	0.4861 ± 0.1797	0.0296 (*↓)	0.9414 ± 0.0374	0.0117 (*↓)	0.5031 ± 0.1334	0.0052 (**↓)
TransMIL	0.9146 ± 0.0362	0.0002 (***↓)	0.8898 ± 0.0252	0.0016 (**↓)	0.5748 ± 0.1091	0.0005 (***↓)	0.5958 ± 0.1966	0.4761 (ns↓)	0.9310 ± 0.0345	0.0009 (***↓)	0.5630 ± 0.0949	0.0302 (*↓)
MambaMIL	0.9219 ± 0.0337	0.0244 (*↓)	0.9034 ± 0.0349	0.1232 (ns↓)	0.6467 ± 0.1780	0.1212 (ns↓)	0.5458 ± 0.1602	0.1552 (ns↓)	0.9534 ± 0.0326	0.3277 (ns↓)	0.5820 ± 0.1473	0.1091 (ns↓)
MambaMIL + HiLA-MIL (Ours)	0.9439 ± 0.0323	N/A	0.9215 ± 0.0298	N/A	0.7367 ± 0.1773	N/A	0.6236 ± 0.1394	N/A	0.9638 ± 0.0275	N/A	0.6628 ± 0.1162	N/A
Methods	Macro											
	AUC	*P*-value	Accuracy	*P*-value	Precision	*P*-value	Recall	*P*-value	Specificity	*P*-value	F1-Score	*P*-value
ABMIL	0.9875 ± 0.0099	0.3669 (ns ↑ )	0.9502 ± 0.0225	0.1000 (ns↓)	0.9565 ± 0.0350	0.6227 (ns↓)	0.9387 ± 0.0653	0.0356 (*↓)	0.9603 ± 0.0334	1.0000 (ns↓)	0.9455 ± 0.0267	0.1065 (ns↓)
CLAM-SB	0.9837 ± 0.0116	0.1442 (ns↓)	0.9517 ± 0.0283	0.1090 (ns↓)	0.9545 ± 0.0514	0.4800 (ns↓)	0.9452 ± 0.0457	0.0376 (*↓)	0.9575 ± 0.0524	0.8047 (ns↓)	0.9483 ± 0.0292	0.1163 (ns↓)
CLAM-MB	0.9879 ± 0.0128	0.9951 (ns↑)	0.9593 ± 0.0235	0.5242 (ns↓)	0.9651 ± 0.0344	0.7131 (ns↑)	0.9484 ± 0.0435	0.1095 (ns↓)	0.9690 ± 0.0308	0.7766 (ns↑)	0.9558 ± 0.0261	0.5361 (ns↓)
DSMIL	0.9847 ± 0.0124	0.4282 (ns↓)	0.9472 ± 0.0392	0.0891 (ns↓)	0.9442 ± 0.0655	0.1221 (ns↓)	0.9483 ± 0.0461	0.1085 (ns↓)	0.9463 ± 0.0703	0.2354 (ns↓)	0.9444 ± 0.0390	0.0522 (ns↓)
TransMIL	0.9827 ± 0.0175	0.2248 (ns↓)	0.9532 ± 0.0241	0.1363 (ns↓)	0.9545 ± 0.0483	0.4586 (ns↓)	0.9484 ± 0.0435	0.1057 (ns↓)	0.9574 ± 0.0490	0.7758 (ns↓)	0.9500 ± 0.0252	0.2044 (ns↓)
MambaMIL	0.9839 ± 0.0120	0.2467 (ns↓)	0.9577 ± 0.0234	0.6674 (ns↓)	0.9594 ± 0.0346	0.8948 (ns↓)	0.9516 ± 0.0532	0.5532 (ns↓)	0.9633 ± 0.0326	1.0000 (ns↓)	0.9542 ± 0.0266	0.5088 (ns↓)
MambaMIL + HiLA-MIL (Ours)	0.9854 ± 0.0100	N/A	0.9623 ± 0.0236	N/A	0.9595 ± 0.0382	N/A	0.9613 ± 0.0333	N/A	0.9633 ± 0.0350	N/A	0.9598 ± 0.0248	N/A

*: *P* < 0.05；**: *P* < 0.01；***: *P* < 0.001；*ns* no statistical significance. ↑: an increase in metrics of the baseline models compared with the MambaMIL + HiLA-MIL model. ↓: a decrease in metrics of the baseline models compared with the MambaMIL + HiLA-MIL model. *N/A* Not applicable. Statistical comparison was not performed. A two-sided paired permutation test with 10,000 permutations was applied.

With the ResNet feature extractor, MambaMIL + HiLA-MIL obtained an AUC of 0.9700 ± 0.0140, accuracy of 0.9019 ± 0.0318, precision of 0.8193 ± 0.0661, recall of 0.8029 ± 0.0518, specificity of 0.9657 ± 0.0111, and F1-score of 0.7993 ± 0.0547. The improvements in AUC, accuracy, and recall were statistically significant against all baselines, with particularly large gains in recall (+4.73%) and F1-score (+4.55%) over the best-performing baseline (CLAM-SB).

With the UNI feature extractor, our model achieved an AUC of 0.9710 ± 0.0105, accuracy of 0.9290 ± 0.0256, precision of 0.8722 ± 0.0646, recall of 0.8576 ± 0.0501, specificity of 0.9772 ± 0.0072, and F1-score of 0.8455 ± 0.0470. The improvements in accuracy, precision, recall, and F1-score were statistically significant compared to all baselines, with substantial gains in precision (+5.76%) over the best-performing baseline (TransMIL), as well as recall (+5.49%) and F1-score (+5.63%) over the best-performing baseline (MambaMIL).

With the GigaPath feature extractor, MambaMIL + HiLA-MIL attained an AUC of 0.9759 ± 0.0114, accuracy of 0.9291 ± 0.0307, precision of 0.8522 ± 0.0599, recall of 0.8678 ± 0.0560, specificity of 0.9774 ± 0.0095, and F1-score of 0.8468 ± 0.0610. The improvements in accuracy were statistically significant against all baselines, with remarkable gains in recall (+2.11%) and F1-score (+2.53%) over the best-performing baseline (MambaMIL).

With the Virchow feature extractor, our model exhibited an AUC of 0.9695 ± 0.0131, accuracy of 0.9125 ± 0.0337, precision of 0.8149 ± 0.0763, recall of 0.8076 ± 0.0792, specificity of 0.9714 ± 0.0122, and F1-score of 0.7995 ± 0.0704. The improvements in all metrics except specificity were statistically significant against all baselines, with prominent gains in precision (+4.16%), recall (+5.31%), and F1-score (+4.83%) over the best-performing baseline (MambaMIL).

Besides the overall four-classification, each LNM category task was also investigated. The model achieved statistically significant improvements in all evaluation metrics for the identification of ITCs and micro-metastasis, with marked enhancements in accuracy, recall, and F1-score. For negative and macro-metastasis classification, the model maintained competitive and leading performance across all metrics, while the statistical differences compared with baseline models were relatively limited. Overall, the proposed model delivered balanced and promising performance in tasks for individual LNM categories, and notably improved the discriminative ability for clinically challenging minimal metastatic lesions.

**Fig. 1:**
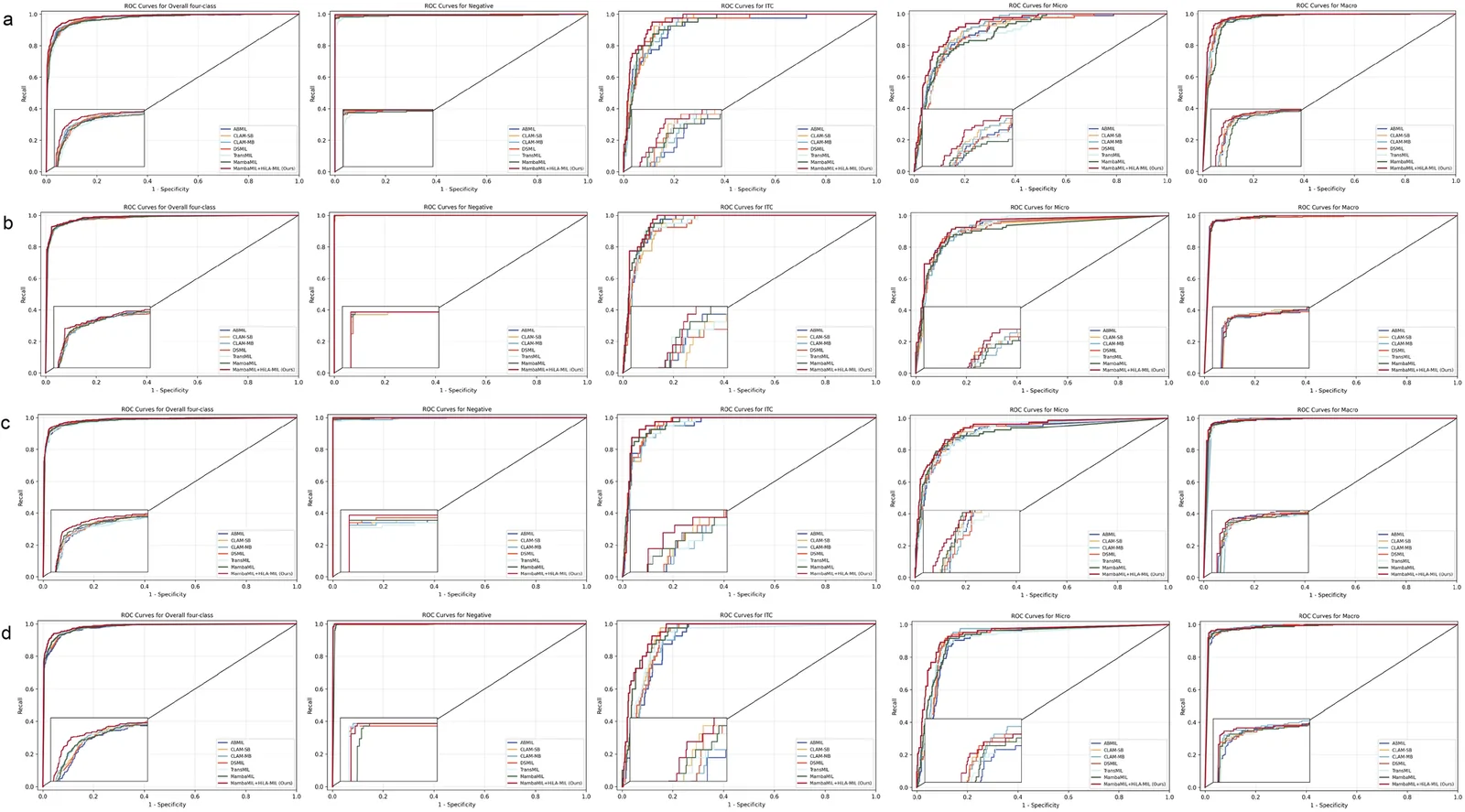
Four‑class LNM classification ROC analysis on the NIMM dataset with four feature extractors. ROC plots of the six baseline models as well as the MambaMIL + HiLA-MIL model for overall four-class classification and individual LNM categories across **a** ResNet, **b** UNI, **c** GigaPath, and **d** Virchow feature extractors. Source data are provided as a Source Data file.

The ROC results based on four feature extractors (Fig. 1) indicated that the MambaMIL+HiLA-MIL model achieves a significantly superior performance over six baseline models in the overall four-class classification task, as well as in identifying clinically challenging ITCs and micro-metastasis. For the negative and macro-metastasis categories with relatively low diagnostic difficulty, our model still retained a slight yet steady performance advantage over all baseline models. In conclusion, these results further demonstrate that the proposed framework possesses favorable robustness and generalization capability, and can remarkably boost the discriminative performance for clinically critical minimal metastatic lesions in pathological diagnosis.

To further validate the robustness of the MambaMIL + HiLA-MIL model, further experiments were conducted using the DLZX and YDSY datasets as external validation cohorts. Detailed results are presented in Supplementary Tables 1 and 2. On the DLZX and YDSY datasets, although the numerical values of all performance metrics were slightly lower compared with those on the NIMM dataset, the MambaMIL + HiLA-MIL model across all four feature extractors comprehensively outperformed all baseline models in overall four-class classification. In particular, the improvements in accuracy, recall, and F1-score were statistically significant.

Likewise, we further explored the performance of MambaMIL + HiLA-MIL in each LNM category task across two external validation datasets. The overall performance of the model on this task was generally consistent with its performance on the internal NIMM dataset. In the ITCs and micro-metastasis classification tasks, the proposed model significantly outperformed the baseline models across all evaluation metrics, with particularly prominent improvements in recall (DLZX and YDSY datasets) and F1-score (YDSY dataset). In the classification of negative samples, the model achieved statistically significant improvements in multiple metrics under three feature extractors, excluding Virchow. For the macro-metastasis classification task, except for the GigaPath feature extractor, the model yielded statistically significant elevations in multiple evaluation metrics compared with the baseline models under the other three feature extractors.

The ROC results (Supplementary Figs. 1 and 2) further confirmed that the proposed MambaMIL + HiLA-MIL model achieved superior discriminative performance over all baseline methods in both the overall four-class classification and individual LNM category, including ITCs and micro-metastasis. Although the improvements in some AUC values did not reach statistical significance, the overall discriminative advantage of the model was evident.

### Comparison of multi-cancer and single-cancer

To comprehensively evaluate the cross-cancer diagnostic performance of the model, the six baseline models and our MambaMIL + HiLA-MIL model were also applied to discriminate multi-cancer and five individual cancer types on the NIMM dataset, including gastric adenocarcinoma, colorectal adenocarcinoma, lung adenocarcinoma, breast invasive ductal carcinoma, and breast invasive lobular carcinoma. Thyroid papillary carcinoma and female genital endometrioid adenocarcinoma were excluded due to limited sample sizes. The detailed cross-cancer diagnostic performance results of our model under GigaPath are summarized in Table 2.

**Table 2:** Evaluation metrics of multi-cancer and individual cancer types on NIMM, DLZX, and YDSY Datasets under GigaPath

NIMM												
Methods	Multi-cancer											
	AUC	*P*-value	Accuracy	*P*-value	Precision	*P*-value	Recall	*P*-value	Specificity	*P*-value	F1-Score	*P*-value
ABMIL	0.9651 ± 0.0040	0.0872 (ns↓)	0.8554 ± 0.0190	<0.0001 (***↓)	0.5517 ± 0.0228	0.0073 (**↓)	0.5452 ± 0.0239	0.0001 (***↓)	0.9146 ± 0.0157	0.0001 (***↓)	0.5403 ± 0.0224	0.0014 (**↓)
CLAM-SB	0.9580 ± 0.0251	0.0135 (*↓)	0.8488 ± 0.0265	<0.0001 (***↓)	0.5581 ± 0.0048	0.0166 (*↓)	0.5418 ± 0.0195	0.0005 (***↓)	0.9215 ± 0.0183	<0.0001 (***↓)	0.5387 ± 0.0178	0.0025 (**↓)
CLAM-MB	0.9651 ± 0.0046	0.1357 (ns↓)	0.8589 ± 0.0158	<0.0001 (***↓)	0.5679 ± 0.0308	0.0061 (**↓)	0.5639 ± 0.0308	0.0107 (*↓)	0.9246 ± 0.0258	0.0002 (***↓)	0.5460 ± 0.0207	0.0009 (***↓)
DSMIL	0.9667 ± 0.0042	0.2010 (ns↓)	0.8566 ± 0.0196	<0.0001 (***↓)	0.5733 ± 0.0296	0.0003 (***↓)	0.5598 ± 0.0272	<0.0001 (***↓)	0.9223 ± 0.0287	<0.0001 (***↓)	0.5542 ± 0.0216	0.0001 (***↓)
TransMIL	0.9668 ± 0.0115	0.0569 (ns↓)	0.8408 ± 0.0328	<0.0001 (***↓)	0.5429 ± 0.0027	<0.0001 (***↓)	0.5552 ± 0.0125	0.0004 (***↓)	0.9233 ± 0.0296	0.0006 (***↓)	0.5302 ± 0.0083	<0.0001 (***↓)
MambaMIL	0.9747 ± 0.0073	0.0315 (*↓)	0.8661 ± 0.0187	<0.0001 (***↓)	0.5984 ± 0.0768	0.0121 (*↓)	0.5980 ± 0.0576	0.0109 (*↓)	0.9320 ± 0.0232	0.0027 (**↓)	0.5825 ± 0.0676	0.0053 (**↓)
MambaMIL + HiLA-MIL (Ours)	0.9839 ± 0.0066	N/A	0.8964 ± 0.0294	N/A	0.6257 ± 0.0598	N/A	0.6210 ± 0.0655	N/A	0.9355 ± 0.0302	N/A	0.6139 ± 0.0614	N/A
Methods	Gastric adenocarcinoma											
	AUC	*P*-value	Accuracy	*P*-value	Precision	P-value	Recall	*P*-value	Specificity	*P*-value	F1-Score	*P*-value
ABMIL	0.9866 ± 0.0117	0.9994 (ns↓)	0.9237 ± 0.0451	0.3802 (ns↓)	0.5248 ± 0.0727	0.7852 (ns↓)	0.5364 ± 0.0713	0.6315 (ns↓)	0.9700 ± 0.0133	0.2509 (ns↓)	0.5289 ± 0.0693	0.7243 (ns↓)
CLAM-SB	0.9916 ± 0.0065	0.9039 (ns↑)	0.9080 ± 0.0514	0.0164 (*↓)	0.5212 ± 0.0822	0.4956 (ns↓)	0.5327 ± 0.0901	0.5363 (ns↓)	0.9682 ± 0.0150	0.1112 (ns↓)	0.5254 ± 0.0846	0.5591 (ns↓)
CLAM-MB	0.9891 ± 0.0107	0.9527 (ns↓)	0.9172 ± 0.0509	<0.0001 (***↓)	0.4841 ± 0.0076	0.3657 (ns↓)	0.4868 ± 0.0156	0.1266 (ns↓)	0.9723 ± 0.0154	0.2819 (ns↓)	0.4852 ± 0.0064	0.3356 (ns↓)
DSMIL	0.9890 ± 0.0109	0.9577 (ns↓)	0.9237 ± 0.0451	0.3802 (ns↓)	0.5232 ± 0.0739	0.7540 (ns↓)	0.5364 ± 0.0713	0.6315 (ns↓)	0.9715 ± 0.0160	0.2829 (ns↓)	0.5281 ± 0.0700	0.7243 (ns↓)
TransMIL	0.9933 ± 0.0068	0.8538 (ns↑)	0.9172 ± 0.0509	0.0292 (*↓)	0.5352 ± 0.0777	0.9110 (ns↓)	0.5366 ± 0.0715	0.5607 (ns↓)	0.9735 ± 0.0105	0.6904 (ns↓)	0.5344 ± 0.0721	0.9110 (ns↓)
MambaMIL	0.9907 ± 0.0083	0.5443 (ns↓)	0.9324 ± 0.0607	1.0000 (ns↑)	0.6001 ± 0.1387	0.9381 (ns↓)	0.6215 ± 0.1251	0.6249 (ns↑)	0.9771 ± 0.0200	0.8457 (ns↓)	0.6057 ± 0.1330	0.7195 (ns↓)
MambaMIL + HiLA-MIL (Ours)	0.9910 ± 0.0082	N/A	0.9313 ± 0.0644	N/A	0.6189 ± 0.1298	N/A	0.5977 ± 0.1379	N/A	0.9777 ± 0.0205	N/A	0.6068 ± 0.1335	N/A
Methods	Colorectal adenocarcinoma											
	AUC	*P*-value	Accuracy	*P*-value	Precision	*P*-value	Recall	*P*-value	Specificity	*P*-value	F1-Score	*P*-value
ABMIL	0.9895 ± 0.0112	0.9147 (ns↓)	0.9212 ± 0.0692	<0.0001 (***↓)	0.6343 ± 0.1206	0.8191 (ns↓)	0.6125 ± 0.1068	0.5035 (ns↓)	0.9728 ± 0.0209	0.0570 (ns↓)	0.6091 ± 0.0946	0.6961 (ns↓)
CLAM-SB	0.9928 ± 0.0042	0.6274 (ns↓)	0.9252 ± 0.0464	0.0148 (*↓)	0.5901 ± 0.1134	0.9155 (ns↓)	0.5659 ± 0.0596	0.5003 (ns↓)	0.9755 ± 0.0150	0.1929 (ns↓)	0.5698 ± 0.0725	0.5277 (ns↓)
CLAM-MB	0.9919 ± 0.0119	0.8383 (ns↓)	0.9489 ± 0.0394	0.2530 (ns↑)	0.7364 ± 0.2057	0.4999 (ns↑)	0.7188 ± 0.1952	0.4999 (ns↑)	0.9859 ± 0.0120	1.0000 (ns↑)	0.6796 ± 0.1682	0.4999 (ns ↑ )
DSMIL	0.9914 ± 0.0128	0.6180 (ns↓)	0.9277 ± 0.0755	<0.0001 (***↓)	0.6815 ± 0.1878	0.9709 (ns↓)	0.6958 ± 0.1881	0.9402 (ns↑)	0.9784 ± 0.0249	0.2840 (ns↓)	0.6675 ± 0.1667	0.9028 (ns ↑ )
TransMIL	0.9917 ± 0.0112	0.6332 (ns↓)	0.9254 ± 0.0463	<0.0001 (***↓)	0.5463 ± 0.0424	<0.0001 (***↓)	0.6292 ± 0.1312	<0.0001 (***↓)	0.9807 ± 0.0125	0.2519 (ns↓)	0.5645 ± 0.0657	<0.0001 (***↓)
MambaMIL	0.9965 ± 0.0052	0.7711 (ns↑)	0.9553 ± 0.0438	0.1192 (ns↑)	0.7846 ± 0.0783	0.2455 (ns↑)	0.7494 ± 0.1250	0.2449 (ns↑)	0.9867 ± 0.0106	0.4349 (ns↑)	0.7369 ± 0.0877	0.3062 (ns ↑ )
MambaMIL + HiLA-MIL (Ours)	0.9957 ± 0.0058	N/A	0.9436 ± 0.0483	N/A	0.6873 ± 0.1875	N/A	0.6910 ± 0.1876	N/A	0.9844 ± 0.0145	N/A	0.6635 ± 0.1668	N/A
Methods	Lung adenocarcinoma											
	AUC	*P*-value	Accuracy	*P*-value	Precision	*P*-value	Recall	*P*-value	Specificity	*P*-value	F1-Score	*P*-value
ABMIL	0.9779 ± 0.0162	0.6258 (ns↓)	0.9147 ± 0.0508	0.0288 (*↓)	0.6569 ± 0.1731	0.4611 (ns↓)	0.6349 ± 0.1172	0.1215 (ns↓)	0.9683 ± 0.0195	0.2753 (ns↓)	0.6339 ± 0.1282	0.0909 (ns↓)
CLAM-SB	0.9711 ± 0.0250	0.3784 (ns↓)	0.9027 ± 0.0563	<0.0001 (***↓)	0.7419 ± 0.1107	0.9960 (ns↑)	0.6709 ± 0.0900	0.0336 (*↓)	0.9605 ± 0.0276	0.0334 (*↓)	0.6816 ± 0.0761	0.3408 (ns↓)
CLAM-MB	0.9873 ± 0.0137	0.6840 (ns↓)	0.9103 ± 0.0548	<0.0001 (***↓)	0.6599 ± 0.1686	0.0634 (ns↓)	0.6357 ± 0.1388	0.0634 (ns↓)	0.9691 ± 0.0225	0.1262 (ns↓)	0.6361 ± 0.1345	0.0634 (ns↓)
DSMIL	0.9846 ± 0.0106	0.7211 (ns↓)	0.8992 ± 0.0387	<0.0001 (***↓)	0.6599 ± 0.1686	<0.0001 (***↓)	0.6273 ± 0.1303	<0.0001 (***↓)	0.9663 ± 0.0186	<0.0001 (***↓)	0.6317 ± 0.1319	<0.0001 (***↓)
TransMIL	0.9695 ± 0.0404	0.1891 (ns↓)	0.8750 ± 0.0969	0.0006 (***↓)	0.6039 ± 0.1547	0.1039 (ns↓)	0.6432 ± 0.1387	0.2748 (ns↓)	0.9612 ± 0.0208	0.1590 (ns↓)	0.5989 ± 0.1556	0.1762 (ns↓)
MambaMIL	0.9847 ± 0.0086	0.2459 (ns↓)	0.8910 ± 0.0706	<0.0001 (***↓)	0.5840 ± 0.0533	0.1127 (ns↓)	0.6443 ± 0.0676	0.0248 (*↓)	0.9609 ± 0.0270	0.0517 (ns↓)	0.6047 ± 0.0458	0.0540 (ns↓)
MambaMIL + HiLA-MIL (Ours)	0.9930 ± 0.0044	N/A	0.9339 ± 0.0311	N/A	0.7090 ± 0.1629	N/A	0.7051 ± 0.0914	N/A	0.9777 ± 0.0126	N/A	0.6958 ± 0.1147	N/A
Methods	Breast invasive ductal carcinoma											
	AUC	*P*-value	Accuracy	*P*-value	Precision	*P*-value	Recall	*P*-value	Specificity	*P*-value	F1-Score	*P*-value
ABMIL	0.9292 ± 0.0174	0.0561 (ns↓)	0.7560 ± 0.0663	<0.0001 (***↓)	0.7304 ± 0.0451	0.0060 (**↓)	0.7090 ± 0.0775	0.0009 (***↓)	0.9145 ± 0.0244	0.0010 (**↓)	0.7103 ± 0.0610	0.0037 (**↓)
CLAM-SB	0.9201 ± 0.0388	0.0036 (**↓)	0.7463 ± 0.0773	<0.0001 (***↓)	0.7026 ± 0.0636	0.0035 (**↓)	0.7061 ± 0.0565	0.0066 (**↓)	0.9144 ± 0.0240	0.0055 (**↓)	0.6843 ± 0.0732	0.0044 (**↓)
CLAM-MB	0.9196 ± 0.0307	0.0296 (*↓)	0.7287 ± 0.0697	<0.0001 (***↓)	0.6502 ± 0.0873	0.0080 (**↓)	0.7239 ± 0.0144	0.0190 (*↓)	0.9137 ± 0.0182	0.0054 (**↓)	0.6681 ± 0.0623	0.0029 (**↓)
DSMIL	0.9287 ± 0.0153	0.0808 (ns↓)	0.7432 ± 0.0464	<0.0001 (***↓)	0.6930 ± 0.0157	0.0001 (***↓)	0.6850 ± 0.0569	<0.0001 (***↓)	0.9132 ± 0.0189	0.0006 (***↓)	0.6826 ± 0.0371	<0.0001 (***↓)
TransMIL	0.9305 ± 0.0094	0.0457 (*↓)	0.7247 ± 0.0392	<0.0001 (***↓)	0.7107 ± 0.0443	0.0012 (**↓)	0.7231 ± 0.0045	0.0024 (**↓)	0.9124 ± 0.0149	0.0011 (**↓)	0.6931 ± 0.0190	0.0002 (***↓)
MambaMIL	0.9435 ± 0.0113	0.1036 (ns↓)	0.7348 ± 0.0460	<0.0001 (***↓)	0.7077 ± 0.0280	0.0018 (**↓)	0.6998 ± 0.0384	0.0015 (**↓)	0.9122 ± 0.0154	0.0010 (**↓)	0.6782 ± 0.0540	0.0006 (***↓)
MambaMIL + HiLA-MIL (Ours)	0.9575 ± 0.0131	N/A	0.8285 ± 0.0518	N/A	0.8042 ± 0.0489	N/A	0.8154 ± 0.0536	N/A	0.9419 ± 0.0198	N/A	0.8043 ± 0.0460	N/A
Methods	Breast invasive lobular carcinoma											
	AUC	*P*-value	Accuracy	*P*-value	Precision	*P*-value	Recall	*P*-value	Specificity	*P*-value	F1-Score	*P*-value
ABMIL	0.9422 ± 0.0336	0.5684 (ns↓)	0.7616 ± 0.0827	<0.0001 (***↓)	0.2121 ± 0.0347	<0.0001 (***↓)	0.2333 ± 0.0289	<0.0001 (***↓)	0.7472 ± 0.0459	<0.0001 (***↓)	0.2192 ± 0.0077	<0.0001 (***↓)
CLAM-SB	0.9143 ± 0.0732	0.3179 (ns↓)	0.7616 ± 0.0827	<0.0001 (***↓)	0.2348 ± 0.0262	0.0626 (ns↓)	0.2333 ± 0.0289	<0.0001 (***↓)	0.7889 ± 0.1134	0.9379 (ns↓)	0.2324 ± 0.0153	0.0626 (ns↓)
CLAM-MB	0.9378 ± 0.0308	0.5766 (ns↓)	0.7894 ± 0.0353	<0.0001 (***↓)	0.3089 ± 0.1432	0.4991 (ns↓)	0.2542 ± 0.0564	<0.0001 (***↓)	0.7819 ± 0.1017	0.5037 (ns↓)	0.2608 ± 0.0645	0.2500 (ns↓)
DSMIL	0.9400 ± 0.0347	0.5592 (ns↓)	0.7894 ± 0.0353	<0.0001 (***↓)	0.3089 ± 0.1432	0.1266 (ns↓)	0.2542 ± 0.0564	<0.0001 (***↓)	0.7819 ± 0.1017	0.5037 (ns↓)	0.2608 ± 0.0645	0.1266 (ns↓)
TransMIL	0.9488 ± 0.0321	0.4023 (ns↓)	0.7616 ± 0.0827	<0.0001 (***↓)	0.3182 ± 0.1591	0.1266 (ns↑)	0.2437 ± 0.0410	<0.0001 (***↓)	0.7889 ± 0.1134	0.9371 (ns↓)	0.2602 ± 0.0634	0.0630 (ns↓)
MambaMIL	0.9580 ± 0.0248	0.7515 (ns↓)	0.8172 ± 0.0167	<0.0001 (***↓)	0.3157 ± 0.1361	0.2569 (ns↑)	0.2750 ± 0.0901	<0.0001 (***↓)	0.8230 ± 0.1113	0.7536 (ns↑)	0.2870 ± 0.0998	0.2569 (ns↓)
MambaMIL + HiLA-MIL (Ours)	0.9823 ± 0.0135	N/A	0.8449 ± 0.0628	N/A	0.3089 ± 0.1432	N/A	0.2958 ± 0.1252	N/A	0.7958 ± 0.1252	N/A	0.2989 ± 0.1305	N/A
DLZX												
Methods	Multi-cancer											
	AUC	*P*-value	Accuracy	*P*-value	Precision	*P*-value	Recall	*P*-value	Specificity	*P*-value	F1-Score	*P*-value
ABMIL	0.9417 ± 0.0111	0.1187 (ns↓)	0.8181 ± 0.0335	<0.0001 (***↓)	0.4723 ± 0.0048	0.2436 (ns↓)	0.4570 ± 0.0272	0.1057 (ns↓)	0.9036 ± 0.0261	0.0322 (*↓)	0.4472 ± 0.0189	0.0911 (ns↓)
CLAM-SB	0.9492 ± 0.0191	0.2693 (ns↓)	0.8456 ± 0.0697	0.0193 (*↓)	0.4995 ± 0.0333	0.2461 (ns↓)	0.4819 ± 0.0074	0.1471 (ns↓)	0.9108 ± 0.0362	0.1205 (ns↓)	0.4692 ± 0.0207	0.1834 (ns↓)
CLAM-MB	0.9339 ± 0.0182	0.0975 (ns↓)	0.7963 ± 0.0464	<0.0001 (***↓)	0.4761 ± 0.0218	0.0599 (ns↓)	0.4441 ± 0.0085	0.0409 (*↓)	0.8994 ± 0.0367	0.0520 (ns↓)	0.4380 ± 0.0215	0.0311 (*↓)
DSMIL	0.9551 ± 0.0146	0.4330 (ns↓)	0.8142 ± 0.0410	<0.0001 (***↓)	0.4527 ± 0.0740	0.1310 (ns↓)	0.4417 ± 0.0505	0.0032 (**↓)	0.9041 ± 0.0332	0.0129 (*↓)	0.4297 ± 0.0601	0.0121 (*↓)
TransMIL	0.9538 ± 0.0180	0.2847 (ns↓)	0.8285 ± 0.0530	0.0024 (**↓)	0.4613 ± 0.0362	0.1856 (ns↓)	0.4352 ± 0.0381	0.0141 (*↓)	0.9045 ± 0.0360	0.0343 (*↓)	0.4383 ± 0.0446	0.0373 (*↓)
MambaMIL	0.9576 ± 0.0157	0.5967 (ns↑)	0.8486 ± 0.0204	0.0387 (*↓)	0.4825 ± 0.0244	0.4608 (ns↓)	0.4552 ± 0.0232	0.0192 (*↓)	0.9179 ± 0.0282	0.0675 (ns↓)	0.4577 ± 0.0230	0.0718 (ns↓)
MambaMIL + HiLA-MIL (Ours)	0.9560 ± 0.0140	N/A	0.8507 ± 0.0219	N/A	0.5402 ± 0.0472	N/A	0.5074 ± 0.0112	N/A	0.9207 ± 0.0257	N/A	0.5101 ± 0.0232	N/A
Methods	Gastric adenocarcinoma											
	AUC	*P*-value	Accuracy	*P*-value	Precision	*P*-value	Recall	*P*-value	Specificity	*P*-value	F1-Score	*P*-value
ABMIL	0.9052 ± 0.0408	0.5384 (ns↓)	0.7994 ± 0.0134	<0.0001 (***↑)	0.5972 ± 0.0060	0.6307 (ns↑)	0.6174 ± 0.0445	0.1303 (ns↑)	0.9260 ± 0.0098	0.4772 (ns↑)	0.5805 ± 0.0104	0.4429 (ns↑)
CLAM-SB	0.9036 ± 0.0529	0.7237 (ns↓)	0.8008 ± 0.0598	0.0321 (*↑)	0.6291 ± 0.0619	0.6709 (ns↑)	0.6125 ± 0.0836	0.2511 (ns↑)	0.9281 ± 0.0187	0.5326 (ns↑)	0.5866 ± 0.0604	0.4376 (ns↑)
CLAM-MB	0.8816 ± 0.0226	0.9719 (ns↓)	0.7772 ± 0.0403	0.2548 (ns↑)	0.6138 ± 0.0440	0.9368 (ns↑)	0.5943 ± 0.0539	0.2548 (ns↑)	0.9264 ± 0.0057	0.4417 (ns↑)	0.5615 ± 0.0216	0.5590 (ns↑)
DSMIL	0.9319 ± 0.0423	0.9717 (ns↑)	0.7802 ± 0.0672	0.4602 (ns↑)	0.4923 ± 0.1387	0.8523 (ns↓)	0.5056 ± 0.1262	0.8191 (ns↓)	0.9092 ± 0.0321	0.8654 (ns↓)	0.4882 ± 0.1256	0.9467 (ns↓)
TransMIL	0.9430 ± 0.0498	0.9052 (ns↑)	0.8024 ± 0.0536	<0.0001 (***↑)	0.5786 ± 0.0815	0.9686 (ns↑)	0.5674 ± 0.0535	0.2430 (ns↑)	0.9224 ± 0.0169	0.5321 (ns↑)	0.5658 ± 0.0651	0.5971 (ns↑)
MambaMIL	0.9342 ± 0.0497	0.3614 (ns↑)	0.8024 ± 0.0536	<0.0001 (***↑)	0.5633 ± 0.1192	0.7541 (ns↓)	0.5611 ± 0.1262	<0.0001 (***↑)	0.9199 ± 0.0278	0.5041 (ns↑)	0.5429 ± 0.1167	0.2543 (ns↑)
MambaMIL + HiLA-MIL (Ours)	0.9119 ± 0.0415	N/A	0.7577 ± 0.0612	N/A	0.5675 ± 0.1122	N/A	0.5229 ± 0.1326	N/A	0.9139 ± 0.0224	N/A	0.5164 ± 0.0970	N/A
Methods	Colorectal adenocarcinoma											
	AUC	*P*-value	Accuracy	*P*-value	Precision	*P*-value	Recall	*P*-value	Specificity	*P*-value	F1-Score	*P*-value
ABMIL	0.9483 ± 0.0307	0.5862 (ns↓)	0.8596 ± 0.0290	<0.0001 (***↓)	0.5955 ± 0.1142	0.0651 (ns↓)	0.5532 ± 0.1224	0.1309 (ns↓)	0.9538 ± 0.0074	0.0629 (ns↓)	0.5431 ± 0.0788	0.0374 (*↓)
CLAM-SB	0.9519 ± 0.0350	0.8490 (ns↓)	0.8910 ± 0.0388	<0.0001 (***↓)	0.6170 ± 0.1690	<0.0001 (***↓)	0.6264 ± 0.1523	<0.0001 (***↓)	0.9607 ± 0.0081	0.0605 (ns↓)	0.5842 ± 0.1094	<0.0001 (***↓)
CLAM-MB	0.9607 ± 0.0148	0.8367 (ns↓)	0.8989 ± 0.0214	0.0402 (*↓)	0.6333 ± 0.0663	0.2482 (ns↓)	0.6126 ± 0.0760	0.3985 (ns↓)	0.9627 ± 0.0079	0.3682 (ns↓)	0.5939 ± 0.0229	0.3167 (ns↓)
DSMIL	0.9522 ± 0.0178	0.8086 (ns↓)	0.8734 ± 0.0406	<0.0001 (***↓)	0.5569 ± 0.0422	0.4899 (ns↓)	0.6385 ± 0.0937	0.0242 (*↓)	0.9562 ± 0.0083	0.0201 (*↓)	0.5766 ± 0.0707	0.1338 (ns↓)
TransMIL	0.9474 ± 0.0357	0.9132 (ns↓)	0.8974 ± 0.0474	<0.0001 (***↓)	0.6225 ± 0.0805	0.0640 (ns↓)	0.5645 ± 0.0455	0.0640 (ns↓)	0.9610 ± 0.0148	0.2536 (ns↓)	0.5730 ± 0.0377	0.0318 (*↓)
MambaMIL	0.9611 ± 0.0177	1.0000 (ns↓)	0.9046 ± 0.0364	<0.0001 (***↓)	0.6283 ± 0.0649	<0.0001 (***↓)	0.6094 ± 0.0059	<0.0001 (***↓)	0.9628 ± 0.0122	<0.0001 (***↓)	0.6074 ± 0.0247	<0.0001 (***↓)
MambaMIL + HiLA-MIL (Ours)	0.9661 ± 0.0151	N/A	0.9177 ± 0.0417	N/A	0.7175 ± 0.2184	N/A	0.6965 ± 0.1456	N/A	0.9689 ± 0.0104	N/A	0.6958 ± 0.1738	N/A
Methods	Breast invasive ductal carcinoma											
	AUC	*P*-value	Accuracy	*P*-value	Precision	*P*-value	Recall	*P*-value	Specificity	*P*-value	F1-Score	*P*-value
ABMIL	0.9748 ± 0.0130	0.9912 (ns↓)	0.8423 ± 0.0627	0.0156 (*↓)	0.3868 ± 0.0573	0.5983 (ns↓)	0.3611 ± 0.0434	0.5200 (ns↓)	0.9098 ± 0.0248	0.0571 (ns↓)	0.3674 ± 0.0421	0.5221 (ns↓)
CLAM-SB	0.9695 ± 0.0068	0.8838 (ns↓)	0.8527 ± 0.0577	0.0331 (*↓)	0.4425 ± 0.0482	0.2839 (ns↓)	0.3646 ± 0.0477	0.1889 (ns↓)	0.9130 ± 0.0281	0.2056 (ns↓)	0.3915 ± 0.0445	0.2818 (ns↓)
CLAM-MB	0.9619 ± 0.0125	0.0292 (*↓)	0.8119 ± 0.0686	<0.0001 (***↓)	0.3479 ± 0.0917	0.0444 (*↓)	0.2917 ± 0.0361	0.0077 (**↓)	0.9022 ± 0.0183	0.0119 (*↓)	0.3105 ± 0.0559	0.0447 (*↓)
DSMIL	0.9641 ± 0.0108	0.6996 (ns↓)	0.8318 ± 0.0720	<0.0001 (***↓)	0.4523 ± 0.0669	0.2848 (ns↓)	0.3264 ± 0.0470	0.0233 (*↓)	0.9264 ± 0.0119	0.5909 (ns↓)	0.3561 ± 0.0518	0.0309 (*↓)
TransMIL	0.9638 ± 0.0042	0.4244 (ns↓)	0.8432 ± 0.0429	0.0154 (*↓)	0.3345 ± 0.0839	0.0999 (ns↓)	0.3125 ± 0.0827	0.0486 (*↓)	0.9099 ± 0.0263	0.1112 (ns↓)	0.3165 ± 0.0818	0.0587 (ns↓)
MambaMIL	0.9679 ± 0.0080	0.9806 (ns↓)	0.8327 ± 0.0463	<0.0001 (***↓)	0.3356 ± 0.0828	0.0242 (*↓)	0.2986 ± 0.0592	0.0242 (*↓)	0.9261 ± 0.0190	0.2390 (ns↓)	0.3097 ± 0.0678	0.0242 (*↓)
MambaMIL + HiLA-MIL (Ours)	0.9780 ± 0.0049	N/A	0.8726 ± 0.0715	N/A	0.4729 ± 0.0599	N/A	0.4583 ± 0.1301	N/A	0.9368 ± 0.0260	N/A	0.4573 ± 0.0987	N/A
Methods	Breast invasive lobular carcinoma											
	AUC	*P*-value	Accuracy	*P*-value	Precision	*P*-value	Recall	*P*-value	Specificity	*P*-value	F1-Score	*P*-value
ABMIL	0.9388 ± 0.0523	0.5494 (ns↓)	0.7713 ± 0.1343	<0.0001 (***↓)	0.3095 ± 0.1688	<0.0001 (***↓)	0.2963 ± 0.1052	<0.0001 (***↓)	0.8248 ± 0.1156	<0.0001 (***↓)	0.2979 ± 0.1324	<0.0001 (***↓)
CLAM-SB	0.9719 ± 0.0353	0.6981 (ns↑)	0.8380 ± 0.1926	0.2489 (ns↓)	0.3095 ± 0.1688	0.2489 (ns↓)	0.3241 ± 0.1530	0.2489 (ns↓)	0.8414 ± 0.1430	0.4962 (ns↓)	0.3145 ± 0.1612	0.2489 (ns↓)
CLAM-MB	0.9311 ± 0.0403	0.2318 (ns↓)	0.6972 ± 0.0914	<0.0001 (***↓)	0.3095 ± 0.1688	<0.0001 (***↓)	0.2778 ± 0.1273	<0.0001 (***↓)	0.8062 ± 0.1417	<0.0001 (***↓)	0.2861 ± 0.1420	<0.0001 (***↓)
DSMIL	0.9723 ± 0.0257	0.8145 (ns↑)	0.7713 ± 0.1343	<0.0001 (***↓)	0.3095 ± 0.1688	<0.0001 (***↓)	0.2963 ± 0.1052	<0.0001 (***↓)	0.8248 ± 0.1156	<0.0001 (***↓)	0.2979 ± 0.1324	<0.0001 (***↓)
TransMIL	0.9610 ± 0.0346	0.6470 (ns↓)	0.7713 ± 0.1343	<0.0001 (***↓)	0.3095 ± 0.1688	0.2489 (ns↓)	0.2963 ± 0.1052	<0.0001 (***↓)	0.8248 ± 0.1156	0.2489 (ns↓)	0.2979 ± 0.1324	0.2489 (ns↓)
MambaMIL	0.9670 ± 0.0244	0.9348 (ns↓)	0.8546 ± 0.0478	1.0000 (ns↓)	0.4028 ± 0.1339	<0.0001 (***↓)	0.3519 ± 0.1123	1.0000 (ns↓)	0.8630 ± 0.1230	1.0000 (ns↓)	0.3709 ± 0.1179	<0.0001 (***↓)
MambaMIL + HiLA-MIL (Ours)	0.9683 ± 0.0183	N/A	0.8546 ± 0.0478	N/A	0.4028 ± 0.1339	N/A	0.3519 ± 0.1123	N/A	0.8630 ± 0.1230	N/A	0.3709 ± 0.1179	N/A
YDSY												
Methods	Multi-cancer											
	AUC	*P*-value	Accuracy	*P*-value	Precision	*P*-value	Recall	*P*-value	Specificity	P-value	F1-Score	*P*-value
ABMIL	0.9024 ± 0.0472	0.3984 (ns↓)	0.7902 ± 0.0388	0.0684 (ns↓)	0.5692 ± 0.0725	0.0478 (*↓)	0.5326 ± 0.0566	0.2696 (ns↓)	0.9244 ± 0.0221	0.3106 (ns↑)	0.5401 ± 0.0543	0.1439 (ns↓)
CLAM-SB	0.9113 ± 0.0476	0.3925 (ns↓)	0.7684 ± 0.0643	0.0248 (*↓)	0.5450 ± 0.0574	0.0274 (*↓)	0.5494 ± 0.0679	0.0916 (ns↓)	0.9037 ± 0.0279	0.1125 (ns↓)	0.5391 ± 0.0575	0.0586 (ns↓)
CLAM-MB	0.9190 ± 0.0320	0.8656 (ns↑)	0.7726 ± 0.0447	0.0338 (*↓)	0.5284 ± 0.0574	0.2548 (ns↓)	0.5085 ± 0.0514	0.0043 (**↓)	0.8953 ± 0.0444	0.0122 (*↓)	0.5058 ± 0.0600	0.0187 (*↓)
DSMIL	0.9154 ± 0.0484	0.4837 (ns↓)	0.7671 ± 0.0639	<0.0001 (***↓)	0.5523 ± 0.0774	0.0039 (**↓)	0.5304 ± 0.0694	0.0101 (*↓)	0.9127 ± 0.0349	0.0083 (**↓)	0.5272 ± 0.0642	0.0052 (**↓)
TransMIL	0.9072 ± 0.0393	0.1768 (ns↓)	0.7157 ± 0.0260	<0.0001 (***↓)	0.5142 ± 0.0845	0.0040 (**↓)	0.4602 ± 0.0558	<0.0001 (***↓)	0.8676 ± 0.0367	<0.0001 (***↓)	0.4641 ± 0.0597	0.0005 (***↓)
MambaMIL	0.9174 ± 0.0413	0.6646 (ns↓)	0.7916 ± 0.0700	0.0579 (ns↓)	0.5594 ± 0.0808	0.3241 (ns↓)	0.5327 ± 0.0645	0.1002 (ns↓)	0.9086 ± 0.0336	0.0647 (ns↓)	0.5378 ± 0.0671	0.2092 (ns↓)
MambaMIL + HiLA-MIL (Ours)	0.9184 ± 0.0278	N/A	0.7917 ± 0.0624	N/A	0.5861 ± 0.0813	N/A	0.5811 ± 0.0939	N/A	0.9242 ± 0.0398	N/A	0.5719 ± 0.0803	N/A
	Gastric adenocarcinoma											
	AUC	*P*-value	Accuracy	*P*-value	Precision	*P*-value	Recall	*P*-value	Specificity	*P*-value	F1-Score	*P*-value
ABMIL	0.7589 ± 0.2207	0.7922 (ns↑)	0.6500 ± 0.2179	<0.0001 (***↑)	0.3750 ± 0.2165	<0.0001 (***↑)	0.3194 ± 0.1339	<0.0001 (***↑)	0.8625 ± 0.1409	<0.0001 (***↑)	0.3365 ± 0.1685	<0.0001 (***↑)
CLAM-SB	0.7469 ± 0.2449	0.2510 (ns↓)	0.4333 ± 0.4041	<0.0001 (***↓)	0.2222 ± 0.2097	<0.0001 (***↓)	0.2222 ± 0.2097	<0.0001 (***↓)	0.7639 ± 0.2295	<0.0001 (***↓)	0.2167 ± 0.2021	<0.0001 (***↓)
CLAM-MB	0.7603 ± 0.1736	0.9685 (ns↑)	0.4333 ± 0.0577	0.2480 (ns↓)	0.1667 ± 0.0722	0.4281 (ns↓)	0.1944 ± 0.0481	0.2480 (ns↓)	0.7472 ± 0.1119	0.4281 (ns↓)	0.1786 ± 0.0619	0.3069 (ns↓)
DSMIL	0.7806 ± 0.2038	0.7869 (ns↑)	0.5833 ± 0.3329	<0.0001 (***↑)	0.3611 ± 0.2406	1.0000 (ns↑)	0.2917 ± 0.1816	<0.0001 (***↑)	0.8236 ± 0.2082	<0.0001 (***↑)	0.3167 ± 0.2028	<0.0001 (***↑)
TransMIL	0.7975 ± 0.2341	0.9407 (ns↑)	0.4500 ± 0.2784	0.2525 (ns↓)	0.2083 ± 0.1816	0.3744 (ns↓)	0.2083 ± 0.1502	0.2525 (ns↓)	0.7194 ± 0.1370	0.1872 (ns↓)	0.1976 ± 0.1494	0.2814 (ns↓)
MambaMIL	0.7672 ± 0.2513	0.5310 (ns↑)	0.5167 ± 0.4481	1.0000 (ns↑)	0.3056 ± 0.2679	0.7489 (ns↑)	0.2639 ± 0.2295	0.5007 (ns↑)	0.7958 ± 0.2276	0.6284 (ns↓)	0.2722 ± 0.2359	0.7489 (ns↑)
MambaMIL + HiLA-MIL (Ours)	0.7553 ± 0.0998	N/A	0.5000 ± 0.3000	N/A	0.2778 ± 0.2097	N/A	0.2500 ± 0.1667	N/A	0.8028 ± 0.1937	N/A	0.2611 ± 0.1836	N/A
Methods	Colorectal adenocarcinoma											
	AUC	*P*-value	Accuracy	*P*-value	Precision	*P*-value	Recall	P-value	Specificity	*P*-value	F1-Score	*P*-value
ABMIL	0.9635 ± 0.0269	0.8468 (ns↓)	0.8875 ± 0.0437	0.0715 (ns↓)	0.6559 ± 0.0341	0.9373 (ns↓)	0.6399 ± 0.0791	0.5799 (ns↓)	0.9625 ± 0.0108	0.9599 (ns↓)	0.6434 ± 0.0519	0.8308 (ns↓)
CLAM-SB	0.9708 ± 0.0391	0.9581 (ns↓)	0.8524 ± 0.0974	<0.0001 (***↓)	0.6228 ± 0.0410	0.4109 (ns↓)	0.5962 ± 0.0447	0.0772 (ns↓)	0.9382 ± 0.0265	0.0854 (ns↓)	0.6068 ± 0.0434	0.1484 (ns↓)
CLAM-MB	0.9682 ± 0.0291	0.6252 (ns↓)	0.8905 ± 0.0728	0.0317 (*↓)	0.6777 ± 0.0827	0.7762 (ns ↑ )	0.6028 ± 0.0560	0.1007 (ns↓)	0.9425 ± 0.0282	0.2235 (ns↓)	0.6145 ± 0.0673	0.2235 (ns↓)
DSMIL	0.9670 ± 0.0396	0.7843 (ns↓)	0.8543 ± 0.1098	0.0007 (***↓)	0.6267 ± 0.0696	0.4174 (ns↓)	0.6164 ± 0.1020	0.2013 (ns↓)	0.9430 ± 0.0410	0.1998 (ns↓)	0.6168 ± 0.0821	0.2412 (ns↓)
TransMIL	0.9574 ± 0.0453	0.9253 (ns↓)	0.8428 ± 0.1104	<0.0001 (***↓)	0.6282 ± 0.0788	0.3352 (ns↓)	0.5616 ± 0.0958	0.0067 (**↓)	0.9329 ± 0.0461	0.0608 (ns↓)	0.5785 ± 0.0915	0.0567 (ns↓)
MambaMIL	0.9644 ± 0.0298	0.5349 (ns↓)	0.8541 ± 0.0574	<0.0001 (***↓)	0.5956 ± 0.0521	0.1735 (ns↓)	0.5726 ± 0.0619	0.0516 (ns↓)	0.9311 ± 0.0298	0.0516 (ns↓)	0.5788 ± 0.0580	0.0711 (ns↓)
MambaMIL + HiLA-MIL (Ours)	0.9844 ± 0.0226	N/A	0.9134 ± 0.0855	N/A	0.6721 ± 0.0800	N/A	0.6706 ± 0.0643	N/A	0.9659 ± 0.0296	N/A	0.6666 ± 0.0653	N/A
Methods	Lung adenocarcinoma											
	AUC	*P*-value	Accuracy	P-value	Precision	*P*-value	Recall	*P*-value	Specificity	*P*-value	F1-Score	*P*-value
ABMIL	0.8387 ± 0.0797	0.8877 (ns↓)	0.6435 ± 0.0637	0.2559 (ns↓)	0.5409 ± 0.0819	0.1273 (ns↑ )	0.4750 ± 0.0661	0.5097 (ns↓)	0.8736 ± 0.0187	0.8716 (ns↓)	0.4963 ± 0.0570	0.3843 (ns↑)
CLAM-SB	0.8864 ± 0.0888	0.9365 (ns↑)	0.7263 ± 0.0961	0.0712 (ns ↑ )	0.5284 ± 0.0529	0.3874 (ns↓)	0.5292 ± 0.0481	0.5643 (ns↑)	0.8968 ± 0.0244	0.4695 (ns↑)	0.5250 ± 0.0531	0.8585 (ns↑)
CLAM-MB	0.8971 ± 0.0768	0.8528 (ns↑)	0.7757 ± 0.0824	<0.0001 (***↑)	0.5774 ± 0.1305	0.7667 (ns↑)	0.5278 ± 0.0481	0.5319 (ns↑)	0.9036 ± 0.0338	0.1876 (ns↑)	0.5272 ± 0.0634	0.5166 (ns↑)
DSMIL	0.8795 ± 0.1073	0.9828 (ns↑)	0.6663 ± 0.1935	0.4968 (ns↓)	0.5194 ± 0.1766	0.2201 (ns↓)	0.4667 ± 0.1402	0.5125 (ns↓)	0.8796 ± 0.0502	0.7491 (ns↓)	0.4840 ± 0.1459	0.3724 (ns↓)
TransMIL	0.8497 ± 0.1368	0.5891 (ns↓)	0.6388 ± 0.2522	0.1490 (ns↓)	0.5863 ± 0.1337	0.7661 (ns↑)	0.4708 ± 0.1393	0.5988 (ns↓)	0.8719 ± 0.0660	0.4316 (ns↓)	0.4819 ± 0.1428	0.7759 (ns↓)
MambaMIL	0.9033 ± 0.0771	0.5032 (ns↑)	0.7652 ± 0.1077	<0.0001 (***↑)	0.6081 ± 0.1133	0.5676 (ns↑)	0.5361 ± 0.0774	0.4497 (ns↑)	0.9093 ± 0.0244	0.2537 (ns↑)	0.5550 ± 0.0826	0.4497 (ns↑)
MambaMIL + HiLA-MIL (Ours)	0.8788 ± 0.0496	N/A	0.6673 ± 0.0247	N/A	0.5298 ± 0.0901	N/A	0.4889 ± 0.0428	N/A	0.8868 ± 0.0122	N/A	0.4914 ± 0.0471	N/A
Methods	Breast invasive ductal carcinoma											
	AUC	*P*-value	Accuracy	*P*-value	Precision	P-value	Recall	*P*-value	Specificity	*P*-value	F1-Score	*P*-value
ABMIL	0.9704 ± 0.0229	0.9519 (ns↓)	0.8593 ± 0.0733	0.3672 (ns↓)	0.7323 ± 0.0981	0.4339 (ns↓)	0.6868 ± 0.1050	0.2453 (ns↓)	0.9478 ± 0.0315	0.2790 (ns↓)	0.6968 ± 0.1132	0.4034 (ns↓)
CLAM-SB	0.9572 ± 0.0076	0.3639 (ns↓)	0.8778 ± 0.0434	1.0000 (ns↓)	0.7823 ± 0.0845	0.9417 (ns↓)	0.8160 ± 0.1086	0.4978 (ns↓)	0.9615 ± 0.0127	0.4953 (ns↓)	0.7713 ± 0.0729	0.5593 (ns↓)
CLAM-MB	0.9765 ± 0.0190	0.9357 (ns↑)	0.8111 ± 0.0823	<0.0001 (***↓)	0.6509 ± 0.0448	0.0183 (*↓)	0.6344 ± 0.0829	0.0141 (*↓)	0.9248 ± 0.0414	0.0079 (**↓)	0.6327 ± 0.0767	0.0595 (ns↓)
DSMIL	0.9544 ± 0.0160	0.1291 (ns↓)	0.7793 ± 0.0200	<0.0001 (***↓)	0.7127 ± 0.0222	0.0141 (*↓)	0.6939 ± 0.1246	0.0035 (**↓)	0.9310 ± 0.0177	0.0065 (**↓)	0.6628 ± 0.0379	0.0196 (*↓)
TransMIL	0.9424 ± 0.0214	0.1019 (ns↓)	0.7422 ± 0.1545	<0.0001 (***↓)	0.6719 ± 0.0563	0.0089 (**↓)	0.5600 ± 0.1162	0.0015 (**↓)	0.8968 ± 0.0692	0.0010 (**↓)	0.5764 ± 0.1135	0.0067 (**↓)
MambaMIL	0.9659 ± 0.0275	0.8856 (ns↓)	0.8696 ± 0.0623	0.3718 (ns↓)	0.7183 ± 0.0408	0.4341 (ns↓)	0.7075 ± 0.0182	0.2465 (ns↓)	0.9485 ± 0.0266	0.1989 (ns↓)	0.7072 ± 0.0111	0.4310 (ns↓)
MambaMIL + HiLA-MIL (Ours)	0.9734 ± 0.0073	N/A	0.8778 ± 0.0434	N/A	0.7840 ± 0.0656	N/A	0.8292 ± 0.1239	N/A	0.9654 ± 0.0118	N/A	0.7737 ± 0.0619	N/A
Methods	Breast invasive lobular carcinoma											
	AUC	*P*-value	Accuracy	*P*-value	Precision	*P*-value	Recall	*P*-value	Specificity	*P*-value	F1-Score	*P*-value
ABMIL	0.9805 ± 0.0170	1.0000 (ns↓)	0.9107 ± 0.0778	<0.0001 (***↓)	0.5417 ± 0.1909	0.5025 (ns↓)	0.5417 ± 0.0722	<0.0001 (***↓)	0.9757 ± 0.0217	<0.0001 (***↓)	0.5278 ± 0.1273	<0.0001 (***↓)
CLAM-SB	0.9955 ± 0.0079	0.4949 (ns↓)	0.9524 ± 0.0825	<0.0001 (***↓)	0.5694 ± 0.1577	1.0000 (ns↓)	0.5833 ± 0.1443	<0.0001 (***↓)	0.9583 ± 0.0722	<0.0001 (***↓)	0.5758 ± 0.1513	<0.0001 (***↓)
CLAM-MB	0.9932 ± 0.0118	0.8737 (ns↓)	0.9524 ± 0.0825	<0.0001 (***↓)	0.5694 ± 0.1577	1.0000 (ns↓)	0.5833 ± 0.1443	<0.0001 (***↓)	0.9583 ± 0.0722	<0.0001 (***↓)	0.5758 ± 0.1513	<0.0001 (***↓)
DSMIL	0.9955 ± 0.0079	0.8368 (ns↓)	0.9524 ± 0.0825	<0.0001 (***↓)	0.5417 ± 0.1909	1.0000 (ns↓)	0.5833 ± 0.1443	<0.0001 (***↓)	0.9861 ± 0.0241	<0.0001 (***↓)	0.5556 ± 0.1735	<0.0001 (***↓)
TransMIL	0.9892 ± 0.0144	<0.0001 (***↓)	0.9048 ± 0.1650	<0.0001 (***↓)	0.4762 ± 0.2865	0.5032 (ns↓)	0.5000 ± 0.2500	<0.0001 (***↓)	0.9167 ± 0.1443	<0.0001 (***↓)	0.4861 ± 0.2711	<0.0001 (***↓)
MambaMIL	0.9864 ± 0.0236	0.9667 (ns↓)	0.9524 ± 0.0825	<0.0001 (***↓)	0.5694 ± 0.1577	1.0000 (ns↓)	0.5833 ± 0.1443	<0.0001 (***↓)	0.9583 ± 0.0722	<0.0001 (***↓)	0.5758 ± 0.1513	<0.0001 (***↓)
MambaMIL+HiLA-MIL (Ours)	1.0000 ± 0.0000	N/A	1.0000 ± 0.0000	N/A	0.6667 ± 0.1443	N/A	0.6667 ± 0.1443	N/A	1.0000 ± 0.0000	N/A	0.6667 ± 0.1443	N/A

*: P < 0.05；**: P < 0.01；***: P < 0.001；ns: no statistical significance. ↑: an increase in metrics of the baseline models compared with the MambaMIL + HiLA-MIL model. ↓: a decrease in metrics of the baseline models compared with the MambaMIL + HiLA-MIL model. *N/A* Not applicable. Statistical comparison was not performed. A two-sided paired permutation test with 10,000 permutations was applied.

In the overall four-class classification task of LNM on the NIMM dataset, the MambaMIL + HiLA-MIL model exhibited prominent overall performance advantages in both multi-cancer and single-cancer cohorts. In the multi-cancer and breast invasive ductal carcinoma cohorts, the model achieved highly consistent superior performance with optimal values across all evaluation metrics. It yielded statistically significant improvements in accuracy, precision, recall, specificity, and F1-score compared with all six baseline models. Meanwhile, the multi-cancer cohort presented significantly higher AUC values than CLAM-SB and MambaMIL, and the breast invasive ductal carcinoma cohort showed statistically significant AUC improvements over CLAM-SB, CLAM-MB, and TransMIL. In the breast invasive lobular carcinoma cohort, the model obtained very significant elevations in accuracy and recall against all six baseline models. In the lung adenocarcinoma cohort, our model achieved statistically significant improvements in accuracy compared with all six baseline models; meanwhile, significant increases in recall, specificity, and F1-score were observed relative to most baseline models. In the gastric adenocarcinoma and colorectal adenocarcinoma cohorts, the model achieved statistically significant improvements in accuracy over multiple baseline models.

**Fig. 2:**
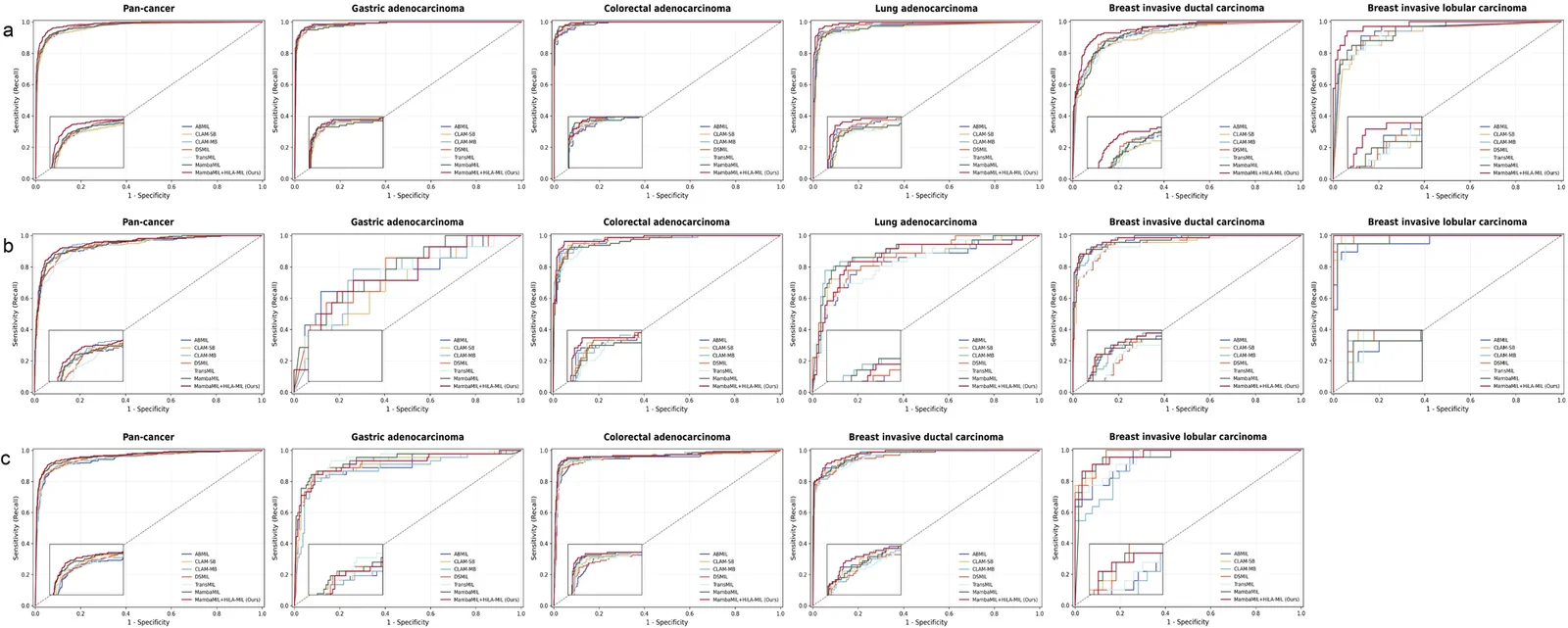
Multi‑cancer classification ROC analysis with the GigaPath feature extractor across three independent datasets. ROC plots of the six baseline models as well as the MambaMIL + HiLA-MIL model for multi-cancer, gastric adenocarcinoma, colorectal adenocarcinoma, lung adenocarcinoma, breast invasive ductal carcinoma, and breast invasive lobular carcinoma across **a** the NIMM, **b** YDSY, and **c** DLZX datasets. Source data are provided as a Source Data file.

The ROC results of the MambaMIL + HiLA-MIL model and six baseline models based on the GigaPath feature extractor are presented in Fig. 2a for the multi-cancer and five individual cancer types on the NIMM dataset. On the NIMM dataset, although the AUC differences among models did not reach statistical significance in most cancer subtypes, the ROC plots further demonstrated that the MambaMIL + HiLA-MIL model achieved superior overall discriminative performance compared with all baseline models in the multi-cancer, colorectal adenocarcinoma, lung adenocarcinoma, breast invasive ductal carcinoma, and breast invasive lobular carcinoma cohorts. No obvious differences in discriminative ability were observed among different models in the gastric adenocarcinoma cohort. Overall, the MambaMIL + HiLA-MIL model realized consistent multi-dimensional performance gains over baseline models in both multi-cancer and single-cancer cohorts of the NIMM dataset. It delivered stable cross-cancer superiority, especially in accuracy, which fully verified the effectiveness and cross-cancer robustness of our model for overall four-class classification of LNM.

To further verify the generalization ability of the model, we conducted experiments on two external datasets, DLZX and YDSY. Consistent with the results of the internal NIMM dataset, the model achieved excellent overall performance, while certain performance differences were observed across different cancer cohorts. The detailed results are presented in Table 2. Our model exhibited prominent superiority in the multi-cancer, breast invasive ductal carcinoma, breast invasive lobular carcinoma, and colorectal adenocarcinoma cohorts across the two external datasets. It achieved statistically significant improvements in accuracy, recall, specificity, and F1-score, among which the advantage in accuracy is the most stable and prominent. In the gastric adenocarcinoma cohort of the YDSY dataset, most evaluation metrics were significantly improved, while AUC remained at a moderate level. However, in the DLZX gastric adenocarcinoma cohort, the overall performance of the model was limited. For the lung adenocarcinoma cohort, only accuracy showed a statistically significant difference, and other metrics remained at high levels without reaching statistical significance.

The ROC results (Fig. 2b, c) of the external DLZX and YDSY datasets exhibited good consistency with the internal NIMM dataset. The MambaMIL + HiLA-MIL model delivered better overall discriminative performance than all baseline models in the multi-cancer, colorectal adenocarcinoma, breast invasive ductal carcinoma, and breast invasive lobular carcinoma cohorts. In the gastric adenocarcinoma cohort, the curves of each model overlapped obviously, and no significant performance differences were detected among groups. Slightly differently, in the YDSY dataset, the model presented suboptimal overall discriminative performance for lung adenocarcinoma, with a markedly limited performance advantage compared with other cohorts. Overall, external validation results indicated that the MambaMIL + HiLA-MIL model possesses excellent cross-cancer generalization focused on stable accuracy.

In addition to GigaPath, this study further incorporated the ResNet, UNI, and Virchow feature extractors for supplementary comparative analysis. It aims to comprehensively investigate the performance of our model in identifying LNM across multi-cancer and individual cancer types under different feature extraction backbones. The detailed results are presented in Supplementary Tables 3–5. In this study, the term “excellent and stable performance” is uniformly defined as that the proposed model outperforms six baseline models in nearly all evaluation metrics, with statistically significant improvements in multiple core indicators compared with various baseline methods. In the internal NIMM dataset, the proposed model exhibited distinct performance differentiation across different feature extractors. With the ResNet extractor, the model achieved favorable and stable performance in the classification of multi-cancer, colorectal adenocarcinoma, lung adenocarcinoma, breast invasive ductal carcinoma and breast invasive lobular carcinoma. With the UNI extractor, the model maintained stable and robust diagnostic performance in multi-cancer, gastric adenocarcinoma, colorectal adenocarcinoma and breast invasive ductal carcinoma. With the Virchow extractor, the model delivered superior comprehensive performance in multi-cancer, gastric adenocarcinoma and breast invasive ductal carcinoma. Considering the comprehensive performance of ResNet, UNI, and Virchow, ROC analysis on the NIMM dataset further confirmed our model’s strong discriminative ability in multi-cancer and invasive ductal breast carcinoma, outperforming most baselines (Supplementary Fig. 3). Synthesizing the comparative results of the three feature extractors, the proposed model presented the optimal generalization stability in multi-cancer and breast invasive ductal carcinoma of within the NIMM dataset.

To further validate the external generalizability and robustness of the model in evaluating LNM tasks across multi-cancer and individual cancer types, cross-cohort validation was conducted on two independent external datasets. Under the ResNet extractor, the model had moderate overall performance, performing well only in colorectal adenocarcinoma. Under the UNI extractor, the model realized excellent and stable performance in multi-cancer and colorectal adenocarcinoma in the DLZX dataset, while it showed competitive advantages in multi-cancer, lung adenocarcinoma and breast invasive lobular carcinoma in the YDSY dataset. Under the Virchow extractor, the model maintained steady performance in multi-cancer and colorectal adenocarcinoma in the DLZX dataset and achieved prominent comprehensive performance in multi-cancer, gastric adenocarcinoma, breast invasive ductal carcinoma and breast invasive lobular carcinoma in the YDSY dataset. On the two external datasets DLZX and YDSY, ROC results showed our model achieved high AUC and stable discriminative efficacy in multi-cancer and colorectal adenocarcinoma; despite no statistically significant AUC differences from baselines, it remained highly competitive with optimal models (Supplementary Figs. 4 and 5). Collectively, the proposed algorithm possessed the most stable performance in nulti-cancer and colorectal cancer in the DLZX dataset, and demonstrated the strongest generalization in multi-cancer and breast invasive lobular carcinoma in the YDSY dataset.

In conclusion, the comprehensive results from multiple feature extractors and multicenter datasets confirmed that the MambaMIL + HiLA-MIL model achieved excellent and stable overall performance in LNM detection tasks across multi-cancer and individual cancer types. Although model performance was modulated by feature extraction architecture, data distribution and cancer type characteristics, its distinctive strengths remained prominent.

### Comparison with feature extractor models

On the internal NIMM dataset, the proposed MambaMIL + HiLA-MIL architecture adopted four distinct feature extractors. The comparative results of all performance metrics for overall four-class classification and individual LNM category are presented in Table 3.

**Table 3:** Performance comparison of feature extractors on NIMM, DLZX, and YDSY Datasets

NIMM																			
Baseline Model	Compare Model	Overall																	
		AUC			Accuracy			Precision			Recall			Specificity			F1-Score		
		Base	Comp	*P*-value	Base	Comp	*P*-value	Base	Comp	*P*-value	Base	Comp	*P*-value	Base	Comp	*P*-value	Base	Comp	*P*-value
ResNet	UNI	0.9700±0.0140	0.9710±0.0105	0.4429 (ns ↑ )	0.9019±0.0318	0.9290±0.0256	<0.0001 (***↑)	0.8193±0.0661	0.8722±0.0646	0.1775 (ns ↑ )	0.8029±0.0518	0.8576±0.0501	0.0602 (ns ↑ )	0.9657±0.0111	0.9772±0.0072	0.0029 (**↑)	0.7993±0.0547	0.8455±0.0470	0.0935 (ns ↑ )
ResNet	Virchow	0.9700±0.0140	0.9695±0.0131	0.5221 (ns↓)	0.9019±0.0318	0.9125±0.0337	0.0857 (ns ↑ )	0.8193±0.0661	0.8149±0.0763	0.9940 (ns↓)	0.8029±0.0518	0.8076±0.0792	0.9046 (ns ↑ )	0.9657±0.0111	0.9714±0.0122	0.2015 (ns ↑ )	0.7993±0.0547	0.7995±0.0704	0.9453 (ns ↑ )
ResNet	GigaPath	0.9700±0.0140	0.9759±0.0114	0.1618 (ns ↑ )	0.9019±0.0318	0.9291±0.0307	<0.0001 (***↑)	0.8193±0.0661	0.8522±0.0599	0.1492 (ns ↑ )	0.8029±0.0518	0.8678±0.0560	0.0228 (*↑)	0.9657±0.0111	0.9774±0.0095	0.0013 (**↑)	0.7993±0.0547	0.8468±0.0610	0.1009 (ns ↑ )
UNI	Virchow	0.9710±0.0105	0.9695±0.0131	0.8268 (ns↓)	0.9290±0.0256	0.9125±0.0337	0.0014 (**↓)	0.8722±0.0646	0.8149±0.0763	0.1074 (ns↓)	0.8576±0.0501	0.8076±0.0792	0.0424 (*↓)	0.9772±0.0072	0.9714±0.0122	0.1089 (ns↓)	0.8455±0.0470	0.7995±0.0704	0.0645 (ns↓)
UNI	GigaPath	0.9710±0.0105	0.9759±0.0114	0.7240 (ns ↑ )	0.9290±0.0256	0.9291±0.0307	1.0000 (ns ↑ )	0.8722±0.0646	0.8522±0.0599	0.9650 (ns↓)	0.8576±0.0501	0.8678±0.0560	0.6553 (ns ↑ )	0.9772±0.0072	0.9774±0.0095	0.9470 (ns ↑ )	0.8455±0.0470	0.8468±0.0610	0.9405 (ns ↑ )
Virchow	GigaPath	0.9695±0.0131	0.9759±0.0114	0.4666 (ns ↑ )	0.9125±0.0337	0.9291±0.0307	0.0031 (**↑)	0.8149±0.0763	0.8522±0.0599	0.1416 (ns ↑ )	0.8076±0.0792	0.8678±0.0560	0.0379 (*↑)	0.9714±0.0122	0.9774±0.0095	0.1165 (ns ↑ )	0.7995±0.0704	0.8468±0.0610	0.1138 (ns ↑ )
Baseline Model	Compare Model	Negative																	
		AUC			Accuracy			Precision			Recall			Specificity			F1-Score		
		Base	Comp	*P*-value	Base	Comp	*P*-value	Base	Comp	*P*-value	Base	Comp	*P*-value	Base	Comp	*P*-value	Base	Comp	*P*-value
ResNet	UNI	0.9960±0.0125	1.0000±0.0000	<0.0001 (***↑)	0.9970±0.0063	1.0000±0.0000	0.4955 (ns ↑ )	0.9958±0.0132	1.0000±0.0000	0.4955 (ns ↑ )	0.9957±0.0137	1.0000±0.0000	1.0000 (ns ↑ )	0.9977±0.0072	1.0000±0.0000	1.0000 (ns ↑ )	0.9957±0.0092	1.0000±0.0000	0.4955 (ns ↑ )
ResNet	Virchow	0.9960±0.0125	0.9978±0.0069	0.5046 (ns ↑ )	0.9970±0.0063	0.9924±0.0192	0.4452 (ns↓)	0.9958±0.0132	0.9852±0.0468	0.1388 (ns↓)	0.9957±0.0137	0.9957±0.0137	1.0000 (ns↓)	0.9977±0.0072	0.9907±0.0294	0.0638 (ns↓)	0.9957±0.0092	0.9898±0.0255	0.2780 (ns↓)
ResNet	GigaPath	0.9960±0.0125	1.0000±0.0000	<0.0001 (***↑)	0.9970±0.0063	0.9985±0.0048	1.0000 (ns ↑ )	0.9958±0.0132	1.0000±0.0000	0.7486 (ns ↑ )	0.9957±0.0137	0.9957±0.0137	1.0000 (ns↓)	0.9977±0.0072	1.0000±0.0000	1.0000 (ns ↑ )	0.9957±0.0092	0.9978±0.0070	1.0000 (ns ↑ )
UNI	Virchow	1.0000±0.0000	0.9978±0.0069	0.9174 (ns↓)	1.0000±0.0000	0.9924±0.0192	0.0635 (ns↓)	1.0000±0.0000	0.9852±0.0468	<0.0001 (***↓)	1.0000±0.0000	0.9957±0.0137	1.0000 (ns↓)	1.0000±0.0000	0.9907±0.0294	0.1280 (ns↓)	1.0000±0.0000	0.9898±0.0255	0.0635 (ns↓)
UNI	GigaPath	1.0000±0.0000	1.0000±0.0000	1.0000 (ns↓)	1.0000±0.0000	0.9985±0.0048	1.0000 (ns↓)	1.0000±0.0000	1.0000±0.0000	1.0000 (ns↓)	1.0000±0.0000	0.9957±0.0137	1.0000 (ns↓)	1.0000±0.0000	1.0000±0.0000	1.0000 (ns↓)	1.0000±0.0000	0.9978±0.0070	1.0000 (ns↓)
Virchow	GigaPath	0.9978±0.0069	1.0000±0.0000	0.7228 (ns ↑ )	0.9924±0.0192	0.9985±0.0048	0.2199 (ns ↑ )	0.9852±0.0468	1.0000±0.0000	0.0320 (*↑)	0.9957±0.0137	0.9957±0.0137	1.0000 (ns↓)	0.9907±0.0294	1.0000±0.0000	0.1280 (ns ↑ )	0.9898±0.0255	0.9978±0.0070	0.1557 (ns ↑ )
Baseline Model	Compare Model	ITC																	
		AUC			Accuracy			Precision			Recall			Specificity			F1-Score		
		Base	Comp	*P*-value	Base	Comp	*P*-value	Base	Comp	*P*-value	Base	Comp	*P*-value	Base	Comp	*P*-value	Base	Comp	*P*-value
ResNet	UNI	0.9643±0.0240	0.9630±0.0199	0.7732 (ns↓)	0.9562±0.0181	0.9623±0.0178	0.4026 (ns ↑ )	0.6462±0.1640	0.7395±0.2062	0.5764 (ns ↑ )	0.7000±0.1581	0.7750±0.2486	0.2722 (ns ↑ )	0.9727±0.0171	0.9743±0.0241	1.0000 (ns ↑ )	0.6607±0.1278	0.7072±0.1106	0.4627 (ns ↑ )
ResNet	Virchow	0.9643±0.0240	0.9510±0.0207	0.3985 (ns↓)	0.9562±0.0181	0.9487±0.0190	0.5133 (ns↓)	0.6462±0.1640	0.5783±0.1796	0.3975 (ns↓)	0.7000±0.1581	0.6500±0.2934	0.4271 (ns↓)	0.9727±0.0171	0.9679±0.0212	0.4032 (ns↓)	0.6607±0.1278	0.5857±0.1873	0.4229 (ns↓)
ResNet	GigaPath	0.9643±0.0240	0.9707±0.0149	0.6084 (ns ↑ )	0.9562±0.0181	0.9578±0.0220	0.7506 (ns ↑ )	0.6462±0.1640	0.6289±0.1413	0.7725 (ns↓)	0.7000±0.1581	0.8750±0.1318	0.0203 (*↑)	0.9727±0.0171	0.9631±0.0213	0.3314 (ns↓)	0.6607±0.1278	0.7226±0.1177	0.4325 (ns ↑ )
UNI	Virchow	0.9630±0.0199	0.9510±0.0207	0.1045 (ns↓)	0.9623±0.0178	0.9487±0.0190	0.0422 (*↓)	0.7395±0.2062	0.5783±0.1796	0.1019 (ns↓)	0.7750±0.2486	0.6500±0.2934	0.0402 (*↓)	0.9743±0.0241	0.9679±0.0212	0.2111 (ns↓)	0.7072±0.1106	0.5857±0.1873	0.0427 (*↓)
UNI	GigaPath	0.9630±0.0199	0.9707±0.0149	0.8002 (ns ↑ )	0.9623±0.0178	0.9578±0.0220	0.6889 (ns↓)	0.7395±0.2062	0.6289±0.1413	0.2725 (ns↓)	0.7750±0.2486	0.8750±0.1318	0.1114 (ns ↑ )	0.9743±0.0241	0.9631±0.0213	0.1190 (ns↓)	0.7072±0.1106	0.7226±0.1177	0.9998 (ns ↑ )
Virchow	GigaPath	0.9510±0.0207	0.9707±0.0149	0.1588 (ns ↑ )	0.9487±0.0190	0.9578±0.0220	0.2421 (ns ↑ )	0.5783±0.1796	0.6289±0.1413	0.5371 (ns ↑ )	0.6500±0.2934	0.8750±0.1318	0.0071 (**↑)	0.9679±0.0212	0.9631±0.0213	0.6602 (ns↓)	0.5857±0.1873	0.7226±0.1177	0.0768 (ns ↑ )
Baseline Model	Compare Model	Micro																	
		AUC			Accuracy			Precision			Recall			Specificity			F1-Score		
		Base	Comp	*P*-value	Base	Comp	*P*-value	Base	Comp	*P*-value	Base	Comp	*P*-value	Base	Comp	*P*-value	Base	Comp	*P*-value
ResNet	UNI	0.9398±0.0260	0.9355±0.0312	0.6975 (ns↓)	0.9124±0.0300	0.9320±0.0216	0.0719 (ns ↑ )	0.7102±0.1895	0.7839±0.1702	0.2265 (ns ↑ )	0.5708±0.2013	0.6972±0.1722	0.0511 (ns ↑ )	0.9603±0.0282	0.9655±0.0304	0.4610 (ns ↑ )	0.6065±0.1402	0.7140±0.0878	0.0714 (ns ↑ )
ResNet	Virchow	0.9398±0.0260	0.9439±0.0323	0.4368 (ns ↑ )	0.9124±0.0300	0.9215±0.0298	0.4028 (ns ↑ )	0.7102±0.1895	0.7367±0.1773	0.5327 (ns ↑ )	0.5708±0.2013	0.6236±0.1394	0.4179 (ns ↑ )	0.9603±0.0282	0.9638±0.0275	0.8592 (ns ↑ )	0.6065±0.1402	0.6628±0.1162	0.4543 (ns ↑ )
ResNet	GigaPath	0.9398±0.0260	0.9427±0.0302	0.5521 (ns ↑ )	0.9124±0.0300	0.9351±0.0254	0.0337 (*↑)	0.7102±0.1895	0.8151±0.1301	0.0297 (*↑)	0.5708±0.2013	0.6361±0.1566	0.2860 (ns ↑ )	0.9603±0.0282	0.9776±0.0164	0.0284 (*↑)	0.6065±0.1402	0.7024±0.1225	0.1216 (ns ↑ )
UNI	Virchow	0.9355±0.0312	0.9439±0.0323	0.7699 (ns ↑ )	0.9320±0.0216	0.9215±0.0298	0.3731 (ns↓)	0.7839±0.1702	0.7367±0.1773	0.5056 (ns↓)	0.6972±0.1722	0.6236±0.1394	0.1812 (ns↓)	0.9655±0.0304	0.9638±0.0275	0.6486 (ns↓)	0.7140±0.0878	0.6628±0.1162	0.2432 (ns↓)
UNI	GigaPath	0.9355±0.0312	0.9427±0.0302	0.9740 (ns ↑ )	0.9320±0.0216	0.9351±0.0254	0.8772 (ns ↑ )	0.7839±0.1702	0.8151±0.1301	0.2611 (ns ↑ )	0.6972±0.1722	0.6361±0.1566	0.1445 (ns↓)	0.9655±0.0304	0.9776±0.0164	0.2140 (ns ↑ )	0.7140±0.0878	0.7024±0.1225	0.8243 (ns↓)
Virchow	GigaPath	0.9439±0.0323	0.9427±0.0302	0.6806 (ns↓)	0.9215±0.0298	0.9351±0.0254	0.2821 (ns ↑ )	0.7367±0.1773	0.8151±0.1301	0.1171 (ns ↑ )	0.6236±0.1394	0.6361±0.1566	0.7096 (ns ↑ )	0.9638±0.0275	0.9776±0.0164	0.0760 (ns ↑ )	0.6628±0.1162	0.7024±0.1225	0.3762 (ns ↑ )
Baseline Model	Compare Model	Macro																	
		AUC			Accuracy			Precision			Recall			Specificity			F1-Score		
		Base	Comp	*P*-value	Base	Comp	*P*-value	Base	Comp	*P*-value	Base	Comp	*P*-value	Base	Comp	*P*-value	Base	Comp	*P*-value
ResNet	UNI	0.9800±0.0171	0.9857±0.0089	0.2643 (ns ↑ )	0.9381±0.0279	0.9638±0.0162	0.0046 (**↑)	0.9249±0.0354	0.9655±0.0272	0.0006 (***↑)	0.9452±0.0431	0.9581±0.0457	0.4254 (ns ↑ )	0.9320±0.0334	0.9689±0.0247	0.0028 (**↑)	0.9343±0.0303	0.9607±0.0184	0.0042 (**↑)
ResNet	Virchow	0.9800±0.0171	0.9854±0.0100	0.0136 (*↑)	0.9381±0.0279	0.9623±0.0236	0.0060 (**↑)	0.9249±0.0354	0.9595±0.0382	0.0182 (*↑)	0.9452±0.0431	0.9613±0.0333	0.0934 (ns ↑ )	0.9320±0.0334	0.9633±0.0350	0.0464 (*↑)	0.9343±0.0303	0.9598±0.0248	0.0106 (*↑)
ResNet	GigaPath	0.9800±0.0171	0.9900±0.0090	0.0039 (**↑)	0.9381±0.0279	0.9668±0.0185	0.0005 (***↑)	0.9249±0.0354	0.9649±0.0230	0.0002 (***↑)	0.9452±0.0431	0.9645±0.0355	0.0388 (*↑)	0.9320±0.0334	0.9689±0.0209	0.0022 (**↑)	0.9343±0.0303	0.9642±0.0204	0.0009 (***↑)
UNI	Virchow	0.9857±0.0089	0.9854±0.0100	0.4010 (ns↓)	0.9638±0.0162	0.9623±0.0236	1.0000 (ns↓)	0.9655±0.0272	0.9595±0.0382	0.5803 (ns↓)	0.9581±0.0457	0.9613±0.0333	0.5492 (ns ↑ )	0.9689±0.0247	0.9633±0.0350	0.7677 (ns↓)	0.9607±0.0184	0.9598±0.0248	0.9516 (ns↓)
UNI	GigaPath	0.9857±0.0089	0.9900±0.0090	0.1498 (ns ↑ )	0.9638±0.0162	0.9668±0.0185	0.4561 (ns ↑ )	0.9655±0.0272	0.9649±0.0230	0.9276 (ns↓)	0.9581±0.0457	0.9645±0.0355	0.3431 (ns ↑ )	0.9689±0.0247	0.9689±0.0209	1.0000 (ns↓)	0.9607±0.0184	0.9642±0.0204	0.5181 (ns ↑ )
Virchow	GigaPath	0.9854±0.0100	0.9900±0.0090	0.6210 (ns ↑ )	0.9623±0.0236	0.9668±0.0185	0.6824 (ns ↑ )	0.9595±0.0382	0.9649±0.0230	0.5222 (ns ↑ )	0.9613±0.0333	0.9645±0.0355	1.0000 (ns ↑ )	0.9633±0.0350	0.9689±0.0209	0.7984 (ns ↑ )	0.9598±0.0248	0.9642±0.0204	0.5958 (ns ↑ )
DLZX																			
Baseline Model	Compare Model	Overall																	
		AUC			Accuracy			Precision			Recall			Specificity			F1-Score		
		Base	Comp	*P*-value	Base	Comp	*P*-value	Base	Comp	*P*-value	Base	Comp	*P*-value	Base	Comp	*P*-value	Base	Comp	*P*-value
ResNet	UNI	0.9080±0.0484	0.9483±0.0273	0.0007 (***↑)	0.8209±0.0367	0.8784±0.0521	<0.0001 (***↑)	0.7423±0.0797	0.8664±0.0719	0.0027 (**↑)	0.7142±0.0827	0.8481±0.0669	0.0002 (***↑)	0.9316±0.0178	0.9518±0.0194	0.0164 (*↑)	0.7129±0.0724	0.8420±0.0691	0.0003 (***↑)
ResNet	Virchow	0.9080±0.0484	0.9338±0.0485	0.0548 (ns ↑ )	0.8209±0.0367	0.8360±0.0682	0.1614 (ns ↑ )	0.7423±0.0797	0.8276±0.0815	0.0260 (*↑)	0.7142±0.0827	0.8347±0.0826	0.0029 (**↑)	0.9316±0.0178	0.9387±0.0251	0.4019 (ns ↑ )	0.7129±0.0724	0.8181±0.0851	0.0032 (**↑)
ResNet	GigaPath	0.9080±0.0484	0.9440±0.0284	0.0093 (**↑)	0.8209±0.0367	0.8882±0.0307	<0.0001 (***↑)	0.7423±0.0797	0.8962±0.0320	0.0005 (***↑)	0.7142±0.0827	0.8339±0.0555	0.0003 (***↑)	0.9316±0.0178	0.9562±0.0122	0.0010 (**↑)	0.7129±0.0724	0.8432±0.0504	0.0002 (***↑)
UNI	Virchow	0.9483±0.0273	0.9338±0.0485	0.0656 (ns↓)	0.8784±0.0521	0.8360±0.0682	<0.0001 (***↓)	0.8664±0.0719	0.8276±0.0815	0.2067 (ns↓)	0.8481±0.0669	0.8347±0.0826	0.5605 (ns↓)	0.9518±0.0194	0.9387±0.0251	0.0382 (*↓)	0.8420±0.0691	0.8181±0.0851	0.3090 (ns↓)
UNI	GigaPath	0.9483±0.0273	0.9440±0.0284	0.4822 (ns↓)	0.8784±0.0521	0.8882±0.0307	0.2431 (ns ↑ )	0.8664±0.0719	0.8962±0.0320	0.3489 (ns ↑ )	0.8481±0.0669	0.8339±0.0555	0.6535 (ns↓)	0.9518±0.0194	0.9562±0.0122	0.4636 (ns ↑ )	0.8420±0.0691	0.8432±0.0504	0.8027 (ns ↑ )
Virchow	GigaPath	0.9338±0.0485	0.9440±0.0284	0.4040 (ns ↑ )	0.8360±0.0682	0.8882±0.0307	<0.0001 (***↑)	0.8276±0.0815	0.8962±0.0320	0.0352 (*↑)	0.8347±0.0826	0.8339±0.0555	0.9518 (ns↓)	0.9387±0.0251	0.9562±0.0122	0.0175 (*↑)	0.8181±0.0851	0.8432±0.0504	0.3129 (ns ↑ )
Baseline Model	Compare Model	Negative																	
		AUC			Accuracy			Precision			Recall			Specificity			F1-Score		
		Base	Comp	*P*-value	Base	Comp	*P*-value	Base	Comp	*P*-value	Base	Comp	*P*-value	Base	Comp	*P*-value	Base	Comp	*P*-value
ResNet	UNI	0.9327±0.0591	0.9663±0.0423	0.0123 (*↑)	0.9154±0.0269	0.9603±0.0314	0.0006 (***↑)	0.8087±0.0988	0.9365±0.0760	0.0002 (***↑)	0.8733±0.0867	0.8956±0.0972	0.3416 (ns ↑ )	0.9276±0.0465	0.9805±0.0226	0.0002 (***↑)	0.8326±0.0482	0.9126±0.0720	0.0018 (**↑)
ResNet	Virchow	0.9327±0.0591	0.9388±0.0538	0.6936 (ns ↑ )	0.9154±0.0269	0.9180±0.0470	1.0000 (ns ↑ )	0.8087±0.0988	0.8496±0.1407	0.4232 (ns ↑ )	0.8733±0.0867	0.8311±0.1565	0.1130 (ns↓)	0.9276±0.0465	0.9444±0.0601	0.4436 (ns ↑ )	0.8326±0.0482	0.8256±0.1117	0.9422 (ns↓)
ResNet	GigaPath	0.9327±0.0591	0.9678±0.0391	0.0165 (*↑)	0.9154±0.0269	0.9651±0.0317	0.0001 (***↑)	0.8087±0.0988	0.9194±0.0827	0.0014 (**↑)	0.8733±0.0867	0.9356±0.0764	0.0097 (**↑)	0.9276±0.0465	0.9740±0.0255	0.0014 (**↑)	0.8326±0.0482	0.9261±0.0702	0.0002 (***↑)
UNI	Virchow	0.9663±0.0423	0.9388±0.0538	0.0066 (**↓)	0.9603±0.0314	0.9180±0.0470	0.0002 (***↓)	0.9365±0.0760	0.8496±0.1407	0.0003 (***↓)	0.8956±0.0972	0.8311±0.1565	0.0216 (*↓)	0.9805±0.0226	0.9444±0.0601	0.0002 (***↓)	0.9126±0.0720	0.8256±0.1117	0.0005 (***↓)
UNI	GigaPath	0.9663±0.0423	0.9678±0.0391	0.9560 (ns ↑ )	0.9603±0.0314	0.9651±0.0317	0.3435 (ns ↑ )	0.9365±0.0760	0.9194±0.0827	0.4263 (ns↓)	0.8956±0.0972	0.9356±0.0764	0.0697 (ns ↑ )	0.9805±0.0226	0.9740±0.0255	0.4989 (ns↓)	0.9126±0.0720	0.9261±0.0702	0.3978 (ns ↑ )
Virchow	GigaPath	0.9388±0.0538	0.9678±0.0391	0.0032 (**↑)	0.9180±0.0470	0.9651±0.0317	<0.0001 (***↑)	0.8496±0.1407	0.9194±0.0827	0.0061 (**↑)	0.8311±0.1565	0.9356±0.0764	<0.0001 (***↑)	0.9444±0.0601	0.9740±0.0255	0.0065 (**↑)	0.8256±0.1117	0.9261±0.0702	<0.0001 (***↑)
Baseline Model	Compare Model	ITC																	
		AUC			Accuracy			Precision			Recall			Specificity			F1-Score		
		Base	Comp	*P*-value	Base	Comp	*P*-value	Base	Comp	*P*-value	Base	Comp	*P*-value	Base	Comp	*P*-value	Base	Comp	*P*-value
ResNet	UNI	0.8828±0.1345	0.9785±0.0376	<0.0001 (***↑)	0.9554±0.0342	0.9802±0.0225	0.0297 (*↑)	0.6000±0.3944	0.8500±0.1995	0.0881 (ns ↑ )	0.5333±0.3752	0.8667±0.2194	<0.0001 (***↑)	0.9791±0.0267	0.9868±0.0186	0.5411 (ns ↑ )	0.5333±0.3407	0.8305±0.1628	0.0052 (**↑)
ResNet	Virchow	0.8828±0.1345	0.9737±0.0656	<0.0001 (***↑)	0.9554±0.0342	0.9901±0.0172	0.0003 (***↑)	0.6000±0.3944	0.9000±0.1610	0.0064 (**↑)	0.5333±0.3752	0.9667±0.1054	<0.0001 (***↑)	0.9791±0.0267	0.9921±0.0127	0.1773 (ns ↑ )	0.5333±0.3407	0.9267±0.1235	0.0003 (***↑)
ResNet	GigaPath	0.8828±0.1345	0.9420±0.0712	0.1336 (ns ↑ )	0.9554±0.0342	0.9802±0.0155	0.0231 (*↑)	0.6000±0.3944	0.9333±0.1405	0.0240 (*↑)	0.5333±0.3752	0.7167±0.2491	0.0308 (*↑)	0.9791±0.0267	0.9947±0.0111	0.1069 (ns ↑ )	0.5333±0.3407	0.7800±0.1573	0.0160 (*↑)
UNI	Virchow	0.9785±0.0376	0.9737±0.0656	0.4879 (ns↓)	0.9802±0.0225	0.9901±0.0172	0.0293 (*↑)	0.8500±0.1995	0.9000±0.1610	0.1182 (ns ↑ )	0.8667±0.2194	0.9667±0.1054	<0.0001 (***↑)	0.9868±0.0186	0.9921±0.0127	0.6272 (ns ↑ )	0.8305±0.1628	0.9267±0.1235	0.0293 (*↑)
UNI	GigaPath	0.9785±0.0376	0.9420±0.0712	0.0579 (ns↓)	0.9802±0.0225	0.9802±0.0155	1.0000 (ns↓)	0.8500±0.1995	0.9333±0.1405	0.3455 (ns ↑ )	0.8667±0.2194	0.7167±0.2491	<0.0001 (***↓)	0.9868±0.0186	0.9947±0.0111	0.3856 (ns ↑ )	0.8305±0.1628	0.7800±0.1573	0.7270 (ns↓)
Virchow	GigaPath	0.9737±0.0656	0.9420±0.0712	0.2155 (ns↓)	0.9901±0.0172	0.9802±0.0155	0.0712 (ns↓)	0.9000±0.1610	0.9333±0.1405	0.9743 (ns ↑ )	0.9667±0.1054	0.7167±0.2491	<0.0001 (***↓)	0.9921±0.0127	0.9947±0.0111	1.0000 (ns ↑ )	0.9267±0.1235	0.7800±0.1573	0.0712 (ns↓)
Baseline Model	Compare Model	Micro																	
		AUC			Accuracy			Precision			Recall			Specificity			F1-Score		
		Base	Comp	*P*-value	Base	Comp	*P*-value	Base	Comp	*P*-value	Base	Comp	*P*-value	Base	Comp	*P*-value	Base	Comp	*P*-value
ResNet	UNI	0.8673±0.0829	0.8970±0.0759	0.4197 (ns ↑ )	0.8704±0.0486	0.9032±0.0423	0.0574 (ns ↑ )	0.6617±0.1934	0.7693±0.1746	0.2590 (ns ↑ )	0.5286±0.1911	0.7000±0.2177	0.0142 (*↑)	0.9426±0.0390	0.9461±0.0536	1.0000 (ns ↑ )	0.5769±0.1865	0.7064±0.1470	0.0320 (*↑)
ResNet	Virchow	0.8673±0.0829	0.8881±0.0917	0.6823 (ns ↑ )	0.8704±0.0486	0.8732±0.0556	1.0000 (ns ↑ )	0.6617±0.1934	0.6495±0.1897	0.6005 (ns↓)	0.5286±0.1911	0.6571±0.2626	0.0305 (*↑)	0.9426±0.0390	0.9185±0.0686	0.0767 (ns↓)	0.5769±0.1865	0.6269±0.2015	0.2931 (ns ↑ )
ResNet	GigaPath	0.8673±0.0829	0.9121±0.0598	0.0714 (ns ↑ )	0.8704±0.0486	0.9130±0.0332	0.0136 (*↑)	0.6617±0.1934	0.8079±0.1632	0.1898 (ns ↑ )	0.5286±0.1911	0.7571±0.2026	0.0004 (***↑)	0.9426±0.0390	0.9460±0.0573	1.0000 (ns ↑ )	0.5769±0.1865	0.7437±0.1175	0.0041 (**↑)
UNI	Virchow	0.8970±0.0759	0.8881±0.0917	0.5886 (ns↓)	0.9032±0.0423	0.8732±0.0556	0.0372 (*↓)	0.7693±0.1746	0.6495±0.1897	0.0440 (*↓)	0.7000±0.2177	0.6571±0.2626	0.2973 (ns↓)	0.9461±0.0536	0.9185±0.0686	0.0419 (*↓)	0.7064±0.1470	0.6269±0.2015	0.0813 (ns↓)
UNI	GigaPath	0.8970±0.0759	0.9121±0.0598	0.2246 (ns ↑ )	0.9032±0.0423	0.9130±0.0332	0.6180 (ns ↑ )	0.7693±0.1746	0.8079±0.1632	0.7801 (ns ↑ )	0.7000±0.2177	0.7571±0.2026	0.1414 (ns ↑ )	0.9461±0.0536	0.9460±0.0573	1.0000 (ns↓)	0.7064±0.1470	0.7437±0.1175	0.3517 (ns ↑ )
Virchow	GigaPath	0.8881±0.0917	0.9121±0.0598	0.1080 (ns ↑ )	0.8732±0.0556	0.9130±0.0332	0.0162 (*↑)	0.6495±0.1897	0.8079±0.1632	0.0373 (*↑)	0.6571±0.2626	0.7571±0.2026	0.0783 (ns ↑ )	0.9185±0.0686	0.9460±0.0573	0.0705 (ns ↑ )	0.6269±0.2015	0.7437±0.1175	0.0245 (*↑)
Baseline Model	Compare Model	Macro																	
		AUC			Accuracy			Precision			Recall			Specificity			F1-Score		
		Base	Comp	*P*-value	Base	Comp	*P*-value	Base	Comp	*P*-value	Base	Comp	*P*-value	Base	Comp	*P*-value	Base	Comp	*P*-value
ResNet	UNI	0.9493±0.0297	0.9515±0.0392	0.6019 (ns ↑ )	0.9006±0.0468	0.9131±0.0499	0.5535 (ns ↑ )	0.8989±0.0571	0.9098±0.0458	0.5477 (ns ↑ )	0.9214±0.0481	0.9303±0.0735	0.8248 (ns ↑ )	0.8771±0.0754	0.8937±0.0531	0.4415 (ns ↑ )	0.9089±0.0424	0.9186±0.0490	0.4923 (ns ↑ )
ResNet	Virchow	0.9493±0.0297	0.9346±0.0616	0.1465 (ns↓)	0.9006±0.0468	0.8907±0.0623	0.6792 (ns↓)	0.8989±0.0571	0.9114±0.0560	0.5490 (ns ↑ )	0.9214±0.0481	0.8838±0.1098	0.0671 (ns↓)	0.8771±0.0754	0.8999±0.0606	0.3323 (ns ↑ )	0.9089±0.0424	0.8933±0.0660	0.4774 (ns↓)
ResNet	GigaPath	0.9493±0.0297	0.9541±0.0326	0.4009 (ns ↑ )	0.9006±0.0468	0.9181±0.0401	0.3548 (ns ↑ )	0.8989±0.0571	0.9242±0.0522	0.1628 (ns ↑ )	0.9214±0.0481	0.9262±0.0683	1.0000 (ns ↑ )	0.8771±0.0754	0.9101±0.0654	0.1127 (ns ↑ )	0.9089±0.0424	0.9229±0.0394	0.3111 (ns ↑ )
UNI	Virchow	0.9515±0.0392	0.9346±0.0616	0.0433 (*↓)	0.9131±0.0499	0.8907±0.0623	0.1623 (ns↓)	0.9098±0.0458	0.9114±0.0560	1.0000 (ns ↑ )	0.9303±0.0735	0.8838±0.1098	0.0086 (**↓)	0.8937±0.0531	0.8999±0.0606	1.0000 (ns ↑ )	0.9186±0.0490	0.8933±0.0660	0.0835 (ns↓)
UNI	GigaPath	0.9515±0.0392	0.9541±0.0326	0.6902 (ns ↑ )	0.9131±0.0499	0.9181±0.0401	0.8436 (ns ↑ )	0.9098±0.0458	0.9242±0.0522	0.3844 (ns ↑ )	0.9303±0.0735	0.9262±0.0683	1.0000 (ns↓)	0.8937±0.0531	0.9101±0.0654	0.5440 (ns ↑ )	0.9186±0.0490	0.9229±0.0394	0.7505 (ns ↑ )
Virchow	GigaPath	0.9346±0.0616	0.9541±0.0326	0.0218 (*↑)	0.8907±0.0623	0.9181±0.0401	0.1284 (ns ↑ )	0.9114±0.0560	0.9242±0.0522	0.4939 (ns ↑ )	0.8838±0.1098	0.9262±0.0683	0.0460 (*↑)	0.8999±0.0606	0.9101±0.0654	0.8050 (ns ↑ )	0.8933±0.0660	0.9229±0.0394	0.0755 (ns ↑ )
YDSY																			
Baseline Model	Compare Model	Overall																	
		AUC			Accuracy			Precision			Recall			Specificity			F1-Score		
		Base	Comp	*P*-value	Base	Comp	*P*-value	Base	Comp	*P*-value	Base	Comp	*P*-value	Base	Comp	*P*-value	Base	Comp	*P*-value
ResNet	UNI	0.9040±0.0431	0.9352±0.0433	0.0004 (***↑)	0.7938±0.0546	0.8672±0.0563	<0.0001 (***↑)	0.7734±0.1448	0.8807±0.0946	0.0141 (*↑)	0.7535±0.1062	0.8330±0.0871	0.0087 (**↑)	0.9238±0.0204	0.9484±0.0190	0.0089 (**↑)	0.7466±0.1157	0.8414±0.0914	0.0061 (**↑)
ResNet	Virchow	0.9040±0.0431	0.9240±0.0472	0.0197 (*↑)	0.7938±0.0546	0.8240±0.0702	0.0268 (*↑)	0.7734±0.1448	0.8431±0.0612	0.1604 (ns ↑ )	0.7535±0.1062	0.8238±0.0631	0.0285 (*↑)	0.9238±0.0204	0.9379±0.0226	0.1627 (ns ↑ )	0.7466±0.1157	0.8172±0.0595	0.0550 (ns ↑ )
ResNet	GigaPath	0.9040±0.0431	0.9424±0.0306	<0.0001 (***↑)	0.7938±0.0546	0.8471±0.0594	<0.0001 (***↑)	0.7734±0.1448	0.8566±0.0859	0.0048 (**↑)	0.7535±0.1062	0.8228±0.0904	0.0142 (*↑)	0.9238±0.0204	0.9442±0.0185	0.0285 (*↑)	0.7466±0.1157	0.8229±0.0868	0.0080 (**↑)
UNI	Virchow	0.9352±0.0433	0.9240±0.0472	0.2103 (ns↓)	0.8672±0.0563	0.8240±0.0702	0.0001 (***↓)	0.8807±0.0946	0.8431±0.0612	0.2154 (ns↓)	0.8330±0.0871	0.8238±0.0631	0.9356 (ns↓)	0.9484±0.0190	0.9379±0.0226	0.1969 (ns↓)	0.8414±0.0914	0.8172±0.0595	0.4788 (ns↓)
UNI	GigaPath	0.9352±0.0433	0.9424±0.0306	0.7327 (ns ↑ )	0.8672±0.0563	0.8471±0.0594	0.0374 (*↓)	0.8807±0.0946	0.8566±0.0859	0.6500 (ns↓)	0.8330±0.0871	0.8228±0.0904	0.6514 (ns↓)	0.9484±0.0190	0.9442±0.0185	0.5337 (ns↓)	0.8414±0.0914	0.8229±0.0868	0.5293 (ns↓)
Virchow	GigaPath	0.9240±0.0472	0.9424±0.0306	0.3803 (ns ↑ )	0.8240±0.0702	0.8471±0.0594	0.0268 (*↑)	0.8431±0.0612	0.8566±0.0859	0.3926 (ns ↑ )	0.8238±0.0631	0.8228±0.0904	0.7848 (ns↓)	0.9379±0.0226	0.9442±0.0185	0.4418 (ns ↑ )	0.8172±0.0595	0.8229±0.0868	0.8164 (ns ↑ )
Baseline Model	Compare Model	Negative																	
		AUC			Accuracy			Precision			Recall			Specificity			F1-Score		
		Base	Comp	*P*-value	Base	Comp	*P*-value	Base	Comp	*P*-value	Base	Comp	*P*-value	Base	Comp	*P*-value	Base	Comp	*P*-value
ResNet	UNI	0.9195±0.0575	0.9724±0.0417	<0.0001 (***↑)	0.8934±0.0626	0.9435±0.0417	0.0067 (**↑)	0.7595±0.1613	0.8538±0.1013	0.0160 (*↑)	0.8571±0.1650	0.9286±0.0753	0.0940 (ns ↑ )	0.9037±0.0716	0.9476±0.0404	0.0166 (*↑)	0.7931±0.1247	0.8877±0.0809	0.0117 (*↑)
ResNet	Virchow	0.9195±0.0575	0.9794±0.0249	<0.0001 (***↑)	0.8934±0.0626	0.9369±0.0429	0.0226 (*↑)	0.7595±0.1613	0.8324±0.0952	0.0592 (ns ↑ )	0.8571±0.1650	0.9286±0.1543	0.0972 (ns ↑ )	0.9037±0.0716	0.9385±0.0379	0.0641 (ns ↑ )	0.7931±0.1247	0.8707±0.0983	0.0301 (*↑)
ResNet	GigaPath	0.9195±0.0575	0.9818±0.0177	<0.0001 (***↑)	0.8934±0.0626	0.9435±0.0386	0.0021 (**↑)	0.7595±0.1613	0.8470±0.0996	0.0082 (**↑)	0.8571±0.1650	0.9446±0.0717	0.0394 (*↑)	0.9037±0.0716	0.9433±0.0417	0.0126 (*↑)	0.7931±0.1247	0.8903±0.0725	0.0041 (**↑)
UNI	Virchow	0.9724±0.0417	0.9794±0.0249	0.5790 (ns ↑ )	0.9435±0.0417	0.9369±0.0429	0.8414 (ns↓)	0.8538±0.1013	0.8324±0.0952	0.6013 (ns↓)	0.9286±0.0753	0.9286±0.1543	1.0000 (ns↓)	0.9476±0.0404	0.9385±0.0379	0.8116 (ns↓)	0.8877±0.0809	0.8707±0.0983	0.6711 (ns↓)
UNI	GigaPath	0.9724±0.0417	0.9818±0.0177	0.7808 (ns ↑ )	0.9435±0.0417	0.9435±0.0386	1.0000 (ns↓)	0.8538±0.1013	0.8470±0.0996	0.7660 (ns↓)	0.9286±0.0753	0.9446±0.0717	0.4505 (ns ↑ )	0.9476±0.0404	0.9433±0.0417	1.0000 (ns↓)	0.8877±0.0809	0.8903±0.0725	1.0000 (ns ↑ )
Virchow	GigaPath	0.9794±0.0249	0.9818±0.0177	0.7995 (ns ↑ )	0.9369±0.0429	0.9435±0.0386	0.8234 (ns ↑ )	0.8324±0.0952	0.8470±0.0996	0.6804 (ns ↑ )	0.9286±0.1543	0.9446±0.0717	0.4532 (ns ↑ )	0.9385±0.0379	0.9433±0.0417	1.0000 (ns ↑ )	0.8707±0.0983	0.8903±0.0725	0.5910 (ns ↑ )
Baseline Model	Compare Model	ITC																	
		AUC			Accuracy			Precision			Recall			Specificity			F1-Score		
		Base	Comp	*P*-value	Base	Comp	*P*-value	Base	Comp	*P*-value	Base	Comp	*P*-value	Base	Comp	*P*-value	Base	Comp	*P*-value
ResNet	UNI	0.9403±0.0934	0.9589±0.0663	0.0035 (**↑)	0.9768±0.0316	0.9768±0.0316	1.0000 (ns↓)	0.8667±0.3220	0.9333±0.2108	1.0000 (ns ↑ )	0.7833±0.3338	0.7833±0.2364	1.0000 (ns↓)	0.9929±0.0151	0.9929±0.0226	1.0000 (ns↓)	0.8067±0.3086	0.8333±0.2067	1.0000 (ns ↑ )
ResNet	Virchow	0.9403±0.0934	0.9405±0.1389	0.1325 (ns ↑ )	0.9768±0.0316	0.9868±0.0171	0.4561 (ns ↑ )	0.8667±0.3220	0.9750±0.0791	0.3000 (ns ↑ )	0.7833±0.3338	0.8500±0.2415	0.1213 (ns ↑ )	0.9929±0.0151	0.9964±0.0113	1.0000 (ns ↑ )	0.8067±0.3086	0.8857±0.1575	0.1710 (ns ↑ )
ResNet	GigaPath	0.9403±0.0934	0.9684±0.0666	<0.0001 (***↑)	0.9768±0.0316	0.9768±0.0316	1.0000 (ns↓)	0.8667±0.3220	0.9000±0.3162	0.5109 (ns ↑ )	0.7833±0.3338	0.7333±0.3352	<0.0001 (***↓)	0.9929±0.0151	0.9964±0.0113	1.0000 (ns ↑ )	0.8067±0.3086	0.7933±0.3118	0.5109 (ns↓)
UNI	Virchow	0.9589±0.0663	0.9405±0.1389	0.4818 (ns↓)	0.9768±0.0316	0.9868±0.0171	0.4561 (ns ↑ )	0.9333±0.2108	0.9750±0.0791	0.2950 (ns ↑ )	0.7833±0.2364	0.8500±0.2415	0.1222 (ns ↑ )	0.9929±0.0226	0.9964±0.0113	1.0000 (ns ↑ )	0.8333±0.2067	0.8857±0.1575	0.1680 (ns ↑ )
UNI	GigaPath	0.9589±0.0663	0.9684±0.0666	0.9177 (ns ↑ )	0.9768±0.0316	0.9768±0.0316	1.0000 (ns↓)	0.9333±0.2108	0.9000±0.3162	0.6303 (ns↓)	0.7833±0.2364	0.7333±0.3352	<0.0001 (***↓)	0.9929±0.0226	0.9964±0.0113	1.0000 (ns ↑ )	0.8333±0.2067	0.7933±0.3118	0.6303 (ns↓)
Virchow	GigaPath	0.9405±0.1389	0.9684±0.0666	0.6630 (ns ↑ )	0.9868±0.0171	0.9768±0.0316	0.3708 (ns↓)	0.9750±0.0791	0.9000±0.3162	0.6282 (ns↓)	0.8500±0.2415	0.7333±0.3352	<0.0001 (***↓)	0.9964±0.0113	0.9964±0.0113	1.0000 (ns↓)	0.8857±0.1575	0.7933±0.3118	0.0610 (ns↓)
Baseline Model	Compare Model	Micro																	
		AUC			Accuracy			Precision			Recall			Specificity			F1-Score		
		Base	Comp	*P*-value	Base	Comp	*P*-value	Base	Comp	*P*-value	Base	Comp	*P*-value	Base	Comp	*P*-value	Base	Comp	*P*-value
ResNet	UNI	0.8240±0.0908	0.8722±0.0876	0.0800 (ns ↑ )	0.8370±0.0434	0.9103±0.0416	0.0005 (***↑)	0.5859±0.2878	0.8363±0.1580	0.0049 (**↑)	0.4933±0.2314	0.7067±0.2095	0.0037 (**↑)	0.9172±0.0705	0.9590±0.0432	0.0295 (*↑)	0.5077±0.2033	0.7404±0.1377	0.0007 (***↑)
ResNet	Virchow	0.8240±0.0908	0.8586±0.0744	0.1694 (ns ↑ )	0.8370±0.0434	0.8539±0.0722	0.4257 (ns ↑ )	0.5859±0.2878	0.6421±0.1860	0.8412 (ns ↑ )	0.4933±0.2314	0.7033±0.1651	0.0103 (*↑)	0.9172±0.0705	0.8887±0.0836	0.1586 (ns↓)	0.5077±0.2033	0.6530±0.1340	0.1024 (ns ↑ )
ResNet	GigaPath	0.8240±0.0908	0.8780±0.0759	0.0373 (*↑)	0.8370±0.0434	0.8902±0.0591	0.0161 (*↑)	0.5859±0.2878	0.7649±0.2175	0.1147 (ns ↑ )	0.4933±0.2314	0.7600±0.1377	0.0008 (***↑)	0.9172±0.0705	0.9223±0.0822	1.0000 (ns ↑ )	0.5077±0.2033	0.7331±0.1118	0.0032 (**↑)
UNI	Virchow	0.8722±0.0876	0.8586±0.0744	0.5865 (ns↓)	0.9103±0.0416	0.8539±0.0722	0.0023 (**↓)	0.8363±0.1580	0.6421±0.1860	0.0005 (***↓)	0.7067±0.2095	0.7033±0.1651	1.0000 (ns↓)	0.9590±0.0432	0.8887±0.0836	<0.0001 (***↓)	0.7404±0.1377	0.6530±0.1340	0.0557 (ns↓)
UNI	GigaPath	0.8722±0.0876	0.8780±0.0759	0.8727 (ns ↑ )	0.9103±0.0416	0.8902±0.0591	0.1679 (ns↓)	0.8363±0.1580	0.7649±0.2175	0.0476 (*↓)	0.7067±0.2095	0.7600±0.1377	0.1808 (ns ↑ )	0.9590±0.0432	0.9223±0.0822	0.0106 (*↓)	0.7404±0.1377	0.7331±0.1118	0.5682 (ns↓)
Virchow	GigaPath	0.8586±0.0744	0.8780±0.0759	0.4117 (ns ↑ )	0.8539±0.0722	0.8902±0.0591	0.0989 (ns ↑ )	0.6421±0.1860	0.7649±0.2175	0.0640 (ns ↑ )	0.7033±0.1651	0.7600±0.1377	0.2309 (ns ↑ )	0.8887±0.0836	0.9223±0.0822	0.0721 (ns ↑ )	0.6530±0.1340	0.7331±0.1118	0.0741 (ns ↑ )
Baseline Model	Compare Model	Macro																	
		AUC			Accuracy			Precision			Recall			Specificity			F1-Score		
		Base	Comp	*P*-value	Base	Comp	*P*-value	Base	Comp	*P*-value	Base	Comp	*P*-value	Base	Comp	*P*-value	Base	Comp	*P*-value
ResNet	UNI	0.9320±0.0508	0.9371±0.0352	0.6937 (ns ↑ )	0.8803±0.0634	0.9038±0.0326	0.2044 (ns ↑ )	0.8817±0.0642	0.8993±0.0530	0.5657 (ns ↑ )	0.8800±0.0878	0.9133±0.0632	0.1689 (ns ↑ )	0.8812±0.0673	0.8942±0.0641	0.5116 (ns ↑ )	0.8791±0.0645	0.9041±0.0343	0.2304 (ns ↑ )
ResNet	Virchow	0.9320±0.0508	0.9176±0.0462	0.3800 (ns↓)	0.8803±0.0634	0.8704±0.0429	0.7551 (ns↓)	0.8817±0.0642	0.9229±0.0513	0.1806 (ns ↑ )	0.8800±0.0878	0.8133±0.1080	0.0142 (*↓)	0.8812±0.0673	0.9279±0.0554	0.0636 (ns ↑ )	0.8791±0.0645	0.8593±0.0539	0.4307 (ns↓)
ResNet	GigaPath	0.9320±0.0508	0.9414±0.0338	0.6142 (ns ↑ )	0.8803±0.0634	0.8837±0.0551	1.0000 (ns ↑ )	0.8817±0.0642	0.9144±0.0539	0.3216 (ns ↑ )	0.8800±0.0878	0.8533±0.1398	0.1467 (ns↓)	0.8812±0.0673	0.9146±0.0608	0.1697 (ns ↑ )	0.8791±0.0645	0.8746±0.0720	0.9760 (ns↓)
UNI	Virchow	0.9371±0.0352	0.9176±0.0462	0.1128 (ns↓)	0.9038±0.0326	0.8704±0.0429	0.0509 (ns↓)	0.8993±0.0530	0.9229±0.0513	0.3290 (ns ↑ )	0.9133±0.0632	0.8133±0.1080	0.0001 (***↓)	0.8942±0.0641	0.9279±0.0554	0.0991 (ns ↑ )	0.9041±0.0343	0.8593±0.0539	0.0320 (*↓)
UNI	GigaPath	0.9371±0.0352	0.9414±0.0338	0.2224 (ns ↑ )	0.9038±0.0326	0.8837±0.0551	0.1700 (ns↓)	0.8993±0.0530	0.9144±0.0539	0.5036 (ns ↑ )	0.9133±0.0632	0.8533±0.1398	0.0121 (*↓)	0.8942±0.0641	0.9146±0.0608	0.1850 (ns ↑ )	0.9041±0.0343	0.8746±0.0720	0.1695 (ns↓)
Virchow	GigaPath	0.9176±0.0462	0.9414±0.0338	0.5821 (ns ↑ )	0.8704±0.0429	0.8837±0.0551	0.6011 (ns ↑ )	0.9229±0.0513	0.9144±0.0539	0.7284 (ns↓)	0.8133±0.1080	0.8533±0.1398	0.1141 (ns ↑ )	0.9279±0.0554	0.9146±0.0608	0.3926 (ns↓)	0.8593±0.0539	0.8746±0.0720	0.3819 (ns ↑ )

*: *P* < 0.05；**: *P* < 0.01；***: *P* < 0.001；*ns* no statistical significance. ↑: an increase in metrics of the compare model relative to the baseline model. ↓: a decrease in metrics of the compare model relative to the baseline model. A two-sided paired permutation test with 10,000 permutations was applied.

In overall four-class classification task on the NIMM dataset, the UNI and GigaPath models significantly outperformed the conventional ResNet baseline. Such superiority was mainly reflected in the marked improvements of classification accuracy (*P* < 0.001) and specificity (*P* < 0.01). There was no statistically significant difference between the Virchow and ResNet models. Further comparisons among the three pathological foundation models demonstrated that UNI and GigaPath achieved comparable performance, and both were statistically superior to Virchow in terms of accuracy (*P* < 0.01) and recall (*P* < 0.05).

In tasks for individual LNM categories on the NIMM dataset, the performance advantages of pathology-oriented pre-trained models exhibited obvious task dependence and model heterogeneity. Specifically, in the detection tasks of ITCs and micro-metastasis, only GigaPath yielded statistically significant performance improvements compared with ResNet. It achieved a marked increase in the recall of ITCs (*P* < 0.01) and notable improvements in the accuracy and specificity of micro-metastatic lesions (*P* < 0.05), thereby substantially reducing the risk of missed and misdiagnosed minimal metastatic lesions. Specifically, UNI and GigaPath achieved near-perfect performance in the negative identification task (AUC = 1.0000, *P* < 0.0001) and were significantly better than ResNet and Virchow, effectively avoiding misdiagnosis of negative samples. In macro-metastasis detection, all three pathological models surpassed ResNet; notably, UNI and GigaPath presented greater improvements and higher statistical significance, showing dominant advantages in accuracy, precision, and F1-score. Overall, GigaPath delivered consistent and significant improvements across all stratified tasks, making it the most robust feature extractor. UNI showed competitive performance mainly in negative recognition and macro-metastasis detection, while Virchow exhibited limited overall superiority and only achieved marginal benefits in the macro-metastasis task.

In the overall four-class classification task of LNM in both DLZX and YDSY datasets, the performance of different feature extractors was generally consistent with that on the NIMM dataset. Specifically, compared with ResNet, UNI and GigaPath achieved the most comprehensive improvements, with statistically significant differences in core metrics including accuracy, precision, recall, specificity, and F1-score. On the YDSY dataset, both models also exhibited significant advantages for AUC. Virchow significantly outperformed ResNet in precision, recall and F1-score on the DLZX dataset, as well as in AUC, accuracy and recall on the YDSY dataset. Among the three pathological foundation models, both UNI and GigaPath achieved statistically superior performance over Virchow in accuracy (*P* < 0.001) and specificity (*P* < 0.05).

In tasks for individual LNM categories on the DLZX and YDSY datasets, our model exhibits obvious task dependence and model heterogeneity. In ITCs detection, the pathological foundation models as a whole significantly outperformed ResNet. Notably, GigaPath consistently showed significantly lower recall across both datasets, indicating a clear limitation in identifying extremely rare tumor cells, whereas UNI and Virchow exhibited more robust performance. For micro-metastasis detection, UNI and GigaPath performed optimally in both datasets, significantly outperforming ResNet in recall and F1-score; Virchow only achieved a limited improvement in recall on the YDSY dataset, reflecting weaker overall performance. For negative lymph node identification, UNI and GigaPath achieved the strongest results in both datasets, significantly surpassing ResNet across core metrics including AUC, accuracy, precision, and specificity. In macro-metastasis identification, the three pathological foundation models yielded similar performance to the traditional ResNet model. UNI and GigaPath significantly outperformed Virchow in both AUC and recall on DLZX; in contrast, UNI showed a statistically significant advantage over both Virchow and GigaPath in recall on YDSY. Overall, UNI achieved stable and statistically significant performance improvements across all classification tasks, emerging as the most robust feature extractor. GigaPath achieved statistically significant advantages in the negative and micro-metastasis categories across the two external cohorts, as well as in the macro-metastasis task on the DLZX dataset. Virchow only exhibited statistical advantages in ITCs detection, with its overall performance considerably inferior to UNI and GigaPath.

Taken together, across multi-center datasets (NIMM/DLZX/YDSY) and in both the overall four-classification and each LNM classification task, UNI and GigaPath achieved optimal and robust overall performance, significantly outperforming ResNet and Virchow.

### Qualitative comparison of visualization

**Fig. 3:**
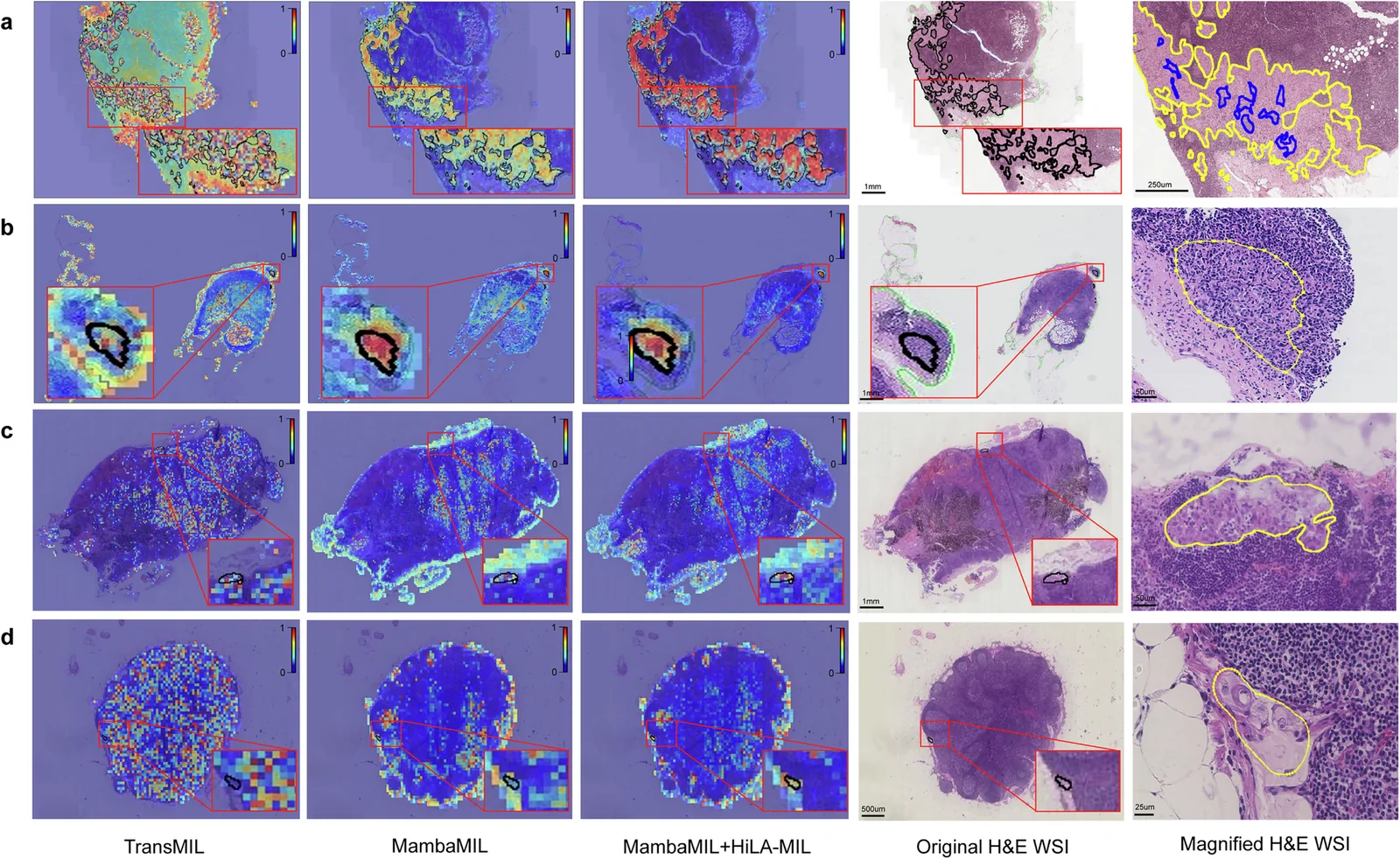
Visualization of images with the TransMIL, MambaMIL, and MambaMIL + HiLA-MIL models, as well as the original H&E WSIs and related magnified versions. **a** Macro-metastasis. **b** Micro-metastasis. **c** Micro-metastasis. **d** ITCs. Prediction score [0–1]. Red (value close to 1) represents high attention scores, blue (value close to 0) represents low attention scores, and the cancer metastasis areas annotated by pathologists are marked with black and yellow borders.

Figure 3 shows the visualization images of the TransMIL, MambaMIL, and MambaMIL + HiLA-MIL models, as well as the original and magnified H&E-stained WSIs. As compared to TransMIL, MambaMIL achieved higher attention scores for actual lesion areas and lower attention scores for non-lesion areas. The performance of the MambaMIL + HiLA-MIL model was further improved as compared to MambaMIL, with higher attention scores of lesion areas and lower attention scores of non-lesion areas, demonstrating more precise correspondence to actual lesions. The MambaMIL + HiLA-MIL model achieved higher specificity (0.9581 ± 0.0136) for overall four-class classification of LNM, while the sensitivity was slightly lower (0.7434 ± 0.0228).

Through analysis of misclassified cases, false-positive predictions were primarily caused by three categories of factors: preparation artifacts, special structures, and normal or benign histological structures. Preparation artifacts included uneven staining, air bubbles, and knife marks in certain slides; special structures included carbon deposits, necrosis, and mucoid degeneration; and normal or benign histological structures included germinal centers, compressed lymphocyte clusters, and hyperplastic sinus histiocytes. These structures displayed strong morphological resemblance to metastatic tumor foci and thus caused model over-recognition. In contrast, false-negative predictions were mainly due to atypical tumor cell morphology caused by preoperative chemotherapy, poor differentiation, and low tumor cell density (Supplementary Fig. 6). In such instances, cellular atypia was inconspicuous and prone to being neglected, leading to under-recognition by the model.

## Discussion

This study included a total of 662 multi-cancer lymph node WSIs (309 macro-metastasis, 82 micro-metastasis, 40 ITCs, and 231 negative), which represents one of the most balanced cohorts in the field of LNM four-class classification studies to date, allowing for a more objective and realistic assessment of the model’s performance. Experimental results demonstrate that when validated on the internal NIMM dataset and external DLZX and YDSY datasets with four distinct feature extractors (ResNet, UNI, Virchow, and GigaPath), the MambaMIL + HiLA-MIL model comprehensively outperformed AB-MIL, CLAM-SB, CLAM-MB, DSMIL, TransMIL, and MambaMIL in the overall four-class classification of multi-cancer LNM, with most performance improvements reaching statistical significance.

Campanella et al.^24^ reported an AUC of 0.965 using a MIL model with an internal test set of breast cancer lymph node WSIs from a single center. Their research did not focus on the precise four-class classification of LNM, as 15% of the 9894 breast cancer lymph node WSIs were used as the test set, which included 1081 negative cases and 403 positive cases of LNM. However, the distribution of macro-metastasis, micro-metastasis, and ITCs among the positive cases was not specified, indicating a significant issue of class imbalance in their cohort, which is a key factor contributing to the inflated performance metrics. A similar problem was observed in the study by Lu et al.^25^, which included 399 WSIs from the CAMELYON16 dataset (no ITCs cases) and 500 WSIs from the CAMELYON17 dataset (only 16 ITCs cases). Only 10% of the combined dataset was used as the internal test set, which means that the test set included only 1 or 2 ITCs cases. The CLAM model achieved an AUC of 0.953 with the internal test sets CAMELYON16 and CAMELYON17. The reason for this high performance can also be attributed to class imbalance. In contrast, the proposed MambaMIL + HiLA-MIL model in this study achieved an AUC of at least 0.9695 ± 0.0131 across four different feature extractors on the internal dataset. On the two external datasets, the AUC values fluctuated between 0.90 and 0.95 depending on the feature extractor adopted; nevertheless, our model still outperformed six baseline models, including CLAM. These findings collectively indicate that class imbalance in datasets can significantly affect model performance. The MambaMIL + HiLA-MIL model, which was developed using a more balanced dataset, demonstrated superior performance than other weakly supervised models.

For LNM classification, like other weakly supervised deep learning algorithms, the overall performance of the MambaMIL + HiLA-MIL model was comparable to that of fully supervised deep learning models. As compared to other weakly supervised deep learning algorithms, the most significant breakthrough of the MambaMIL + HiLA-MIL model is top performance with identification of ITCs, the smallest metastasis type. In terms of AUC performance, Huang et al.^3^ developed an end-to-end weakly supervised method, called Enhanced Streaming CNN (ESCNN), which was tested with 983 WSIs of gastric cancer lymph nodes from a single center (861 negative, 36 macro-metastasis, 58 micro-metastasis, and 28 ITCs). The AUC reached 0.9936 with a test set of 201 WSIs (20% of the dataset). Unfortunately, this study did not list the performance metrics for each classification category, but rather established two test subsets, each including all 861 negative lymph nodes, with one subset containing 58 micro-metastasis lymph nodes and the other containing 28 ITCs lymph nodes. The ESCNN model performed well with the micro-metastasis and ITCs test subsets, achieving AUC values of 0.9940 and 0.9228, respectively. The MambaMIL + HiLA-MIL model proposed in this study achieved an AUC of at least 0.9403 ± 0.0934 for ITCs across four different feature extractors on the internal NIMM dataset and the external YDSY dataset, which completely outperformed the single-center ESCNN model. Although our model did not outperform the performance of ESCNN model in micro-metastasis discrimination, the study artificially transformed the four-class LNM classification problem into a binary classification problem. Chuang et al.^34^ used a series of data augmentation methods to develop a model for detection of LNM of colorectal cancer in a manner similar to MIL. In a single-center test set of colorectal cancer lymph nodes (500 negative, 39 macro-metastasis, 56 micro-metastasis, and 5 ITCs), the model performed well for identification of macro-metastasis and micro-metastasis, with slice-level AUC values of 0.9993 and 0.9956, respectively. However, for identification of ITCs, the AUC dropped to 0.7828. Li et al.^26^ also tested a DT-DSMIL model with WSIs of colorectal cancer lymph nodes and encountered the same issue as Chuang et al. The DT-DSMIL model performed poorly for identification of ITCs, with an AUC of 0.8120 and an accuracy of only 27.3%. Under multiple centers and various feature extractors, the MambaMIL + HiLA-MIL model achieved an AUC of at least 0.9403 ± 0.0934 and an accuracy above 0.9487 ± 0.0190 for detection of ITCs, which is significantly better than the performance of the previous two studies.

In terms of sensitivity and specificity, Challa et al.^35^ directly applied an automated LNM AI tool, the Visiopharm Integrated System (VIS), to detect LNM of breast cancer. In the sentinel lymph node validation cohort, the VIS algorithm detected all 46 metastasis, including 19 macro-metastasis, 26 micro-metastasis, and 1 ITCs, achieving a sensitivity of 100%, but with a specificity of only 41.5%. In contrast, the MambaMIL + HiLA-MIL model achieved a specificity of at least 0.9631 ± 0.0213 for ITCs detection across multiple centers and multiple feature extractors, which is significantly superior to the VIS algorithm. Subsequently, Dooijeweert et al.^36^ applied the VIS algorithm in a non-randomized, single-center clinical trial involving 190 sentinel lymph nodes to evaluate LNM of breast cancer. Although the primary outcome of this study was that, with AI assistance, pathologists achieved a significantly reduced adjusted relative risk using immunohistochemistry, a shorter diagnostic time, and improved detection sensitivity, the sensitivity for detection of ITCs was only 44.4%. In comparison, even in the worst-performing configuration with the ResNet feature extractor on the external DLZX dataset, the recall/sensitivity of the proposed MambaMIL + HiLA-MIL model for ITCs detection still attained 0.5333 ± 0.3752. Retamero et al.^37^ evaluated the Paige Breast Cancer Lymph Node (Paige BLN) system, which is based on a weakly supervised deep learning algorithm, with a dataset of 167 WSIs of breast cancer lymph nodes (98 negative and 69 positive cases). For ITCs detection, the independent sensitivity of Paige BLN was 78%. By comparison, although the ITCs detection sensitivity of our model was slightly lower than this value, it generally remains above 70% across three datasets and four feature extractors. Notably, Paige BLN was developed exclusively for LNM of breast cancer, and its performance for ITCs detection in a multi-cancer setting still needs further evaluation. In summary, one of the challenges of CAMELYON17 is the detection of ITCs, as algorithms based on fully supervised learning have reported sensitivities below 40%. The MambaMIL+HiLA-MIL algorithm successfully overcame the challenges of the CAMELYON17^6^ and outperformed the VIS algorithm^36^.

To investigate the generalization ability of the MambaMIL + HiLA-MIL model in the multi-cancer LNM detection task, our study cohort enrolled five cancer types: gastric adenocarcinoma, colorectal adenocarcinoma, lung adenocarcinoma, breast invasive ductal carcinoma, and breast invasive lobular carcinoma. Across three centers and four feature extractors, the MambaMIL + HiLA-MIL model exhibited the most stable performance in multi-cancer detection based on comprehensive performance metrics, demonstrating excellent generalizability for LNM detection.

These results were consistent with the study by Pan et al.^22^, who developed two deep learning models (DeepLab v3 + ResNet-50) for LNM detection using pan-origin WSIs from the internal dataset. Their pan-cancer slide-level AUC, sensitivity, specificity, and accuracy reached 0.958, 0.952, 0.722, and 0.848, respectively, and the pan-cancer slide-level AUC achieved 0.925 in the external cohort. In the internal dataset of this study, the MambaMIL + HiLA-MIL model with the ResNet feature extractor achieved multi-cancer detection AUC, sensitivity, specificity, and accuracy of 0.9773 ± 0.0063, 0.6309 ± 0.0805, 0.9418 ± 0.0285, and 0.9047 ± 0.0200, respectively. The AUC values on the two external datasets were 0.9299 ± 0.0206 and 0.8949 ± 0.0187, respectively. The performance of our model is superior to that of Pan et al. Furthermore, the two deep learning models used in their study were based on fully supervised learning, which further highlights the significant advantages of the weakly supervised MambaMIL + HiLA-MIL model for LNM detection across multi-cancer settings. Another recent study presented the PanCAM model developed on ultra-large-scale multi-center and multi-cancer datasets. After extensive retrospective and prospective multi-center validation, the model attained a sensitivity near 1.00 and a consistently high AUC of around 0.99 for LNM detection^38^. Although the PanCAM model exhibits nearly perfect diagnostic performance in pan-cancer LNM detection, it relies on a fully supervised learning framework and requires extensive pixel-level fine annotations, resulting in high costs for clinical application. The MambaMIL + HiLA-MIL model proposed in this study exhibited its key advantage over the PanCAM model by adopting a weakly supervised framework that only required slide-level labels. It showed robust performance in multi-cancer LNM detection under multi-center and multi-feature extractor validation, while maintaining highly competitive diagnostic accuracy. Therefore, MambaMIL + HiLA-MIL offered greater advantages for real clinical practice.

This study performed a comprehensive comparative analysis of the overall performance of four feature extractors based on the multi-center datasets. The results revealed that for both overall four-class LNM classification and individual subtype classification tasks, the MambaMIL + HiLA-MIL model with UNI and GigaPath achieved significantly better performance than ResNet and Virchow. In terms of the UNI feature extractor, the experimental results of the present study are consistent with those reported by Chen et al.^30^. Chen et al. demonstrated that when UNI was adopted as the feature extractor and combined with ABMIL for binary LNM classification on the CAMELYON16 and CAMELYON17 datasets, it achieved the highest AUC and optimal cross-center generalization ability.

Specifically, the cross-center accuracy drop of UNI was only 5.1%, whereas that of ResNet-50 reached 15.0%. Our work performed multi-cancer LNM four-class classification across three datasets. The cross-center accuracy drop of UNI was 6.2%, which was comparable to the findings of Chen et al., while the corresponding decline of ResNet-50 was 10.8%. These findings confirmed that UNI exhibited strong cross-center stability. Built upon the UNI feature extractor, our model effectively alleviated performance fluctuations caused by dataset heterogeneity and demonstrated superior adaptability, generalization capability, and robustness.

In terms of the Virchow model, the findings of our research were inconsistent with those of another study^32^. This study combined the Virchow feature extractor with the Agata aggregator (a lightweight cross-attention MIL model) and performed binary LNM detection on the CAMELYON16 and CAMELYON17 datasets. The Virchow model performed excellently, comparable to the clinical dedicated breast lymph node model (Paige BLN), with significantly higher AUC than the UNI model and better performance in macroscopic metastasis detection. Our results show that compared with other foundational pathological models, Virchow only achieves marginal performance gains in the macro-metastasis task on the internal dataset, and exhibits statistical advantages solely in the ITCs task on external datasets. Overall, Virchow delivers limited overall performance improvement compared with UNI and GigaPath. The discrepancy in results may stem from the fact that the CAMELYON16/CAMELYON17 datasets are publicly available and consist of a single cancer type. In contrast, our study was based on multicenter and multi-cancer cohorts, which may be a key factor contributing to the inconsistent performance of the Virchow model.

Our study fills the gap of using GigaPath as a feature extractor in LNM four-class detection. GigaPath performs similarly to UNI, achieving optimal and robust overall performance that is significantly superior to ResNet and Virchow. With GigaPath feature extraction, MambaMIL + HiLA-MIL maintains stable and high diagnostic accuracy across multi-center datasets and multi-cancer cohorts, showing excellent cross-dataset stability in multi-cancer, colorectal cancer and breast cancer subtypes.

The following limitations to this study must be addressed. First, all internal and external datasets were retrospectively collected, lacking independent validation with prospective cohorts in real clinical pathological workflows. Second, although multiple cancer types were enrolled, rare pathological subtypes and low-incidence cancers had limited sample sizes; more data are needed to validate the model’s performance in detecting rare LNM subtypes. Third, while three-center datasets and four feature extractors confirmed the model’s architectural generalization and robustness, this study did not fully cover image heterogeneity from inter-hospital variations in slide preparation, staining protocols and scanning devices. Its cross-platform generalization in real clinical scenarios requires further validation.

In summary, we developed a weakly supervised WSI classification model multi-cancer LNM detection. Across multiple centers and feature extractors, our model achieves state-of-the-art performance in the overall four-class LNM classification task. It demonstrates good detection capability for ITCs and micro-metastasis, especially for ITCs. The model still demonstrated good stability in detecting LNM across multi-cancer and various cancer types. Moreover, UNI and GigaPath achieve markedly better comprehensive performance than ResNet and Virchow in the overall four-class classification of LNM. MambaMIL + HiLA-MIL, a multi-cancer model for four-class LNM classification, achieves compelling efficacy and robustness across multiple centers and feature extractors with prominent ITCs detection capability, offering a reliable tool for clinical LNM stratification.

## Methods

### Dataset description

#### Internal dataset

The NIMM dataset (662 lymph node histopathological sections) was collected from Cancer Hospital of Dalian University of Technology (Shenyang, China) from January 2018 to January 2024 and included 384 patients (135 males and 249 females; age range 26–86 years; median age 58 years [interquartile range 52–64]), including 141 gastric adenocarcinoma, 168 colorectal adenocarcinoma, 116 lung invasive adenocarcinoma, 156 breast invasive ductal carcinoma, 33 breast invasive lobular carcinoma, 36 endometrioid adenocarcinoma, and 12 thyroid papillary carcinoma sections. No sex- and gender-related analysis were performed in this study. All tissue sections were formalin-fixed, paraffin-embedded, and stained with hematoxylin and eosin (H&E). All slides were digitally scanned with a NanoZoomer 2.0-HT Brightfield Fluorescence Slide Scanning System (Hamamatsu Photonics, Hamamatsu, Japan) to construct a WSI dataset for lymph node pathology. Hamamatsu NDP.view2 (v2.9.25) is used for image viewing and screenshot export. Adobe Illustrator 2024 (v28.0) and BioRender are adopted for figure preparation. Classification of LNM was based on the presence or absence of metastasis, the number of cancer cells in the metastatic lymph nodes, and the size of the tumor foci as no metastasis/negative, ITCs (≤200 scattered tumor cells or tumor clusters ≤0.2 mm), micro-metastasis (>0.2–≤ 2 mm), or macro-metastasis (>2 mm)^8^. If multiple metastatic foci were present on a section, diagnosis was based on the largest metastatic focus. This internal dataset was named NIMM, based on the first letters of the four-class LNM categories (Negative, ITCs, Micro-metastasis, and Macro-metastasis). The NIMM dataset was reviewed by two pathologists with >10 years of diagnostic experience. In cases of disagreement, immunohistochemical methods were used to assist in diagnosis. The final accurate four-class labels comprised 231 cases classified as negative, 40 as ITCs, 82 as micro-metastasis, and 309 as macro-metastasis. The detailed LNM classification information for each cancer type in the NIMM dataset is displayed in Table 4. The study protocol was approved by the Ethics Committee of Cancer Hospital of Dalian University of Technology (approval number: KY20231115). The ethics committee granted a waiver of informed consent for the retrospective analysis of de-identified pathological WSIs.

**Table 4:** LNM classification across different carcinoma types on NIMM, DLZX, and YDSY datasets

NIMM								
LNM classification	Gastric adenocarcinoma	Colorectal adenocarcinoma	Lung adenocarcinoma	Breast invasive ductal carcinoma	Breast invasive lobular carcinoma	Papillary thyroid carcinoma	Endometrioid carcinoma of female reproductive tract	Total
Negative	53	73	47	23	0	3	32	231
ITC	1	8	2	21	0	5	3	40
Micro-metastasis	7	6	10	48	6	4	1	82
Macro-metastasis	80	81	57	64	27	0	0	309
Total	141	168	116	156	33	12	36	662

DLZX									
LNM classification	Gastric adenocarcinoma	Colorectal adenocarcinoma	Lung adenocarcinoma	Breast invasive ductal carcinoma	Breast invasive lobular carcinoma	Papillary thyroid carcinoma	Cervical adenocarcinoma	Submandibular duct adenocarcinoma	Total
Negative	16	68	0	0	0	12	0	0	96
ITC	1	2	0	14	0	2	2	0	21
Micro-metastasis	7	9	1	4	3	46	0	0	70
Macro-metastasis	21	71	1	79	17	25	0	1	215
Total	45	150	2	97	20	85	2	1	402

YDSY										
LNM classification	Gastric adenocarcinoma	Colorectal adenocarcinoma	Lung adenocarcinoma	Breast invasive ductal carcinoma	Breast invasive lobular carcinoma	Papillary thyroid carcinoma	Cervical adenocarcinoma	High-grade serous carcinoma of female genital tract	Biliary tract adenocarcinoma	Total
Negative	0	22	14	18	0	15	1	1	0	71
ITC	0	1	2	14	0	2	3	0	0	22
Micro-metastasis	6	12	6	3	3	28	0	0	0	58
Macro-metastasis	8	46	14	33	13	28	1	2	5	150
Total	14	81	36	68	16	73	5	3	5	301

#### External dataset

The DLZX dataset (402 sections) was collected from Dalian Central Hospital (Dalian, China) from January 2018 to January 2026 and included 241 patients (70 males and 171 females; age range 23–93 years; median age 60 years [interquartile range 48–68]), including 45 gastric adenocarcinoma, 150 colorectal adenocarcinoma, 2 lung invasive adenocarcinoma, 97 breast invasive ductal carcinoma, 20 breast invasive lobular carcinoma, 85 thyroid papillary carcinoma, 2 cervical adenocarcinoma, and 1 submandibular duct adenocarcinoma sections. The YDSY dataset (301 sections) was collected from the Fourth Affiliated Hospital of China Medical University (Shenyang, China) from January 2018 to January 2026 and included 283 patients (103 males and 180 females; age range 20–89 years; median age 60 years [interquartile range 49–68]), including 14 gastric adenocarcinoma, 81 colorectal adenocarcinoma, 36 lung invasive adenocarcinoma, 68 breast invasive ductal carcinoma, 16 breast invasive lobular carcinoma, 73 thyroid papillary carcinoma, 5 cervical adenocarcinoma, 3 high-grade serous carcinoma of female genital tract, and 5 biliary tract adenocarcinoma sections.

No sex- and gender-related analysis were performed in this study. The final accurate four-class labels are distributed as follows: on the DLZX dataset, there are 96 negative cases, 21 ITCs cases, 70 micro-metastasis cases, and 215 macro-metastasis cases; and on the YDSY dataset, 71 cases are negative, 22 are ITCs, 58 are micro-metastasis, and 150 are macro-metastasis. The detailed LNM classification information for each cancer type in the DLZX and YDSY datasets is also presented in Table 4. The study protocol was approved by the Ethics Committees of Dalian Central Hospital and the Fourth Affiliated Hospital of China Medical University (approval numbers: YN2025-118-01, EC-2024-KS-119). The ethics committees granted a waiver of informed consent for the retrospective analysis of de-identified pathological WSIs.

### WSI processing

In accordance with previous studies^23,25,26,28^, each WSI was divided into non-overlapping patches of 256 × 256 pixels at a magnification of 20×, while excluding background areas. Multiple pre-trained models were utilized to extract patch-level features, including a ResNet-50 backbone pre-trained on ImageNet^29^ and three histology-specific foundation models (UNI, Virchow, and Prov-GigaPath)^30–32^. The resulting feature vectors, which range from 1024 dimensions (for ResNet-50 and UNI) to 1536 and 2560 dimensions (for GigaPath and Virchow), were compressed to a fixed size of 512 dimensions using a linear bottleneck layer before being fed into the MIL framework.

Each of the three datasets was divided into ten folds using Stratified K-Fold cross-validation. By employing stratified sampling based on class labels, this K-Fold method maintains a consistent class distribution across all folds.

For NIMM, each fold contains 595 training samples and 67 testing samples. Specifically, the distribution of training samples for each category of each fold comprised 595 samples (208 negative, 36 ITCs, 73 micro-metastasis, and 278 macro-metastasis). The test set for each category included 67 samples (23 negative, 4 ITCs, 9 micro-metastasis, and 31 macro-metastasis).

For ZDSY, each fold contains 270 training samples and 31 testing samples. Specifically, the distribution of training samples for each category of each fold comprised 270 samples (64 negative, 19 ITCs, 52 micro-metastasis, and 135 macro-metastasis). The test set for each category included 31 samples (7 negative, 3 ITCs, 6 micro-metastasis, and 15 macro-metastasis).

For DLZX, each fold contains 361 training samples and 41 testing samples. Specifically, the distribution of training samples for each category of each fold comprised 361 samples (86 negative, 19 ITCs, 63 micro-metastasis, and 193 macro-metastasis). The test set for each category included 41 samples (10 negative, 2 ITCs, 7 micro-metastasis, and 22 macro-metastasis).

### HiLA-MIL model architecture

**Fig. 4:**
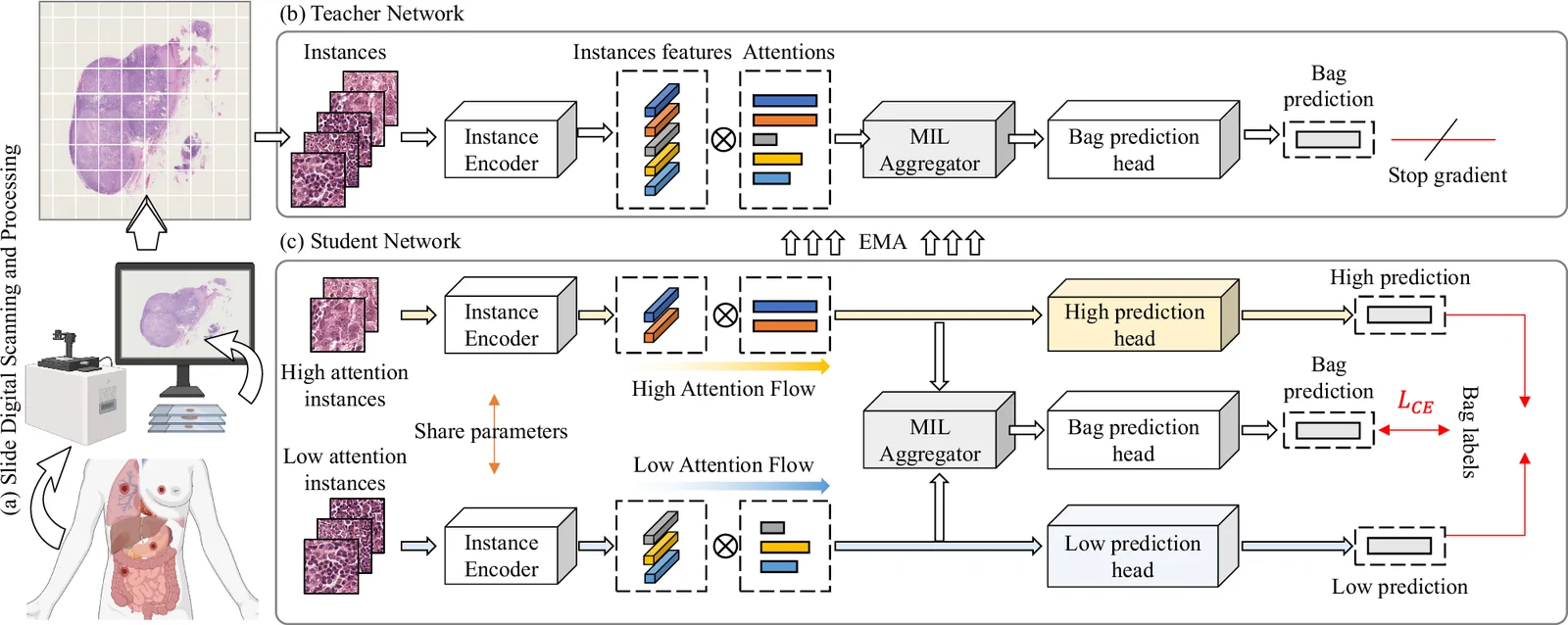
Schematic overview of the proposed HiLA-MIL framework. **a** Slide Digital Scanning and Processing. Whole slide images from multi-cancer cohorts are digitally scanned and segmented into independent instance patches. **b** Teacher Network. The unseparated instance patches are fed into the instance encoder. The extracted instance features are weighted by their corresponding attention scores and aggregated through the MIL aggregator to generate a holistic bag-level prediction. During the joint teacher-student training phase, the parameters of the teacher network are updated via EMA from the student network, and a stop-gradient barrier is applied during backpropagation. **c** Student Network. Based on the attention scores, instances are decoupled into high-attention and low-attention subsets, processing via two parallel pathways (High and Low Attention Flows) with shared encoder parameters. The separated streams feed into customized prediction heads (High, Low, and standard Bag prediction heads). The entire architecture is optimized jointly by minimizing the bag-level cross-entropy loss and the separation enhancement loss to accurately refine positive and negative sample mining. In this figure (**a**) was created with BioRender.com.

Here, we propose a simple and efficient MIL framework, called HiLA-MIL, to improve classification of WSIs. A separation learning strategy was designed based on a Siamese architecture. The framework introduces a collaboration between a student model and a teacher model, with high-low attention mechanisms to enhance feature extraction and the ability to discriminate local regions (such as tumor cells, immune cells, etc.). The overall framework is shown in Fig. 4, where the student model is used to aggregate instance features and make predictions, while the teacher model guides the student model by providing attention weights and generating positive-negative sample masks.

#### MIL

In the MIL framework, each high-resolution WSI is considered a bag, consisting of multiple non-overlapping patches, known as instances. Given a WSI$\,X\,$, the image is divided into a set of patches, forming an instance collection$\,X=\{{x}_{1},{x}_{2},\ldots,{x}_{N}\}$, whereN denotes the number of patches within the WSI. This collection of patches forms a single bag, with each patch ${x}_{i}$ acting as an instance in that bag. The goal of the MIL approach is to classify the entire bag based on the bag-level label Y, thereby achieving slide-level classification. In MIL, only the bag-level label is available, and no specific labels are provided for individual instances (patches). This characteristic leads to a two-step MIL process:

(1) Instance Encoder: Each instance ${x}_{i}$ is processed through an encoder$\,{{\rm{h}}}$(⋅*;***θ**_1_), which generates a latent feature representation ${{{\bf{z}}}}_{i}$ as follows:

1 $${{{\bf{z}}}}_{i}={{\rm{h}}}\left({x}_{i};{{{\boldsymbol{\theta }}}}_{1}\right)$$

(2) MIL Aggregator: The set of instance features ${{\bf{Z}}}=\{{{{\bf{z}}}}_{1},{{{\bf{z}}}}_{2},\ldots,{{{\bf{z}}}}_{N}\}$ is aggregated through an aggregator $g\left(\cdot ;{{{\boldsymbol{\theta }}}}_{2}\right)$ to produce the bag-level prediction $\hat{Y}$:

2 $$\hat{Y}={{\rm{\sigma }}}\left(g\left({{\bf{Z}}};{{{\boldsymbol{\theta }}}}_{2}\right)\right)$$

where ${{\rm{\sigma }}}\left(\cdot \right)$ is the Sigmoid function. Through this process, MIL effectively consolidates local information to enhance prediction accuracy at the bag (WSI) level.

#### Instance encoder—MambaMIL

For this task, we adopted the Vision Mamba framework, which efficiently models long-range dependencies and complex tissue interactions. Vision Mamba excels at modeling spatial relationships, outperforming or matching Transformer-based models for visual tasks, as shown in Supplementary Fig. 7. In contrast to Transformer-based methods, such as TransMIL, and CNN-based methods, such as AB-MIL, we introduce MambaMIL, which obtains instance representations ${{{\bf{z}}}}_{i}$ by combining pre-trained ResNet50 (UNI, GigaPath and Virchow) and the Vision Mamba module as the Instance Encoder.

#### Attention mechanism

To adaptively aggregate instance features into a compact slide-level representation, we employ an attention-based instance weighting module that adaptively assigns importance scores to each instance based on its diagnostic relevance. An architecture diagram illustrating the full pipeline is shown in Supplementary Fig. 8. The representations ${{{\bf{z}}}}_{i}$ are first projected to raw attention logits ${\widetilde{a}}_{i}$ via a three-layer fully connected network. This network serves as a learnable attention head, allowing the model to capture non-linear associations between instance features and their potential diagnostic value.

These attention logits ${\widetilde{a}}_{i}$ are then normalized using the softmax function to produce attention scores ${a}_{i}\in \left[0,1\right]$, ensuring that the contributions of all N instances are properly normalized:

3 $${a}_{i}=\frac{\exp \left({\widetilde{a}}_{i}\right)}{{\Sigma }_{j=1}^{N}\exp \left({\widetilde{a}}_{j}\right)}$$

These normalized attention scores ${a}_{i}$ are used to reweight the original instance features ${{{\bf{z}}}}_{i}$ and a weighted sum is performed to generate a unified slide-level representation:

4 $${{\bf{h}}}=\sum _{i=1}^{N}{a}_{i}{{{\bf{z}}}}_{i}$$

#### MIL aggregator

Finally, the unified slide-level representation is fed into a fully connected classification layer followed by a softmax activation to produce the final slide-level prediction:

5 $$\hat{Y}={{\rm{softmax}}}({{\bf{h}}})$$

#### Attention separation

To enhance the discriminative ability of the student model to separate positive and negative samples, a separation learning mechanism was introduced. In the student network, the positive and negative branches share the same network parameters. By optimizing the separation loss function, the model increases the distance between positive and negative instances to further improve the distinguishing ability. Specifically, the separation loss function is defined as:

6 $${L}_{{{\rm{SE}}}}=\sum _{i}-{{\rm{softmax}}}\left(\frac{{{{\bf{h}}}}_{i}^{H}}{{T}_{{{\rm{temp}}}}}\right)\cdot \log \left({{\rm{softmax}}}\left(\frac{{{{\bf{h}}}}_{i}^{L}}{{S}_{{{\rm{temp}}}}}\right)\right)$$

where ${{{\bf{h}}}}_{i}^{H}$ denotes the feature output from the high attention flow, ${{{\bf{h}}}}_{i}^{L}$ is the feature output from the low attention flow, and ${T}_{{{\rm{temp}}}}=1$ and ${S}_{{{\rm{temp}}}}=1$ are temperature parameters controlling the softmax scaling. This loss function maximizes the similarity between positive samples and minimizes the similarity between negative samples, effectively separating them in the feature space. Through optimization of separation loss, the model progressively learns more discriminative feature representations.

#### Stochastic gradient descent and model optimization

Since the number of instances in each bag can vary and the feature distributions of instances often differ, Stochastic Gradient Descent with a batch size of 1 was used for model optimization. Although potentially less efficient computationally, this optimization strategy ensures that each gradient update is based on a single sample, which allows the model to make optimal adjustments for each individual instance, and helps to improve the model’s adaptability to WSIs with varying instance numbers and feature distributions, while maintaining strong generalization ability.

#### Student optimization

The final loss function consists of two components: separation loss and a class-weighted bag-level classification loss. The overall loss function is given by:

7 $$L={L}_{{{\rm{SE}}}}+{L}_{{{\rm{CE}}}}$$

where ${L}_{{{\rm{SE}}}}$ is the separation loss defined as above and ${L}_{{{\rm{CE}}}}$ is the bag-level classification loss, implemented as a class-weighted cross-entropy loss to mitigate the bias toward majority classes caused by imbalanced sample distribution. The formulation is:

8 $${L}_{{{\rm{CE}}}}=-\sum _{c=1}^{C}{w}_{c}{y}_{c}\log \left({\hat{y}}_{c}\right)$$

where ${y}_{c}\in \{0,1\}$ is the one-hot true bag-level label, ${\hat{y}}_{c}$ is the predicted probability for class c, and ${w}_{c}=\frac{N}{C\cdot {N}_{c}}$ is the inverse class frequency weight assigned to class c, ${N}_{c}$ denotes the number of samples in class c, C is the total number of classes, and $N={\sum }_{c=1}^{C}{N}_{c}$ is the total number of training samples across all classes. This weighting scheme assigns higher weights to minority classes and lower weights to majority classes.

#### Teacher optimization

The parameters of teacher ${{{\boldsymbol{\theta }}}}_{t}$ are updated by an exponential moving average (EMA) of the student parameters ${{{\boldsymbol{\theta }}}}_{s}$. The updated rule is ${{{\boldsymbol{\theta }}}}_{t}\leftarrow \lambda {{{\boldsymbol{\theta }}}}_{t}+\left(1-\lambda \right){{{\boldsymbol{\theta }}}}_{s}$, where λ is a hyperparameter.

#### Inference pipeline

During inference, HiLA-MIL relies solely on the trained student model and performs prediction in a fully end-to-end manner, without introducing the teacher model or applying the high-low-attention instance grouping used in the training stage.

It is important to note that the high-/low-attention grouping strategy is employed only during training as an auxiliary supervision mechanism, where contrastive learning is used to guide the student model to distinguish informative high-attention instances from less relevant low-attention ones. Through this process, the student model progressively learns a discriminative parameters, including the instance encoder, the MIL aggregation module, and the two-layer MLP used for generating attention weights. As a result, at inference time, the model is already capable of effectively differentiating instances without requiring explicit high-/low-attention grouping. Instead, all instances are directly utilized for feature modeling and attention-based aggregation, which not only preserves the completeness of information but also leads to a more streamlined inference pipeline.

Given a test whole slide image X, we first partition it into a set of non-overlapping patches, forming a bag of instances $X=\{{x}_{1},{x}_{2},\ldots,{x}_{N}\}$, where N denotes the total number of patches, and each patch ${x}_{i}$ corresponds to an instance in the bag. All patches are then passed through a pretrained backbone network (e.g., ResNet50, UNI, Gigapath, or Virchow) to extract feature embeddings. The extracted features are fed into the trained student model with all parameters fixed. Unlike the training stage, no instance grouping is performed; instead, all instance features are directly processed by the instance encoder to obtain$\,{{\bf{Z}}}=\{{{{\bf{z}}}}_{1},{{{\bf{z}}}}_{2},\ldots,{{{\bf{z}}}}_{N}\}$.

Each instance feature ${{{\bf{z}}}}_{i}$ is passed through a two-layer multilayer perceptron (MLP) to produce a raw attention logit ${\widetilde{a}}_{i}$. These logits are normalized via a softmax function to obtain attention weights:

9 $${a}_{i}=\frac{\exp \left({\widetilde{a}}_{i}\right)}{{\Sigma }_{j=1}^{N}\exp \left({\widetilde{a}}_{j}\right)},{a}_{i}\in \left[0,1\right]$$

The bag-level representation is then computed as a weighted sum of instance features:

10 $${{\bf{h}}}=\sum _{i=1}^{N}{a}_{i}{{{\bf{z}}}}_{i}$$

Finally, the aggregated representation ${{\bf{h}}}$ is fed into a fully connected layer followed by a softmax function to obtain the bag-level prediction$\,\hat{{{\rm{Y}}}}={{\rm{softmax}}}({{\bf{h}}}).$

### Implementation details

The model was trained for 200 max epochs using the Adam optimizer (learning rate: $2\times {10}^{-4}$, weight decay:$1\times {10}^{-5}$), with early stopping (patience: 30). The EMA momentum rate λ was set to 0.9999, while and prototype loss temperature was set to 0.1. All experiments were conducted with a NVIDIA GeForce RTX 3090 (24GB) graphics processing unit (NVIDIA Corporation, Santa Clara, CA, USA). The software stack was built with CUDA 11.8 and PyTorch 2.1.1, and all model training was performed with full 32-bit floating-point precision. To systematically evaluate the computational efficiency of the proposed HiLA-MIL framework, we performed inference speed tests under three standard input configurations, which correspond to the feature dimensions of three mainstream pathological encoders. For each test, the input was a feature tensor of a single whole slide image, containing 4096 patch-level features extracted from the corresponding encoder. Specifically, for the 1024-dimensional feature input (compatible with UNI and ResNet 50 encoders), the model achieved an average inference time of 3.68 ms with a compact model size of 25.94 MB. For the 1536-dimensional input adapted to the GigaPath encoder, the framework completed WSI-level inference within 7.41 ms, with a model size of 57.66 MB. For the 2560-dimensional input supporting the Virchow encoder, the average inference time was 19.42 ms, and the corresponding model size was 158.59 MB.

### Evaluation metrics

AUC evaluates a model’s ability to distinguish between classes, derived from the ROC curve. AUC values range from 0 to 1, with higher values indicating better performance. An AUC of 0.5 suggests random guessing, while 1.0 indicates perfect discrimination. Accuracy measures the proportion of correctly predicted instances in a dataset, calculated as $\frac{{{\rm{TP}}}+{{\rm{TN}}}}{{{\rm{TP}}}+{{\rm{TN}}}+{{\rm{FP}}}+{{\rm{FN}}}}$, where ${{\rm{TP}}}$ represents true positives, ${\mbox{TN}}$ denotes true negatives, ${{\rm{FP}}}$ indicates false positives, and ${{\rm{FN}}}$ refers to false negatives. Precision focuses on the accuracy of positive predictions, calculated as $\frac{{{\rm{TP}}}}{{{\rm{TP}}}+{{\rm{FP}}}}$, which is crucial in scenarios where false positives carry a high cost, such as in medical diagnoses. High precision reflects effective identification of relevant instances. Recall, or sensitivity, measures the ability to identify all relevant instances, defined as $\frac{{{\rm{TP}}}}{{{\rm{TP}}}+{{\rm{FN}}}}$. High recall is vital in situations where missing a positive instance could have serious consequences, such as disease screening. Specificity measures the ability to correctly identify all instances that are actually negative, defined as $\frac{{{\rm{TN}}}}{{{\rm{TN}}}+{{\rm{FP}}}}$. High specificity indicates high reliability for identification of negative categories, while reducing the occurrence of false positives. High specificity indicates reliable identification of false negatives with fewer false positives. The F1-score combines precision and recall into a single value, calculated as $2\times \frac{{{\rm{Precision}}}\times {{\rm{Recall}}}}{{{\rm{Precision}}}+{{\rm{Recall}}}}$, which is especially useful for imbalanced datasets by balancing the trade-off between false positives and false negatives, with a higher score indicating better model performance.

### Statistical analysis

The experiments were conducted using the NIMM dataset, DLZX dataset, YDSY dataset and two public datasets, CAMELYON17 and CAMELYON16. We compared the performance of the MambaMIL + HiLA-MIL model with six other weakly supervised deep learning algorithms (AB-MIL, CLAM-SB, CLAM-MB, DSMIL, TransMIL, and MambaMIL) across four feature extractors (ResNet, UNI, Virchow, and GigaPath). The average values and standard deviations of each evaluation metric were reported over tenfold cross-validation for overall four-class and individual classification of LNM, and over threefold cross-validation for overall four-class classification across multiple and single cancer types. Stratified k-fold cross-validation was performed using the StratifiedKFold function in Scikit-learn (v.1.3.2) with Python (v.3.10). All evaluation metrics were computed using Metrics function in Scikit-learn (v.1.3.2) with Python (v.3.10). To test for statistical significance, a two-sided paired permutation test was used, with 10,000 permutations to evaluate performance differences between models. All statistical analysis were performed in Python (v.3.10). A *P*-value < 0.05 was considered statistically significant.

### Model visualization

To facilitate interpretability of the model and enhance the transparency of decision-making, an attention-based WSI visualization method was adopted, which utilizes attention weights extracted from a trained model to generate heatmaps that highlight regions of diagnostic importance, enabling intuitive understanding of the model’s focus areas. During preprocessing, WSIs are divided into fixed-size patches, and corresponding patch-level features are extracted and saved in the.h5 format. Simultaneously, spatial coordinates for each patch were obtained by alignment with the corresponding thumbnail image. These coordinates serve as a reference to project attention scores back to the spatial layout of the WSI. In the visualization phase, a pretrained classification model is loaded to infer the attention scores for each patch. These attention weights reflect the relative contribution of each patch to the final prediction ability of the model. The scores are then mapped back to the original coordinate space to construct a two-dimensional attention mask aligned with the thumbnail resolution that encodes the significance of each region within the WSI. To generate the final visualization, the attention mask is first upsampled to match the size of the original thumbnail image using bilinear interpolation. Then, a colormap (e.g., COLORMAP_JET) was applied to convert the normalized attention scores into a heatmap, which is blended with the thumbnail image to produce a Class Activation Map. All heatmap values are normalized to the range of 0–1 with no fixed absolute physical extrema. Warmer colors in the heatmap correspond to regions with higher model attention (values approaching 1), while cooler colors represent regions with lower attention (values approaching 0). This fusion allows for intuitive visual interpretation of the WSI and provides insights into the spatial distribution of informative regions driving the model’s decision.

### Reporting summary

Further information on research design is available in the Nature Portfolio Reporting Summary linked to this article.

## Supplementary information

Supplementary Information

Reporting Summary

Transparent Peer Review file

## Source data

Source Data

## Data Availability

To protect patient privacy, raw whole-slide images (WSIs) data are not publicly available. Researchers requiring access to the raw data may contact the corresponding author, Yanmei Zhu. Remote access to WSIs data via Virtual Private Network will only be granted upon approval from the Institutional Review Boards of Cancer Hospital of Dalian University of Technology, Dalian Central Hospital, and the Fourth Affiliated Hospital of China Medical University. All data access requests will be processed within 20 working days. Source data are provided with this paper.

## Code availability

The full source code for implementing the HiLA-MIL framework is publicly available in the GitHub repository (https://github.com/YAOSL98/HiLA-MIL) under the MIT License and archived on Zenodo (DOI: 10.5281/zenodo.21028097)^39^. The slide preprocessing modules in our codebase are adapted from the open-source CLAM framework (https://github.com/mahmoodlab/CLAM), and the original license and copyright statements have been preserved in compliance with its MIT License.

## References

[1] Amin, M. B. et al. *AJCC Cancer Staging System, Version 9* (American Joint Committee on Cancer, 2023).

[2] Liikanen, J. S., Leidenius, M. H., Joensuu, H., Vironen, J. H. & Meretoja, T. J. Prognostic value of isolated tumour cells in sentinel lymph nodes in early-stage breast cancer: a prospective study. *Br. J. Cancer* **118**, 1529–35. (2018). 29686324 10.1038/s41416-018-0052-7 PMC5988733

[3] Huang, S. C. et al. Deep neural network trained on gigapixel images improves lymph node metastasis detection in clinical settings. *Nat. Commun.* **13**, 3347 (2022). 35688834 10.1038/s41467-022-30746-1 PMC9187676

[4] van Diest, P. J., van Deurzen, C. H. & Cserni, G. Pathology issues related to SN procedures and increased detection of micrometastases and isolated tumor cells. *Breast Dis.* **31**, 65–81 (2010). 21368369 10.3233/BD-2010-0298

[5] Ehteshami Bejnordi, B. et al. Diagnostic assessment of deep learning algorithms for detection of lymph node metastases in women with breast cancer. *JAMA* **318**, 2199–2210 (2017). 29234806 10.1001/jama.2017.14585 PMC5820737

[6] Bandi, P. et al. From detection of individual metastases to classification of lymph node status at the patient level: the CAMELYON17 challenge. *IEEE Trans. Med. Imaging* **38**, 550–60. (2019). 30716025 10.1109/TMI.2018.2867350

[7] Liu, Y. et al. Artificial intelligence-based breast cancer nodal metastasis detection: insights into the black box for pathologists. *Arch. Pathol. Lab. Med.* **143**, 859–68. (2019). 30295070 10.5858/arpa.2018-0147-OA

[8] Steiner, D. F. et al. Impact of deep learning assistance on the histopathologic review of lymph nodes for metastatic breast cancer. *Am. J. Surg. Pathol.* **42**, 1636–46. (2018). 30312179 10.1097/PAS.0000000000001151 PMC6257102

[9] Khalil, M. A., Lee, Y. C., Lien, H. C., Jeng, Y. M. & Wang, C. W. Fast segmentation of metastatic foci in H&E whole-slide images for breast cancer diagnosis. *Diagnostics* **12**, 990 (2022). 35454038 10.3390/diagnostics12040990 PMC9030573

[10] Pham, H. H. N. et al. Detection of lung cancer lymph node metastases from whole-slide histopathologic images using a two-step deep learning approach. *Am. J. Pathol.* **189**, 2428–39. (2019). 31541645 10.1016/j.ajpath.2019.08.014

[11] Pan, Y. et al. Automatic detection of squamous cell carcinoma metastasis in esophageal lymph nodes using semantic segmentation. *Clin. Transl. Med.* **10**, e129 (2020). 32722861 10.1002/ctm2.129 PMC7418811

[12] Tang, H. et al. Diagnosis of lymph node metastasis in head and neck squamous cell carcinoma using deep learning. *Laryngoscope Investig. Otolaryngol.* **7**, 161–169 (2022). 35155794 10.1002/lio2.742 PMC8823170

[13] Hu, Y. et al. Deep learning system for lymph node quantification and metastatic cancer identification from whole-slide pathology images. *Gastric Cancer* **24**, 868–77. (2021). 33484355 10.1007/s10120-021-01158-9

[14] Wang, X. et al. Predicting gastric cancer outcome from resected lymph node histopathology images using deep learning. *Nat. Commun.* **12**, 1637 (2021). 33712598 10.1038/s41467-021-21674-7 PMC7954798

[15] Matsushima, J. et al. The use of deep learning-based computer diagnostic algorithm for detection of lymph node metastases of gastric adenocarcinoma. *Int. J. Surg. Pathol.* **31**, 975–81. (2023). 35898183 10.1177/10668969221113475

[16] Khan, A. et al. Computer-assisted diagnosis of lymph node metastases in colorectal cancers using transfer learning with an ensemble model. *Mod. Pathol.* **36**, 100118 (2023). 36805793 10.1016/j.modpat.2023.100118

[17] Bándi, P. et al. Continual learning strategies for cancer-independent detection of lymph node metastases. *Med. Image Anal.* **85**, 102755 (2023). 36724605 10.1016/j.media.2023.102755

[18] Kindler, C., Elfwing, S., Öhrvik, J., Nikberg, M. & Deep, A. Neural network-based decision support tool for the detection of lymph node metastases in colorectal cancer specimens. *Mod. Pathol.* **36**, 100015 (2023). 36853787 10.1016/j.modpat.2022.100015

[19] Giammanco, A. et al. Fast-track development and multi-institutional clinical validation of an artificial intelligence algorithm for detection of lymph node metastasis in colorectal cancer. *Mod. Pathol.* **37**, 100496 (2024). 38636778 10.1016/j.modpat.2024.100496

[20] Wu, S. et al. Artificial intelligence-based model for lymph node metastases detection on whole slide images in bladder cancer: a retrospective, multicentre, diagnostic study. *Lancet Oncol.* **24**, 360–370 (2023). 36893772 10.1016/S1470-2045(23)00061-X

[21] Wu, S. et al. An artificial intelligence model for detecting pathological lymph node metastasis in prostate cancer using whole slide images: a retrospective, multicentre, diagnostic study. *EClinicalMedicine* **71**, 102580 (2024). 38618206 10.1016/j.eclinm.2024.102580 PMC11015342

[22] Pan, Y. et al. Clinically applicable pan-origin cancer detection for lymph nodes via artificial intelligence-based pathology. *Pathobiology* **91**, 345–358 (2024). 38718783 10.1159/000539010

[23] Ilse, M., Tomczak, J. M. & Welling, M. Attention-based deep multiple instance learning. In* Proceedings of the 35th**International Conference on Machine Learning* **80**, 2132–2141 (PMLR, 2018).

[24] Campanella, G. et al. Clinical-grade computational pathology using weakly supervised deep learning on whole slide images. *Nat. Med.* **25**, 1301–1309 (2019). 31308507 10.1038/s41591-019-0508-1 PMC7418463

[25] Lu, M. Y. et al. Data-efficient and weakly supervised computational pathology on whole-slide images. *Nat. Biomed. Eng.* **5**, 555–570 (2021). 33649564 10.1038/s41551-020-00682-w PMC8711640

[26] Li, B., Li, Y. & Eliceiri, K. W. Dual-stream multiple instance learning network for whole slide image classification with self-supervised contrastive learning. In *Proceedins of the IEEE/CVF Conference on Computer Vision and Pattern Recognition Workshops* 14318–14328 (IEEE, 2021). 10.1109/CVPR46437.2021.01409 PMC8765709 35047230

[27] Tan, L. et al. Colorectal cancer lymph node metastasis prediction with weakly supervised transformer-based multi-instance learning. *Med. Biol. Eng. Comput.* **61**, 1565–1580 (2023). 36809427 10.1007/s11517-023-02799-x PMC10182132

[28] Shao, Z. et al. TransMIL: transformer based correlated multiple instance learning for whole slide image classification. In *Adv. Neural Inf. Process. Syst*. **34**, 20908–20921 (PMLR, 2021).

[29] He, K., Zhang, X., Ren, S. & Sun, J. Deep residual learning for image recognition. In *Proceedings of the IEEE Conference on Computer Vision and Pattern Recognition* 770–778 (IEEE, 2016).

[30] Chen, R. J. et al. Towards a general-purpose foundation model for computational pathology. *Nat. Med.* **30**, 850–862 (2024). 38504018 10.1038/s41591-024-02857-3 PMC11403354

[31] Xu, H. et al. A whole-slide foundation model for digital pathology from real-world data. *Nature* **630**, 181–188 (2024). 38778098 10.1038/s41586-024-07441-w PMC11153137

[32] Vorontsov, E. et al. A foundation model for clinical-grade computational pathology and rare cancers detection. *Nat. Med.* **30**, 2924–2935 (2024). 39039250 10.1038/s41591-024-03141-0 PMC11485232

[33] Zhu, L. et al. Vision Mamba: efficient visual representation learning with bidirectional state space model. In *Proceedings of the 41st International Conference on Machine Learning* **235**, 62429–62442 (PMLR, 2024).

[34] Chuang, W. Y. et al. Identification of nodal micrometastasis in colorectal cancer using deep learning on annotation-free whole-slide images. *Mod. Pathol.* **34**, 1901–1911 (2021). 34103664 10.1038/s41379-021-00838-2 PMC8443445

[35] Challa, B. et al. Artificial intelligence-aided diagnosis of breast cancer lymph node metastasis on histologic slides in a digital workflow. *Mod. Pathol.* **36**, 100216 (2023). 37178923 10.1016/j.modpat.2023.100216

[36] van Dooijeweert, C. et al. Clinical implementation of artificial-intelligence-assisted detection of breast cancer metastases in sentinel lymph nodes: the CONFIDENT-B single-center, non-randomized clinical trial. *Nat. Cancer* **5**, 1195–1205 (2024). 38937624 10.1038/s43018-024-00788-z PMC11358151

[37] Retamero, J. A. et al. Artificial intelligence helps pathologists increase diagnostic accuracy and efficiency in the detection of breast cancer lymph node metastases. *Am. J. Surg. Pathol.* **48**, 846–854 (2024). 38809272 10.1097/PAS.0000000000002248 PMC11191045

[38] Wu, S. et al. Artificial intelligence-based pathological model for pan-cancer lymph node metastasis detection: a multicentre diagnostic study with retrospective and prospective validation. *Lancet Digit Health* **8**, 100961 (2026). 41792018 10.1016/j.landig.2025.100961

[39] Sun, L., Yao, S., Jia, Q. & Zhu, Y. Weakly supervised artificial intelligence for multi-cancer detection of lymph node metastasis on whole slide images. *GitHub Repos. HiLA-MIL*10.5281/zenodo21028097 (2026).42733055

