# Supplementary material for: Weakly supervised artificial intelligence for multi-cancer detection of lymph node metastasis on whole slide images
Source: Nat Commun. 2026 Aug 14;17:9770. doi: 10.1038/s41467-026-76410-w (PMC13572452; doi:10.1038/s41467-026-76410-w)
Supplement: Supplementary file 1 [file 41467_2026_76410_MOESM1_ESM.pdf]

Weakly Supervised Artificial Intelligence for Multi-cancer Detection of Lymph Node Metastasis on Whole Slide Images

Supplementary Information

Content

Supplementary Fig.1 ROC analysis for four-class LNM classification on the DLZX dataset. 2

Supplementary Fig.2 ROC analysis for four-class LNM classification on the YDSY dataset. 3

Supplementary Fig.3 ROC analysis of multi-cancer LNM classification using different feature extractors on the NIMM dataset.. 4

Supplementary Fig. 4 ROC analysis of multi-cancer LNM classification using different feature extractors on the DLZX dataset. 5

Supplementary Fig. 5 ROC analysis of multi-cancer LNM classification using different feature extractors on the YDSY dataset. 6

Supplementary Fig. 6 Visual retrospective analysis of multimodal WSIs using multiple MIL models. 7

Supplementary Fig. 7 Architectural pipeline of the MambaMIL framework. 8

Supplementary Fig. 8 The architecture diagram illustrating the full pipeline of the attention mechanism. 9

Supplementary Table 1 Evaluation metrics of overall four-class classification and each LNM category across four feature extractors on DLZX dataset. 10

Supplementary Table 2. Evaluation metrics of overall four-class classification and each LNM category across four feature extractors on YDSY Dataset. 17

Supplementary Table 3. Evaluation metrics of multi-cancer and individual cancer types on NIMM, DLZX, and YDSY Datasets under ResNet. 24

Supplementary Table 4. Evaluation metrics of multi-cancer and individual cancer types on NIMM, DLZX, and YDSY Datasets under UNI. 30

Supplementary Table 5. Evaluation metrics of multi-cancer and individual cancer types on NIMM, DLZX, and YDSY Datasets under Virchow. 36

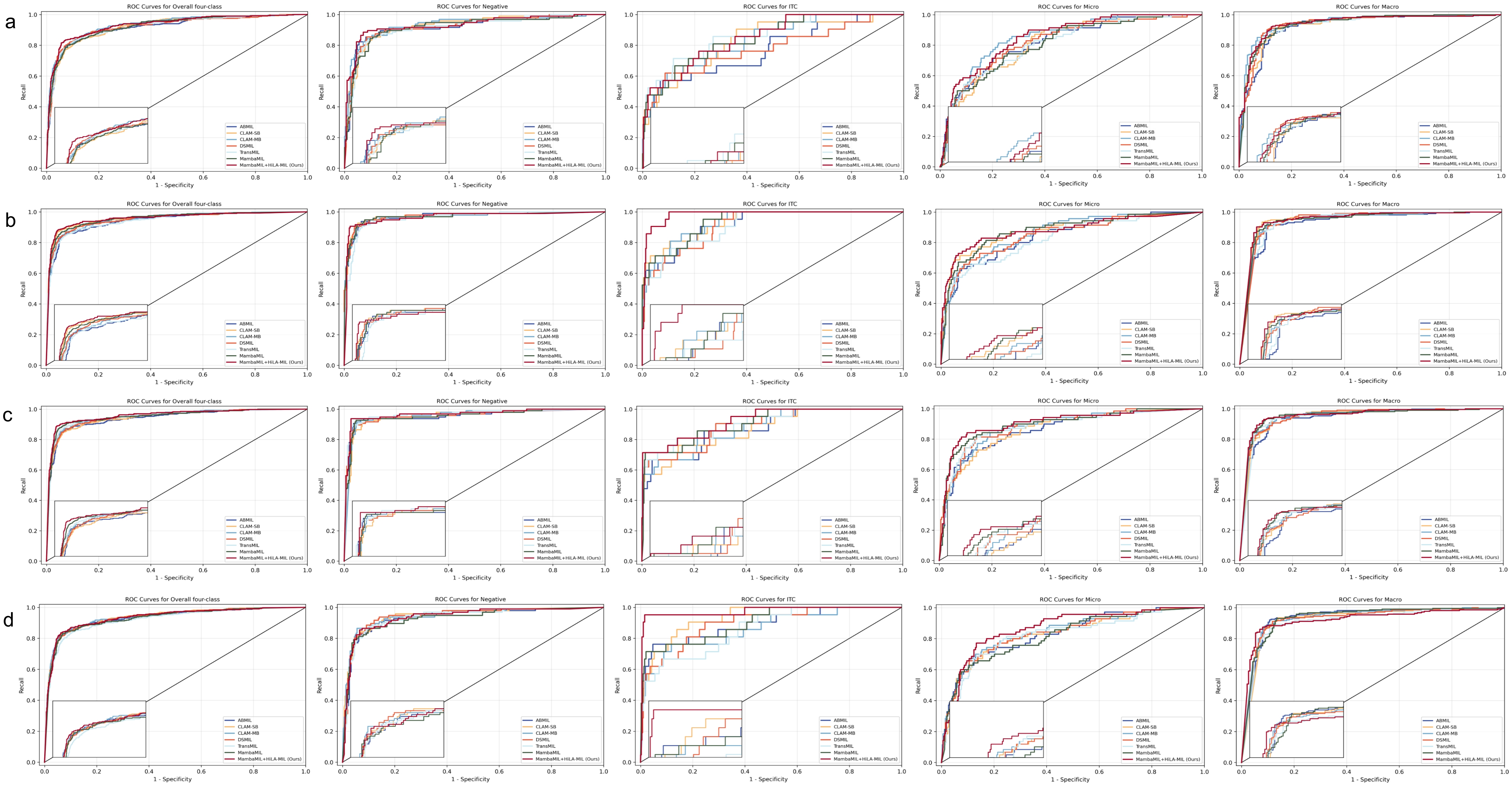

Supplementary Fig. 1 ROC analysis for four-class LNM classification on the DLZX dataset. ROC plots of the six baseline models as well as the MambaMIL+HiLA-MIL model for overall four-class classification and individual LNM categories across the (a) ResNet, (b) UNI, (c) GigaPath, and (d) Virchow feature extractors on the DLZX dataset. Source data are provided as a Source Data file.

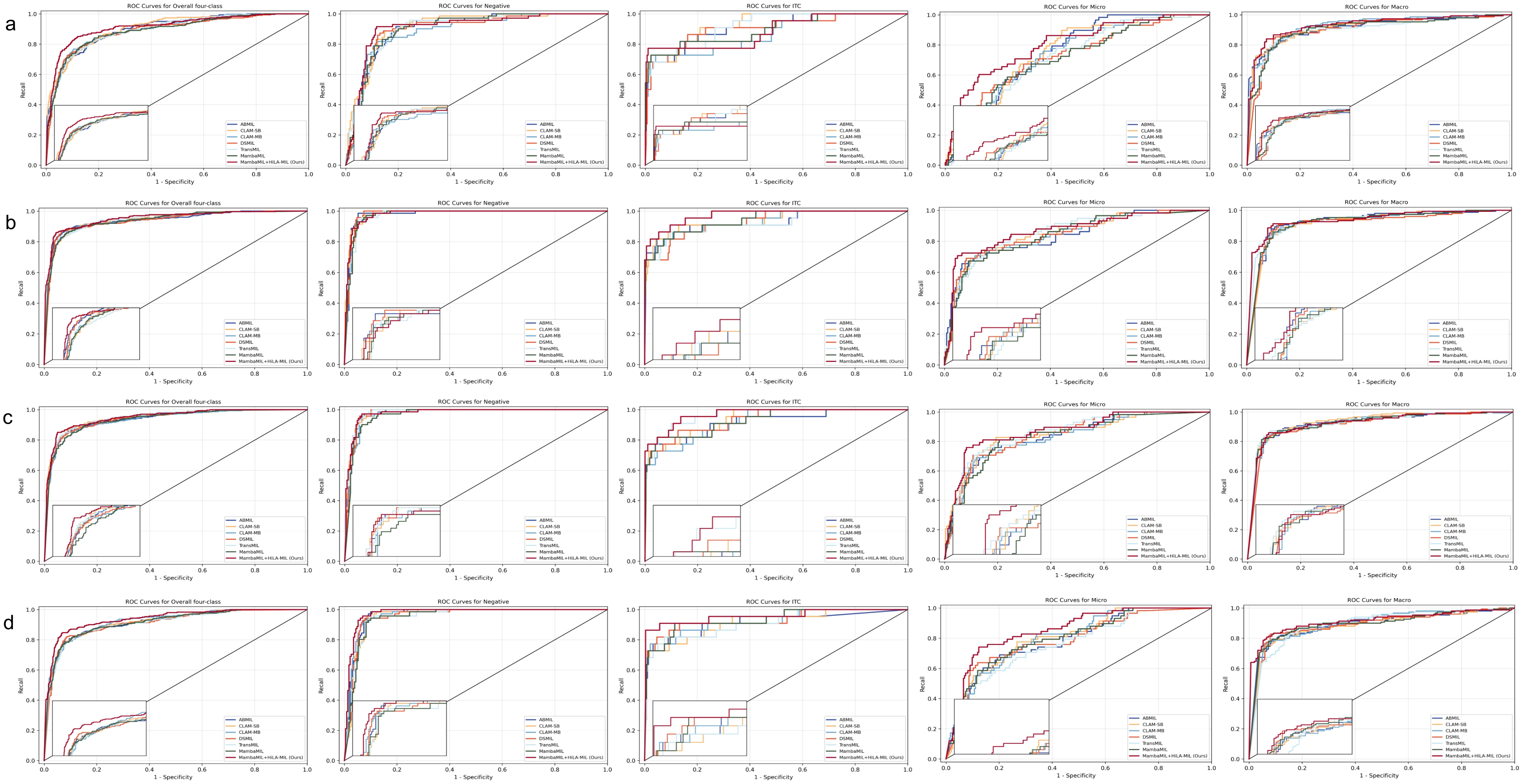

Supplementary Fig. 2 ROC analysis for four-class LNM classification on the YDSY dataset. ROC plots of the six baseline models as well as the MambaMIL+HiLA-MIL model for overall four-class classification and individual LNM categories across the (a) ResNet, (b) UNI, (c) GigaPath, and (d) Virchow feature extractors on the YDSY dataset. Source data are provided as a Source Data file.

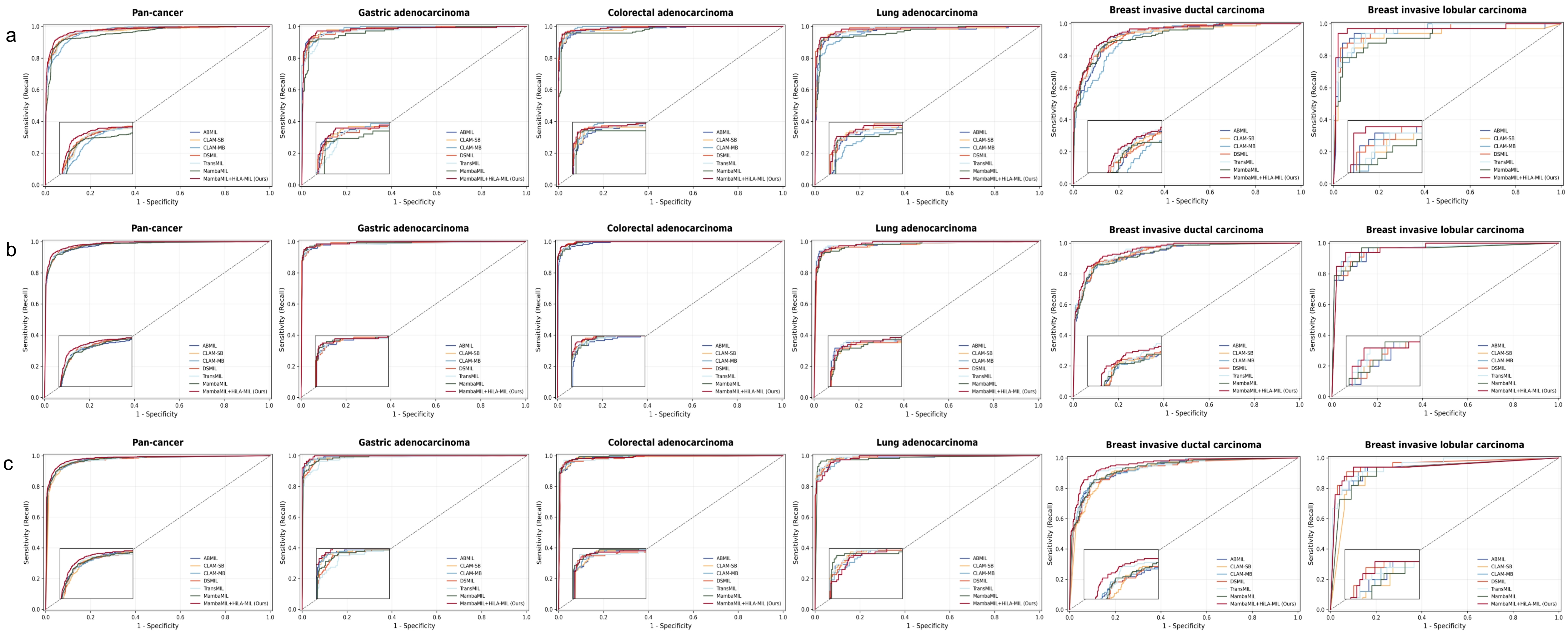

Supplementary Fig. 3 ROC analysis of multi-cancer LNM classification using different feature extractors on the NIMM dataset. ROC plots of the six baseline models as well as the MambaMIL+HiLA-MIL model for multi-cancer, gastric adenocarcinoma, colorectal adenocarcinoma, lung adenocarcinoma, breast invasive ductal carcinoma, and breast invasive lobular carcinoma across with the (a) ResNet, (b) UNI, and (c) Virchow on the NIMM dataset. Source data are provided as a Source Data file.

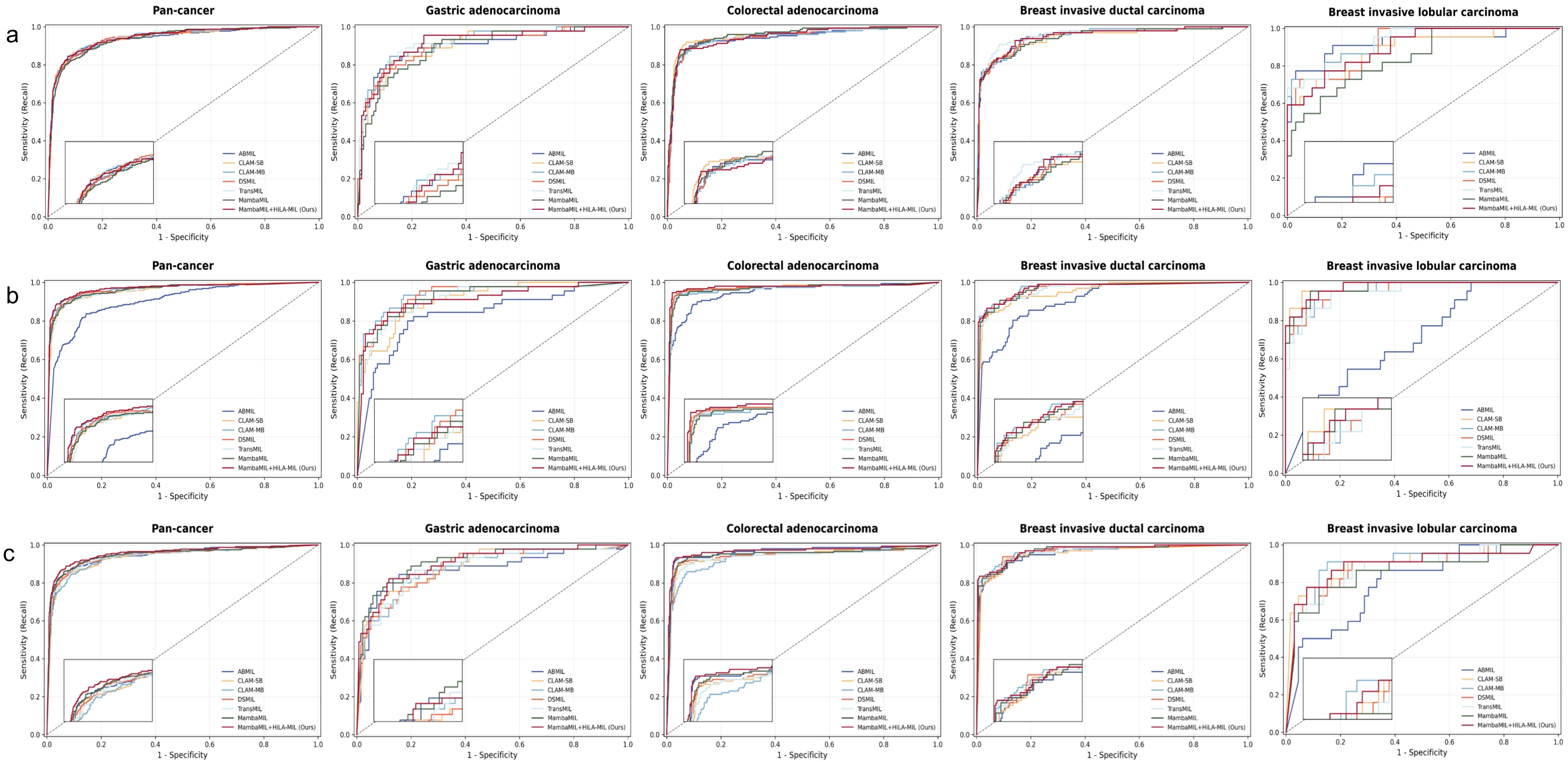

Supplementary Fig. 4 ROC analysis of multi-cancer LNM classification using different feature extractors on the DLZX dataset. ROC plots of the six baseline models as well as the MambaMIL+HiLA-MIL model for multi-cancer, gastric adenocarcinoma, colorectal adenocarcinoma, breast invasive ductal carcinoma, and breast invasive lobular carcinoma across with the (a) ResNet, (b) UNI, and (c) Virchow on the DLZX dataset. Source data are provided as a Source Data file.

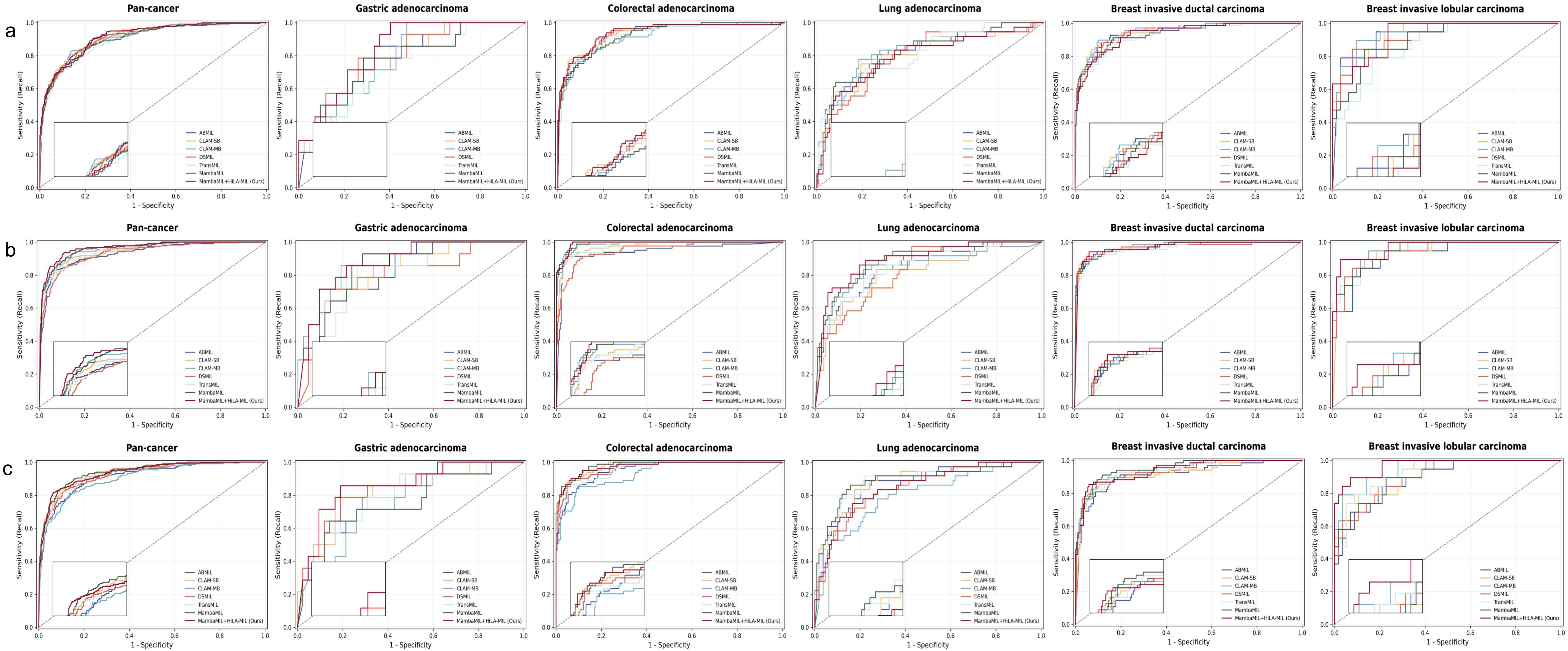

Supplementary Fig. 5 ROC analysis of multi-cancer LNM classification using different feature extractors on the YDSY dataset. ROC plots of the six baseline models as well as the MambaMIL+HiLA-MIL model for multi-cancer, gastric adenocarcinoma, colorectal adenocarcinoma, lung adenocarcinoma, breast invasive ductal carcinoma, and breast invasive lobular carcinoma across with the (a) ResNet, (b) UNI, and (c) Virchow on the YDSY dataset. Source data are provided as a Source Data file.

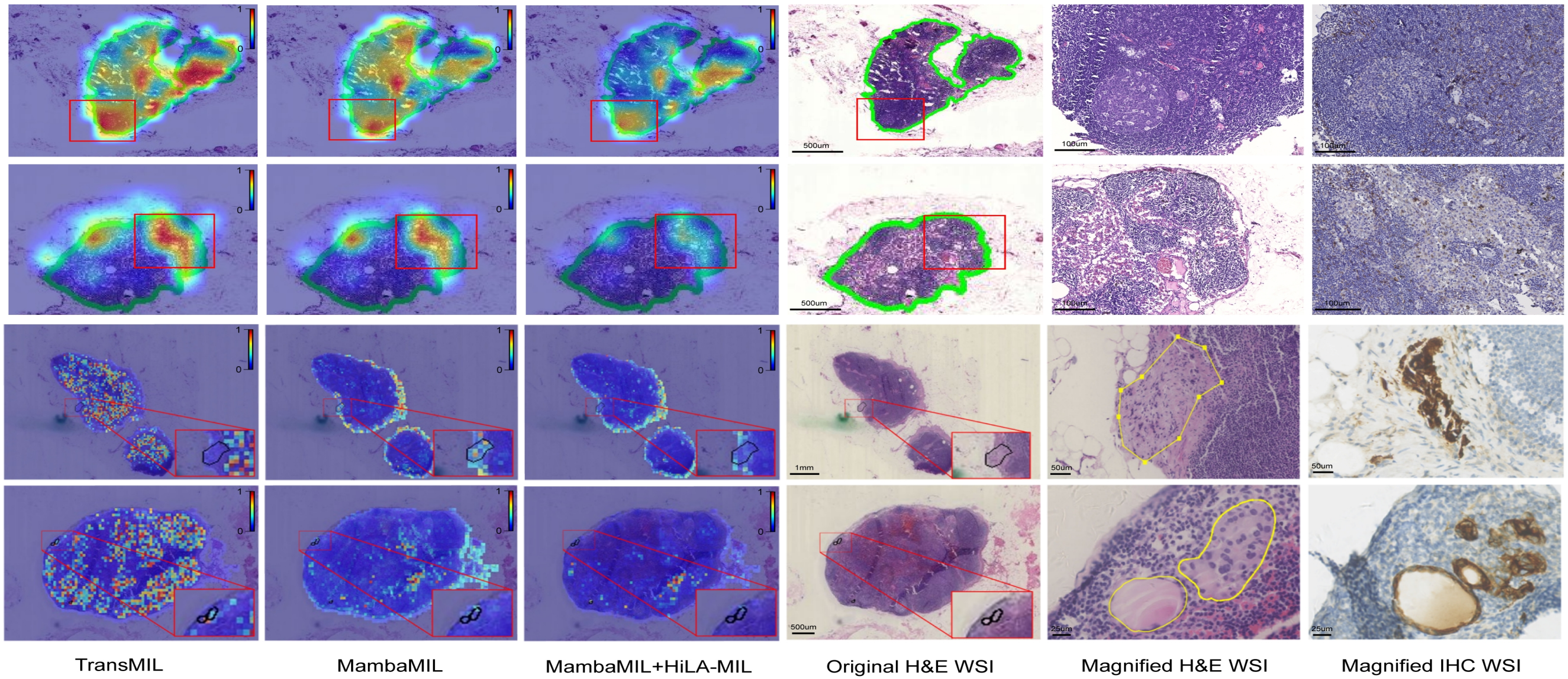

Supplementary Fig. 6 Visual retrospective analysis of multimodal WSIs using multiple MIL models. Visualization and retrospective analysis of images from the TransMIL, MambaMIL, and MambaMIL+HiLA-MIL models, as well as the original H&E, magnified H&E, and cytokeratin IHC WSIs. (a) Negative case of lung adenocarcinoma. (b) Negative case of papillary thyroid carcinoma. (c) ITCs of breast invasive ductal carcinoma. (d) ITCs of papillary thyroid carcinoma. Prediction score [0–1]. Red (value close to 1) represents high attention scores, blue (value close to 0) represents low attention scores, and the cancer metastasis areas annotated by pathologists are marked with black and yellow borders.

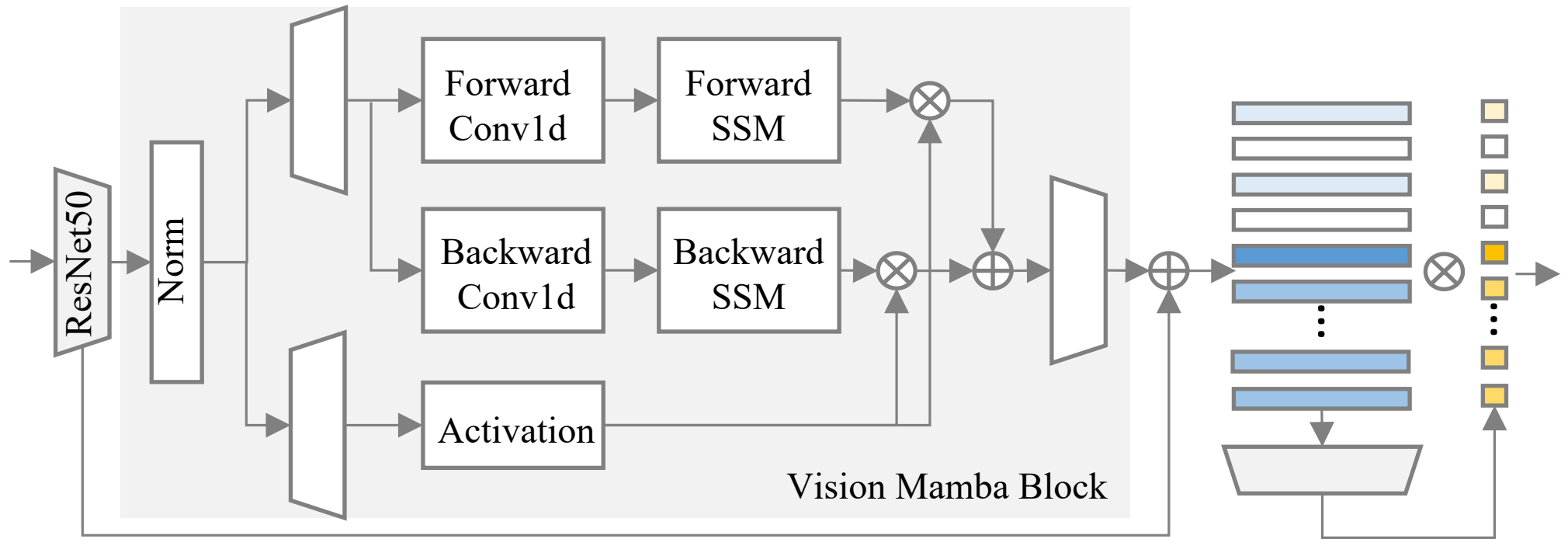

Supplementary Fig. 7 Architectural pipeline of the MambaMIL framework. Instance patches from whole slide images are processed through a pretrained backbone for feature extraction, followed by a layer normalization (Norm). The normalized features are then fed into the Vision Mamba Block for bidirectional sequence modeling. Within this block, the feature stream is projected into three parallel pathways: a forward pathway comprising a Forward Conv1d and a Forward SSM (State Space Model), a backward pathway consisting of a Backward Conv1d and a Backward SSM, and a gating pathway with an Activation function. The bidirectional contextual representations are modulated via element-wise multiplication with the activation gate, aggregated, and projected back to the original feature dimension. A residual skip connection is applied around the entire Vision Mamba Block. Finally, the refined instance features are aggregated through an attention-based MIL pooling operator to generate the final bag-level diagnostic prediction.

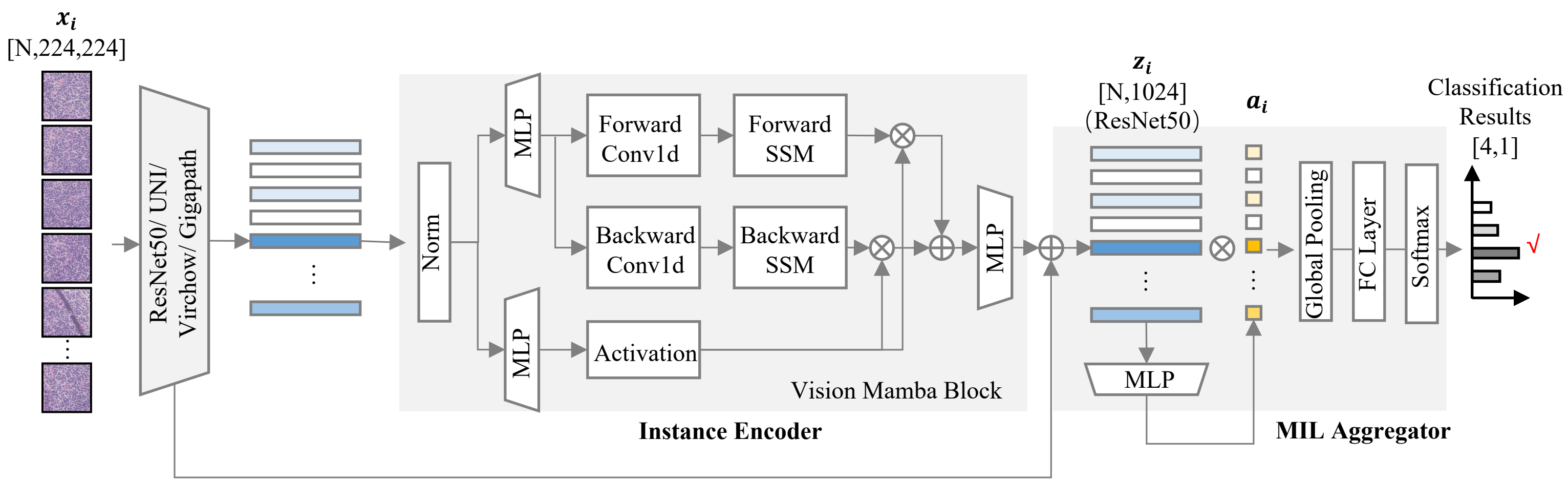

Supplementary Fig. 8 The architecture diagram illustrating the full pipeline of the attention mechanism. In the Instance Encoder stage, a sequence of instance patches xi is embedded into low-dimensional representations using alternative foundation backbones and refined by the Vision Mamba Block. In the MIL Aggregator stage, these resulting instance features zi are mapped through a MLP operator to explicitly calculate the instance-level attention weights ai (visualized as orange squares). The instance features zi are then modulated by their corresponding weights ai via element-wise multiplication. Finally, these weighted features are integrated via Global Pooling, projected through a Fully Connected (FC) Layer, and normalized by Softmax to yield the precise four-class probability distribution.

Supplementary Table 1. Evaluation metrics of overall four-class classification and each LNM category across four feature extractors on DLZX dataset

ResNet

Methods	Overall											
	AUC	P-value	Accuracy	P-value	Precision	P-value	Recall	P-value	Specificity	P-value	F1-Score	P-value
ABMIL	0.8939±0.0623	0.0068 (**↓)	0.7786±0.0492	<0.0001 (***↓)	0.6755±0.0971	0.3839 (ns↓)	0.6324±0.0699	0.0024 (**↓)	0.9118±0.0235	0.0011 (**↓)	0.6343±0.0760	0.0179 (*↓)
CLAM-SB	0.8890±0.0535	0.1277 (ns↓)	0.7762±0.0453	<0.0001 (***↓)	0.6610±0.0929	0.0154 (*↓)	0.6163±0.0660	<0.0001 (***↓)	0.9149±0.0219	0.0016 (**↓)	0.6212±0.0671	0.0008 (***↓)
CLAM-MB	0.8927±0.0474	0.6688 (ns↓)	0.7760±0.0543	<0.0001 (***↓)	0.6611±0.1023	0.0008 (***↓)	0.6343±0.0621	0.0010 (**↓)	0.9175±0.0226	0.0048 (**↓)	0.6349±0.0698	0.0008 (***↓)
DSMIL	0.8927±0.0565	0.0430 (*↓)	0.7910±0.0357	<0.0001 (***↓)	0.6721±0.0805	0.3302 (ns↓)	0.6550±0.0739	0.0100 (*↓)	0.9213±0.0193	0.0288 (*↓)	0.6522±0.0656	0.0491 (*↓)
TransMIL	0.8981±0.0496	0.2613 (ns↓)	0.7835±0.0476	<0.0001 (***↓)	0.6944±0.0857	0.0911 (ns↓)	0.6885±0.0813	0.2476 (ns↓)	0.9200±0.0198	0.0321 (*↓)	0.6750±0.0742	0.1418 (ns↓)
MambaMIL	0.8863±0.0520	0.1661 (ns↓)	0.7834±0.0400	<0.0001 (***↓)	0.7083±0.0832	0.4240 (ns↓)	0.6739±0.0578	0.0309 (*↓)	0.9178±0.0132	0.0111 (*↓)	0.6752±0.0658	0.1144 (ns↓)
MambaMIL+HiLA-MIL (Ours)	0.9080±0.0484	N/A	0.8209±0.0367	N/A	0.7423±0.0797	N/A	0.7142±0.0827	N/A	0.9316±0.0178	N/A	0.7129±0.0724	N/A

Methods	Negative											
	AUC	P-value	Accuracy	P-value	Precision	P-value	Recall	P-value	Specificity	P-value	F1-Score	P-value
ABMIL	0.9446±0.0375	0.1851 (ns↑)	0.8930±0.0485	0.0419 (*↓)	0.7685±0.1351	0.0598 (ns↓)	0.8556±0.1224	0.2802 (ns↓)	0.9051±0.0785	0.0484 (*↓)	0.7962±0.0735	0.0756 (ns↓)
CLAM-SB	0.9351±0.0427	0.5906 (ns↑)	0.8855±0.0315	0.0067 (**↓)	0.7426±0.1142	0.0078 (**↓)	0.8456±0.1130	0.1801 (ns↓)	0.8985±0.0521	0.0056 (**↓)	0.7796±0.0497	0.0158 (*↓)
CLAM-MB	0.9351±0.0588	0.9343 (ns↑)	0.8879±0.0396	0.0019 (**↓)	0.7515±0.1229	0.0065 (**↓)	0.8433±0.0765	0.0582 (ns↓)	0.9015±0.0604	0.0062 (**↓)	0.7861±0.0590	0.0053 (**↓)
DSMIL	0.9426±0.0394	0.2373 (ns↑)	0.8904±0.0273	0.0518 (ns↓)	0.7563±0.0854	0.0800 (ns↓)	0.8244±0.0867	0.0389 (*↓)	0.9116±0.0411	0.0907 (ns↓)	0.7828±0.0448	0.0242 (*↓)
TransMIL	0.9318±0.0506	0.1642 (ns↓)	0.8955±0.0405	0.0758 (ns↓)	0.7913±0.1096	0.3291 (ns↓)	0.7933±0.1338	0.0052 (**↓)	0.9276±0.0465	1.0000 (ns↓)	0.7830±0.0870	0.0331 (*↓)
MambaMIL	0.9305±0.0564	0.2009 (ns↓)	0.8855±0.0378	0.0070 (**↓)	0.7656±0.1484	0.0299 (*↓)	0.8233±0.1109	0.0377 (*↓)	0.9052±0.0729	0.0350 (*↓)	0.7776±0.0556	0.0113 (*↓)
MambaMIL+HiLA-MIL (Ours)	0.9327±0.0591	N/A	0.9154±0.0269	N/A	0.8087±0.0988	N/A	0.8733±0.0867	N/A	0.9276±0.0465	N/A	0.8326±0.0482	N/A

Methods	ITC											
	AUC	P-value	Accuracy	P-value	Precision	P-value	Recall	P-value	Specificity	P-value	F1-Score	P-value
ABMIL	0.8334±0.1735	0.0776 (ns↓)	0.9554±0.0252	1.0000 (ns↓)	0.5000±0.4714	0.5662 (ns↓)	0.3333±0.3333	<0.0001 (***↓)	0.9896±0.0181	0.2193 (ns↑)	0.3833±0.3605	0.1651 (ns↓)
CLAM-SB	0.8355±0.1605	0.6420 (ns↓)	0.9479±0.0271	0.1812 (ns↓)	0.5000±0.4714	0.5021 (ns↓)	0.2833±0.2491	<0.0001 (***↓)	0.9843±0.0219	0.6274 (ns↑)	0.3500±0.3088	0.0457 (*↓)
CLAM-MB	0.8450±0.1558	0.6193 (ns↓)	0.9404±0.0335	0.0705 (ns↓)	0.4250±0.4417	0.0071 (**↓)	0.3333±0.3333	<0.0001 (***↓)	0.9738±0.0276	0.1243 (ns↓)	0.3500±0.3465	0.0071 (**↓)
DSMIL	0.8348±0.1884	0.1649 (ns↓)	0.9552±0.0258	1.0000 (ns↓)	0.4667±0.4431	0.7505 (ns↓)	0.3833±0.3932	<0.0001 (***↓)	0.9868±0.0186	0.4455 (ns↑)	0.3967±0.3743	0.3643 (ns↓)
TransMIL	0.8692±0.1347	0.7729 (ns↓)	0.9502±0.0354	0.7762 (ns↓)	0.5400±0.4241	0.4461 (ns↓)	0.5333±0.3752	1.0000 (ns↓)	0.9737±0.0372	0.3456 (ns↓)	0.4905±0.3476	0.5957 (ns↓)
MambaMIL	0.8356±0.1478	0.9451 (ns↓)	0.9603±0.0266	0.6819 (ns↑)	0.6333±0.4216	0.2180 (ns↑)	0.4833±0.3375	<0.0001 (***↓)	0.9869±0.0186	0.3691 (ns↑)	0.5233±0.3432	1.0000 (ns↓)

MambaMIL+HiLA-MIL (Ours)	0.8828±0.1345	N/A	0.9554±0.0342	N/A	0.6000±0.3944	N/A	0.5333±0.3752	N/A	0.9791±0.0267	N/A	0.5333±0.3407	N/A

Methods	Micro											
	AUC	P-value	Accuracy	P-value	Precision	P-value	Recall	P-value	Specificity	P-value	F1-Score	P-value
ABMIL	0.8530±0.1104	0.1230 (ns↓)	0.8481±0.0452	0.0597 (ns↓)	0.5762±0.1795	0.1316 (ns↓)	0.4429±0.2279	0.0585 (ns↓)	0.9336±0.0345	0.3046 (ns↓)	0.4839±0.1931	0.0671 (ns↓)
CLAM-SB	0.8414±0.0823	0.0216 (*↓)	0.8357±0.0476	0.0043 (**↓)	0.5167±0.1822	0.0158 (*↓)	0.4286±0.2130	0.0098 (**↓)	0.9217±0.0253	0.1632 (ns↓)	0.4614±0.1927	0.0050 (**↓)
CLAM-MB	0.8481±0.0867	0.7869 (ns↓)	0.8406±0.0534	0.0095 (**↓)	0.5719±0.2043	0.0152 (*↓)	0.4714±0.1176	0.1115 (ns↓)	0.9185±0.0432	0.0223 (*↓)	0.5128±0.1432	0.0471 (*↓)
DSMIL	0.8505±0.0940	0.1526 (ns↓)	0.8432±0.0529	0.0145 (*↓)	0.5698±0.1762	0.0173 (*↓)	0.5000±0.1543	0.2856 (ns↓)	0.9157±0.0422	0.0109 (*↓)	0.5262±0.1496	0.0958 (ns↓)
TransMIL	0.8457±0.0915	0.0119 (*↓)	0.8430±0.0558	0.0188 (*↓)	0.5545±0.1768	0.0120 (*↓)	0.5429±0.2108	0.4971 (ns↑)	0.9066±0.0414	0.0018 (**↓)	0.5403±0.1701	0.2962 (ns↓)
MambaMIL	0.8375±0.0915	0.0161 (*↓)	0.8455±0.0516	0.0665 (ns↓)	0.5523±0.1659	0.0201 (*↓)	0.5000±0.2156	0.3917 (ns↓)	0.9185±0.0382	0.0050 (**↓)	0.5159±0.1912	0.1869 (ns↓)
MambaMIL+HiLA-MIL (Ours)	0.8673±0.0829	N/A	0.8704±0.0486	N/A	0.6617±0.1934	N/A	0.5286±0.1911	N/A	0.9426±0.0390	N/A	0.5769±0.1865	N/A

Methods	Macro											
	AUC	P-value	Accuracy	P-value	Precision	P-value	Recall	P-value	Specificity	P-value	F1-Score	P-value
ABMIL	0.9445±0.0304	0.0371 (*↓)	0.8607±0.0487	0.0018 (**↓)	0.8575±0.0734	0.0032 (**↓)	0.8978±0.0653	0.0945 (ns↓)	0.8189±0.1126	0.0027 (**↓)	0.8738±0.0424	0.0050 (**↓)
CLAM-SB	0.9438±0.0269	0.1105 (ns↓)	0.8832±0.0469	0.1851 (ns↓)	0.8848±0.0674	0.1338 (ns↓)	0.9076±0.0523	0.2274 (ns↓)	0.8549±0.1042	0.1064 (ns↓)	0.8937±0.0382	0.1602 (ns↓)
CLAM-MB	0.9428±0.0318	0.5909 (ns↓)	0.8832±0.0664	0.1866 (ns↓)	0.8960±0.0657	0.7600 (ns↓)	0.8892±0.0783	0.0114 (*↓)	0.8763±0.0891	1.0000 (ns↓)	0.8908±0.0617	0.0936 (ns↓)
DSMIL	0.9429±0.0309	0.3434 (ns↓)	0.8932±0.0618	0.2996 (ns↓)	0.8957±0.0755	0.5220 (ns↓)	0.9121±0.0398	0.2839 (ns↓)	0.8710±0.0997	1.0000 (ns↓)	0.9030±0.0533	0.3688 (ns↓)
TransMIL	0.9455±0.0255	0.2291 (ns↓)	0.8782±0.0430	0.0908 (ns↓)	0.8918±0.0607	0.5549 (ns↓)	0.8844±0.0533	0.0061 (**↓)	0.8721±0.0759	1.0000 (ns↓)	0.8863±0.0381	0.0408 (*↓)
MambaMIL	0.9415±0.0279	0.5254 (ns↓)	0.8755±0.0393	0.0163 (*↓)	0.8820±0.0381	0.2250 (ns↓)	0.8890±0.0649	0.0126 (*↓)	0.8608±0.0528	0.2244 (ns↓)	0.8839±0.0368	0.0191 (*↓)
MambaMIL+HiLA-MIL (Ours)	0.9493±0.0297	N/A	0.9006±0.0468	N/A	0.8989±0.0571	N/A	0.9214±0.0481	N/A	0.8771±0.0754	N/A	0.9089±0.0424	N/A

Supplementary Table 1. Continued

UNI

Methods	Overall											
	AUC	P-value	Accuracy	P-value	Precision	P-value	Recall	P-value	Specificity	P-value	F1-Score	P-value
ABMIL	0.9307±0.0302	0.0017 (**↓)	0.8337±0.0523	<0.0001 (***↓)	0.8228±0.0756	0.1835 (ns↓)	0.7428±0.0665	0.0018 (**↓)	0.9378±0.0179	0.0144 (*↓)	0.7606±0.0675	0.0048 (**↓)
CLAM-SB	0.9397±0.0358	0.2150 (ns↓)	0.8683±0.0436	0.1371 (ns↓)	0.8551±0.0811	0.6823 (ns↓)	0.7786±0.0646	0.0328 (*↓)	0.9498±0.0152	0.6719 (ns↓)	0.7952±0.0634	0.1200 (ns↓)
CLAM-MB	0.9351±0.0320	0.0474 (*↓)	0.8360±0.0397	<0.0001 (***↓)	0.7716±0.1246	0.0101 (*↓)	0.7191±0.0824	0.0003 (***↓)	0.9374±0.0140	0.0099 (**↓)	0.7200±0.0882	0.0005 (***↓)
DSMIL	0.9278±0.0298	0.0135 (*↓)	0.8310±0.0461	<0.0001 (***↓)	0.7928±0.0943	0.0542 (ns↓)	0.7317±0.0777	0.0003 (***↓)	0.9327±0.0187	0.0006 (***↓)	0.7388±0.0793	0.0018 (**↓)
TransMIL	0.9286±0.0240	0.0006 (***↓)	0.8286±0.0579	<0.0001 (***↓)	0.7872±0.0915	0.0353 (*↓)	0.7301±0.0851	0.0008 (***↓)	0.9302±0.0194	0.0026 (**↓)	0.7318±0.0855	0.0033 (**↓)

MambaMIL	0.9394±0.0343	0.1350 (ns↓)	0.8534±0.0515	<0.0001 (***↓)	0.8443±0.0848	0.4071 (ns↓)	0.7606±0.0618	0.0050 (**↓)	0.9415±0.0197	0.0475 (*↓)	0.7789±0.0665	0.0291 (*↓)
MambaMIL+HiLA-MIL (Ours)	0.9483±0.0273	N/A	0.8784±0.0521	N/A	0.8664±0.0719	N/A	0.8481±0.0669	N/A	0.9518±0.0194	N/A	0.8420±0.0691	N/A

Methods	Negative											
	AUC	P-value	Accuracy	P-value	Precision	P-value	Recall	P-value	Specificity	P-value	F1-Score	P-value
ABMIL	0.9687±0.0412	0.8466 (ns↑)	0.9329±0.0497	0.0140 (*↓)	0.8288±0.1203	<0.0001 (***↓)	0.9156±0.1012	0.1195 (ns↑)	0.9381±0.0444	<0.0001 (***↓)	0.8677±0.1025	0.0140 (*↓)
CLAM-SB	0.9697±0.0424	0.4380 (ns↑)	0.9428±0.0409	0.0345 (*↓)	0.8549±0.1109	0.0017 (**↓)	0.9244±0.1047	0.0605 (ns↑)	0.9478±0.0413	<0.0001 (***↓)	0.8844±0.0899	0.1205 (ns↓)
CLAM-MB	0.9671±0.0370	0.8516 (ns↑)	0.9205±0.0477	0.0001 (***↓)	0.7992±0.1161	<0.0001 (***↓)	0.9056±0.1076	0.3686 (ns↑)	0.9249±0.0461	<0.0001 (***↓)	0.8453±0.0975	0.0015 (**↓)
DSMIL	0.9650±0.0458	0.3782 (ns↓)	0.9379±0.0456	0.0214 (*↓)	0.8541±0.1020	0.0006 (***↓)	0.8956±0.0850	1.0000 (ns↓)	0.9511±0.0352	<0.0001 (***↓)	0.8738±0.0917	0.0200 (*↓)
TransMIL	0.9655±0.0332	0.4540 (ns↓)	0.9232±0.0580	0.0001 (***↓)	0.8265±0.1177	0.0007 (***↓)	0.8444±0.2012	0.0667 (ns↓)	0.9480±0.0316	0.0006 (***↓)	0.8278±0.1626	0.0021 (**↓)
MambaMIL	0.9691±0.0426	0.6725 (ns↑)	0.9429±0.0371	0.0349 (*↓)	0.8656±0.1089	0.0028 (**↓)	0.9144±0.1014	0.2144 (ns↑)	0.9511±0.0442	<0.0001 (***↓)	0.8844±0.0807	0.1202 (ns↓)
MambaMIL+HiLA-MIL (Ours)	0.9663±0.0423	N/A	0.9603±0.0314	N/A	0.9365±0.0760	N/A	0.8956±0.0972	N/A	0.9805±0.0226	N/A	0.9126±0.0720	N/A

Methods	ITC											
	AUC	P-value	Accuracy	P-value	Precision	P-value	Recall	P-value	Specificity	P-value	F1-Score	P-value
ABMIL	0.9457±0.0668	0.0069 (**↓)	0.9702±0.0155	0.3836 (ns↓)	0.9000±0.2108	0.5065 (ns↑)	0.5333±0.1721	<0.0001 (***↓)	0.9948±0.0110	0.3657 (ns↑)	0.6500±0.1459	0.0797 (ns↓)
CLAM-SB	0.9576±0.0402	0.0984 (ns↓)	0.9702±0.0155	0.3852 (ns↓)	0.8667±0.2194	0.8885 (ns↑)	0.5833±0.2257	<0.0001 (***↓)	0.9922±0.0126	0.6764 (ns↑)	0.6633±0.1535	0.0903 (ns↓)
CLAM-MB	0.9558±0.0547	0.0251 (*↓)	0.9602±0.0210	0.0552 (ns↓)	0.6400±0.4169	0.2655 (ns↓)	0.4833±0.3375	<0.0001 (***↓)	0.9868±0.0256	1.0000 (ns↓)	0.5071±0.3051	0.0095 (**↓)
DSMIL	0.9269±0.0674	0.0142 (*↓)	0.9652±0.0173	0.1767 (ns↓)	0.7500±0.3536	0.7285 (ns↓)	0.5333±0.2919	<0.0001 (***↓)	0.9895±0.0184	1.0000 (ns↑)	0.5833±0.2515	0.0354 (*↓)
TransMIL	0.9589±0.0438	<0.0001 (***↓)	0.9627±0.0175	0.1426 (ns↓)	0.7000±0.3496	0.3118 (ns↓)	0.5833±0.3263	0.0094 (**↓)	0.9842±0.0222	1.0000 (ns↓)	0.5833±0.2515	0.0436 (*↓)
MambaMIL	0.9537±0.0463	0.1067 (ns↓)	0.9702±0.0155	0.3868 (ns↓)	0.8667±0.2194	0.8899 (ns↑)	0.5833±0.2257	<0.0001 (***↓)	0.9922±0.0126	0.6814 (ns↑)	0.6633±0.1535	0.0902 (ns↓)
MambaMIL+HiLA-MIL (Ours)	0.9785±0.0376	N/A	0.9802±0.0225	N/A	0.8500±0.1995	N/A	0.8667±0.2194	N/A	0.9868±0.0186	N/A	0.8305±0.1628	N/A

Methods	Micro											
	AUC	P-value	Accuracy	P-value	Precision	P-value	Recall	P-value	Specificity	P-value	F1-Score	P-value
ABMIL	0.8687±0.0627	0.0498 (*↓)	0.8684±0.0413	0.0063 (**↓)	0.6518±0.1330	0.0095 (**↓)	0.6286±0.1380	0.1152 (ns↓)	0.9189±0.0576	0.0126 (*↓)	0.6248±0.0925	0.0194 (*↓)
CLAM-SB	0.8857±0.0821	0.8887 (ns↓)	0.9005±0.0405	1.0000 (ns↓)	0.7762±0.1972	0.9112 (ns↑)	0.6714±0.1176	0.4239 (ns↓)	0.9488±0.0490	0.5497 (ns↑)	0.7054±0.1095	0.7005 (ns↓)
CLAM-MB	0.8751±0.0791	0.4609 (ns↓)	0.8807±0.0342	0.0431 (*↓)	0.7403±0.2007	0.4068 (ns↓)	0.5571±0.1838	0.0038 (**↓)	0.9490±0.0446	0.5029 (ns↑)	0.6105±0.1267	0.0198 (*↓)
DSMIL	0.8764±0.0795	0.1606 (ns↓)	0.8731±0.0342	0.0124 (*↓)	0.6858±0.1598	0.0653 (ns↓)	0.5857±0.2177	0.0139 (*↓)	0.9339±0.0527	0.1751 (ns↓)	0.6041±0.1227	0.0142 (*↓)
TransMIL	0.8552±0.0814	0.0261 (*↓)	0.8807±0.0457	0.1149 (ns↓)	0.7517±0.1878	0.5602 (ns↓)	0.5571±0.1710	0.0157 (*↓)	0.9493±0.0588	0.6452 (ns↑)	0.6153±0.1246	0.0605 (ns↓)

MambaMIL	0.8898±0.0786	0.9097 (ns↓)	0.8881±0.0392	0.1329 (ns↓)	0.7470±0.1966	0.5164 (ns↓)	0.6143±0.1513	0.0355 (*↓)	0.9458±0.0464	1.0000 (ns↓)	0.6552±0.1170	0.1156 (ns↓)
MambaMIL+HiLA-MIL (Ours)	0.8970±0.0759	N/A	0.9032±0.0423	N/A	0.7693±0.1746	N/A	0.7000±0.2177	N/A	0.9461±0.0536	N/A	0.7064±0.1470	N/A

Methods	Macro											
	AUC	P-value	Accuracy	P-value	Precision	P-value	Recall	P-value	Specificity	P-value	F1-Score	P-value
ABMIL	0.9399±0.0435	0.0080 (**↓)	0.8958±0.0524	0.2000 (ns↓)	0.9107±0.0451	0.9921 (ns↑)	0.8939±0.0909	0.0058 (**↓)	0.8996±0.0509	1.0000 (ns↑)	0.8998±0.0562	0.0916 (ns↓)
CLAM-SB	0.9460±0.0431	0.9427 (ns↓)	0.9230±0.0413	0.3891 (ns↑)	0.9227±0.0422	0.1853 (ns↑)	0.9351±0.0624	1.0000 (ns↑)	0.9101±0.0486	0.4542 (ns↑)	0.9276±0.0400	0.2501 (ns↑)
CLAM-MB	0.9423±0.0321	0.3060 (ns↓)	0.9105±0.0312	1.0000 (ns↓)	0.9069±0.0425	0.8568 (ns↓)	0.9305±0.0587	1.0000 (ns↑)	0.8888±0.0553	0.5409 (ns↓)	0.9169±0.0303	0.9125 (ns↓)
DSMIL	0.9432±0.0278	0.5195 (ns↓)	0.8857±0.0390	0.0068 (**↓)	0.8815±0.0439	0.0232 (*↓)	0.9121±0.0759	0.0658 (ns↓)	0.8563±0.0596	0.0211 (*↓)	0.8941±0.0384	0.0129 (*↓)
TransMIL	0.9347±0.0475	0.4420 (ns↓)	0.8905±0.0476	0.1559 (ns↓)	0.8705±0.0379	0.0260 (*↓)	0.9355±0.0685	1.0000 (ns↑)	0.8394±0.0507	0.0110 (*↓)	0.9008±0.0447	0.1491 (ns↓)
MambaMIL	0.9450±0.0381	0.2604 (ns↓)	0.9055±0.0421	0.5757 (ns↓)	0.8980±0.0398	0.2737 (ns↓)	0.9305±0.0660	1.0000 (ns↑)	0.8768±0.0519	0.1737 (ns↓)	0.9127±0.0393	0.4189 (ns↓)
MambaMIL+HiLA-MIL (Ours)	0.9515±0.0392	N/A	0.9131±0.0499	N/A	0.9098±0.0458	N/A	0.9303±0.0735	N/A	0.8937±0.0531	N/A	0.9186±0.0490	N/A

Supplementary Table 1. Continued

GigaPath

Methods	Overall											
	AUC	P-value	Accuracy	P-value	Precision	P-value	Recall	P-value	Specificity	P-value	F1-Score	P-value
ABMIL	0.9359±0.0223	<0.0001 (***↓)	0.8457±0.0484	<0.0001 (***↓)	0.7841±0.1019	0.1281 (ns↓)	0.7313±0.0443	0.0003 (***↓)	0.9378±0.0243	0.0024 (**↓)	0.7403±0.0561	0.0016 (**↓)
CLAM-SB	0.9171±0.0316	0.0089 (**↓)	0.8285±0.0534	<0.0001 (***↓)	0.7779±0.0867	0.0674 (ns↓)	0.7242±0.0628	<0.0001 (***↓)	0.9329±0.0236	0.0002 (***↓)	0.7295±0.0585	0.0004 (***↓)
CLAM-MB	0.9195±0.0382	0.0451 (*↓)	0.8385±0.0507	<0.0001 (***↓)	0.7949±0.0902	0.1742 (ns↓)	0.7377±0.0622	0.0001 (***↓)	0.9357±0.0214	0.0008 (***↓)	0.7452±0.0607	0.0016 (**↓)
DSMIL	0.9339±0.0342	0.0248 (*↓)	0.8335±0.0400	<0.0001 (***↓)	0.8186±0.0605	0.0068 (**↓)	0.7634±0.0695	0.0002 (***↓)	0.9359±0.0166	0.0004 (***↓)	0.7701±0.0594	0.0004 (***↓)
TransMIL	0.9299±0.0386	0.0811 (ns↓)	0.8509±0.0280	<0.0001 (***↓)	0.8367±0.0505	0.0251 (*↓)	0.7738±0.0623	0.0022 (**↓)	0.9414±0.0124	0.0134 (*↓)	0.7864±0.0486	0.0026 (**↓)
MambaMIL	0.9325±0.0284	0.0142 (*↓)	0.8684±0.0395	0.0004 (***↓)	0.8637±0.0687	0.0337 (*↓)	0.8091±0.0579	0.0529 (ns↓)	0.9494±0.0129	0.1780 (ns↓)	0.8163±0.0504	0.0302 (*↓)
MambaMIL+HiLA-MIL (Ours)	0.9440±0.0284	N/A	0.8882±0.0307	N/A	0.8962±0.0320	N/A	0.8339±0.0555	N/A	0.9562±0.0122	N/A	0.8432±0.0504	N/A

Methods	Negative											
	AUC	P-value	Accuracy	P-value	Precision	P-value	Recall	P-value	Specificity	P-value	F1-Score	P-value
ABMIL	0.9697±0.0344	0.0063 (**↑)	0.9452±0.0308	<0.0001 (***↓)	0.8802±0.0838	<0.0001 (***↓)	0.8922±0.1049	<0.0001 (***↓)	0.9609±0.0253	0.1291 (ns↓)	0.8826±0.0744	<0.0001 (***↓)
CLAM-SB	0.9615±0.0378	0.1676 (ns↓)	0.9206±0.0526	<0.0001 (***↓)	0.8096±0.1263	<0.0001 (***↓)	0.9033±0.0970	<0.0001 (***↓)	0.9251±0.0648	<0.0001 (***↓)	0.8476±0.0917	<0.0001 (***↓)
CLAM-MB	0.9608±0.0405	0.1857 (ns↓)	0.9404±0.0392	<0.0001 (***↓)	0.8546±0.1019	<0.0001 (***↓)	0.9144±0.1014	<0.0001 (***↓)	0.9477±0.0416	<0.0001 (***↓)	0.8797±0.0838	<0.0001 (***↓)
DSMIL	0.9645±0.0406	0.0260 (*↓)	0.9404±0.0410	<0.0001 (***↓)	0.8756±0.1035	<0.0001 (***↓)	0.8844±0.1257	<0.0001 (***↓)	0.9576±0.0379	0.0649 (ns↓)	0.8743±0.0907	<0.0001 (***↓)

TransMIL	0.9666±0.0377	0.6516 (ns↓)	0.9502±0.0373	<0.0001 (***↓)	0.9059±0.0953	0.0308 (*↓)	0.8833±0.1089	<0.0001 (***↓)	0.9706±0.0286	1.0000 (ns↓)	0.8918±0.0879	<0.0001 (***↓)
MambaMIL	0.9593±0.0390	0.0000 (***↓)	0.9454±0.0304	<0.0001 (***↓)	0.8844±0.0954	<0.0001 (***↓)	0.8944±0.0527	<0.0001 (***↓)	0.9610±0.0335	0.1254 (ns↓)	0.8872±0.0617	<0.0001 (***↓)
MambaMIL+HiLA-MIL (Ours)	0.9678±0.0391	N/A	0.9651±0.0317	N/A	0.9194±0.0827	N/A	0.9356±0.0764	N/A	0.9740±0.0255	N/A	0.9261±0.0702	N/A

Methods	ITC											
	AUC	P-value	Accuracy	P-value	Precision	P-value	Recall	P-value	Specificity	P-value	F1-Score	P-value
ABMIL	0.9400±0.0617	<0.0001 (***↓)	0.9677±0.0235	0.1763 (ns↓)	0.7500±0.4249	0.6424 (ns↓)	0.4833±0.3375	<0.0001 (***↓)	0.9948±0.0110	1.0000 (ns↑)	0.5667±0.3443	0.0375 (*↓)
CLAM-SB	0.8983±0.0907	0.1372 (ns↓)	0.9727±0.0183	0.4497 (ns↓)	0.7667±0.4172	0.9529 (ns↓)	0.5333±0.3752	<0.0001 (***↓)	0.9974±0.0083	0.2532 (ns↑)	0.5967±0.3508	0.1263 (ns↓)
CLAM-MB	0.8889±0.1041	0.2321 (ns↓)	0.9727±0.0183	0.4504 (ns↓)	0.7667±0.4172	0.9514 (ns↓)	0.5333±0.3752	<0.0001 (***↓)	0.9974±0.0083	0.2499 (ns↑)	0.5967±0.3508	0.1264 (ns↓)
DSMIL	0.9177±0.0857	0.1350 (ns↓)	0.9677±0.0264	0.1821 (ns↓)	0.8333±0.2833	0.1264 (ns↓)	0.6333±0.2582	<0.0001 (***↓)	0.9868±0.0224	0.4547 (ns↓)	0.6767±0.2132	0.0399 (*↓)
TransMIL	0.9289±0.0758	0.4456 (ns↓)	0.9727±0.0183	0.3759 (ns↓)	0.8667±0.2194	0.2468 (ns↓)	0.6333±0.2582	<0.0001 (***↓)	0.9921±0.0127	1.0000 (ns↓)	0.6967±0.1869	0.0617 (ns↓)
MambaMIL	0.9205±0.0809	0.0892 (ns↓)	0.9752±0.0165	0.5086 (ns↓)	0.8833±0.1933	<0.0001 (***↓)	0.7167±0.2491	1.0000 (ns↓)	0.9895±0.0184	<0.0001 (***↓)	0.7467±0.1398	0.5086 (ns↓)
MambaMIL+HiLA-MIL (Ours)	0.9420±0.0712	N/A	0.9802±0.0155	N/A	0.9333±0.1405	N/A	0.7167±0.2491	N/A	0.9947±0.0111	N/A	0.7800±0.1573	N/A

Methods	Micro											
	AUC	P-value	Accuracy	P-value	Precision	P-value	Recall	P-value	Specificity	P-value	F1-Score	P-value
ABMIL	0.8838±0.0540	0.0007 (***↓)	0.8780±0.0548	0.0278 (*↓)	0.6170±0.2625	0.0831 (ns↓)	0.6143±0.2939	0.0038 (**↓)	0.9337±0.0490	0.2593 (ns↓)	0.6022±0.2607	0.0072 (**↓)
CLAM-SB	0.8572±0.0833	0.0134 (*↓)	0.8682±0.0526	0.0028 (**↓)	0.6399±0.1940	0.0430 (*↓)	0.5429±0.2502	0.0003 (***↓)	0.9366±0.0484	0.6504 (ns↓)	0.5700±0.2082	0.0008 (***↓)
CLAM-MB	0.8755±0.0775	0.1034 (ns↓)	0.8707±0.0505	0.0018 (**↓)	0.6677±0.1688	0.0283 (*↓)	0.5857±0.2070	0.0019 (**↓)	0.9308±0.0566	0.3590 (ns↓)	0.6037±0.1609	0.0013 (**↓)
DSMIL	0.8986±0.0589	0.2150 (ns↓)	0.8732±0.0377	0.0033 (**↓)	0.6702±0.1304	0.0154 (*↓)	0.6429±0.1388	0.0138 (*↓)	0.9219±0.0583	0.1129 (ns↓)	0.6383±0.0822	0.0043 (**↓)
TransMIL	0.8794±0.0821	0.0913 (ns↓)	0.8782±0.0269	0.0190 (*↓)	0.6752±0.1024	0.0205 (*↓)	0.6571±0.1536	0.0344 (*↓)	0.9250±0.0522	0.0464 (*↓)	0.6498±0.0658	0.0098 (**↓)
MambaMIL	0.8984±0.0522	0.3400 (ns↓)	0.9007±0.0346	0.4243 (ns↓)	0.7674±0.1634	0.5578 (ns↓)	0.7000±0.1421	0.1057 (ns↓)	0.9430±0.0554	1.0000 (ns↓)	0.7112±0.0865	0.2238 (ns↓)
MambaMIL+HiLA-MIL (Ours)	0.9121±0.0598	N/A	0.9130±0.0332	N/A	0.8079±0.1632	N/A	0.7571±0.2026	N/A	0.9460±0.0573	N/A	0.7437±0.1175	N/A

Methods	Macro											
	AUC	P-value	Accuracy	P-value	Precision	P-value	Recall	P-value	Specificity	P-value	F1-Score	P-value
ABMIL	0.9501±0.0366	0.0088 (**↓)	0.9005±0.0644	0.2683 (ns↓)	0.8892±0.0737	0.0339 (*↓)	0.9355±0.0685	0.7711 (ns↑)	0.8619±0.1013	0.0128 (*↓)	0.9099±0.0570	0.2605 (ns↓)
CLAM-SB	0.9512±0.0330	0.4440 (ns↓)	0.8956±0.0630	0.1659 (ns↓)	0.8952±0.0723	0.0692 (ns↓)	0.9171±0.0733	0.4621 (ns↓)	0.8724±0.0952	0.0495 (*↓)	0.9039±0.0567	0.1404 (ns↓)
CLAM-MB	0.9529±0.0336	0.6553 (ns↓)	0.8932±0.0652	0.0415 (*↓)	0.8908±0.0649	0.0324 (*↓)	0.9173±0.0952	0.4195 (ns↓)	0.8669±0.0883	0.0211 (*↓)	0.9008±0.0610	0.0833 (ns↓)
DSMIL	0.9549±0.0318	0.5241 (ns↑)	0.8857±0.0562	0.0098 (**↓)	0.8951±0.0514	0.0784 (ns↓)	0.8929±0.0938	0.0353 (*↓)	0.8771±0.0715	0.0764 (ns↓)	0.8912±0.0577	0.0155 (*↓)

TransMIL	0.9449±0.0378	0.2091 (ns↓)	0.9007±0.0417	0.2401 (ns↓)	0.8991±0.0506	0.1284 (ns↓)	0.9214±0.0774	1.0000 (ns↓)	0.8777±0.0698	0.0952 (ns↓)	0.9073±0.0424	0.1850 (ns↓)
MambaMIL	0.9517±0.0306	0.5969 (ns↓)	0.9156±0.0369	1.0000 (ns↓)	0.9198±0.0457	0.8076 (ns↓)	0.9251±0.0739	1.0000 (ns↓)	0.9040±0.0608	1.0000 (ns↓)	0.9201±0.0379	0.8068 (ns↓)
MambaMIL+HiLA-MIL (Ours)	0.9541±0.0326	N/A	0.9181±0.0401	N/A	0.9242±0.0522	N/A	0.9262±0.0683	N/A	0.9101±0.0654	N/A	0.9229±0.0394	N/A

Supplementary Table 1. Continued

Virchow

Methods	Overall											
	AUC	P-value	Accuracy	P-value	Precision	P-value	Recall	P-value	Specificity	P-value	F1-Score	P-value
ABMIL	0.9097±0.0536	0.1041 (ns↓)	0.8160±0.0577	0.0227 (*↓)	0.7593±0.1037	0.0162 (*↓)	0.7290±0.0980	0.0025 (**↓)	0.9314±0.0237	0.2988 (ns↓)	0.7220±0.0897	0.0017 (**↓)
CLAM-SB	0.9213±0.0469	0.2365 (ns↓)	0.8135±0.0551	0.0071 (**↓)	0.7353±0.0943	0.0513 (ns↓)	0.7098±0.0835	0.0005 (***↓)	0.9282±0.0238	0.1182 (ns↓)	0.7063±0.0778	0.0021 (**↓)
CLAM-MB	0.9094±0.0506	0.0933 (ns↓)	0.8085±0.0539	0.0011 (**↓)	0.6999±0.1008	0.0020 (**↓)	0.6937±0.0918	0.0007 (***↓)	0.9285±0.0189	0.1423 (ns↓)	0.6773±0.0895	0.0005 (***↓)
DSMIL	0.9108±0.0393	0.1104 (ns↓)	0.8112±0.0462	0.0031 (**↓)	0.7356±0.0830	0.0208 (*↓)	0.7104±0.0875	0.0012 (**↓)	0.9289±0.0180	0.1682 (ns↓)	0.7041±0.0727	0.0020 (**↓)
TransMIL	0.9065±0.0549	0.0040 (**↓)	0.7887±0.0364	<0.0001 (***↓)	0.7136±0.0825	0.0009 (***↓)	0.6924±0.0776	<0.0001 (***↓)	0.9219±0.0156	0.0140 (*↓)	0.6787±0.0657	0.0001 (***↓)
MambaMIL	0.9075±0.0374	0.0519 (ns↓)	0.8062±0.0694	0.0003 (***↓)	0.7841±0.0799	0.0304 (*↓)	0.7357±0.0926	0.0030 (**↓)	0.9271±0.0269	0.0978 (ns↓)	0.7373±0.0804	0.0041 (**↓)
MambaMIL+HiLA-MIL (Ours)	0.9338±0.0485	N/A	0.8360±0.0682	N/A	0.8276±0.0815	N/A	0.8347±0.0826	N/A	0.9387±0.0251	N/A	0.8181±0.0851	N/A

Methods	Negative											
	AUC	P-value	Accuracy	P-value	Precision	P-value	Recall	P-value	Specificity	P-value	F1-Score	P-value
ABMIL	0.9379±0.0451	0.7754 (ns↓)	0.9005±0.0564	0.2976 (ns↓)	0.7714±0.1193	0.1418 (ns↓)	0.8333±0.1546	1.0000 (ns↑)	0.9216±0.0413	0.0954 (ns↓)	0.7972±0.1244	0.2687 (ns↓)
CLAM-SB	0.9471±0.0334	0.5105 (ns↑)	0.9080±0.0372	0.5890 (ns↓)	0.7916±0.0706	0.3506 (ns↓)	0.8322±0.1261	1.0000 (ns↑)	0.9313±0.0242	0.5069 (ns↓)	0.8087±0.0869	0.5000 (ns↓)
CLAM-MB	0.9505±0.0408	0.5983 (ns↑)	0.9154±0.0376	1.0000 (ns↓)	0.8093±0.1002	0.4758 (ns↓)	0.8622±0.1353	0.1141 (ns↑)	0.9311±0.0458	0.5289 (ns↓)	0.8274±0.0879	0.9997 (ns↑)
DSMIL	0.9410±0.0385	0.5704 (ns↑)	0.9056±0.0363	0.4612 (ns↓)	0.7985±0.0773	0.4114 (ns↓)	0.8111±0.1106	0.3486 (ns↓)	0.9347±0.0303	0.6539 (ns↓)	0.8015±0.0802	0.3269 (ns↓)
TransMIL	0.9434±0.0418	0.7663 (ns↑)	0.8955±0.0350	0.1027 (ns↓)	0.7776±0.1080	0.0591 (ns↓)	0.8100±0.1343	0.2866 (ns↓)	0.9215±0.0463	0.0483 (*↓)	0.7842±0.0820	0.0706 (ns↓)
MambaMIL	0.9359±0.0442	0.5238 (ns↓)	0.9105±0.0499	0.6950 (ns↓)	0.8310±0.1235	0.9535 (ns↓)	0.7922±0.1482	0.1112 (ns↓)	0.9478±0.0382	1.0000 (ns↑)	0.8050±0.1155	0.4264 (ns↓)
MambaMIL+HiLA-MIL (Ours)	0.9388±0.0538	N/A	0.9180±0.0470	N/A	0.8496±0.1407	N/A	0.8311±0.1565	N/A	0.9444±0.0601	N/A	0.8256±0.1117	N/A

Methods	ITC											
	AUC	P-value	Accuracy	P-value	Precision	P-value	Recall	P-value	Specificity	P-value	F1-Score	P-value
ABMIL	0.9178±0.1227	0.0884 (ns↓)	0.9602±0.0241	0.0004 (***↓)	0.7000±0.3583	0.0209 (*↓)	0.6333±0.3496	<0.0001 (***↓)	0.9789±0.0242	0.1733 (ns↓)	0.6033±0.2650	0.0004 (***↓)
CLAM-SB	0.9512±0.0817	0.2365 (ns↓)	0.9627±0.0175	0.0007 (***↓)	0.6167±0.3932	0.0806 (ns↓)	0.5333±0.3752	<0.0001 (***↓)	0.9869±0.0186	0.6855 (ns↓)	0.5300±0.3187	0.0007 (***↓)
CLAM-MB	0.9009±0.1295	0.0420 (*↓)	0.9528±0.0138	<0.0001 (***↓)	0.4167±0.3263	0.0012 (**↓)	0.4333±0.3702	<0.0001 (***↓)	0.9816±0.0178	0.0303 (*↓)	0.4033±0.3004	<0.0001 (***↓)

DSMIL	0.9192±0.1034	0.0757 (ns↓)	0.9602±0.0173	0.0003 (***↓)	0.6000±0.3944	0.0445 (*↓)	0.5333±0.3752	<0.0001 (***↓)	0.9843±0.0222	0.4509 (ns↓)	0.5167±0.3088	0.0003 (***↓)
TransMIL	0.9022±0.1330	0.0165 (*↓)	0.9578±0.0202	0.0003 (***↓)	0.5833±0.4025	0.0113 (*↓)	0.5333±0.3752	<0.0001 (***↓)	0.9817±0.0248	0.0697 (ns↓)	0.5067±0.3110	0.0003 (***↓)
MambaMIL	0.9217±0.0801	0.0753 (ns↓)	0.9652±0.0210	0.0128 (*↓)	0.8000±0.2699	0.0656 (ns↓)	0.6333±0.2582	<0.0001 (***↓)	0.9842±0.0222	0.4443 (ns↓)	0.6533±0.1679	0.0012 (**↓)
MambaMIL+HiLA-MIL (Ours)	0.9737±0.0656	N/A	0.9901±0.0172	N/A	0.9000±0.1610	N/A	0.9667±0.1054	N/A	0.9921±0.0127	N/A	0.9267±0.1235	N/A

Methods	Micro											
	AUC	P-value	Accuracy	P-value	Precision	P-value	Recall	P-value	Specificity	P-value	F1-Score	P-value
ABMIL	0.8443±0.1040	0.2119 (ns↓)	0.8657±0.0488	0.5243 (ns↓)	0.6589±0.1904	0.8805 (ns↑)	0.5286±0.1656	0.0082 (**↓)	0.9367±0.0414	0.1486 (ns↑)	0.5746±0.1545	0.1543 (ns↓)
CLAM-SB	0.8511±0.1160	0.1581 (ns↓)	0.8632±0.0591	0.6026 (ns↓)	0.6447±0.2208	0.7954 (ns↓)	0.5571±0.1838	0.0237 (*↓)	0.9278±0.0475	0.3678 (ns↑)	0.5853±0.1725	0.1587 (ns↓)
CLAM-MB	0.8552±0.0850	0.3166 (ns↓)	0.8607±0.0515	0.3170 (ns↓)	0.6742±0.2142	0.5566 (ns↑)	0.5857±0.2376	0.1246 (ns↓)	0.9188±0.0835	1.0000 (ns↑)	0.5855±0.1420	0.2387 (ns↓)
DSMIL	0.8536±0.1069	0.1995 (ns↓)	0.8609±0.0574	0.3351 (ns↓)	0.6482±0.2053	0.5864 (ns↓)	0.5857±0.1710	0.1243 (ns↓)	0.9189±0.0598	1.0000 (ns↑)	0.5958±0.1385	0.2466 (ns↓)
TransMIL	0.8526±0.1126	0.0235 (*↓)	0.8434±0.0511	0.0413 (*↓)	0.6006±0.2026	0.0831 (ns↓)	0.5429±0.2108	0.0250 (*↓)	0.9068±0.0623	0.5407 (ns↓)	0.5373±0.1431	0.0291 (*↓)
MambaMIL	0.8336±0.1107	0.0665 (ns↓)	0.8559±0.0579	0.3086 (ns↓)	0.6174±0.2030	0.2147 (ns↓)	0.6286±0.1928	0.3805 (ns↓)	0.9041±0.0733	0.4086 (ns↓)	0.6041±0.1456	0.2860 (ns↓)
MambaMIL+HiLA-MIL (Ours)	0.8881±0.0917	N/A	0.8732±0.0556	N/A	0.6495±0.1897	N/A	0.6571±0.2626	N/A	0.9185±0.0686	N/A	0.6269±0.2015	N/A

Methods	Macro											
	AUC	P-value	Accuracy	P-value	Precision	P-value	Recall	P-value	Specificity	P-value	F1-Score	P-value
ABMIL	0.9387±0.0459	0.3519 (ns↑)	0.9055±0.0495	0.4235 (ns↑)	0.9069±0.0632	0.7993 (ns↓)	0.9208±0.0386	0.0400 (*↑)	0.8882±0.0829	0.4852 (ns↓)	0.9129±0.0438	0.2625 (ns↑)
CLAM-SB	0.9356±0.0486	0.6563 (ns↑)	0.8931±0.0588	1.0000 (ns↑)	0.8880±0.0535	0.2041 (ns↓)	0.9165±0.0677	0.0380 (*↑)	0.8668±0.0679	0.0755 (ns↓)	0.9012±0.0535	0.7006 (ns↑)
CLAM-MB	0.9308±0.0465	0.3038 (ns↓)	0.8880±0.0543	0.7109 (ns↓)	0.8994±0.0425	0.4956 (ns↓)	0.8935±0.1034	0.7904 (ns↑)	0.8824±0.0555	0.3137 (ns↓)	0.8928±0.0564	0.9185 (ns↓)
DSMIL	0.9294±0.0377	0.5618 (ns↓)	0.8956±0.0434	0.8669 (ns↑)	0.8958±0.0439	0.4701 (ns↓)	0.9115±0.0639	0.0993 (ns↑)	0.8777±0.0556	0.2696 (ns↓)	0.9024±0.0424	0.6318 (ns↑)
TransMIL	0.9277±0.0436	0.5620 (ns↓)	0.8808±0.0517	0.3946 (ns↓)	0.8931±0.0492	0.2794 (ns↓)	0.8835±0.0772	1.0000 (ns↓)	0.8777±0.0612	0.1790 (ns↓)	0.8867±0.0520	0.5829 (ns↓)
MambaMIL	0.9389±0.0408	0.3330 (ns↑)	0.8808±0.0666	0.4314 (ns↓)	0.8881±0.0531	0.2633 (ns↓)	0.8887±0.0915	1.0000 (ns↑)	0.8721±0.0629	0.1687 (ns↓)	0.8868±0.0667	0.6250 (ns↓)
MambaMIL+HiLA-MIL (Ours)	0.9346±0.0616	N/A	0.8907±0.0623	N/A	0.9114±0.0560	N/A	0.8838±0.1098	N/A	0.8999±0.0606	N/A	0.8933±0.0660	N/A

*：P<0.05；**：P<0.01；***：P<0.001；ns：no statistical significance.

↑: an increase in metrics of the baseline models compared with the MambaMIL+HiLA-MIL model.

↓: a decrease in metrics of the baseline models compared with the MambaMIL+HiLA-MIL model.

N/A: Not applicable. Statistical comparison was not performed.

A two-sided paired permutation test with 10,000 permutations was applied.

Supplementary Table 2. Evaluation metrics of overall four-class classification and each LNM category across four feature extractors on YDSY Dataset

ResNet

Methods	Overall											
	AUC	P-value	Accuracy	P-value	Precision	P-value	Recall	P-value	Specificity	P-value	F1-Score	P-value
ABMIL	0.8765±0.0668	0.3078 (ns↓)	0.7077±0.0638	<0.0001 (***↓)	0.6745±0.0814	<0.0001 (***↓)	0.6498±0.1159	<0.0001 (***↓)	0.8948±0.0234	0.0001 (***↓)	0.6349±0.1085	<0.0001 (***↓)
CLAM-SB	0.8751±0.0603	0.7136 (ns↓)	0.7075±0.0943	<0.0001 (***↓)	0.6825±0.0749	<0.0001 (***↓)	0.6470±0.1426	0.0001 (***↓)	0.8921±0.0359	0.0001 (***↓)	0.6369±0.1229	<0.0001 (***↓)
CLAM-MB	0.8779±0.0623	0.0620 (ns↓)	0.7340±0.0792	<0.0001 (***↓)	0.7183±0.0783	0.0006 (***↓)	0.6681±0.1272	0.0006 (***↓)	0.9026±0.0321	0.0030 (**↓)	0.6532±0.1090	0.0003 (***↓)
DSMIL	0.8861±0.0588	0.1846 (ns↓)	0.7342±0.0802	<0.0001 (***↓)	0.7189±0.0905	0.0016 (**↓)	0.6702±0.1406	0.0011 (**↓)	0.9010±0.0406	0.0040 (**↓)	0.6576±0.1193	0.0004 (***↓)
TransMIL	0.8736±0.0727	0.1574 (ns↓)	0.7106±0.0924	<0.0001 (***↓)	0.6878±0.1196	0.0001 (***↓)	0.6485±0.1397	<0.0001 (***↓)	0.8961±0.0357	<0.0001 (***↓)	0.6526±0.1265	<0.0001 (***↓)
MambaMIL	0.8858±0.0512	0.0259 (*↓)	0.7206±0.0642	<0.0001 (***↓)	0.6748±0.1053	0.0036 (**↓)	0.6641±0.1144	0.0001 (***↓)	0.8987±0.0290	0.0002 (***↓)	0.6509±0.1041	0.0001 (***↓)
MambaMIL+HiLA-MIL (Ours)	0.9040±0.0431	N/A	0.7938±0.0546	N/A	0.7734±0.1448	N/A	0.7535±0.1062	N/A	0.9238±0.0204	N/A	0.7466±0.1157	N/A

Methods	Negative											
	AUC	P-value	Accuracy	P-value	Precision	P-value	Recall	P-value	Specificity	P-value	F1-Score	P-value
ABMIL	0.9089±0.0667	0.6258 (ns↓)	0.8505±0.0670	0.0058 (**↓)	0.6892±0.1529	0.0174 (*↓)	0.7625±0.1597	0.0325 (*↓)	0.8778±0.0867	0.1461 (ns↓)	0.7084±0.1196	0.0117 (*↓)
CLAM-SB	0.9097±0.0570	0.8029 (ns↓)	0.8537±0.0851	0.0440 (*↓)	0.6903±0.1812	0.0552 (ns↓)	0.7679±0.2029	0.0334 (*↓)	0.8824±0.0895	0.1187 (ns↓)	0.7115±0.1766	0.0207 (*↓)
CLAM-MB	0.9173±0.0636	0.2341 (ns↓)	0.8569±0.0632	0.0310 (*↓)	0.6783±0.1174	0.0438 (*↓)	0.8089±0.2034	0.1779 (ns↓)	0.8739±0.0723	0.0496 (*↓)	0.7228±0.1285	0.0612 (ns↓)
DSMIL	0.9168±0.0565	0.5213 (ns↓)	0.8605±0.0748	0.0188 (*↓)	0.7026±0.1541	0.0741 (ns↓)	0.7643±0.2131	0.0223 (*↓)	0.8911±0.0804	0.1792 (ns↓)	0.7181±0.1537	0.0369 (*↓)
TransMIL	0.9148±0.0646	0.7146 (ns↓)	0.8503±0.0727	0.0038 (**↓)	0.6853±0.1678	0.0259 (*↓)	0.7339±0.1795	0.0084 (**↓)	0.8867±0.0690	0.1093 (ns↓)	0.6986±0.1419	0.0077 (**↓)
MambaMIL	0.9222±0.0417	0.6516 (ns↑)	0.8437±0.0502	0.0042 (**↓)	0.6490±0.1016	0.0030 (**↓)	0.8196±0.1425	0.2693 (ns↓)	0.8518±0.0746	0.0023 (**↓)	0.7135±0.0718	0.0192 (*↓)
MambaMIL+HiLA-MIL (Ours)	0.9195±0.0575	N/A	0.8934±0.0626	N/A	0.7595±0.1613	N/A	0.8571±0.1650	N/A	0.9037±0.0716	N/A	0.7931±0.1247	N/A

Methods	ITC											
	AUC	P-value	Accuracy	P-value	Precision	P-value	Recall	P-value	Specificity	P-value	F1-Score	P-value
ABMIL	0.9088±0.1437	0.3841 (ns↓)	0.9502±0.0359	0.0016 (**↓)	0.6900±0.3304	0.0075 (**↓)	0.6833±0.3282	<0.0001 (***↓)	0.9713±0.0329	0.0075 (**↓)	0.6538±0.2828	0.0016 (**↓)
CLAM-SB	0.9058±0.1408	0.0853 (ns↓)	0.9535±0.0357	0.0038 (**↓)	0.7400±0.3362	0.0162 (*↓)	0.6833±0.3282	<0.0001 (***↓)	0.9749±0.0339	0.0162 (*↓)	0.6705±0.2776	0.0038 (**↓)
CLAM-MB	0.9116±0.1096	0.6662 (ns↓)	0.9535±0.0357	0.0038 (**↓)	0.7400±0.3362	0.0162 (*↓)	0.6833±0.3282	<0.0001 (***↓)	0.9749±0.0339	0.0162 (*↓)	0.6705±0.2776	0.0038 (**↓)
DSMIL	0.9302±0.0934	0.7447 (ns↓)	0.9502±0.0422	0.0022 (**↓)	0.7400±0.3362	0.0084 (**↓)	0.6833±0.3282	<0.0001 (***↓)	0.9713±0.0369	0.0084 (**↓)	0.6705±0.2776	0.0022 (**↓)
TransMIL	0.9050±0.1593	0.2243 (ns↓)	0.9602±0.0304	0.0146 (*↓)	0.8000±0.3220	0.1254 (ns↓)	0.6333±0.3123	<0.0001 (***↓)	0.9856±0.0186	0.1254 (ns↓)	0.6800±0.2754	0.0146 (*↓)
MambaMIL	0.9278±0.1320	0.6993 (ns↓)	0.9669±0.0269	0.4518 (ns↓)	0.8167±0.3375	0.3660 (ns↓)	0.6833±0.3282	<0.0001 (***↓)	0.9893±0.0241	1.0000 (ns↓)	0.7133±0.2932	0.1242 (ns↓)

MambaMIL+HiLA-MIL (Ours)	0.9403±0.0934	N/A	0.9768±0.0316	N/A	0.8667±0.3220	N/A	0.7833±0.3338	N/A	0.9929±0.0151	N/A	0.8067±0.3086	N/A

Methods	Micro											
	AUC	P-value	Accuracy	P-value	Precision	P-value	Recall	P-value	Specificity	P-value	F1-Score	P-value
ABMIL	0.7572±0.1204	0.1204 (ns↓)	0.7709±0.0613	0.0008 (***↓)	0.4688±0.3039	0.0008 (***↓)	0.3133±0.1847	0.0008 (***↓)	0.8813±0.0775	0.0480 (*↓)	0.3361±0.1490	0.0006 (***↓)
CLAM-SB	0.7585±0.1307	0.1100 (ns↓)	0.7741±0.0843	0.0016 (**↓)	0.4683±0.2679	0.0021 (**↓)	0.2900±0.1963	0.0006 (***↓)	0.8893±0.0911	0.1061 (ns↓)	0.3308±0.1777	0.0006 (***↓)
CLAM-MB	0.7494±0.1418	0.0567 (ns↓)	0.7972±0.0764	0.0363 (*↓)	0.5955±0.3370	0.0828 (ns↑)	0.3067±0.2351	0.0019 (**↓)	0.9137±0.0974	0.6940 (ns↓)	0.3576±0.1959	0.0064 (**↓)
DSMIL	0.7654±0.1170	0.0665 (ns↓)	0.8073±0.0516	0.1048 (ns↓)	0.5755±0.3056	0.1852 (ns↓)	0.3600±0.2624	0.0126 (*↓)	0.9138±0.0792	0.6785 (ns↓)	0.3860±0.2046	0.0335 (*↓)
TransMIL	0.7485±0.1232	0.0128 (*↓)	0.7639±0.0699	<0.0001 (***↓)	0.4050±0.2094	<0.0001 (***↓)	0.3933±0.2171	0.0097 (**↓)	0.8512±0.0656	0.0007 (***↓)	0.3874±0.1856	0.0003 (***↓)
MambaMIL	0.7650±0.1030	0.0034 (**↓)	0.7839±0.0401	0.0051 (**↓)	0.3700±0.2202	0.0141 (*↓)	0.3200±0.2394	0.0004 (***↓)	0.8930±0.0592	0.1673 (ns↓)	0.3319±0.2042	0.0008 (***↓)
MambaMIL+HiLA-MIL (Ours)	0.8240±0.0908	N/A	0.8370±0.0434	N/A	0.5859±0.2878	N/A	0.4933±0.2314	N/A	0.9172±0.0705	N/A	0.5077±0.2033	N/A

Methods	Macro											
	AUC	P-value	Accuracy	P-value	Precision	P-value	Recall	P-value	Specificity	P-value	F1-Score	P-value
ABMIL	0.9309±0.0646	0.2866 (ns↓)	0.8439±0.0545	0.0068 (**↓)	0.8502±0.0649	0.0387 (*↓)	0.8400±0.1004	0.0411 (*↓)	0.8487±0.0786	0.0369 (*↓)	0.8413±0.0602	0.0144 (*↓)
CLAM-SB	0.9262±0.0550	0.3602 (ns↓)	0.8338±0.0650	0.0002 (***↓)	0.8316±0.0780	0.0010 (**↓)	0.8467±0.0996	0.0395 (*↓)	0.8217±0.0981	0.0010 (**↓)	0.8346±0.0634	0.0019 (**↓)
CLAM-MB	0.9333±0.0556	0.8022 (ns↑)	0.8603±0.0683	0.0792 (ns↓)	0.8595±0.0892	0.0976 (ns↓)	0.8733±0.0914	0.3741 (ns↓)	0.8479±0.1086	0.0645 (ns↓)	0.8619±0.0628	0.1375 (ns↓)
DSMIL	0.9321±0.0635	0.0883 (ns↑)	0.8503±0.0999	0.0505 (ns↓)	0.8574±0.1317	0.0492 (*↓)	0.8733±0.1109	0.5054 (ns↓)	0.8279±0.1863	0.0324 (*↓)	0.8558±0.0861	0.0965 (ns↓)
TransMIL	0.9262±0.0575	0.0421 (*↓)	0.8469±0.0822	0.0074 (**↓)	0.8610±0.0907	0.1289 (ns↓)	0.8333±0.0956	0.0118 (*↓)	0.8608±0.1017	0.1304 (ns↓)	0.8445±0.0793	0.0101 (*↓)
MambaMIL	0.9283±0.0442	0.0823 (ns↓)	0.8469±0.0743	0.0038 (**↓)	0.8636±0.0925	0.1243 (ns↓)	0.8333±0.0846	0.0042 (**↓)	0.8608±0.1064	0.1257 (ns↓)	0.8450±0.0689	0.0045 (**↓)
MambaMIL+HiLA-MIL (Ours)	0.9320±0.0508	N/A	0.8803±0.0634	N/A	0.8817±0.0642	N/A	0.8800±0.0878	N/A	0.8812±0.0673	N/A	0.8791±0.0645	N/A

Supplementary Table 2. Continued

UNI

Methods	Overall											
	AUC	P-value	Accuracy	P-value	Precision	P-value	Recall	P-value	Specificity	P-value	F1-Score	P-value
ABMIL	0.9255±0.0142	0.1271 (ns↓)	0.8370±0.0731	0.0012 (**↓)	0.8400±0.0873	0.2130 (ns↓)	0.7977±0.0886	0.1202 (ns↓)	0.9394±0.0241	0.2260 (ns↓)	0.7927±0.0889	0.1167 (ns↓)
CLAM-SB	0.9222±0.0287	0.1493 (ns↓)	0.8074±0.0335	<0.0001 (***↓)	0.7926±0.1008	0.0085 (**↓)	0.7493±0.0659	0.0007 (***↓)	0.9261±0.0129	0.0014 (**↓)	0.7380±0.0666	0.0010 (**↓)
CLAM-MB	0.9168±0.0280	0.0782 (ns↓)	0.8206±0.0388	<0.0001 (***↓)	0.8061±0.0814	0.0559 (ns↓)	0.7674±0.0694	0.0051 (**↓)	0.9295±0.0168	0.0120 (*↓)	0.7599±0.0689	0.0063 (**↓)
DSMIL	0.9243±0.0222	0.0909 (ns↓)	0.8405±0.0262	0.0013 (**↓)	0.8342±0.0925	0.2476 (ns↓)	0.7870±0.0684	0.0533 (ns↓)	0.9387±0.0154	0.1858 (ns↓)	0.7792±0.0703	0.0630 (ns↓)
TransMIL	0.9252±0.0320	0.1465 (ns↓)	0.8106±0.0472	<0.0001 (***↓)	0.7941±0.0889	0.0425 (*↓)	0.7624±0.0616	0.0025 (**↓)	0.9262±0.0209	0.0028 (**↓)	0.7553±0.0679	0.0039 (**↓)

MambaMIL	0.9212±0.0304	0.0984 (ns↓)	0.8140±0.0320	<0.0001 (***↓)	0.7974±0.0890	0.0305 (*↓)	0.7640±0.0717	0.0029 (**↓)	0.9280±0.0164	0.0073 (**↓)	0.7525±0.0698	0.0040 (**↓)
MambaMIL+HiLA-MIL (Ours)	0.9352±0.0433	N/A	0.8672±0.0563	N/A	0.8807±0.0946	N/A	0.8330±0.0871	N/A	0.9484±0.0190	N/A	0.8414±0.0914	N/A

Methods	Negative											
	AUC	P-value	Accuracy	P-value	Precision	P-value	Recall	P-value	Specificity	P-value	F1-Score	P-value
ABMIL	0.9808±0.0222	0.8989 (ns↑)	0.9500±0.0550	0.8154 (ns↑)	0.9010±0.1142	0.3453 (ns↑)	0.9143±0.2254	0.5076 (ns↓)	0.9605±0.0483	0.5055 (ns↑)	0.8822±0.1680	0.7077 (ns↓)
CLAM-SB	0.9814±0.0231	0.6707 (ns↑)	0.9304±0.0526	0.2390 (ns↓)	0.8249±0.1353	0.2330 (ns↓)	0.9304±0.1379	1.0000 (ns↑)	0.9302±0.0624	0.1510 (ns↓)	0.8647±0.1090	0.3795 (ns↓)
CLAM-MB	0.9771±0.0238	0.6251 (ns↑)	0.9404±0.0459	1.0000 (ns↓)	0.8413±0.1189	0.5548 (ns↓)	0.9429±0.1380	0.3702 (ns↑)	0.9389±0.0513	0.7598 (ns↓)	0.8810±0.1031	0.8286 (ns↓)
DSMIL	0.9801±0.0268	0.9666 (ns↑)	0.9470±0.0354	1.0000 (ns↑)	0.8418±0.0979	0.6758 (ns↓)	0.9714±0.0602	0.0644 (ns↑)	0.9389±0.0429	0.7525 (ns↓)	0.8991±0.0661	0.6806 (ns↑)
TransMIL	0.9793±0.0221	0.4015 (ns↑)	0.9337±0.0348	0.6037 (ns↓)	0.8172±0.0891	0.2259 (ns↓)	0.9429±0.1205	0.3730 (ns↑)	0.9302±0.0423	0.1118 (ns↓)	0.8691±0.0745	0.5174 (ns↓)
MambaMIL	0.9774±0.0223	0.4790 (ns↑)	0.9371±0.0421	0.8071 (ns↓)	0.8288±0.1062	0.3941 (ns↓)	0.9429±0.1380	0.3702 (ns↑)	0.9346±0.0473	0.5556 (ns↓)	0.8743±0.0965	0.6764 (ns↓)
MambaMIL+HiLA-MIL (Ours)	0.9724±0.0417	N/A	0.9435±0.0417	N/A	0.8538±0.1013	N/A	0.9286±0.0753	N/A	0.9476±0.0404	N/A	0.8877±0.0809	N/A

Methods	ITC											
	AUC	P-value	Accuracy	P-value	Precision	P-value	Recall	P-value	Specificity	P-value	F1-Score	P-value
ABMIL	0.9403±0.0905	0.2572 (ns↓)	0.9735±0.0259	1.0000 (ns↓)	0.8333±0.3239	1.0000 (ns↓)	0.7333±0.3352	<0.0001 (***↓)	0.9929±0.0151	1.0000 (ns↓)	0.7600±0.3050	0.3770 (ns↓)
CLAM-SB	0.9349±0.1277	0.4017 (ns↓)	0.9602±0.0261	0.0409 (*↓)	0.7500±0.3263	0.2258 (ns↓)	0.6500±0.3465	<0.0001 (***↓)	0.9857±0.0184	0.2258 (ns↓)	0.6600±0.2901	0.0409 (*↓)
CLAM-MB	0.9326±0.1276	0.2870 (ns↓)	0.9669±0.0312	0.4561 (ns↓)	0.7833±0.3338	0.3774 (ns↓)	0.7000±0.3583	<0.0001 (***↓)	0.9893±0.0173	1.0000 (ns↓)	0.7133±0.3233	0.1292 (ns↓)
DSMIL	0.9386±0.0860	0.1843 (ns↓)	0.9702±0.0289	0.6879 (ns↓)	0.8333±0.3239	0.8127 (ns↓)	0.7000±0.3583	<0.0001 (***↓)	0.9929±0.0151	1.0000 (ns↓)	0.7300±0.3152	0.2230 (ns↓)
TransMIL	0.9362±0.1188	0.2261 (ns↓)	0.9702±0.0329	0.6892 (ns↓)	0.8167±0.3375	0.8127 (ns↓)	0.7000±0.3583	<0.0001 (***↓)	0.9929±0.0151	1.0000 (ns↓)	0.7333±0.3352	0.2265 (ns↓)
MambaMIL	0.9344±0.1248	0.3016 (ns↓)	0.9669±0.0312	0.4561 (ns↓)	0.7833±0.3338	0.3774 (ns↓)	0.7000±0.3583	<0.0001 (***↓)	0.9893±0.0173	1.0000 (ns↓)	0.7133±0.3233	0.1292 (ns↓)
MambaMIL+HiLA-MIL (Ours)	0.9589±0.0663	N/A	0.9768±0.0316	N/A	0.9333±0.2108	N/A	0.7833±0.2364	N/A	0.9929±0.0226	N/A	0.8333±0.2067	N/A

Methods	Micro											
	AUC	P-value	Accuracy	P-value	Precision	P-value	Recall	P-value	Specificity	P-value	F1-Score	P-value
ABMIL	0.8510±0.0808	0.3337 (ns↓)	0.8603±0.0784	0.0031 (**↓)	0.7251±0.2199	0.0026 (**↓)	0.6567±0.2206	0.2320 (ns↓)	0.9108±0.1060	0.0026 (**↓)	0.6398±0.1521	0.0157 (*↓)
CLAM-SB	0.8559±0.0893	0.3521 (ns↓)	0.8572±0.0414	0.0004 (***↓)	0.7272±0.1844	0.0029 (**↓)	0.5367±0.2613	0.0016 (**↓)	0.9347±0.0582	0.0200 (*↓)	0.5586±0.1888	0.0008 (***↓)
CLAM-MB	0.8426±0.0985	0.2143 (ns↓)	0.8638±0.0484	0.0034 (**↓)	0.7375±0.2257	0.0394 (*↓)	0.5333±0.2611	0.0023 (**↓)	0.9428±0.0553	0.3823 (ns↓)	0.5726±0.2034	0.0034 (**↓)
DSMIL	0.8605±0.0869	0.3921 (ns↓)	0.8703±0.0335	0.0039 (**↓)	0.7669±0.1816	0.0304 (*↓)	0.5700±0.2769	0.0143 (*↓)	0.9425±0.0486	0.2903 (ns↓)	0.5922±0.1906	0.0078 (**↓)
TransMIL	0.8561±0.0982	0.5135 (ns↓)	0.8571±0.0630	0.0009 (***↓)	0.6842±0.2596	0.0066 (**↓)	0.5333±0.2944	0.0049 (**↓)	0.9347±0.0475	0.0357 (*↓)	0.5566±0.2449	0.0020 (**↓)

MambaMIL	0.8523±0.0966	0.2516 (ns↓)	0.8571±0.0447	0.0009 (***↓)	0.7108±0.2109	0.0131 (*↓)	0.5333±0.2726	0.0021 (**↓)	0.9345±0.0519	0.0561 (ns↓)	0.5540±0.2115	0.0013 (**↓)
MambaMIL+HiLA-MIL (Ours)	0.8722±0.0876	N/A	0.9103±0.0416	N/A	0.8363±0.1580	N/A	0.7067±0.2095	N/A	0.9590±0.0432	N/A	0.7404±0.1377	N/A

Methods	Macro											
	AUC	P-value	Accuracy	P-value	Precision	P-value	Recall	P-value	Specificity	P-value	F1-Score	P-value
ABMIL	0.9298±0.0390	0.3631 (ns↓)	0.8901±0.0475	0.3261 (ns↓)	0.9006±0.0723	0.8747 (ns↑)	0.8867±0.0945	0.1400 (ns↓)	0.8933±0.0900	1.0000 (ns↓)	0.8886±0.0532	0.3510 (ns↓)
CLAM-SB	0.9163±0.0350	0.1657 (ns↓)	0.8670±0.0388	0.0072 (**↓)	0.8681±0.0795	0.0426 (*↓)	0.8800±0.0820	0.0389 (*↓)	0.8538±0.1083	0.0403 (*↓)	0.8685±0.0372	0.0132 (*↓)
CLAM-MB	0.9150±0.0378	0.1767 (ns↓)	0.8702±0.0486	0.0316 (*↓)	0.8624±0.0789	0.0462 (*↓)	0.8933±0.0900	0.2242 (ns↓)	0.8471±0.1000	0.0367 (*↓)	0.8726±0.0485	0.0574 (ns↓)
DSMIL	0.9178±0.0343	0.1587 (ns↓)	0.8935±0.0412	0.3572 (ns↓)	0.8946±0.0843	0.4787 (ns↓)	0.9067±0.0717	0.4578 (ns↓)	0.8804±0.1127	0.3955 (ns↓)	0.8956±0.0373	0.4542 (ns↓)
TransMIL	0.9292±0.0430	0.6087 (ns↓)	0.8603±0.0543	0.0044 (**↓)	0.8583±0.0825	0.0382 (*↓)	0.8733±0.0798	0.0220 (*↓)	0.8471±0.1000	0.0376 (*↓)	0.8620±0.0536	0.0064 (**↓)
MambaMIL	0.9206±0.0374	0.2462 (ns↓)	0.8669±0.0448	0.0211 (*↓)	0.8666±0.0787	0.0636 (ns↓)	0.8800±0.0820	0.0890 (ns↓)	0.8538±0.1036	0.0586 (ns↓)	0.8684±0.0438	0.0338 (*↓)
MambaMIL+HiLA-MIL (Ours)	0.9371±0.0352	N/A	0.9038±0.0326	N/A	0.8993±0.0530	N/A	0.9133±0.0632	N/A	0.8942±0.0641	N/A	0.9041±0.0343	N/A

Supplementary Table 2. Continued

GigaPath

Methods	Overall											
	AUC	P-value	Accuracy	P-value	Precision	P-value	Recall	P-value	Specificity	P-value	F1-Score	P-value
ABMIL	0.9179±0.0372	0.0733 (ns↓)	0.8105±0.0525	<0.0001 (***↓)	0.7925±0.0963	0.0966 (ns↓)	0.7601±0.0881	0.0048 (**↓)	0.9291±0.0201	0.0383 (*↓)	0.7596±0.0923	0.0089 (**↓)
CLAM-SB	0.9246±0.0433	0.2919 (ns↓)	0.8139±0.0528	0.0005 (***↓)	0.8000±0.1008	0.0903 (ns↓)	0.7580±0.0869	0.0055 (**↓)	0.9302±0.0207	0.0704 (ns↓)	0.7555±0.0903	0.0117 (*↓)
CLAM-MB	0.9166±0.0445	0.0335 (*↓)	0.8139±0.0615	<0.0001 (***↓)	0.7704±0.1204	0.1496 (ns↓)	0.7560±0.1019	0.0069 (**↓)	0.9325±0.0214	0.0816 (ns↓)	0.7477±0.1127	0.0105 (*↓)
DSMIL	0.9222±0.0410	0.1073 (ns↓)	0.8040±0.0532	<0.0001 (***↓)	0.7949±0.1002	0.0021 (**↓)	0.7518±0.0872	0.0006 (***↓)	0.9262±0.0160	0.0093 (**↓)	0.7546±0.0945	0.0007 (***↓)
TransMIL	0.9368±0.0356	0.6823 (ns↓)	0.8238±0.0526	0.0031 (**↓)	0.8066±0.0965	0.0184 (*↓)	0.7751±0.0958	0.0155 (*↓)	0.9337±0.0183	0.1393 (ns↓)	0.7723±0.0957	0.0116 (*↓)
MambaMIL	0.9213±0.0369	0.0854 (ns↓)	0.7874±0.0610	<0.0001 (***↓)	0.7781±0.1034	0.0001 (***↓)	0.7414±0.0878	0.0002 (***↓)	0.9241±0.0205	0.0045 (**↓)	0.7377±0.0936	0.0001 (***↓)
MambaMIL+HiLA-MIL (Ours)	0.9424±0.0306	N/A	0.8471±0.0594	N/A	0.8566±0.0859	N/A	0.8228±0.0904	N/A	0.9442±0.0185	N/A	0.8229±0.0868	N/A

Methods	Negative											
	AUC	P-value	Accuracy	P-value	Precision	P-value	Recall	P-value	Specificity	P-value	F1-Score	P-value
ABMIL	0.9771±0.0239	0.5566 (ns↓)	0.9403±0.0340	1.0000 (ns↓)	0.8264±0.0847	0.3773 (ns↓)	0.9571±0.0964	0.2493 (ns↑)	0.9346±0.0374	0.6252 (ns↓)	0.8832±0.0706	0.7960 (ns↓)
CLAM-SB	0.9825±0.0199	0.8512 (ns↑)	0.9337±0.0348	0.5695 (ns↓)	0.8323±0.1120	0.3168 (ns↓)	0.9286±0.1010	0.4461 (ns↓)	0.9346±0.0515	0.6896 (ns↓)	0.8699±0.0675	0.3839 (ns↓)
CLAM-MB	0.9781±0.0256	0.4471 (ns↓)	0.9303±0.0455	0.1454 (ns↓)	0.8059±0.1126	0.1107 (ns↓)	0.9571±0.0964	0.2381 (ns↑)	0.9215±0.0574	0.0414 (*↓)	0.8689±0.0826	0.3313 (ns↓)
DSMIL	0.9795±0.0260	0.8513 (ns↓)	0.9435±0.0445	1.0000 (ns↓)	0.8494±0.1289	0.6853 (ns↑)	0.9571±0.0690	<0.0001 (***↓)	0.9389±0.0590	1.0000 (ns↓)	0.8937±0.0782	1.0000 (ns↑)

TransMIL	0.9854±0.0156	0.7515 (ns↑)	0.9403±0.0463	1.0000 (ns↓)	0.8325±0.1179	0.4570 (ns↓)	0.9571±0.0964	0.2472 (ns↑)	0.9346±0.0515	0.6898 (ns↓)	0.8855±0.0898	0.7971 (ns↓)
MambaMIL	0.9776±0.0215	0.1825 (ns↓)	0.9272±0.0481	0.0950 (ns↓)	0.8270±0.0989	0.5079 (ns↓)	0.8857±0.1881	0.0283 (*↓)	0.9389±0.0471	1.0000 (ns↓)	0.8438±0.1253	0.1504 (ns↓)
MambaMIL+HiLA-MIL (Ours)	0.9818±0.0177	N/A	0.9435±0.0386	N/A	0.8470±0.0996	N/A	0.9446±0.0717	N/A	0.9433±0.0417	N/A	0.8903±0.0725	N/A

Methods	ITC											
	AUC	P-value	Accuracy	P-value	Precision	P-value	Recall	P-value	Specificity	P-value	F1-Score	P-value
ABMIL	0.9348±0.0887	0.1025 (ns↓)	0.9702±0.0289	0.6324 (ns↓)	0.8667±0.3220	0.7584 (ns↓)	0.6500±0.3465	<0.0001 (***↓)	0.9964±0.0113	1.0000 (ns↓)	0.7167±0.3148	0.1243 (ns↓)
CLAM-SB	0.9343±0.0969	0.0238 (*↓)	0.9669±0.0269	0.3753 (ns↓)	0.8333±0.3239	0.2475 (ns↓)	0.6500±0.3465	<0.0001 (***↓)	0.9929±0.0151	1.0000 (ns↓)	0.6967±0.3008	0.0603 (ns↓)
CLAM-MB	0.9345±0.0935	0.1393 (ns↓)	0.9669±0.0312	0.3712 (ns↓)	0.7667±0.4172	0.6289 (ns↓)	0.6000±0.4022	<0.0001 (***↓)	0.9964±0.0113	1.0000 (ns↓)	0.6500±0.3885	0.0615 (ns↓)
DSMIL	0.9398±0.0804	0.2543 (ns↓)	0.9669±0.0349	0.2489 (ns↓)	0.8667±0.3220	<0.0001 (***↓)	0.6500±0.3465	<0.0001 (***↓)	0.9929±0.0151	1.0000 (ns↓)	0.7167±0.3148	<0.0001 (***↓)
TransMIL	0.9755±0.0373	0.7090 (ns↑)	0.9637±0.0393	<0.0001 (***↓)	0.8167±0.3375	<0.0001 (***↓)	0.7000±0.3583	<0.0001 (***↓)	0.9857±0.0250	<0.0001 (***↓)	0.7205±0.3188	<0.0001 (***↓)
MambaMIL	0.9446±0.0831	0.2574 (ns↓)	0.9637±0.0393	<0.0001 (***↓)	0.8500±0.3375	<0.0001 (***↓)	0.6500±0.3465	<0.0001 (***↓)	0.9893±0.0241	0.5029 (ns↓)	0.7071±0.3179	<0.0001 (***↓)
MambaMIL+HiLA-MIL (Ours)	0.9684±0.0666	N/A	0.9768±0.0316	N/A	0.9000±0.3162	N/A	0.7333±0.3352	N/A	0.9964±0.0113	N/A	0.7933±0.3118	N/A

Methods	Micro											
	AUC	P-value	Accuracy	P-value	Precision	P-value	Recall	P-value	Specificity	P-value	F1-Score	P-value
ABMIL	0.8388±0.0905	0.0956 (ns↓)	0.8438±0.0449	0.0033 (**↓)	0.5985±0.1334	0.0285 (*↓)	0.5800±0.2026	0.0003 (***↓)	0.9055±0.0471	0.2073 (ns↓)	0.5740±0.1577	0.0012 (**↓)
CLAM-SB	0.8470±0.0911	0.3851 (ns↓)	0.8504±0.0480	0.0205 (*↓)	0.6531±0.1752	0.1167 (ns↓)	0.5800±0.2310	0.0008 (***↓)	0.9135±0.0491	0.4954 (ns↓)	0.5794±0.1645	0.0055 (**↓)
CLAM-MB	0.8304±0.1024	0.0371 (*↓)	0.8504±0.0453	0.0096 (**↓)	0.6064±0.1309	0.0440 (*↓)	0.6133±0.2150	0.0014 (**↓)	0.9053±0.0437	0.2054 (ns↓)	0.5960±0.1661	0.0071 (**↓)
DSMIL	0.8479±0.0916	0.1786 (ns↓)	0.8371±0.0403	0.0008 (***↓)	0.5948±0.1341	0.0109 (*↓)	0.5467±0.1989	<0.0001 (***↓)	0.9052±0.0552	0.1786 (ns↓)	0.5499±0.1399	0.0001 (***↓)
TransMIL	0.8542±0.0912	0.5710 (ns↓)	0.8635±0.0511	0.0542 (ns↓)	0.6955±0.1864	0.5660 (ns↓)	0.5633±0.1856	0.0001 (***↓)	0.9343±0.0551	0.5017 (ns↑)	0.6044±0.1604	0.0210 (*↓)
MambaMIL	0.8396±0.0892	0.1260 (ns↓)	0.8172±0.0529	0.0001 (***↓)	0.5419±0.1362	0.0001 (***↓)	0.5967±0.2437	0.0012 (**↓)	0.8683±0.0746	0.0015 (**↓)	0.5402±0.1470	0.0001 (***↓)
MambaMIL+HiLA-MIL (Ours)	0.8780±0.0759	N/A	0.8902±0.0591	N/A	0.7649±0.2175	N/A	0.7600±0.1377	N/A	0.9223±0.0822	N/A	0.7331±0.1118	N/A

Methods	Macro											
	AUC	P-value	Accuracy	P-value	Precision	P-value	Recall	P-value	Specificity	P-value	F1-Score	P-value
ABMIL	0.9207±0.0475	0.6994 (ns↓)	0.8668±0.0590	0.4210 (ns↓)	0.8785±0.0655	0.1887 (ns↓)	0.8533±0.0757	1.0000 (ns↓)	0.8800±0.0689	0.1179 (ns↓)	0.8644±0.0618	0.4027 (ns↓)
CLAM-SB	0.9345±0.0376	0.2821 (ns↓)	0.8768±0.0547	0.8461 (ns↓)	0.8814±0.0645	0.2510 (ns↓)	0.8733±0.0663	0.2219 (ns↑)	0.8800±0.0689	0.1176 (ns↓)	0.8761±0.0561	0.8567 (ns↑)
CLAM-MB	0.9234±0.0502	0.8138 (ns↓)	0.8801±0.0528	1.0000 (ns↓)	0.9027±0.0546	0.8313 (ns↓)	0.8533±0.0757	1.0000 (ns↓)	0.9067±0.0562	1.0000 (ns↓)	0.8759±0.0567	0.9111 (ns↑)
DSMIL	0.9214±0.0401	0.5571 (ns↓)	0.8604±0.0345	0.0936 (ns↓)	0.8689±0.0505	0.0527 (ns↓)	0.8533±0.0757	1.0000 (ns↓)	0.8679±0.0615	0.0254 (*↓)	0.8581±0.0390	0.2059 (ns↓)

TransMIL	0.9320±0.0444	0.6900 (ns↓)	0.8800±0.0549	1.0000 (ns↓)	0.8819±0.0550	0.2840 (ns↓)	0.8800±0.0878	0.0316 (*↑)	0.8800±0.0613	0.1182 (ns↓)	0.8787±0.0590	0.9813 (ns↑)
MambaMIL	0.9235±0.0444	0.9713 (ns↓)	0.8668±0.0687	0.4067 (ns↓)	0.8936±0.0677	0.4989 (ns↓)	0.8333±0.1054	0.2228 (ns↓)	0.9000±0.0648	0.7793 (ns↓)	0.8599±0.0789	0.2569 (ns↓)
MambaMIL+HiLA-MIL (Ours)	0.9414±0.0338	N/A	0.8837±0.0551	N/A	0.9144±0.0539	N/A	0.8533±0.1398	N/A	0.9146±0.0608	N/A	0.8746±0.0720	N/A

Supplementary Table 2. Continued

Virchow

Methods	Overall											
	AUC	P-value	Accuracy	P-value	Precision	P-value	Recall	P-value	Specificity	P-value	F1-Score	P-value
ABMIL	0.8887±0.0578	0.0050 (**↓)	0.7738±0.0756	<0.0001 (***↓)	0.7536±0.0622	0.0006 (***↓)	0.7457±0.0939	0.0022 (**↓)	0.9179±0.0301	0.0134 (*↓)	0.7388±0.0805	0.0006 (***↓)
CLAM-SB	0.8949±0.0627	0.0036 (**↓)	0.7672±0.0851	<0.0001 (***↓)	0.7476±0.0716	0.0001 (***↓)	0.7346±0.1089	0.0013 (**↓)	0.9148±0.0341	0.0044 (**↓)	0.7296±0.0917	0.0002 (***↓)
CLAM-MB	0.8955±0.0587	0.0196 (*↓)	0.7739±0.0904	<0.0001 (***↓)	0.7846±0.0484	0.0060 (**↓)	0.7505±0.0865	0.0029 (**↓)	0.9153±0.0323	0.0080 (**↓)	0.7497±0.0738	0.0037 (**↓)
DSMIL	0.8940±0.0540	0.0018 (**↓)	0.7805±0.0555	<0.0001 (***↓)	0.7961±0.0408	0.0107 (*↓)	0.7420±0.0876	0.0010 (**↓)	0.9143±0.0233	0.0040 (**↓)	0.7470±0.0747	0.0018 (**↓)
TransMIL	0.8926±0.0536	0.0002 (***↓)	0.7738±0.0570	<0.0001 (***↓)	0.7598±0.0713	0.0048 (**↓)	0.7353±0.0863	0.0015 (**↓)	0.9150±0.0230	0.0085 (**↓)	0.7329±0.0807	0.0015 (**↓)
MambaMIL	0.8977±0.0457	0.0094 (**↓)	0.7705±0.0699	<0.0001 (***↓)	0.7527±0.0843	0.0014 (**↓)	0.7395±0.0954	0.0018 (**↓)	0.9160±0.0252	0.0098 (**↓)	0.7303±0.0932	0.0004 (***↓)
MambaMIL+HiLA-MIL (Ours)	0.9240±0.0472	N/A	0.8240±0.0702	N/A	0.8431±0.0612	N/A	0.8238±0.0631	N/A	0.9379±0.0226	N/A	0.8172±0.0595	N/A

Methods	Negative											
	AUC	P-value	Accuracy	P-value	Precision	P-value	Recall	P-value	Specificity	P-value	F1-Score	P-value
ABMIL	0.9619±0.0287	0.1067 (ns↓)	0.9234±0.0447	0.4488 (ns↓)	0.7943±0.1249	0.1450 (ns↓)	0.9429±0.0999	0.3754 (ns↑)	0.9168±0.0527	0.0653 (ns↓)	0.8561±0.0846	0.3907 (ns↓)
CLAM-SB	0.9588±0.0363	0.0129 (*↓)	0.9203±0.0320	0.1398 (ns↓)	0.7804±0.0661	0.1195 (ns↓)	0.9286±0.1010	1.0000 (ns↑)	0.9172±0.0331	0.0681 (ns↓)	0.8454±0.0660	0.2690 (ns↓)
CLAM-MB	0.9656±0.0357	0.1863 (ns↓)	0.9269±0.0492	0.3777 (ns↓)	0.8224±0.1188	0.7315 (ns↓)	0.9054±0.1388	0.2924 (ns↓)	0.9348±0.0470	1.0000 (ns↓)	0.8543±0.0996	0.5241 (ns↓)
DSMIL	0.9572±0.0424	0.0246 (*↓)	0.9302±0.0532	0.4416 (ns↓)	0.8115±0.1254	0.3361 (ns↓)	0.9446±0.0717	0.4596 (ns↑)	0.9257±0.0552	0.1747 (ns↓)	0.8699±0.0933	0.6877 (ns↓)
TransMIL	0.9540±0.0374	0.0064 (**↓)	0.9202±0.0359	0.1484 (ns↓)	0.7757±0.0748	0.0687 (ns↓)	0.9446±0.0717	0.4596 (ns↑)	0.9126±0.0366	0.0206 (*↓)	0.8500±0.0617	0.3064 (ns↓)
MambaMIL	0.9607±0.0397	0.0133 (*↓)	0.9037±0.0656	0.0217 (*↓)	0.7526±0.1410	0.0064 (**↓)	0.9446±0.0983	0.4574 (ns↑)	0.8909±0.0804	0.0021 (**↓)	0.8296±0.1031	0.0822 (ns↓)
MambaMIL+HiLA-MIL (Ours)	0.9794±0.0249	N/A	0.9369±0.0429	N/A	0.8324±0.0952	N/A	0.9286±0.1543	N/A	0.9385±0.0379	N/A	0.8707±0.0983	N/A

Methods	ITC											
	AUC	P-value	Accuracy	P-value	Precision	P-value	Recall	P-value	Specificity	P-value	F1-Score	P-value
ABMIL	0.9199±0.1378	0.1931 (ns↓)	0.9669±0.0269	0.0347 (*↓)	0.8333±0.2222	<0.0001 (***↓)	0.7333±0.2383	<0.0001 (***↓)	0.9857±0.0184	<0.0001 (***↓)	0.7600±0.1936	<0.0001 (***↓)
CLAM-SB	0.9236±0.1146	0.0840 (ns↓)	0.9602±0.0408	<0.0001 (***↓)	0.7833±0.3338	<0.0001 (***↓)	0.6833±0.3282	<0.0001 (***↓)	0.9821±0.0253	0.1270 (ns↓)	0.7100±0.3023	<0.0001 (***↓)
CLAM-MB	0.9207±0.1226	0.2316 (ns↓)	0.9702±0.0242	0.0660 (ns↓)	0.8833±0.1933	<0.0001 (***↓)	0.7333±0.2383	<0.0001 (***↓)	0.9893±0.0173	0.5035 (ns↓)	0.7767±0.1750	<0.0001 (***↓)

DSMIL	0.9190±0.1232	0.1460 (ns↓)	0.9735±0.0206	0.1303 (ns↓)	0.9333±0.1405	0.1303 (ns↓)	0.7333±0.2383	<0.0001 (***↓)	0.9929±0.0151	1.0000 (ns↓)	0.7933±0.1522	<0.0001 (***↓)
TransMIL	0.9293±0.0962	0.0720 (ns↓)	0.9702±0.0329	0.0604 (ns↓)	0.8667±0.3220	0.0604 (ns↓)	0.6833±0.3282	<0.0001 (***↓)	0.9929±0.0151	1.0000 (ns↓)	0.7467±0.3060	<0.0001 (***↓)
MambaMIL	0.9242±0.1081	0.2998 (ns↓)	0.9669±0.0269	0.0329 (*↓)	0.8333±0.2222	<0.0001 (***↓)	0.7333±0.2383	<0.0001 (***↓)	0.9857±0.0184	<0.0001 (***↓)	0.7600±0.1936	<0.0001 (***↓)
MambaMIL+HiLA-MIL (Ours)	0.9405±0.1389	N/A	0.9868±0.0171	N/A	0.9750±0.0791	N/A	0.8500±0.2415	N/A	0.9964±0.0113	N/A	0.8857±0.1575	N/A

Methods	Micro											
	AUC	P-value	Accuracy	P-value	Precision	P-value	Recall	P-value	Specificity	P-value	F1-Score	P-value
ABMIL	0.7883±0.1092	0.0307 (*↓)	0.8138±0.0727	0.0375 (*↓)	0.5158±0.2163	0.1069 (ns↓)	0.5000±0.2131	0.0001 (***↓)	0.8892±0.0473	1.0000 (ns↑)	0.5032±0.2045	0.0061 (**↓)
CLAM-SB	0.8141±0.1148	0.0622 (ns↓)	0.8237±0.0850	0.0889 (ns↓)	0.5676±0.2110	0.2405 (ns↓)	0.5333±0.1950	0.0049 (**↓)	0.8928±0.0740	0.6529 (ns↑)	0.5413±0.1866	0.0306 (*↓)
CLAM-MB	0.8098±0.1154	0.0750 (ns↓)	0.8237±0.0906	0.1217 (ns↓)	0.5843±0.1965	0.2720 (ns↓)	0.5567±0.2183	0.0139 (*↓)	0.8898±0.1080	1.0000 (ns↑)	0.5490±0.1777	0.0559 (ns↓)
DSMIL	0.8020±0.0904	0.0207 (*↓)	0.8270±0.0472	0.1196 (ns↓)	0.6129±0.2035	0.4859 (ns↓)	0.4500±0.1451	0.0001 (***↓)	0.9177±0.0517	0.0359 (*↑)	0.4937±0.1346	0.0095 (**↓)
TransMIL	0.7871±0.0922	0.0026 (**↓)	0.8237±0.0452	0.1182 (ns↓)	0.5448±0.1448	0.3334 (ns↓)	0.5000±0.2131	0.0011 (**↓)	0.9012±0.0489	0.4345 (ns↑)	0.5070±0.1558	0.0163 (*↓)
MambaMIL	0.8083±0.0781	0.0605 (ns↓)	0.8270±0.0522	0.1543 (ns↓)	0.5531±0.1643	0.4883 (ns↓)	0.4667±0.2102	0.0002 (***↓)	0.9137±0.0360	0.1191 (ns↑)	0.4939±0.1814	0.0166 (*↓)
MambaMIL+HiLA-MIL (Ours)	0.8586±0.0744	N/A	0.8539±0.0722	N/A	0.6421±0.1860	N/A	0.7033±0.1651	N/A	0.8887±0.0836	N/A	0.6530±0.1340	N/A

Methods	Macro											
	AUC	P-value	Accuracy	P-value	Precision	P-value	Recall	P-value	Specificity	P-value	F1-Score	P-value
ABMIL	0.8848±0.0538	0.1567 (ns↓)	0.8434±0.0706	0.0984 (ns↓)	0.8709±0.0708	0.0613 (ns↓)	0.8067±0.0966	0.5987 (ns↓)	0.8800±0.0689	0.0344 (*↓)	0.8358±0.0789	0.2108 (ns↓)
CLAM-SB	0.8832±0.0823	0.0591 (ns↓)	0.8302±0.0796	0.0123 (*↓)	0.8592±0.0866	0.0172 (*↓)	0.7933±0.1109	0.2224 (ns↓)	0.8671±0.0892	0.0128 (*↓)	0.8217±0.0868	0.0337 (*↓)
CLAM-MB	0.8859±0.0748	0.1618 (ns↓)	0.8270±0.0720	0.0139 (*↓)	0.8485±0.0829	0.0015 (**↓)	0.8067±0.1350	0.6234 (ns↓)	0.8475±0.0998	0.0001 (***↓)	0.8187±0.0907	0.0517 (ns↓)
DSMIL	0.8979±0.0621	0.0787 (ns↓)	0.8303±0.0488	0.0110 (*↓)	0.8265±0.0560	0.0002 (***↓)	0.8400±0.0717	0.0711 (ns↑)	0.8208±0.0713	<0.0001 (***↓)	0.8312±0.0488	0.0774 (ns↓)
TransMIL	0.9003±0.0453	0.1978 (ns↓)	0.8334±0.0631	0.0348 (*↓)	0.8520±0.0728	0.0077 (**↓)	0.8133±0.1033	1.0000 (ns↓)	0.8533±0.0820	0.0029 (**↓)	0.8280±0.0707	0.1159 (ns↓)
MambaMIL	0.8976±0.0539	0.1411 (ns↓)	0.8435±0.0593	0.1002 (ns↓)	0.8719±0.0818	0.0465 (*↓)	0.8133±0.0878	1.0000 (ns↑)	0.8737±0.0861	0.0247 (*↓)	0.8377±0.0611	0.2306 (ns↓)
MambaMIL+HiLA-MIL (Ours)	0.9176±0.0462	N/A	0.8704±0.0429	N/A	0.9229±0.0513	N/A	0.8133±0.1080	N/A	0.9279±0.0554	N/A	0.8593±0.0539	N/A

*：P<0.05；**：P<0.01；***：P<0.001；ns：no statistical significance.

↑: an increase in metrics of the baseline models compared with the MambaMIL+HiLA-MIL model.

↓: a decrease in metrics of the baseline models compared with the MambaMIL+HiLA-MIL model.

N/A: Not applicable. Statistical comparison was not performed.

A two-sided paired permutation test with 10,000 permutations was applied.

Supplementary Table 3. Evaluation metrics of multi-cancer and individual cancer types on NIMM, DLZX, and YDSY Datasets under ResNet

NIMM

Methods	Multi-cancer											
	AUC	P-value	Accuracy	P-value	Precision	P-value	Recall	P-value	Specificity	P-value	F1-Score	P-value
ABMIL	0.9699±0.0139	0.2777 (ns↓)	0.8604±0.0517	<0.0001 (***↓)	0.5713±0.0430	0.0060 (**↓)	0.5821±0.0566	0.0115 (*↓)	0.9208±0.0381	0.0002 (***↓)	0.5639±0.0472	0.0102 (*↓)
CLAM-SB	0.9668±0.0065	0.2349 (ns↓)	0.8439±0.0294	<0.0001 (***↓)	0.5372±0.0378	0.0044 (**↓)	0.5195±0.0260	0.0006 (***↓)	0.9091±0.0164	<0.0001 (***↓)	0.5153±0.0328	0.0019 (**↓)
CLAM-MB	0.9605±0.0240	0.4784 (ns↓)	0.8155±0.0756	<0.0001 (***↓)	0.4675±0.0311	<0.0001 (***↓)	0.4896±0.0053	0.0001 (***↓)	0.8989±0.0320	<0.0001 (***↓)	0.4605±0.0300	0.0001 (***↓)
DSMIL	0.9724±0.0075	0.6790 (ns↓)	0.8689±0.0413	<0.0001 (***↓)	0.5885±0.0523	0.0819 (ns↓)	0.5889±0.0480	0.5113 (ns↓)	0.9255±0.0322	0.0643 (ns↓)	0.5796±0.0501	0.2114 (ns↓)
TransMIL	0.9725±0.0018	0.2883 (ns↓)	0.8370±0.0241	<0.0001 (***↓)	0.5480±0.0275	0.0013 (**↓)	0.5433±0.0465	0.0147 (*↓)	0.9170±0.0132	0.0004 (***↓)	0.5284±0.0355	0.0059 (**↓)
MambaMIL	0.9681±0.0091	0.0052 (**↓)	0.8623±0.0239	<0.0001 (***↓)	0.5468±0.0627	0.0730 (ns↓)	0.5632±0.0302	0.0164 (*↓)	0.9222±0.0144	0.0017 (**↓)	0.5487±0.0448	0.0347 (*↓)
MambaMIL+HiLA-MIL (Ours)	0.9773±0.0063	N/A	0.9047±0.0200	N/A	0.6508±0.0953	N/A	0.6309±0.0805	N/A	0.9418±0.0285	N/A	0.6315±0.0835	N/A

Methods	Gastric adenocarcinoma											
	AUC	P-value	Accuracy	P-value	Precision	P-value	Recall	P-value	Specificity	P-value	F1-Score	P-value
ABMIL	0.9778±0.0328	0.9667 (ns↓)	0.9199±0.0894	1.0000 (ns↑)	0.6488±0.0893	0.5347 (ns↑)	0.6638±0.0831	0.3802 (ns↑)	0.9727±0.0291	0.4382 (ns↑)	0.6518±0.0905	0.4231 (ns↑)
CLAM-SB	0.9829±0.0238	0.9471 (ns↓)	0.8900±0.0767	<0.0001 (***↓)	0.5197±0.0428	0.2472 (ns↓)	0.5212±0.0469	0.2797 (ns↓)	0.9548±0.0217	0.1505 (ns↓)	0.5175±0.0462	0.2869 (ns↓)
CLAM-MB	0.9785±0.0326	0.8847 (ns↓)	0.8785±0.1195	0.0024 (**↓)	0.5310±0.0382	0.2608 (ns↓)	0.5707±0.0690	0.9409 (ns↓)	0.9595±0.0280	0.5436 (ns↓)	0.5231±0.0407	0.5206 (ns↓)
DSMIL	0.9841±0.0210	0.9034 (ns↑)	0.8971±0.0810	0.0401 (*↓)	0.5454±0.0707	0.7184 (ns↓)	0.5737±0.1370	0.9304 (ns↓)	0.9642±0.0293	0.8258 (ns↓)	0.5505±0.0928	0.9811 (ns↓)
TransMIL	0.9745±0.0217	0.3195 (ns↓)	0.8469±0.0948	<0.0001 (***↓)	0.5230±0.0378	0.1536 (ns↓)	0.5519±0.0926	1.0000 (ns↓)	0.9479±0.0197	0.0806 (ns↓)	0.5117±0.0262	0.3082 (ns↓)
MambaMIL	0.9869±0.0153	0.4192 (ns↑)	0.9041±0.0669	0.0692 (ns↓)	0.5332±0.0293	0.5125 (ns↓)	0.5540±0.0495	0.8583 (ns↓)	0.9640±0.0274	0.6354 (ns↓)	0.5381±0.0333	0.6708 (ns↓)
MambaMIL+HiLA-MIL (Ours)	0.9833±0.0227	N/A	0.9193±0.0785	N/A	0.6024±0.1420	N/A	0.6153±0.1537	N/A	0.9684±0.0327	N/A	0.6058±0.1468	N/A

Methods	Colorectal adenocarcinoma											
	AUC	P-value	Accuracy	P-value	Precision	P-value	Recall	P-value	Specificity	P-value	F1-Score	P-value
ABMIL	0.9888±0.0152	0.7729 (ns↓)	0.9030±0.0672	<0.0001 (***↓)	0.5671±0.0696	0.0404 (*↓)	0.6560±0.1434	0.0619 (ns↓)	0.9720±0.0229	0.0008 (***↓)	0.5770±0.0744	0.0551 (ns↓)
CLAM-SB	0.9901±0.0128	0.8251 (ns↓)	0.9018±0.0532	<0.0001 (***↓)	0.5118±0.0365	0.0020 (**↓)	0.5368±0.0810	<0.0001 (***↓)	0.9702±0.0133	<0.0001 (***↓)	0.5157±0.0444	0.0020 (**↓)
CLAM-MB	0.9927±0.0098	0.9979 (ns↓)	0.9200±0.0553	<0.0001 (***↓)	0.5161±0.0510	0.1060 (ns↓)	0.5512±0.0887	0.1274 (ns↓)	0.9728±0.0125	0.0338 (*↓)	0.5247±0.0547	0.0592 (ns↓)
DSMIL	0.9923±0.0118	0.9929 (ns↓)	0.9332±0.0719	<0.0001 (***↓)	0.6846±0.2744	0.0305 (*↓)	0.7098±0.2621	0.0305 (*↓)	0.9816±0.0207	0.1241 (ns↓)	0.6890±0.2721	0.0305 (*↓)
TransMIL	0.9926±0.0072	0.6900 (ns↓)	0.9049±0.0315	<0.0001 (***↓)	0.5597±0.1110	0.0014 (**↓)	0.5532±0.1270	0.0014 (**↓)	0.9727±0.0059	0.0014 (**↓)	0.5441±0.0985	0.0014 (**↓)
MambaMIL	0.9933±0.0069	0.4783 (ns↓)	0.9478±0.0303	0.2479 (ns↓)	0.6485±0.1403	0.2479 (ns↓)	0.6795±0.1655	0.2479 (ns↓)	0.9855±0.0094	1.0000 (ns↓)	0.6576±0.1460	0.2479 (ns↓)

MambaMIL+HiLA-MIL (Ours)	0.9943±0.0080	N/A	0.9543±0.0410	N/A	0.7596±0.2506	N/A	0.7628±0.2566	N/A	0.9872±0.0121	N/A	0.7576±0.2530	N/A

Methods	Lung adenocarcinoma											
	AUC	P-value	Accuracy	P-value	Precision	P-value	Recall	P-value	Specificity	P-value	F1-Score	P-value
ABMIL	0.9847±0.0153	0.9406 (ns↓)	0.8879±0.0496	<0.0001 (***↓)	0.6509±0.1400	0.9674 (ns↓)	0.6261±0.0458	<0.0001 (***↓)	0.9578±0.0201	<0.0001 (***↓)	0.6268±0.0802	0.7247 (ns↓)
CLAM-SB	0.9835±0.0166	1.0000 (ns↓)	0.8924±0.0531	<0.0001 (***↓)	0.6329±0.1986	0.1305 (ns↓)	0.5923±0.0971	0.0333 (*↓)	0.9614±0.0168	0.0333 (*↓)	0.5960±0.1283	0.0333 (*↓)
CLAM-MB	0.9575±0.0464	0.9870 (ns↓)	0.8289±0.1268	<0.0001 (***↓)	0.4817±0.0768	0.1863 (ns↓)	0.4815±0.0713	0.0011 (**↓)	0.9358±0.0391	0.0014 (**↓)	0.4654±0.0744	0.0202 (*↓)
DSMIL	0.9807±0.0215	0.9361 (ns↓)	0.9035±0.0483	<0.0001 (***↓)	0.6399±0.1857	<0.0001 (***↓)	0.6059±0.1106	<0.0001 (***↓)	0.9674±0.0189	0.2523 (ns↓)	0.6137±0.1357	<0.0001 (***↓)
TransMIL	0.9858±0.0134	0.9998 (ns↓)	0.8956±0.0602	<0.0001 (***↓)	0.5878±0.1109	<0.0001 (***↓)	0.6010±0.1030	<0.0001 (***↓)	0.9653±0.0168	0.1255 (ns↓)	0.5923±0.1069	<0.0001 (***↓)
MambaMIL	0.9863±0.0159	0.5246 (ns↓)	0.9147±0.0508	<0.0001 (***↓)	0.6505±0.1854	0.5039 (ns↓)	0.6106±0.1103	<0.0001 (***↓)	0.9683±0.0195	<0.0001 (***↓)	0.6228±0.1362	<0.0001 (***↓)
MambaMIL+HiLA-MIL (Ours)	0.9871±0.0158	N/A	0.9303±0.0480	N/A	0.7464±0.1252	N/A	0.6661±0.0835	N/A	0.9756±0.0171	N/A	0.6922±0.0938	N/A

Methods	Breast invasive ductal carcinoma											
	AUC	P-value	Accuracy	P-value	Precision	P-value	Recall	P-value	Specificity	P-value	F1-Score	P-value
ABMIL	0.9374±0.0273	0.2734 (ns↓)	0.7130±0.1099	0.0003 (***↓)	0.6810±0.0743	0.0748 (ns↓)	0.6602±0.0792	0.0372 (*↓)	0.8973±0.0331	0.0315 (*↓)	0.6602±0.0759	0.0713 (ns↓)
CLAM-SB	0.9346±0.0254	0.1207 (ns↓)	0.7459±0.0587	0.0338 (*↓)	0.7259±0.0530	0.3934 (ns↓)	0.6932±0.0300	0.0815 (ns↓)	0.9051±0.0228	0.0792 (ns↓)	0.6946±0.0406	0.2008 (ns↓)
CLAM-MB	0.9105±0.0350	0.0245 (*↓)	0.6608±0.1019	<0.0001 (***↓)	0.5133±0.0969	0.0002 (***↓)	0.5906±0.0667	0.0010 (**↓)	0.8722±0.0279	0.0007 (***↓)	0.5367±0.0936	0.0005 (***↓)
DSMIL	0.9466±0.0173	0.4854 (ns↓)	0.7656±0.0591	0.2654 (ns↓)	0.7636±0.0281	0.8812 (ns↑)	0.7594±0.0074	0.9821 (ns↑)	0.9187±0.0173	0.5937 (ns↓)	0.7457±0.0338	0.9518 (ns↑)
TransMIL	0.9426±0.0329	0.5588 (ns↓)	0.7484±0.0567	0.0611 (ns↓)	0.7263±0.0305	0.3810 (ns↓)	0.7239±0.0487	0.3272 (ns↓)	0.9123±0.0273	0.2708 (ns↓)	0.7007±0.0490	0.4078 (ns↓)
MambaMIL	0.9375±0.0315	0.0433 (*↓)	0.7502±0.1317	0.0140 (*↓)	0.6761±0.1623	0.5008 (ns↓)	0.7303±0.0768	0.2090 (ns↓)	0.9100±0.0498	0.0992 (ns↓)	0.6924±0.1229	0.2192 (ns↓)
MambaMIL+HiLA-MIL (Ours)	0.9496±0.0167	N/A	0.7777±0.0433	N/A	0.7465±0.0667	N/A	0.7559±0.0529	N/A	0.9236±0.0143	N/A	0.7343±0.0514	N/A

Methods	Breast invasive lobular Carcinoma											
	AUC	P-value	Accuracy	P-value	Precision	P-value	Recall	P-value	Specificity	P-value	F1-Score	P-value
ABMIL	0.9609±0.0461	0.5450 (ns↓)	0.8783±0.0527	<0.0001 (***↓)	0.3089±0.1432	<0.0001 (***↓)	0.3042±0.1161	<0.0001 (***↓)	0.8042±0.1161	<0.0001 (***↓)	0.3038±0.1264	<0.0001 (***↓)
CLAM-SB	0.9428±0.0366	0.4591 (ns↓)	0.7894±0.0353	<0.0001 (***↓)	0.2955±0.1203	<0.0001 (***↓)	0.2542±0.0564	<0.0001 (***↓)	0.7542±0.0564	<0.0001 (***↓)	0.2526±0.0502	<0.0001 (***↓)
CLAM-MB	0.9632±0.0045	0.9240 (ns↓)	0.7894±0.0353	<0.0001 (***↓)	0.2955±0.1203	<0.0001 (***↓)	0.2542±0.0564	<0.0001 (***↓)	0.7542±0.0564	<0.0001 (***↓)	0.2526±0.0502	<0.0001 (***↓)
DSMIL	0.9586±0.0213	0.8148 (ns↓)	0.8449±0.0628	<0.0001 (***↓)	0.3089±0.1432	<0.0001 (***↓)	0.2958±0.1252	<0.0001 (***↓)	0.7958±0.1252	<0.0001 (***↓)	0.2989±0.1305	<0.0001 (***↓)
TransMIL	0.9669±0.0134	0.7419 (ns↓)	0.7894±0.0353	<0.0001 (***↓)	0.3430±0.0910	<0.0001 (***↓)	0.2866±0.0770	<0.0001 (***↓)	0.7866±0.0770	<0.0001 (***↓)	0.2933±0.0643	<0.0001 (***↓)
MambaMIL	0.9368±0.0440	0.2257 (ns↓)	0.7949±0.1184	<0.0001 (***↓)	0.2256±0.0229	<0.0001 (***↓)	0.2417±0.0144	<0.0001 (***↓)	0.7833±0.0804	<0.0001 (***↓)	0.2324±0.0064	<0.0001 (***↓)

MambaMIL+HiLA-MIL (Ours)	0.9724±0.0364	N/A	0.9419±0.0504	N/A	0.3991±0.1291	N/A	0.3542±0.0955	N/A	0.8542±0.0955	N/A	0.3677±0.1045	N/A

Supplementary Table 3. Continued

DLZX

Methods	Multi-cancer											
	AUC	P-value	Accuracy	P-value	Precision	P-value	Recall	P-value	Specificity	P-value	F1-Score	P-value
ABMIL	0.9299±0.0091	0.6919 (ns↑)	0.7906±0.0420	0.2113 (ns↑)	0.4144±0.0619	0.0738 (ns↓)	0.3776±0.0303	0.0302 (*↓)	0.8911±0.0469	0.1305 (ns↑)	0.3820±0.0404	0.0402 (*↓)
CLAM-SB	0.9238±0.0196	0.8287 (ns↓)	0.7663±0.0613	0.2626 (ns↓)	0.3958±0.0277	0.1031 (ns↓)	0.3755±0.0389	0.0973 (ns↓)	0.8934±0.0548	0.8534 (ns↑)	0.3734±0.0357	0.0816 (ns↓)
CLAM-MB	0.9338±0.0227	0.8445 (ns↑)	0.7730±0.0612	0.0198 (*↑)	0.4421±0.0928	0.1452 (ns↓)	0.3958±0.0695	0.1127 (ns↓)	0.8942±0.0528	0.4680 (ns↑)	0.4039±0.0761	0.1178 (ns↓)
DSMIL	0.9278±0.0226	0.8405 (ns↓)	0.7725±0.0404	0.0158 (*↑)	0.4526±0.0836	0.2087 (ns↓)	0.3933±0.0499	0.1516 (ns↓)	0.8843±0.0469	0.2528 (ns↑)	0.4015±0.0600	0.1645 (ns↓)
TransMIL	0.9283±0.0190	0.9764 (ns↓)	0.7693±0.0534	0.5757 (ns↓)	0.4317±0.0764	0.8510 (ns↓)	0.4059±0.0285	0.5763 (ns↓)	0.8762±0.0530	0.2198 (ns↓)	0.3985±0.0502	0.7845 (ns↓)
MambaMIL	0.9042±0.0546	0.6646 (ns↓)	0.7540±0.0955	0.0439 (*↓)	0.4487±0.1342	0.0714 (ns↓)	0.3851±0.0778	0.1954 (ns↓)	0.8978±0.0539	0.8299 (ns↑)	0.4030±0.0958	0.1034 (ns↓)
MambaMIL+HiLA-MIL (Ours)	0.9299±0.0206	N/A	0.7694±0.0296	N/A	0.4617±0.0684	N/A	0.4271±0.0435	N/A	0.8840±0.0348	N/A	0.4295±0.0582	N/A

Methods	Gastric adenocarcinoma											
	AUC	P-value	Accuracy	P-value	Precision	P-value	Recall	P-value	Specificity	P-value	F1-Score	P-value
ABMIL	0.9209±0.0264	0.7258 (ns↑)	0.7563±0.0268	0.3660 (ns↑)	0.5262±0.1477	0.5396 (ns↓)	0.5027±0.0352	0.7491 (ns↓)	0.9090±0.0147	0.9673 (ns↓)	0.4960±0.0789	0.9673 (ns↓)
CLAM-SB	0.9114±0.0287	0.8999 (ns↓)	0.7103±0.0418	0.4531 (ns↓)	0.3912±0.0053	0.1723 (ns↓)	0.4352±0.0128	0.2191 (ns↓)	0.8912±0.0101	0.3737 (ns↓)	0.4041±0.0150	0.1723 (ns↓)
CLAM-MB	0.9209±0.0141	0.9999 (ns↑)	0.7341±0.0611	1.0000 (ns↓)	0.5295±0.1301	0.9513 (ns↓)	0.4971±0.0771	0.8453 (ns↓)	0.9066±0.0186	0.8120 (ns↓)	0.5068±0.1013	0.9891 (ns↓)
DSMIL	0.9109±0.0177	0.7469 (ns↓)	0.7325±0.0179	1.0000 (ns↓)	0.5223±0.1273	1.0000 (ns↓)	0.4832±0.0561	0.5356 (ns↓)	0.9040±0.0027	0.6944 (ns↓)	0.4853±0.0643	0.6273 (ns↓)
TransMIL	0.9036±0.0587	0.9150 (ns↓)	0.6954±0.1494	0.0716 (ns↓)	0.5184±0.1774	0.5511 (ns↓)	0.4957±0.0847	0.5971 (ns↓)	0.8879±0.0609	0.3169 (ns↓)	0.4632±0.1211	0.5602 (ns↓)
MambaMIL	0.8832±0.0553	0.5702 (ns↓)	0.6925±0.0834	0.0329 (*↓)	0.4937±0.1793	0.5039 (ns↓)	0.4352±0.0950	0.1565 (ns↓)	0.8961±0.0350	0.3096 (ns↓)	0.4396±0.1173	0.2919 (ns↓)
MambaMIL+HiLA-MIL (Ours)	0.9148±0.0275	N/A	0.7355±0.0491	N/A	0.5667±0.1196	N/A	0.5186±0.0443	N/A	0.9108±0.0236	N/A	0.5176±0.0619	N/A

Methods	Colorectal adenocarcinoma											
	AUC	P-value	Accuracy	P-value	Precision	P-value	Recall	P-value	Specificity	P-value	F1-Score	P-value
ABMIL	0.9385±0.0429	1.0000 (ns↓)	0.8223±0.0749	0.0000 (***↓)	0.4481±0.0228	0.0133 (*↓)	0.4439±0.0410	0.0204 (*↓)	0.9309±0.0285	0.0385 (*↓)	0.4424±0.0342	0.0171 (*↓)
CLAM-SB	0.9531±0.0392	0.9984 (ns↑)	0.8561±0.0705	0.5850 (ns↓)	0.5001±0.0578	0.1283 (ns↓)	0.5250±0.0692	0.2056 (ns↓)	0.9458±0.0257	0.8853 (ns↓)	0.5089±0.0620	0.1448 (ns↓)
CLAM-MB	0.9425±0.0482	0.9270 (ns↑)	0.8231±0.0551	0.0001 (***↓)	0.4807±0.0401	0.0138 (*↓)	0.5072±0.0580	0.0661 (ns↓)	0.9359±0.0209	0.1378 (ns↓)	0.4825±0.0380	0.0227 (*↓)
DSMIL	0.9407±0.0477	0.9914 (ns↑)	0.8217±0.0907	<0.0001 (***↓)	0.5483±0.1341	0.0379 (*↓)	0.5001±0.1008	0.0644 (ns↓)	0.9341±0.0304	0.1349 (ns↓)	0.4979±0.0855	0.0429 (*↓)
TransMIL	0.9411±0.0328	0.9887 (ns↑)	0.8429±0.0867	0.0652 (ns↓)	0.5616±0.0383	0.2354 (ns↓)	0.5746±0.0361	0.3369 (ns↓)	0.9417±0.0313	0.4255 (ns↓)	0.5589±0.0441	0.2625 (ns↓)

MambaMIL	0.9443±0.0375	0.9275 (ns↑)	0.8509±0.0819	0.2528 (ns↓)	0.5571±0.1471	0.5124 (ns↓)	0.5208±0.0906	0.1813 (ns↓)	0.9432±0.0266	0.6324 (ns↓)	0.5309±0.1074	0.2238 (ns↓)
MambaMIL+HiLA-MIL (Ours)	0.9407±0.0412	N/A	0.8644±0.0332	N/A	0.5838±0.0143	N/A	0.5868±0.0038	N/A	0.9475±0.0133	N/A	0.5821±0.0094	N/A

Methods	Breast invasive ductal carcinoma											
	AUC	P-value	Accuracy	P-value	Precision	P-value	Recall	P-value	Specificity	P-value	F1-Score	P-value
ABMIL	0.9455±0.0161	0.9945 (ns↓)	0.8126±0.0854	1.0000 (ns↑)	0.3501±0.1112	0.8450 (ns↓)	0.2812±0.0276	0.5054 (ns↓)	0.8650±0.0460	0.1763 (ns↓)	0.2890±0.0381	0.5948 (ns↓)
CLAM-SB	0.9322±0.0241	0.8108 (ns↓)	0.8015±0.0835	0.4492 (ns↓)	0.3585±0.1244	0.7473 (ns↓)	0.2778±0.0262	0.4492 (ns↓)	0.8955±0.0353	0.9951 (ns↑)	0.2919±0.0399	0.6707 (ns↓)
CLAM-MB	0.9482±0.0223	0.9474 (ns↑)	0.8006±0.1134	0.3682 (ns↓)	0.4248±0.1064	0.7160 (ns↓)	0.3056±0.0610	0.3682 (ns↓)	0.8842±0.0382	0.6828 (ns↓)	0.3310±0.0725	0.5845 (ns↓)
DSMIL	0.9412±0.0208	0.7094 (ns↓)	0.8015±0.0835	0.4527 (ns↓)	0.4302±0.0967	0.8604 (ns↑)	0.3168±0.0447	0.5302 (ns↓)	0.8836±0.0314	0.6036 (ns↓)	0.3415±0.0582	0.8110 (ns↓)
TransMIL	0.9558±0.0124	0.8205 (ns↑)	0.8341±0.0735	0.0734 (ns↑)	0.3448±0.1227	1.0000 (ns↓)	0.2986±0.0434	0.9214 (ns↓)	0.8843±0.0410	0.5160 (ns↓)	0.3073±0.0596	0.9579 (ns↓)
MambaMIL	0.9288±0.0653	0.8727 (ns↓)	0.8124±0.1373	1.0000 (ns↑)	0.4106±0.0910	0.7198 (ns↓)	0.3299±0.0694	1.0000 (ns↓)	0.9204±0.0123	0.1997 (ns↑)	0.3599±0.0724	0.8124 (ns↓)
MambaMIL+HiLA-MIL (Ours)	0.9473±0.0181	N/A	0.8101±0.1280	N/A	0.4283±0.0931	N/A	0.3576±0.1234	N/A	0.8898±0.0402	N/A	0.3677±0.1112	N/A

Methods	Breast invasive lobular Carcinoma											
	AUC	P-value	Accuracy	P-value	Precision	P-value	Recall	P-value	Specificity	P-value	F1-Score	P-value
ABMIL	0.9146±0.0347	0.8488 (ns↓)	0.7713±0.1343	<0.0001 (***↑)	0.3333±0.1443	0.8708 (ns↑)	0.2824±0.0814	<0.0001 (***↑)	0.8595±0.1209	<0.0001 (***↑)	0.3007±0.1007	0.8708 (ns↑)
CLAM-SB	0.8985±0.0072	0.9074 (ns↓)	0.6972±0.0914	1.0000 (ns↑)	0.3333±0.1443	1.0000 (ns↑)	0.2639±0.1049	1.0000 (ns↑)	0.8410±0.1525	0.1866 (ns↑)	0.2889±0.1134	0.8703 (ns↑)
CLAM-MB	0.9235±0.0208	0.4837 (ns↑)	0.7343±0.0953	<0.0001 (***↑)	0.3333±0.1443	1.0000 (ns↑)	0.2731±0.0925	<0.0001 (***↑)	0.8502±0.1367	0.1295 (ns↑)	0.2951±0.1064	1.0000 (ns↑)
DSMIL	0.9183±0.0381	0.6453 (ns↑)	0.7343±0.0953	<0.0001 (***↑)	0.3095±0.1688	<0.0001 (***↑)	0.2731±0.0925	<0.0001 (***↑)	0.8155±0.1283	0.4963 (ns↑)	0.2812±0.1174	<0.0001 (***↑)
TransMIL	0.9129±0.0440	0.6940 (ns↓)	0.7046±0.1601	<0.0001 (***↑)	0.3021±0.1777	<0.0001 (***↑)	0.2546±0.0350	<0.0001 (***↑)	0.7907±0.0956	0.7524 (ns↑)	0.2648±0.0908	<0.0001 (***↑)
MambaMIL	0.8603±0.0682	0.0738 (ns↓)	0.6602±0.1260	0.2513 (ns↓)	0.3333±0.1443	1.0000 (ns↑)	0.2546±0.1181	0.2513 (ns↑)	0.8317±0.1684	0.3121 (ns↑)	0.2817±0.1222	0.6805 (ns↑)
MambaMIL+HiLA-MIL (Ours)	0.9166±0.0228	N/A	0.6676±0.0962	N/A	0.2679±0.0994	N/A	0.2454±0.0488	N/A	0.7877±0.0870	N/A	0.2507±0.0646	N/A

Supplementary Table 3. Continued

YDSY

Methods	Multi-cancer											
	AUC	P-value	Accuracy	P-value	Precision	P-value	Recall	P-value	Specificity	P-value	F1-Score	P-value
ABMIL	0.8883±0.0269	0.8178 (ns↓)	0.6922±0.0190	0.1478 (ns↑)	0.4587±0.0423	0.0195 (*↓)	0.4482±0.0352	0.0973 (ns↓)	0.8960±0.0191	0.6385 (ns↑)	0.4370±0.0257	0.0322 (*↓)
CLAM-SB	0.8846±0.0294	0.7504 (ns↓)	0.6680±0.0342	0.6673 (ns↓)	0.4234±0.0512	0.0915 (ns↓)	0.4285±0.0870	0.0297 (*↓)	0.8647±0.0291	0.7424 (ns↓)	0.4101±0.0624	0.0431 (*↓)
CLAM-MB	0.8860±0.0308	0.5270 (ns↓)	0.6895±0.0310	0.5309 (ns↑)	0.4601±0.0566	0.1283 (ns↓)	0.4596±0.0487	0.4970 (ns↓)	0.8945±0.0095	0.4067 (ns↑)	0.4360±0.0341	0.2128 (ns↓)
DSMIL	0.8894±0.0302	0.8400 (ns↓)	0.6937±0.0435	0.5066 (ns↑)	0.4755±0.0541	0.1485 (ns↓)	0.4586±0.0597	0.2897 (ns↓)	0.8876±0.0234	0.8597 (ns↑)	0.4520±0.0552	0.2053 (ns↓)

TransMIL	0.8634±0.0264	0.2249 (ns↓)	0.6413±0.0358	0.0084 (**↓)	0.4058±0.0176	0.0068 (**↓)	0.4201±0.0259	0.1295 (ns↓)	0.8657±0.0054	0.5594 (ns↓)	0.3935±0.0091	0.0158 (*↓)
MambaMIL	0.8820±0.0066	0.4158 (ns↓)	0.6662±0.0201	0.1060 (ns↓)	0.4348±0.0528	0.0752 (ns↓)	0.4298±0.0558	0.1491 (ns↓)	0.8735±0.0086	0.3792 (ns↓)	0.4116±0.0379	0.0548 (ns↓)
MambaMIL+HiLA-MIL (Ours)	0.8949±0.0187	N/A	0.6828±0.0559	N/A	0.4898±0.0696	N/A	0.4708±0.0599	N/A	0.8835±0.0312	N/A	0.4555±0.0499	N/A

Methods	Gastric adenocarcinoma											
	AUC	P-value	Accuracy	P-value	Precision	P-value	Recall	P-value	Specificity	P-value	F1-Score	P-value
ABMIL	0.7947±0.1455	0.1870 (ns↓)	0.5167±0.2021	1.0000 (ns↓)	0.2917±0.1909	0.4961 (ns↓)	0.2361±0.1203	0.4961 (ns↓)	0.8292±0.1201	0.4961 (ns↑)	0.2532±0.1444	0.4961 (ns↓)
CLAM-SB	0.7589±0.1467	0.2747 (ns↓)	0.5000±0.1000	1.0000 (ns↓)	0.1889±0.0536	0.3706 (ns↓)	0.2222±0.0481	0.6223 (ns↓)	0.8042±0.0505	0.7460 (ns↓)	0.1958±0.0072	0.3706 (ns↓)
CLAM-MB	0.7956±0.1943	0.2158 (ns↓)	0.5333±0.4163	1.0000 (ns↑)	0.2917±0.1909	0.5010 (ns↓)	0.2500±0.2205	1.0000 (ns↓)	0.8333±0.1528	0.6261 (ns↑)	0.2560±0.2115	1.0000 (ns↓)
DSMIL	0.7925±0.1263	0.3390 (ns↓)	0.5833±0.1756	<0.0001 (***↑)	0.3750±0.2165	<0.0001 (***↑)	0.2778±0.1049	<0.0001 (***↑)	0.8458±0.1277	<0.0001 (***↑)	0.3087±0.1458	1.0000 (ns↑)
TransMIL	0.7411±0.1544	<0.0001 (***↓)	0.4333±0.0577	0.2482 (ns↓)	0.1944±0.0481	1.0000 (ns↓)	0.2083±0.0417	0.2482 (ns↓)	0.7764±0.0354	0.2482 (ns↓)	0.1972±0.0293	0.2482 (ns↓)
MambaMIL	0.7906±0.1459	0.0536 (ns↓)	0.5167±0.2021	1.0000 (ns↓)	0.2639±0.2055	0.4961 (ns↓)	0.2361±0.1203	0.4961 (ns↓)	0.8042±0.1214	0.4961 (ns↓)	0.2421±0.1517	0.4961 (ns↓)
MambaMIL+HiLA-MIL (Ours)	0.8281±0.0948	N/A	0.5167±0.2843	N/A	0.3611±0.2406	N/A	0.2500±0.1502	N/A	0.8181±0.1754	N/A	0.2889±0.1798	N/A

Methods	Colorectal adenocarcinoma											
	AUC	P-value	Accuracy	P-value	Precision	P-value	Recall	P-value	Specificity	P-value	F1-Score	P-value
ABMIL	0.9323±0.0341	0.9671 (ns↓)	0.7294±0.0889	<0.0001 (***↓)	0.4620±0.0585	0.1499 (ns↓)	0.4367±0.0434	<0.0001 (***↓)	0.9035±0.0364	0.4914 (ns↓)	0.4416±0.0531	0.0691 (ns↓)
CLAM-SB	0.9319±0.0563	0.7714 (ns↓)	0.7799±0.1073	0.2435 (ns↓)	0.4869±0.1227	0.3057 (ns↓)	0.4673±0.0704	0.2435 (ns↓)	0.9151±0.0447	0.6842 (ns↑)	0.4707±0.0955	0.3366 (ns↓)
CLAM-MB	0.9165±0.0538	0.2190 (ns↓)	0.7419±0.1314	0.0055 (**↓)	0.4410±0.0378	0.3210 (ns↓)	0.4309±0.0423	0.1483 (ns↓)	0.9093±0.0450	0.9880 (ns↓)	0.4270±0.0468	0.2356 (ns↓)
DSMIL	0.9339±0.0384	0.8834 (ns↓)	0.7684±0.1070	<0.0001 (***↓)	0.4931±0.1172	0.2213 (ns↓)	0.4793±0.0657	0.1272 (ns↓)	0.9131±0.0411	0.8137 (ns↑)	0.4780±0.0912	0.2220 (ns↓)
TransMIL	0.9272±0.0564	0.7847 (ns↓)	0.7649±0.1496	0.0679 (ns↓)	0.4847±0.1236	0.5250 (ns↓)	0.4715±0.1050	0.3607 (ns↓)	0.9128±0.0559	0.7905 (ns↑)	0.4737±0.1182	0.4402 (ns↓)
MambaMIL	0.9222±0.0598	0.7711 (ns↓)	0.7419±0.1314	<0.0001 (***↓)	0.4516±0.0824	0.3261 (ns↓)	0.4374±0.0670	0.0312 (*↓)	0.8966±0.0544	0.1730 (ns↓)	0.4337±0.0848	0.0953 (ns↓)
MambaMIL+HiLA-MIL (Ours)	0.9361±0.0493	N/A	0.7914±0.1112	N/A	0.5078±0.1249	N/A	0.5103±0.0992	N/A	0.9112±0.0490	N/A	0.5022±0.1093	N/A

Methods	Lung adenocarcinoma											
	AUC	P-value	Accuracy	P-value	Precision	P-value	Recall	P-value	Specificity	P-value	F1-Score	P-value
ABMIL	0.8323±0.1111	0.6850 (ns↓)	0.6215±0.1748	0.2488 (ns↑)	0.5458±0.2470	0.3777 (ns↑)	0.5278±0.2208	0.2488 (ns↑)	0.8738±0.0751	0.2488 (ns↑)	0.4936±0.1868	0.7454 (ns↑)
CLAM-SB	0.8260±0.1204	0.9167 (ns↓)	0.5606±0.2061	0.2482 (ns↓)	0.5014±0.2479	0.5562 (ns↑)	0.4792±0.2612	0.2482 (ns↓)	0.8497±0.0779	0.4325 (ns↓)	0.4633±0.2376	0.6790 (ns↓)
CLAM-MB	0.8317±0.0647	0.8373 (ns↓)	0.5940±0.0854	1.0000 (ns↓)	0.5625±0.2052	0.2817 (ns↑)	0.5278±0.1375	0.5031 (ns↑)	0.8703±0.0416	0.5968 (ns↑)	0.4882±0.1163	0.7550 (ns↑)
DSMIL	0.8235±0.1125	0.8640 (ns↓)	0.5606±0.2061	0.4536 (ns↓)	0.4770±0.1759	0.2658 (ns↓)	0.4347±0.1520	0.3604 (ns↓)	0.8487±0.0716	0.7637 (ns↓)	0.4282±0.1557	0.2860 (ns↓)

TransMIL	0.8233±0.1078	0.6393 (ns↓)	0.6329±0.1820	0.3766 (ns↑)	0.4518±0.1091	0.2039 (ns↓)	0.4653±0.1457	0.7517 (ns↓)	0.8690±0.0696	0.7198 (ns↑)	0.4480±0.1331	0.4017 (ns↓)
MambaMIL	0.8359±0.0698	0.6930 (ns↓)	0.6567±0.1423	0.0316 (*↑)	0.4667±0.0846	0.5250 (ns↓)	0.4847±0.1128	1.0000 (ns↓)	0.8773±0.0562	0.5635 (ns↑)	0.4683±0.0986	0.9861 (ns↑)
MambaMIL+HiLA-MIL (Ours)	0.8392±0.1199	N/A	0.5958±0.1454	N/A	0.4972±0.1946	N/A	0.4889±0.2028	N/A	0.8596±0.0594	N/A	0.4667±0.1673	N/A

Methods	Breast invasive ductal carcinoma											
	AUC	P-value	Accuracy	P-value	Precision	P-value	Recall	P-value	Specificity	P-value	F1-Score	P-value
ABMIL	0.9443±0.0167	0.3384 (ns↑)	0.7659±0.0807	1.0000 (ns↓)	0.6189±0.0554	0.4578 (ns↓)	0.6239±0.0242	0.4916 (ns↓)	0.9234±0.0352	0.7162 (ns↑)	0.6079±0.0374	0.7181 (ns↓)
CLAM-SB	0.9483±0.0388	0.3825 (ns↑)	0.8030±0.1093	0.0333 (*↑)	0.7244±0.0982	0.4845 (ns↑)	0.7237±0.2003	0.7745 (ns↑)	0.9313±0.0501	0.5624 (ns↑)	0.6903±0.1399	0.3855 (ns↑)
CLAM-MB	0.9489±0.0117	0.6050 (ns↑)	0.7926±0.0420	0.1109 (ns↑)	0.6470±0.0807	0.7646 (ns↓)	0.6895±0.1220	0.8610 (ns↓)	0.9370±0.0165	0.2764 (ns↑)	0.6367±0.0549	0.9961 (ns↑)
DSMIL	0.9447±0.0368	0.4684 (ns↑)	0.7763±0.1261	1.0000 (ns↑)	0.6851±0.1243	0.8776 (ns↑)	0.7426±0.1877	0.5831 (ns↑)	0.9259±0.0490	0.8313 (ns↑)	0.6950±0.1465	0.5009 (ns↑)
TransMIL	0.9346±0.0252	0.7304 (ns↓)	0.7207±0.1200	0.0371 (*↓)	0.5925±0.1543	0.2127 (ns↓)	0.6163±0.1598	0.2211 (ns↓)	0.9092±0.0475	0.3950 (ns↓)	0.5613±0.1160	0.3745 (ns↓)
MambaMIL	0.9393±0.0178	0.9446 (ns↑)	0.7607±0.0389	1.0000 (ns↓)	0.7282±0.0968	0.6869 (ns↑)	0.7072±0.0952	0.6961 (ns↑)	0.9216±0.0174	0.9960 (ns↓)	0.6654±0.0833	0.5286 (ns↑)
MambaMIL+HiLA-MIL (Ours)	0.9369±0.0367	N/A	0.7659±0.0807	N/A	0.6830±0.0199	N/A	0.6911±0.1204	N/A	0.9219±0.0401	N/A	0.6366±0.0645	N/A

Methods	Breast invasive lobular Carcinoma											
	AUC	P-value	Accuracy	P-value	Precision	P-value	Recall	P-value	Specificity	P-value	F1-Score	P-value
ABMIL	0.9379±0.0684	0.9766 (ns↑)	0.8274±0.1893	0.2473 (ns↑)	0.3750±0.1250	1.0000 (ns↓)	0.4167±0.1443	0.7485 (ns↑)	0.9499±0.0548	0.1214 (ns↑)	0.3889±0.1273	0.7485 (ns↑)
CLAM-SB	0.9579±0.0314	0.8987 (ns↑)	0.6964±0.0644	0.3813 (ns↓)	0.2153±0.0318	0.1891 (ns↓)	0.2500±0.0000	0.1891 (ns↓)	0.8234±0.0690	0.4388 (ns↓)	0.2305±0.0181	0.1891 (ns↓)
CLAM-MB	0.9374±0.0601	0.9178 (ns↑)	0.7857±0.2575	1.0000 (ns↑)	0.3583±0.1507	1.0000 (ns↓)	0.4000±0.1732	0.4943 (ns↓)	0.9226±0.1001	1.0000 (ns↑)	0.3722±0.1549	0.4943 (ns↓)
DSMIL	0.9523±0.0447	0.8897 (ns↑)	0.7798±0.1959	1.0000 (ns↑)	0.3472±0.1463	0.6289 (ns↓)	0.3583±0.1283	0.5044 (ns↓)	0.9048±0.0858	1.0000 (ns↓)	0.3498±0.1384	0.5044 (ns↓)
TransMIL	0.8909±0.0842	0.2095 (ns↓)	0.6548±0.1352	<0.0001 (***↓)	0.3056±0.0867	0.7191 (ns↓)	0.3389±0.0839	0.5029 (ns↓)	0.8611±0.0481	0.5034 (ns↓)	0.2873±0.0717	0.5019 (ns↓)
MambaMIL	0.9222±0.0820	0.5720 (ns↓)	0.6548±0.1352	0.0315 (*↓)	0.2639±0.0636	0.3282 (ns↓)	0.2833±0.0577	0.3392 (ns↓)	0.8681±0.0601	0.5148 (ns↓)	0.2484±0.0203	0.2730 (ns↓)
MambaMIL+HiLA-MIL (Ours)	0.9342±0.0783	N/A	0.7440±0.1162	N/A	0.4000±0.0433	N/A	0.4139±0.0875	N/A	0.9067±0.0572	N/A	0.3833±0.0289	N/A

*：P<0.05；**：P<0.01；***：P<0.001；ns：no statistical significance.

↑: an increase in metrics of the baseline models compared with the MambaMIL+HiLA-MIL model.

↓: a decrease in metrics of the baseline models compared with the MambaMIL+HiLA-MIL model.

N/A: Not applicable. Statistical comparison was not performed.

A two-sided paired permutation test with 10,000 permutations was applied.

Supplementary Table 4. Evaluation metrics of multi-cancer and individual cancer types on NIMM, DLZX, and YDSY Datasets under UNI

NIMM

Methods	Multi-cancer											
	AUC	P-value	Accuracy	P-value	Precision	P-value	Recall	P-value	Specificity	P-value	F1-Score	P-value
ABMIL	0.9748±0.0044	0.0590 (ns↓)	0.8841±0.0255	<0.0001 (***↓)	0.5923±0.0307	0.0250 (*↓)	0.5778±0.0226	0.0055 (**↓)	0.9306±0.0271	0.0130 (*↓)	0.5757±0.0231	0.0146 (*↓)
CLAM-SB	0.9731±0.0101	0.0820 (ns↓)	0.8749±0.0190	<0.0001 (***↓)	0.5913±0.0255	0.0206 (*↓)	0.5849±0.0225	0.0112 (*↓)	0.9313±0.0333	0.0134 (*↓)	0.5796±0.0249	0.0181 (*↓)
CLAM-MB	0.9739±0.0086	0.0928 (ns↓)	0.8872±0.0137	<0.0001 (***↓)	0.5882±0.0292	0.0074 (**↓)	0.5851±0.0232	0.0017 (**↓)	0.9334±0.0295	0.0031 (**↓)	0.5786±0.0279	0.0031 (**↓)
DSMIL	0.9726±0.0088	0.0934 (ns↓)	0.8724±0.0151	<0.0001 (***↓)	0.6019±0.0178	0.0617 (ns↓)	0.5910±0.0219	0.0153 (*↓)	0.9297±0.0306	0.0089 (**↓)	0.5868±0.0194	0.0369 (*↓)
TransMIL	0.9750±0.0102	0.1616 (ns↓)	0.8859±0.0204	0.0002 (***↓)	0.5980±0.0252	0.1039 (ns↓)	0.5895±0.0217	0.0336 (*↓)	0.9332±0.0317	0.0127 (*↓)	0.5847±0.0187	0.0587 (ns↓)
MambaMIL	0.9721±0.0099	0.1733 (ns↓)	0.8774±0.0277	0.0002 (***↓)	0.5824±0.0401	0.0333 (*↓)	0.5776±0.0253	0.0107 (*↓)	0.9318±0.0350	0.0091 (**↓)	0.5721±0.0278	0.0226 (*↓)
MambaMIL+HiLA-MIL (Ours)	0.9798±0.0090	N/A	0.8971±0.0410	N/A	0.6474±0.0595	N/A	0.6390±0.0563	N/A	0.9384±0.0379	N/A	0.6340±0.0585	N/A

Methods	Gastric adenocarcinoma											
	AUC	P-value	Accuracy	P-value	Precision	P-value	Recall	P-value	Specificity	P-value	F1-Score	P-value
ABMIL	0.9891±0.0085	0.6263 (ns↓)	0.9303±0.0417	<0.0001 (***↓)	0.5948±0.1308	<0.0001 (***↓)	0.5421±0.0365	<0.0001 (***↓)	0.9747±0.0102	0.2543 (ns↓)	0.5539±0.0574	<0.0001 (***↓)
CLAM-SB	0.9893±0.0105	0.9867 (ns↓)	0.9303±0.0417	<0.0001 (***↓)	0.5480±0.0457	0.0158 (*↓)	0.5670±0.0682	0.5052 (ns↓)	0.9773±0.0149	0.0984 (ns↓)	0.5541±0.0526	0.3957 (ns↓)
CLAM-MB	0.9880±0.0112	0.6587 (ns↓)	0.9303±0.0417	<0.0001 (***↓)	0.5921±0.1263	0.1301 (ns↓)	0.5655±0.0659	0.5052 (ns↓)	0.9726±0.0111	0.0621 (ns↓)	0.5752±0.0906	0.5640 (ns↓)
DSMIL	0.9893±0.0104	0.9912 (ns↓)	0.9303±0.0417	<0.0001 (***↓)	0.5447±0.0510	0.0158 (*↓)	0.5670±0.0682	0.5052 (ns↓)	0.9744±0.0102	0.0819 (ns↓)	0.5524±0.0553	0.3957 (ns↓)
TransMIL	0.9853±0.0126	0.9852 (ns↓)	0.9237±0.0451	<0.0001 (***↓)	0.5392±0.0440	<0.0001 (***↓)	0.5377±0.0337	<0.0001 (***↓)	0.9729±0.0109	0.0653 (ns↓)	0.5377±0.0371	<0.0001 (***↓)
MambaMIL	0.9895±0.0094	0.8168 (ns↓)	0.9439±0.0304	0.2517 (ns↓)	0.5774±0.0791	0.1239 (ns↓)	0.5730±0.0725	0.5052 (ns↓)	0.9810±0.0118	0.4982 (ns↓)	0.5751±0.0757	0.7517 (ns↓)
MambaMIL+HiLA-MIL (Ours)	0.9911±0.0081	N/A	0.9526±0.0417	N/A	0.7025±0.0750	N/A	0.6316±0.1031	N/A	0.9834±0.0144	N/A	0.6517±0.0854	N/A

Methods	Colorectal adenocarcinoma											
	AUC	P-value	Accuracy	P-value	Precision	P-value	Recall	P-value	Specificity	P-value	F1-Score	P-value
ABMIL	0.9929±0.0095	0.2827 (ns↓)	0.9371±0.0417	<0.0001 (***↓)	0.6135±0.1082	<0.0001 (***↓)	0.6351±0.1404	<0.0001 (***↓)	0.9826±0.0130	0.1249 (ns↓)	0.6210±0.1190	<0.0001 (***↓)
CLAM-SB	0.9958±0.0061	0.9089 (ns↓)	0.9488±0.0394	0.0336 (*↓)	0.7284±0.1356	0.5681 (ns↓)	0.7351±0.1035	0.8132 (ns↓)	0.9855±0.0126	0.6581 (ns↓)	0.7038±0.1025	0.5748 (ns↓)
CLAM-MB	0.9931±0.0110	0.5544 (ns↓)	0.9382±0.0573	<0.0001 (***↓)	0.6823±0.0876	0.1229 (ns↓)	0.7155±0.0791	<0.0001 (***↓)	0.9819±0.0187	0.1264 (ns↓)	0.6756±0.0556	0.0614 (ns↓)
DSMIL	0.9960±0.0063	0.9251 (ns↓)	0.9488±0.0394	0.0671 (ns↓)	0.7394±0.1181	0.4950 (ns↓)	0.7351±0.1035	0.8132 (ns↓)	0.9835±0.0120	0.5989 (ns↓)	0.7162±0.0896	0.6141 (ns↓)
TransMIL	0.9946±0.0087	0.6149 (ns↓)	0.9435±0.0483	<0.0001 (***↓)	0.7021±0.0744	0.2530 (ns↓)	0.7047±0.0693	<0.0001 (***↓)	0.9806±0.0167	<0.0001 (***↓)	0.6839±0.0337	0.1265 (ns↓)
MambaMIL	0.9945±0.0071	0.7174 (ns↓)	0.9383±0.0573	<0.0001 (***↓)	0.6718±0.1895	<0.0001 (***↓)	0.6770±0.1884	<0.0001 (***↓)	0.9829±0.0170	0.1178 (ns↓)	0.6590±0.1672	<0.0001 (***↓)

MambaMIL+HiLA-MIL (Ours)	0.9964±0.0053	N/A	0.9617±0.0503	N/A	0.7885±0.0751	N/A	0.8047±0.0639	N/A	0.9890±0.0150	N/A	0.7799±0.0464	N/A

Methods	Lung adenocarcinoma											
	AUC	P-value	Accuracy	P-value	Precision	P-value	Recall	P-value	Specificity	P-value	F1-Score	P-value
ABMIL	0.9880±0.0134	0.8075 (ns↓)	0.9303±0.0480	<0.0001 (***↑)	0.7416±0.1178	<0.0001 (***↑)	0.6942±0.0603	<0.0001 (***↑)	0.9754±0.0170	<0.0001 (***↑)	0.6961±0.0687	<0.0001 (***↑)
CLAM-SB	0.9758±0.0181	0.7037 (ns↓)	0.9103±0.0548	1.0000 (ns↑)	0.6420±0.1335	0.6910 (ns↑)	0.6357±0.1388	0.4999 (ns↓)	0.9691±0.0188	1.0000 (ns↑)	0.6369±0.1347	0.6910 (ns↓)
CLAM-MB	0.9854±0.0228	0.9209 (ns↓)	0.9291±0.0714	<0.0001 (***↑)	0.6497±0.1409	0.8731 (ns↑)	0.6441±0.1375	0.8731 (ns↓)	0.9753±0.0251	0.1263 (ns↑)	0.6453±0.1382	0.8731 (ns↓)
DSMIL	0.9768±0.0192	0.7047 (ns↓)	0.9258±0.0598	<0.0001 (***↑)	0.6838±0.0613	0.1242 (ns↑)	0.6811±0.0622	0.1242 (ns↑)	0.9731±0.0203	0.4958 (ns↑)	0.6806±0.0604	0.1242 (ns↑)
TransMIL	0.9851±0.0218	0.9706 (ns↓)	0.9224±0.0605	<0.0001 (***↑)	0.6916±0.0594	<0.0001 (***↑)	0.6803±0.0500	<0.0001 (***↑)	0.9734±0.0206	0.4993 (ns↑)	0.6701±0.0239	<0.0001 (***↑)
MambaMIL	0.9787±0.0310	1.0000 (ns↓)	0.9069±0.0431	1.0000 (ns↑)	0.6184±0.1266	0.7430 (ns↓)	0.6348±0.1274	0.5020 (ns↓)	0.9684±0.0189	1.0000 (ns↓)	0.6143±0.1117	0.7430 (ns↓)
MambaMIL+HiLA-MIL (Ours)	0.9883±0.0165	N/A	0.9067±0.0643	N/A	0.6407±0.0374	N/A	0.6716±0.0547	N/A	0.9690±0.0226	N/A	0.6486±0.0334	N/A

Methods	Breast invasive ductal carcinoma											
	AUC	P-value	Accuracy	P-value	Precision	P-value	Recall	P-value	Specificity	P-value	F1-Score	P-value
ABMIL	0.9400±0.0243	0.0482 (*↓)	0.7781±0.0613	<0.0001 (***↓)	0.7029±0.0315	0.0512 (ns↓)	0.7220±0.0088	0.0194 (*↓)	0.9241±0.0235	0.0445 (*↓)	0.7085±0.0182	0.0507 (ns↓)
CLAM-SB	0.9451±0.0138	0.0818 (ns↓)	0.7678±0.0626	<0.0001 (***↓)	0.7202±0.0530	0.0405 (*↓)	0.7117±0.0682	0.0272 (*↓)	0.9217±0.0245	0.0152 (*↓)	0.7150±0.0611	0.0325 (*↓)
CLAM-MB	0.9402±0.0186	0.0414 (*↓)	0.7545±0.0710	<0.0001 (***↓)	0.6986±0.0478	0.0073 (**↓)	0.7090±0.0590	0.0143 (*↓)	0.9177±0.0275	0.0045 (**↓)	0.6995±0.0578	0.0073 (**↓)
DSMIL	0.9433±0.0150	0.0527 (ns↓)	0.7678±0.0626	<0.0001 (***↓)	0.7235±0.0586	0.0525 (ns↓)	0.7178±0.0779	0.0340 (*↓)	0.9215±0.0245	0.0154 (*↓)	0.7188±0.0674	0.0413 (*↓)
TransMIL	0.9465±0.0112	0.2565 (ns↓)	0.7895±0.0251	0.0128 (*↓)	0.7391±0.0454	0.2264 (ns↓)	0.7416±0.0533	0.1657 (ns↓)	0.9280±0.0136	0.1393 (ns↓)	0.7388±0.0478	0.2379 (ns↓)
MambaMIL	0.9392±0.0166	0.0291 (*↓)	0.7806±0.0771	0.0051 (**↓)	0.7263±0.0980	0.1409 (ns↓)	0.7280±0.0732	0.0729 (ns↓)	0.9239±0.0284	0.1182 (ns↓)	0.7241±0.0852	0.1215 (ns↓)
MambaMIL+HiLA-MIL (Ours)	0.9533±0.0196	N/A	0.8197±0.0700	N/A	0.7874±0.0613	N/A	0.7913±0.0576	N/A	0.9407±0.0230	N/A	0.7858±0.0645	N/A

Methods	Breast invasive lobular Carcinoma											
	AUC	P-value	Accuracy	P-value	Precision	P-value	Recall	P-value	Specificity	P-value	F1-Score	P-value
ABMIL	0.9641±0.0292	0.6974 (ns↓)	0.8449±0.0628	1.0000 (ns↓)	0.3089±0.1432	0.4972 (ns↓)	0.2958±0.1252	1.0000 (ns↓)	0.7958±0.1252	0.4972 (ns↓)	0.2989±0.1305	0.4972 (ns↓)
CLAM-SB	0.9596±0.0356	0.8719 (ns↓)	0.8172±0.0167	<0.0001 (***↓)	0.3182±0.1591	1.0000 (ns↓)	0.2750±0.0901	<0.0001 (***↓)	0.8028±0.1370	<0.0001 (***↓)	0.2880±0.1115	<0.0001 (***↓)
CLAM-MB	0.9629±0.0411	0.9914 (ns↓)	0.8838±0.1009	0.2440 (ns↑)	0.3182±0.1591	0.2440 (ns↓)	0.2917±0.0722	0.7522 (ns↓)	0.8194±0.1203	0.2440 (ns↑)	0.2972±0.1042	0.7522 (ns↓)
DSMIL	0.9573±0.0341	0.8376 (ns↓)	0.7894±0.0353	<0.0001 (***↓)	0.3182±0.1591	<0.0001 (***↓)	0.2542±0.0564	<0.0001 (***↓)	0.7958±0.1252	<0.0001 (***↓)	0.2657±0.0730	<0.0001 (***↓)
TransMIL	0.9633±0.0402	0.9881 (ns↓)	0.8505±0.0435	1.0000 (ns↑)	0.3182±0.1591	0.5018 (ns↓)	0.2833±0.0804	0.5018 (ns↓)	0.8111±0.1281	1.0000 (ns↑)	0.2928±0.1074	0.5018 (ns↓)
MambaMIL	0.9587±0.0263	0.7179 (ns↓)	0.8172±0.0167	<0.0001 (***↓)	0.3182±0.1591	0.5018 (ns↓)	0.2750±0.0901	<0.0001 (***↓)	0.8028±0.1370	0.5018 (ns↓)	0.2880±0.1115	0.5018 (ns↓)

MambaMIL+HiLA-MIL (Ours)	0.9698±0.0366	N/A	0.8449±0.0628	N/A	0.3182±0.1591	N/A	0.2958±0.1252	N/A	0.8097±0.1489	N/A	0.3038±0.1390	N/A

Supplementary Table 4. Continued

DLZX

Methods	Multi-cancer											
	AUC	P-value	Accuracy	P-value	Precision	P-value	Recall	P-value	Specificity	P-value	F1-Score	P-value
ABMIL	0.8362±0.2087	0.0001 (***↓)	0.6445±0.2876	<0.0001 (***↓)	0.4585±0.1252	0.0001 (***↓)	0.4161±0.0663	<0.0001 (***↓)	0.8750±0.1043	<0.0001 (***↓)	0.3891±0.1372	<0.0001 (***↓)
CLAM-SB	0.9533±0.0425	0.4544 (ns↓)	0.8311±0.1117	<0.0001 (***↓)	0.5126±0.0377	0.0495 (*↓)	0.4798±0.0209	0.3372 (ns↓)	0.9180±0.0366	0.3502 (ns↓)	0.4656±0.0271	0.1125 (ns↓)
CLAM-MB	0.9575±0.0219	0.6771 (ns↓)	0.8346±0.0531	0.0014 (**↓)	0.5170±0.0501	0.0285 (*↓)	0.4685±0.0116	0.0054 (**↓)	0.9249±0.0267	0.3834 (ns↑)	0.4748±0.0138	0.0082 (**↓)
DSMIL	0.9592±0.0239	0.6888 (ns↑)	0.8258±0.0636	0.0001 (***↓)	0.5208±0.0229	0.0378 (*↓)	0.4553±0.0057	0.0036 (**↓)	0.9189±0.0326	0.1851 (ns↓)	0.4698±0.0052	0.0046 (**↓)
TransMIL	0.9578±0.0232	0.4214 (ns↑)	0.8240±0.0626	<0.0001 (***↓)	0.5127±0.0368	0.0065 (**↓)	0.4677±0.0166	0.0054 (**↓)	0.9222±0.0270	0.3281 (ns↓)	0.4720±0.0015	0.0042 (**↓)
MambaMIL	0.9552±0.0231	0.5853 (ns↓)	0.8274±0.0774	<0.0001 (***↓)	0.4995±0.0541	0.0285 (*↓)	0.4409±0.0234	0.0006 (***↓)	0.9134±0.0387	0.0047 (**↓)	0.4511±0.0301	0.0018 (**↓)
MambaMIL+HiLA-MIL (Ours)	0.9578±0.0161	N/A	0.8584±0.0689	N/A	0.5350±0.0546	N/A	0.5054±0.0135	N/A	0.9230±0.0367	N/A	0.5068±0.0200	N/A

Methods	Gastric adenocarcinoma											
	AUC	P-value	Accuracy	P-value	Precision	P-value	Recall	P-value	Specificity	P-value	F1-Score	P-value
ABMIL	0.8401±0.1607	0.7618 (ns↓)	0.6274±0.2286	<0.0001 (***↓)	0.4831±0.2147	0.0151 (*↓)	0.4903±0.0808	<0.0001 (***↓)	0.8820±0.0599	0.0076 (**↓)	0.4388±0.1502	<0.0001 (***↓)
CLAM-SB	0.9027±0.1132	0.9831 (ns↓)	0.6760±0.2156	<0.0001 (***↓)	0.5503±0.1073	0.0900 (ns↓)	0.5200±0.0748	0.2449 (ns↓)	0.8895±0.0571	0.0150 (*↓)	0.4842±0.1216	0.0900 (ns↓)
CLAM-MB	0.9356±0.0476	0.3280 (ns↑)	0.8024±0.0536	0.2537 (ns↑)	0.6149±0.0512	0.3761 (ns↑)	0.5825±0.0821	0.7537 (ns↑)	0.9324±0.0113	0.2537 (ns↑)	0.5691±0.0372	0.3761 (ns↑)
DSMIL	0.9306±0.0624	0.9974 (ns↑)	0.7593±0.0878	0.3773 (ns↓)	0.6145±0.0604	0.9349 (ns↑)	0.5257±0.0573	0.3151 (ns↓)	0.9146±0.0229	0.6829 (ns↓)	0.5395±0.0497	0.6207 (ns↓)
TransMIL	0.9286±0.0612	1.0000 (ns↑)	0.7593±0.0878	0.2539 (ns↓)	0.5957±0.0668	0.8738 (ns↑)	0.5555±0.0767	0.2539 (ns↓)	0.9205±0.0197	0.8738 (ns↓)	0.5442±0.0518	0.7444 (ns↓)
MambaMIL	0.9202±0.0616	0.8138 (ns↑)	0.7607±0.1210	0.2554 (ns↓)	0.6248±0.0849	0.8764 (ns↑)	0.5319±0.0819	0.2554 (ns↓)	0.9104±0.0287	0.3766 (ns↓)	0.5426±0.0804	0.6206 (ns↓)
MambaMIL+HiLA-MIL (Ours)	0.9151±0.0490	N/A	0.7815±0.0863	N/A	0.5935±0.0737	N/A	0.5756±0.0928	N/A	0.9217±0.0209	N/A	0.5602±0.0684	N/A

Methods	Colorectal adenocarcinoma											
	AUC	P-value	Accuracy	P-value	Precision	P-value	Recall	P-value	Specificity	P-value	F1-Score	P-value
ABMIL	0.9280±0.0829	0.6551 (ns↓)	0.7895±0.2133	<0.0001 (***↓)	0.5961±0.2139	<0.0001 (***↓)	0.6117±0.2003	<0.0001 (***↓)	0.9333±0.0513	<0.0001 (***↓)	0.5600±0.2514	<0.0001 (***↓)
CLAM-SB	0.9742±0.0033	0.9824 (ns↓)	0.9271±0.0394	0.0418 (*↓)	0.6357±0.1425	0.0901 (ns↓)	0.6967±0.2203	0.9147 (ns↓)	0.9736±0.0055	0.6951 (ns↓)	0.6416±0.1403	0.2599 (ns↓)
CLAM-MB	0.9624±0.0157	0.4481 (ns↓)	0.9120±0.0179	<0.0001 (***↓)	0.6500±0.2157	<0.0001 (***↓)	0.6548±0.1839	<0.0001 (***↓)	0.9647±0.0025	<0.0001 (***↓)	0.6493±0.1989	<0.0001 (***↓)
DSMIL	0.9730±0.0120	1.0000 (ns↓)	0.9199±0.0166	<0.0001 (***↓)	0.6770±0.1954	0.1308 (ns↓)	0.6589±0.1769	0.0998 (ns↓)	0.9667±0.0065	0.0621 (ns↓)	0.6649±0.1840	0.0998 (ns↓)
TransMIL	0.9717±0.0126	0.9952 (ns↓)	0.9127±0.0106	<0.0001 (***↓)	0.6600±0.1911	0.0348 (*↓)	0.6789±0.1777	0.0653 (ns↓)	0.9664±0.0060	0.0496 (*↓)	0.6674±0.1843	0.0653 (ns↓)

MambaMIL	0.9680±0.0172	0.9353 (ns↓)	0.8982±0.0270	<0.0001 (***↓)	0.5854±0.2230	0.0875 (ns↓)	0.5996±0.2110	<0.0001 (***↓)	0.9568±0.0106	<0.0001 (***↓)	0.5911±0.2182	0.0145 (*↓)
MambaMIL+HiLA-MIL (Ours)	0.9745±0.0141	N/A	0.9467±0.0086	N/A	0.7056±0.2501	N/A	0.6973±0.1841	N/A	0.9765±0.0066	N/A	0.6926±0.2053	N/A

Methods	Breast invasive ductal carcinoma											
	AUC	P-value	Accuracy	P-value	Precision	P-value	Recall	P-value	Specificity	P-value	F1-Score	P-value
ABMIL	0.8544±0.2013	0.0008 (***↓)	0.6860±0.3019	<0.0001 (***↓)	0.5046±0.0153	0.2650 (ns↑)	0.3542±0.0651	0.9276 (ns↓)	0.8994±0.0658	0.0389 (*↓)	0.3356±0.0695	0.5439 (ns↓)
CLAM-SB	0.9546±0.0324	0.7326 (ns↓)	0.8416±0.0738	<0.0001 (***↓)	0.4617±0.0597	0.7172 (ns↑)	0.3507±0.0709	0.1228 (ns↓)	0.9398±0.0157	0.7869 (ns↑)	0.3739±0.0553	0.2488 (ns↓)
CLAM-MB	0.9719±0.0073	0.8347 (ns↑)	0.8527±0.0577	0.2478 (ns↓)	0.4696±0.0460	0.7507 (ns↑)	0.3542±0.0651	0.2478 (ns↓)	0.9432±0.0176	0.5507 (ns↑)	0.3801±0.0554	0.4372 (ns↓)
DSMIL	0.9669±0.0096	0.7184 (ns↓)	0.8527±0.0577	0.2478 (ns↓)	0.4582±0.0657	0.6547 (ns↑)	0.3542±0.0651	0.2478 (ns↓)	0.9349±0.0137	0.8728 (ns↑)	0.3741±0.0511	0.2821 (ns↓)
TransMIL	0.9660±0.0107	0.3835 (ns↓)	0.8527±0.0577	0.2478 (ns↓)	0.4617±0.0597	0.7491 (ns↑)	0.3542±0.0651	0.2478 (ns↓)	0.9426±0.0169	0.6270 (ns↑)	0.3758±0.0522	0.3133 (ns↓)
MambaMIL	0.9647±0.0121	0.7837 (ns↓)	0.8423±0.0627	<0.0001 (***↓)	0.4544±0.0722	0.7953 (ns↑)	0.3403±0.0514	0.1247 (ns↓)	0.9175±0.0357	0.2722 (ns↓)	0.3651±0.0483	0.1863 (ns↓)
MambaMIL+HiLA-MIL (Ours)	0.9674±0.0101	N/A	0.8629±0.0700	N/A	0.4384±0.0469	N/A	0.4062±0.0890	N/A	0.9337±0.0129	N/A	0.4175±0.0734	N/A

Methods	Breast invasive lobular Carcinoma											
	AUC	P-value	Accuracy	P-value	Precision	P-value	Recall	P-value	Specificity	P-value	F1-Score	P-value
ABMIL	0.7222±0.3989	<0.0001 (***↓)	0.4750±0.4206	<0.0001 (***↓)	0.2500±0.2500	<0.0001 (***↓)	0.2083±0.1909	<0.0001 (***↓)	0.7854±0.2481	0.1095 (ns↓)	0.2222±0.2097	<0.0001 (***↓)
CLAM-SB	0.9816±0.0250	0.9071 (ns↑)	0.8796±0.1253	<0.0001 (***↑)	0.4028±0.1339	<0.0001 (***↓)	0.3519±0.1398	<0.0001 (***↑)	0.8692±0.1396	<0.0001 (***↑)	0.3625±0.1327	1.0000 (ns↑)
CLAM-MB	0.9602±0.0467	0.5062 (ns↓)	0.7713±0.1343	0.2575 (ns↓)	0.3333±0.1443	0.1284 (ns↓)	0.2824±0.0814	0.2575 (ns↓)	0.8595±0.1209	0.4985 (ns↓)	0.3007±0.1007	0.1284 (ns↓)
DSMIL	0.9664±0.0433	0.6830 (ns↓)	0.7713±0.1343	0.2508 (ns↓)	0.3333±0.1443	0.3764 (ns↓)	0.2824±0.0814	0.2508 (ns↓)	0.8595±0.1209	0.4999 (ns↓)	0.3007±0.1007	0.1880 (ns↓)
TransMIL	0.9650±0.0420	0.5547 (ns↓)	0.7713±0.1343	0.2508 (ns↓)	0.3333±0.1443	0.2508 (ns↓)	0.2824±0.0814	0.2508 (ns↓)	0.8595±0.1209	0.4999 (ns↓)	0.3007±0.1007	0.1275 (ns↓)
MambaMIL	0.9678±0.0448	0.9257 (ns↓)	0.8083±0.1876	1.0000 (ns↓)	0.3333±0.1443	0.5065 (ns↓)	0.2917±0.0722	0.3809 (ns↓)	0.8688±0.1051	0.3160 (ns↑)	0.3056±0.0962	0.2541 (ns↓)
MambaMIL+HiLA-MIL (Ours)	0.9740±0.0290	N/A	0.8426±0.1370	N/A	0.4028±0.1339	N/A	0.3426±0.1530	N/A	0.8600±0.1544	N/A	0.3570±0.1407	N/A

Supplementary Table 4. Continued

YDSY

Methods	Multi-cancer											
	AUC	P-value	Accuracy	P-value	Precision	P-value	Recall	P-value	Specificity	P-value	F1-Score	P-value
ABMIL	0.9241±0.0199	0.0142 (*↓)	0.7718±0.0542	0.0003 (***↓)	0.5080±0.0614	0.1397 (ns↓)	0.5012±0.0674	0.0574 (ns↓)	0.9010±0.0568	0.1850 (ns↓)	0.4981±0.0651	0.0668 (ns↓)
CLAM-SB	0.8996±0.0487	0.0559 (ns↓)	0.7569±0.0327	<0.0001 (***↓)	0.5008±0.0302	0.2131 (ns↓)	0.4849±0.0506	0.0095 (**↓)	0.8955±0.0469	0.0475 (*↓)	0.4780±0.0454	0.0261 (*↓)
CLAM-MB	0.9256±0.0211	0.3913 (ns↓)	0.7589±0.0392	0.0010 (**↓)	0.4845±0.0520	0.2237 (ns↓)	0.4841±0.0515	0.0389 (*↓)	0.8838±0.0440	0.1442 (ns↓)	0.4768±0.0569	0.0690 (ns↓)
DSMIL	0.8938±0.0380	0.0138 (*↓)	0.7498±0.0391	<0.0001 (***↓)	0.5183±0.0440	0.0282 (*↓)	0.5021±0.0423	0.0522 (ns↓)	0.9002±0.0293	0.0439 (*↓)	0.4886±0.0368	0.0278 (*↓)

TransMIL	0.9109±0.0488	0.0438 (*↓)	0.7329±0.0535	<0.0001 (***↓)	0.5109±0.0210	0.0191 (*↓)	0.4761±0.0288	0.0211 (*↓)	0.8971±0.0225	0.0753 (ns↓)	0.4819±0.0220	0.0132 (*↓)
MambaMIL	0.9295±0.0222	0.5671 (ns↓)	0.7584±0.0134	0.0003 (***↓)	0.5031±0.0336	0.2238 (ns↓)	0.4807±0.0214	0.0162 (*↓)	0.8909±0.0330	0.0910 (ns↓)	0.4797±0.0258	0.0364 (*↓)
MambaMIL+HiLA-MIL (Ours)	0.9444±0.0244	N/A	0.8197±0.0627	N/A	0.5571±0.0883	N/A	0.5619±0.1007	N/A	0.9150±0.0536	N/A	0.5477±0.0935	N/A

Methods	Gastric adenocarcinoma											
	AUC	P-value	Accuracy	P-value	Precision	P-value	Recall	P-value	Specificity	P-value	F1-Score	P-value
ABMIL	0.9156±0.0758	<0.0001 (***↑)	0.7333±0.3055	1.0000 (ns↓)	0.3750±0.2165	1.0000 (ns↓)	0.3611±0.1735	1.0000 (ns↓)	0.8722±0.1798	1.0000 (ns↓)	0.3643±0.1934	1.0000 (ns↓)
CLAM-SB	0.8147±0.1758	0.1834 (ns↓)	0.6500±0.2179	<0.0001 (***↓)	0.3750±0.2165	1.0000 (ns↓)	0.3194±0.1339	<0.0001 (***↓)	0.8514±0.1601	<0.0001 (***↓)	0.3365±0.1685	<0.0001 (***↓)
CLAM-MB	0.8578±0.1295	0.8093 (ns↓)	0.6000±0.3464	<0.0001 (***↓)	0.2500±0.2165	0.5005 (ns↓)	0.2778±0.1925	<0.0001 (***↓)	0.7889±0.1836	<0.0001 (***↓)	0.2619±0.2062	<0.0001 (***↓)
DSMIL	0.8078±0.0964	<0.0001 (***↓)	0.7167±0.1041	1.0000 (ns↓)	0.4306±0.1203	0.5018 (ns↑)	0.3611±0.0636	1.0000 (ns↓)	0.8931±0.0880	0.5018 (ns↑)	0.3861±0.0835	0.5018 (ns↑)
TransMIL	0.8103±0.1629	0.1071 (ns↓)	0.5167±0.2843	<0.0001 (***↓)	0.3333±0.2205	0.2582 (ns↓)	0.2500±0.1502	<0.0001 (***↓)	0.7931±0.1628	<0.0001 (***↓)	0.2778±0.1735	<0.0001 (***↓)
MambaMIL	0.8656±0.0693	0.3737 (ns↓)	0.5833±0.1756	<0.0001 (***↓)	0.3472±0.1969	0.4991 (ns↓)	0.2778±0.1049	<0.0001 (***↓)	0.8097±0.1361	<0.0001 (***↓)	0.2976±0.1404	<0.0001 (***↓)
MambaMIL+HiLA-MIL (Ours)	0.8978±0.1069	N/A	0.7333±0.3055	N/A	0.3750±0.2165	N/A	0.3611±0.1735	N/A	0.8722±0.1798	N/A	0.3643±0.1934	N/A

Methods	Colorectal adenocarcinoma											
	AUC	P-value	Accuracy	P-value	Precision	P-value	Recall	P-value	Specificity	P-value	F1-Score	P-value
ABMIL	0.9451±0.0234	0.1676 (ns↓)	0.8885±0.0083	1.0000 (ns↓)	0.6779±0.0410	0.7053 (ns↑)	0.6334±0.0385	0.6554 (ns↑)	0.9533±0.0120	0.6760 (ns↓)	0.6495±0.0060	0.8387 (ns↑)
CLAM-SB	0.9732±0.0168	0.5317 (ns↓)	0.8771±0.0552	0.2468 (ns↓)	0.6525±0.0813	0.5048 (ns↓)	0.5887±0.0416	0.2468 (ns↓)	0.9428±0.0183	0.2468 (ns↓)	0.5989±0.0587	0.2468 (ns↓)
CLAM-MB	0.9855±0.0123	0.7897 (ns↓)	0.9151±0.0390	0.0301 (*↑)	0.7021±0.0489	1.0000 (ns↑)	0.6431±0.0521	0.3441 (ns↑)	0.9590±0.0169	0.4399 (ns↑)	0.6615±0.0303	0.4399 (ns↑)
DSMIL	0.9521±0.0447	0.4415 (ns↓)	0.7967±0.1333	<0.0001 (***↓)	0.6074±0.1010	0.0180 (*↓)	0.5580±0.0225	0.2191 (ns↓)	0.9265±0.0248	0.0817 (ns↓)	0.5482±0.0590	0.0713 (ns↓)
TransMIL	0.9796±0.0077	0.4057 (ns↓)	0.8771±0.0552	0.4537 (ns↓)	0.6837±0.0949	0.7281 (ns↑)	0.5881±0.0407	0.3747 (ns↓)	0.9501±0.0266	0.9767 (ns↓)	0.6186±0.0695	0.5411 (ns↓)
MambaMIL	0.9900±0.0109	0.9540 (ns↑)	0.8905±0.0728	1.0000 (ns↓)	0.6785±0.0897	0.7604 (ns↑)	0.6000±0.0601	0.4981 (ns↓)	0.9485±0.0238	0.9062 (ns↓)	0.6223±0.0746	0.5278 (ns↓)
MambaMIL+HiLA-MIL (Ours)	0.9895±0.0129	N/A	0.8905±0.0728	N/A	0.6751±0.0589	N/A	0.6239±0.0478	N/A	0.9535±0.0231	N/A	0.6420±0.0553	N/A

Methods	Lung adenocarcinoma											
	AUC	P-value	Accuracy	P-value	Precision	P-value	Recall	P-value	Specificity	P-value	F1-Score	P-value
ABMIL	0.8581±0.0553	0.4682 (ns↓)	0.6530±0.1200	0.0183 (*↓)	0.3886±0.0734	0.1596 (ns↓)	0.4278±0.0855	0.0789 (ns↓)	0.8626±0.0392	0.1362 (ns↓)	0.3991±0.0681	0.1646 (ns↓)
CLAM-SB	0.8145±0.0440	0.1770 (ns↓)	0.6416±0.0257	0.0431 (*↓)	0.4254±0.1472	0.1865 (ns↓)	0.4250±0.0433	0.0924 (ns↓)	0.8543±0.0216	0.1907 (ns↓)	0.4013±0.0716	0.1173 (ns↓)
CLAM-MB	0.8630±0.0743	0.1008 (ns↓)	0.6768±0.1240	<0.0001 (***↓)	0.4282±0.1188	0.4300 (ns↓)	0.4500±0.1014	<0.0001 (***↓)	0.8802±0.0363	0.3704 (ns↓)	0.4204±0.0820	0.3704 (ns↓)
DSMIL	0.7981±0.1158	0.2489 (ns↓)	0.5305±0.1713	<0.0001 (***↓)	0.3722±0.0694	0.0560 (ns↓)	0.3750±0.0750	0.0243 (*↓)	0.8276±0.0410	0.0394 (*↓)	0.3345±0.1104	0.0406 (*↓)

Methods	AUC	P-value	Accuracy	P-value	Precision	P-value	Recall	P-value	Specificity	P-value	F1-Score	P-value
TransMIL	0.8583±0.0877	0.3322 (ns↓)	0.6549±0.1087	0.0172 (*↓)	0.4220±0.1022	0.0693 (ns↓)	0.4250±0.0661	0.0811 (ns↓)	0.8592±0.0258	0.3073 (ns↓)	0.4129±0.0716	0.0597 (ns↓)
MambaMIL	0.8891±0.0682	0.8605 (ns↓)	0.7025±0.0818	0.2416 (ns↓)	0.4365±0.1240	0.2416 (ns↓)	0.4639±0.0774	0.2416 (ns↓)	0.8683±0.0361	0.2416 (ns↓)	0.4291±0.0879	0.2416 (ns↓)
MambaMIL+HiLA-MIL (Ours)	0.9022±0.0416	N/A	0.7281±0.0444	N/A	0.5294±0.0887	N/A	0.5333±0.0441	N/A	0.8852±0.0136	N/A	0.5140±0.0648	N/A

Methods	Breast invasive ductal carcinoma											
	AUC	P-value	Accuracy	P-value	Precision	P-value	Recall	P-value	Specificity	P-value	F1-Score	P-value
ABMIL	0.9665±0.0005	0.8736 (ns↓)	0.8459±0.0590	0.0315 (*↓)	0.7444±0.0951	0.8125 (ns↓)	0.7255±0.1328	0.5686 (ns↓)	0.9522±0.0123	0.5246 (ns↓)	0.7237±0.0977	0.6004 (ns↓)
CLAM-SB	0.9740±0.0103	0.7450 (ns↑)	0.8778±0.0434	1.0000 (ns↓)	0.7386±0.1070	0.8128 (ns↓)	0.7582±0.1044	1.0000 (ns↓)	0.9610±0.0148	1.0000 (ns↓)	0.7318±0.0934	0.8401 (ns↓)
CLAM-MB	0.9722±0.0052	0.5957 (ns↑)	0.8644±0.0482	0.2466 (ns↓)	0.7434±0.0375	0.2466 (ns↓)	0.7166±0.0327	0.2466 (ns↓)	0.9506±0.0223	0.2466 (ns↓)	0.7263±0.0319	0.2466 (ns↓)
DSMIL	0.9646±0.0147	0.8395 (ns↓)	0.8778±0.0434	1.0000 (ns↓)	0.7437±0.0922	1.0000 (ns↓)	0.7582±0.1044	1.0000 (ns↓)	0.9615±0.0135	1.0000 (ns↓)	0.7416±0.0927	0.8766 (ns↓)
TransMIL	0.9657±0.0053	0.5832 (ns↓)	0.8778±0.0434	1.0000 (ns↓)	0.7407±0.0870	0.6315 (ns↓)	0.7285±0.0531	0.6256 (ns↓)	0.9597±0.0143	0.6910 (ns↓)	0.7281±0.0698	0.8743 (ns↓)
MambaMIL	0.9706±0.0039	0.9312 (ns↑)	0.8778±0.0434	1.0000 (ns↓)	0.7407±0.0870	1.0000 (ns↓)	0.7285±0.0531	0.4940 (ns↓)	0.9597±0.0143	0.4940 (ns↓)	0.7281±0.0698	1.0000 (ns↓)
MambaMIL+HiLA-MIL (Ours)	0.9691±0.0090	N/A	0.8778±0.0434	N/A	0.7684±0.0610	N/A	0.7910±0.1003	N/A	0.9615±0.0127	N/A	0.7579±0.0528	N/A

Methods	Breast invasive lobular Carcinoma											
	AUC	P-value	Accuracy	P-value	Precision	P-value	Recall	P-value	Specificity	P-value	F1-Score	P-value
ABMIL	0.9350±0.0121	0.2754 (ns↓)	0.7381±0.0206	<0.0001 (***↓)	0.3542±0.2366	<0.0001 (***↓)	0.3583±0.1876	<0.0001 (***↓)	0.8646±0.0994	<0.0001 (***↓)	0.3538±0.2115	<0.0001 (***↓)
CLAM-SB	0.9216±0.0585	0.5253 (ns↓)	0.7381±0.0206	<0.0001 (***↓)	0.3125±0.1654	<0.0001 (***↓)	0.3333±0.1443	<0.0001 (***↓)	0.8681±0.1028	<0.0001 (***↓)	0.3214±0.1557	<0.0001 (***↓)
CLAM-MB	0.9494±0.0314	0.4987 (ns↓)	0.7381±0.0206	<0.0001 (***↓)	0.2986±0.1747	0.6242 (ns↓)	0.3333±0.1443	<0.0001 (***↓)	0.8403±0.0939	<0.0001 (***↓)	0.3139±0.1613	0.1269 (ns↓)
DSMIL	0.9466±0.0147	0.4793 (ns↓)	0.8274±0.0676	0.2511 (ns↓)	0.4375±0.2864	1.0000 (ns↓)	0.4583±0.1909	0.2511 (ns↓)	0.8924±0.1234	0.4968 (ns↓)	0.4325±0.2266	0.4968 (ns↓)
TransMIL	0.9407±0.0208	0.6101 (ns↓)	0.7381±0.0206	<0.0001 (***↓)	0.3750±0.1250	0.1212 (ns↓)	0.3889±0.1273	0.1212 (ns↓)	0.9236±0.0120	0.7507 (ns↑)	0.3722±0.1251	0.1212 (ns↓)
MambaMIL	0.9320±0.0314	0.3779 (ns↓)	0.7381±0.0206	<0.0001 (***↓)	0.3125±0.1654	<0.0001 (***↓)	0.3333±0.1443	<0.0001 (***↓)	0.8681±0.1028	<0.0001 (***↓)	0.3214±0.1557	<0.0001 (***↓)
MambaMIL+HiLA-MIL (Ours)	0.9633±0.0326	N/A	0.8690±0.1254	N/A	0.4375±0.2864	N/A	0.5000±0.2500	N/A	0.9028±0.1339	N/A	0.4603±0.2705	N/A

*：P<0.05；**：P<0.01；***：P<0.001；ns：no statistical significance.

↑: an increase in metrics of the baseline models compared with the MambaMIL+HiLA-MIL model.

↓: a decrease in metrics of the baseline models compared with the MambaMIL+HiLA-MIL model.

N/A: Not applicable. Statistical comparison was not performed.

A two-sided paired permutation test with 10,000 permutations was applied.

Supplementary Table 5. Evaluation metrics of multi-cancer and individual cancer types on NIMM, DLZX, and YDSY Datasets under Virchow

NIMM

Methods	Multi-cancer											
	AUC	P-value	Accuracy	P-value	Precision	P-value	Recall	P-value	Specificity	P-value	F1-Score	P-value
ABMIL	0.9661±0.0092	0.2350 (ns↓)	0.8656±0.0187	0.0015 (**↓)	0.5718±0.0445	0.0087 (**↓)	0.5573±0.0185	0.0018 (**↓)	0.9220±0.0206	0.0667 (ns↓)	0.5549±0.0237	0.0039 (**↓)
CLAM-SB	0.9600±0.0067	0.0532 (ns↓)	0.8603±0.0106	<0.0001 (***↓)	0.5624±0.0202	0.0065 (**↓)	0.5614±0.0244	0.0234 (*↓)	0.9287±0.0242	0.1741 (ns↓)	0.5533±0.0215	0.0046 (**↓)
CLAM-MB	0.9689±0.0088	0.2689 (ns↓)	0.8665±0.0184	0.0199 (*↓)	0.5700±0.0238	0.1344 (ns↓)	0.5476±0.0125	0.1089 (ns↓)	0.9294±0.0305	0.3525 (ns↓)	0.5464±0.0088	0.0929 (ns↓)
DSMIL	0.9699±0.0074	0.1504 (ns↓)	0.8692±0.0325	<0.0001 (***↓)	0.6252±0.0352	0.0740 (ns↑)	0.6040±0.0374	0.1997 (ns↓)	0.9299±0.0332	0.1634 (ns↓)	0.5905±0.0381	0.0562 (ns↓)
TransMIL	0.9695±0.0128	0.3581 (ns↓)	0.8705±0.0531	0.0010 (**↓)	0.6146±0.0692	0.0890 (ns↑)	0.6020±0.0533	0.3211 (ns↓)	0.9316±0.0396	0.4172 (ns↓)	0.5828±0.0612	0.1107 (ns↓)
MambaMIL	0.9627±0.0079	0.4170 (ns↓)	0.8575±0.0311	0.0167 (*↓)	0.5963±0.0287	0.0856 (ns↓)	0.5733±0.0136	0.0188 (*↓)	0.9225±0.0267	0.1328 (ns↓)	0.5761±0.0210	0.0313 (*↓)
MambaMIL+HiLA-MIL (Ours)	0.9766±0.0126	N/A	0.8857±0.0290	N/A	0.6008±0.0388	N/A	0.6218±0.0387	N/A	0.9342±0.0321	N/A	0.6030±0.0375	N/A

Methods	Gastric adenocarcinoma											
	AUC	P-value	Accuracy	P-value	Precision	P-value	Recall	P-value	Specificity	P-value	F1-Score	P-value
ABMIL	0.9910±0.0094	1.0000 (ns↓)	0.9449±0.0533	<0.0001 (***↓)	0.6567±0.0938	1.0000 (ns↑)	0.6547±0.0927	<0.0001 (***↓)	0.9835±0.0146	1.0000 (ns↓)	0.6551±0.0941	1.0000 (ns↓)
CLAM-SB	0.9902±0.0108	0.9407 (ns↓)	0.9368±0.0412	<0.0001 (***↓)	0.5734±0.0794	0.5089 (ns↓)	0.5714±0.0749	<0.0001 (***↓)	0.9815±0.0112	0.5089 (ns↓)	0.5718±0.0772	0.5089 (ns↓)
CLAM-MB	0.9907±0.0099	0.9996 (ns↓)	0.9368±0.0412	<0.0001 (***↓)	0.6012±0.1251	1.0000 (ns↓)	0.5714±0.0749	<0.0001 (***↓)	0.9816±0.0112	0.5089 (ns↓)	0.5829±0.0952	0.5089 (ns↓)
DSMIL	0.9847±0.0144	0.9645 (ns↓)	0.9111±0.0785	<0.0001 (***↓)	0.6843±0.1056	0.3623 (ns↑)	0.6397±0.1020	0.2426 (ns↓)	0.9749±0.0218	0.0543 (ns↓)	0.6392±0.0977	0.3594 (ns↓)
TransMIL	0.9798±0.0176	0.6995 (ns↓)	0.8899±0.1066	<0.0001 (***↓)	0.6801±0.1128	0.1786 (ns↑)	0.6305±0.1058	0.2426 (ns↓)	0.9693±0.0285	0.0087 (**↓)	0.6286±0.1106	0.2076 (ns↓)
MambaMIL	0.9889±0.0113	0.7519 (ns↓)	0.9193±0.0785	0.0006 (***↓)	0.6495±0.0874	0.9779 (ns↓)	0.6164±0.1171	0.1556 (ns↓)	0.9707±0.0266	0.0514 (ns↓)	0.6292±0.1056	0.5385 (ns↓)
MambaMIL+HiLA-MIL (Ours)	0.9969±0.0044	N/A	0.9515±0.0538	N/A	0.6542±0.0938	N/A	0.6825±0.1023	N/A	0.9872±0.0141	N/A	0.6635±0.0950	N/A

Methods	Colorectal adenocarcinoma											
	AUC	P-value	Accuracy	P-value	Precision	P-value	Recall	P-value	Specificity	P-value	F1-Score	P-value
ABMIL	0.9879±0.0081	0.8911 (ns↓)	0.9059±0.0285	<0.0001 (***↓)	0.5131±0.0523	0.1078 (ns↓)	0.5160±0.0568	0.0961 (ns↓)	0.9654±0.0095	0.1369 (ns↓)	0.5141±0.0546	0.1073 (ns↓)
CLAM-SB	0.9883±0.0108	0.8192 (ns↓)	0.9190±0.0440	0.0781 (ns↓)	0.5797±0.0842	0.3730 (ns↓)	0.5994±0.1301	0.2240 (ns↓)	0.9745±0.0104	0.5519 (ns↓)	0.5865±0.1024	0.5908 (ns↓)
CLAM-MB	0.9921±0.0095	0.8917 (ns↑)	0.9124±0.0353	0.0152 (*↓)	0.5298±0.0742	0.1611 (ns↓)	0.5313±0.0730	0.0808 (ns↓)	0.9709±0.0065	0.3619 (ns↓)	0.5266±0.0671	0.1916 (ns↓)
DSMIL	0.9877±0.0110	0.6464 (ns↓)	0.9422±0.0380	0.1161 (ns↑)	0.7196±0.1324	0.8976 (ns↑)	0.7147±0.0807	0.8738 (ns↑)	0.9822±0.0118	0.3605 (ns↑)	0.6621±0.0458	0.8838 (ns↑)
TransMIL	0.9918±0.0124	0.8581 (ns↑)	0.9360±0.0285	0.5396 (ns↑)	0.6392±0.1739	0.6161 (ns↑)	0.6740±0.1598	0.6916 (ns↓)	0.9826±0.0093	0.4541 (ns↑)	0.6085±0.1062	0.8278 (ns↓)
MambaMIL	0.9944±0.0071	0.8285 (ns↑)	0.9371±0.0417	0.5056 (ns↑)	0.6296±0.1333	0.6856 (ns↑)	0.5966±0.0860	0.5111 (ns↓)	0.9776±0.0086	0.8844 (ns↑)	0.6042±0.0982	0.6785 (ns↓)

MambaMIL+HiLA-MIL (Ours)	0.9906±0.0080	N/A	0.9305±0.0375	N/A	0.6069±0.0443	N/A	0.6994±0.0590	N/A	0.9764±0.0106	N/A	0.6350±0.0429	N/A

Methods	Lung adenocarcinoma											
	AUC	P-value	Accuracy	P-value	Precision	P-value	Recall	P-value	Specificity	P-value	F1-Score	P-value
ABMIL	0.9860±0.0018	0.9905 (ns↓)	0.9081±0.0599	<0.0001 (***↑)	0.6735±0.1097	0.0710 (ns↑)	0.6569±0.0936	0.0151 (*↑)	0.9680±0.0183	0.0930 (ns↑)	0.6530±0.0953	0.0416 (*↑)
CLAM-SB	0.9900±0.0018	0.7153 (ns↑)	0.9115±0.0376	<0.0001 (***↑)	0.6078±0.1228	0.3598 (ns↓)	0.6569±0.1385	0.2604 (ns↑)	0.9730±0.0093	0.0671 (ns↑)	0.6195±0.1247	0.3911 (ns↑)
CLAM-MB	0.9859±0.0070	0.9837 (ns↓)	0.8893±0.0605	<0.0001 (***↑)	0.6407±0.1551	0.3782 (ns↑)	0.6114±0.1520	0.5006 (ns↓)	0.9608±0.0185	0.2965 (ns↑)	0.6172±0.1452	0.7077 (ns↑)
DSMIL	0.9897±0.0040	0.8288 (ns↑)	0.8958±0.0367	<0.0001 (***↑)	0.6585±0.0786	<0.0001 (***↑)	0.6400±0.0698	<0.0001 (***↑)	0.9617±0.0053	0.2587 (ns↑)	0.6392±0.0619	<0.0001 (***↑)
TransMIL	0.9912±0.0086	0.9382 (ns↑)	0.9113±0.0606	<0.0001 (***↑)	0.6720±0.0909	0.0887 (ns↑)	0.6566±0.0800	<0.0001 (***↑)	0.9697±0.0159	<0.0001 (***↑)	0.6531±0.0709	0.0295 (*↑)
MambaMIL	0.9828±0.0170	1.0000 (ns↓)	0.9430±0.0392	<0.0001 (***↑)	0.7717±0.1878	0.0008 (***↑)	0.7222±0.1049	0.0006 (***↑)	0.9792±0.0092	0.0193 (*↑)	0.7244±0.1356	0.0008 (***↑)
MambaMIL+HiLA-MIL (Ours)	0.9880±0.0029	N/A	0.8690±0.0369	N/A	0.6118±0.0591	N/A	0.6267±0.0687	N/A	0.9536±0.0073	N/A	0.6158±0.0632	N/A

Methods	Breast invasive ductal carcinoma											
	AUC	P-value	Accuracy	P-value	Precision	P-value	Recall	P-value	Specificity	P-value	F1-Score	P-value
ABMIL	0.9274±0.0173	0.0141 (*↓)	0.7796±0.0477	<0.0001 (***↓)	0.7141±0.0286	0.0183 (*↓)	0.7048±0.0433	0.0059 (**↓)	0.9250±0.0102	0.0549 (ns↓)	0.6957±0.0389	0.0096 (**↓)
CLAM-SB	0.9226±0.0435	0.0020 (**↓)	0.7447±0.1058	<0.0001 (***↓)	0.7328±0.0686	0.0035 (**↓)	0.7149±0.0812	0.0005 (***↓)	0.9188±0.0316	0.0051 (**↓)	0.7064±0.0912	0.0008 (***↓)
CLAM-MB	0.9373±0.0094	0.0997 (ns↓)	0.8045±0.0308	0.0774 (ns↓)	0.7602±0.0806	0.2529 (ns↓)	0.7695±0.0867	0.2607 (ns↓)	0.9376±0.0090	0.5492 (ns↓)	0.7399±0.0614	0.2467 (ns↓)
DSMIL	0.9249±0.0115	0.0228 (*↓)	0.7521±0.0079	<0.0001 (***↓)	0.7453±0.0609	0.0596 (ns↓)	0.7298±0.0544	0.0463 (*↓)	0.9210±0.0080	0.0255 (*↓)	0.7080±0.0290	0.0201 (*↓)
TransMIL	0.9392±0.0234	0.1593 (ns↓)	0.7703±0.0263	0.0002 (***↓)	0.7636±0.0496	0.1766 (ns↓)	0.7533±0.0360	0.1953 (ns↓)	0.9268±0.0014	0.1235 (ns↓)	0.7199±0.0399	0.0989 (ns↓)
MambaMIL	0.9328±0.0209	0.1104 (ns↓)	0.7597±0.0289	<0.0001 (***↓)	0.7051±0.0486	0.0190 (*↓)	0.7064±0.0471	0.0154 (*↓)	0.9182±0.0109	0.0327 (*↓)	0.7006±0.0426	0.0168 (*↓)
MambaMIL+HiLA-MIL (Ours)	0.9566±0.0126	N/A	0.8326±0.0529	N/A	0.8129±0.0636	N/A	0.8048±0.0441	N/A	0.9439±0.0143	N/A	0.7968±0.0657	N/A

Methods	Breast invasive lobular Carcinoma											
	AUC	P-value	Accuracy	P-value	Precision	P-value	Recall	P-value	Specificity	P-value	F1-Score	P-value
ABMIL	0.9383±0.0529	0.6546 (ns↓)	0.7894±0.0353	<0.0001 (***↓)	0.3015±0.1306	<0.0001 (***↓)	0.2542±0.0564	<0.0001 (***↓)	0.7681±0.0787	<0.0001 (***↓)	0.2565±0.0570	<0.0001 (***↓)
CLAM-SB	0.9088±0.0277	<0.0001 (***↓)	0.7894±0.0353	<0.0001 (***↓)	0.3182±0.1591	0.5045 (ns↓)	0.2646±0.0730	<0.0001 (***↓)	0.7958±0.1252	<0.0001 (***↓)	0.2824±0.1018	<0.0001 (***↓)
CLAM-MB	0.9386±0.0539	0.1867 (ns↓)	0.7894±0.0353	<0.0001 (***↓)	0.3182±0.1591	<0.0001 (***↓)	0.2542±0.0564	<0.0001 (***↓)	0.7958±0.1252	<0.0001 (***↓)	0.2657±0.0730	<0.0001 (***↓)
DSMIL	0.9626±0.0420	0.9541 (ns↑)	0.8449±0.0628	1.0000 (ns↓)	0.3182±0.1591	1.0000 (ns↓)	0.2958±0.1252	1.0000 (ns↓)	0.8097±0.1489	1.0000 (ns↓)	0.3038±0.1390	1.0000 (ns↓)
TransMIL	0.9455±0.0532	0.8920 (ns↓)	0.8449±0.0628	1.0000 (ns↓)	0.3182±0.1591	1.0000 (ns↓)	0.2958±0.1252	1.0000 (ns↓)	0.8097±0.1489	1.0000 (ns↓)	0.3038±0.1390	1.0000 (ns↓)
MambaMIL	0.9147±0.0383	0.2194 (ns↓)	0.7283±0.0796	<0.0001 (***↓)	0.2256±0.0229	<0.0001 (***↓)	0.2250±0.0433	<0.0001 (***↓)	0.7667±0.1010	<0.0001 (***↓)	0.2221±0.0149	<0.0001 (***↓)

MambaMIL+HiLA-MIL (Ours)	0.9510±0.0674	N/A	0.8449±0.0628	N/A	0.3182±0.1591	N/A	0.2958±0.1252	N/A	0.8097±0.1489	N/A	0.3038±0.1390	N/A

Supplementary Table 5. Continued

DLZX

Methods	Multi-cancer											
	AUC	P-value	Accuracy	P-value	Precision	P-value	Recall	P-value	Specificity	P-value	F1-Score	P-value
ABMIL	0.9014±0.0196	0.4928 (ns↓)	0.7532±0.0463	0.0068 (**↓)	0.4827±0.0228	0.2067 (ns↓)	0.4443±0.0229	0.3924 (ns↓)	0.8873±0.0351	0.2260 (ns↓)	0.4340±0.0389	0.2640 (ns↓)
CLAM-SB	0.9125±0.0146	0.3805 (ns↓)	0.7914±0.0117	0.0126 (*↓)	0.4830±0.0534	0.5491 (ns↓)	0.4175±0.0078	0.1511 (ns↓)	0.8939±0.0299	0.0376 (*↓)	0.4278±0.0192	0.2487 (ns↓)
CLAM-MB	0.9147±0.0278	0.2243 (ns↓)	0.7464±0.0729	<0.0001 (***↓)	0.4734±0.0785	0.0525 (ns↓)	0.4243±0.0447	0.0209 (*↓)	0.8813±0.0366	0.0005 (***↓)	0.4300±0.0668	0.0149 (*↓)
DSMIL	0.9175±0.0193	0.3553 (ns↓)	0.7634±0.0221	<0.0001 (***↓)	0.4784±0.0521	0.0126 (*↓)	0.4312±0.0103	0.0034 (**↓)	0.8934±0.0290	0.0465 (*↓)	0.4353±0.0245	0.0052 (**↓)
TransMIL	0.9189±0.0205	0.2271 (ns↓)	0.7595±0.0166	<0.0001 (***↓)	0.4579±0.0891	0.0003 (***↓)	0.4090±0.0579	0.0004 (***↓)	0.8923±0.0336	0.0104 (*↓)	0.4170±0.0612	0.0002 (***↓)
MambaMIL	0.9143±0.0049	0.3878 (ns↓)	0.7714±0.0283	0.0067 (**↓)	0.4649±0.0204	0.1725 (ns↓)	0.4386±0.0184	0.2255 (ns↓)	0.8937±0.0221	0.0657 (ns↓)	0.4355±0.0083	0.1892 (ns↓)
MambaMIL+HiLA-MIL (Ours)	0.9309±0.0161	N/A	0.7965±0.0279	N/A	0.5267±0.0418	N/A	0.4562±0.0139	N/A	0.9047±0.0291	N/A	0.4704±0.0129	N/A

Methods	Gastric adenocarcinoma											
	AUC	P-value	Accuracy	P-value	Precision	P-value	Recall	P-value	Specificity	P-value	F1-Score	P-value
ABMIL	0.8761±0.0297	0.9808 (ns↓)	0.7577±0.0612	<0.0001 (***↑)	0.5333±0.1301	0.7227 (ns↓)	0.5242±0.1449	0.4941 (ns↑)	0.9084±0.0288	0.4278 (ns↑)	0.5030±0.1169	0.7212 (ns↓)
CLAM-SB	0.8888±0.0569	0.9888 (ns↓)	0.7133±0.0636	1.0000 (ns↓)	0.5319±0.0938	0.2481 (ns↓)	0.5006±0.0288	1.0000 (ns↓)	0.8892±0.0184	0.5050 (ns↓)	0.4923±0.0408	0.5050 (ns↓)
CLAM-MB	0.8767±0.0694	1.0000 (ns↓)	0.6492±0.1296	<0.0001 (***↓)	0.5235±0.1231	0.1252 (ns↓)	0.4346±0.1357	<0.0001 (***↓)	0.8726±0.0466	0.0658 (ns↓)	0.4568±0.1159	0.0658 (ns↓)
DSMIL	0.8881±0.0514	1.0000 (ns↓)	0.6687±0.0447	<0.0001 (***↓)	0.5387±0.0996	0.4209 (ns↓)	0.4700±0.0283	0.1210 (ns↓)	0.8865±0.0185	0.6113 (ns↓)	0.4747±0.0434	0.2917 (ns↓)
TransMIL	0.8955±0.0428	0.6589 (ns↓)	0.6895±0.0239	0.3745 (ns↓)	0.5089±0.1492	0.7569 (ns↓)	0.4589±0.0387	0.3794 (ns↓)	0.8866±0.0185	0.6138 (ns↓)	0.4639±0.0732	0.5694 (ns↓)
MambaMIL	0.9188±0.0462	1.0000 (ns↑)	0.7563±0.0268	<0.0001 (***↑)	0.5731±0.0626	0.2476 (ns↑)	0.5277±0.0152	<0.0001 (***↑)	0.9023±0.0084	0.4901 (ns↑)	0.5317±0.0072	0.2476 (ns↑)
MambaMIL+HiLA-MIL (Ours)	0.9012±0.0390	N/A	0.7147±0.0820	N/A	0.5589±0.0915	N/A	0.5034±0.0571	N/A	0.8949±0.0274	N/A	0.5061±0.0614	N/A

Methods	Colorectal adenocarcinoma											
	AUC	P-value	Accuracy	P-value	Precision	P-value	Recall	P-value	Specificity	P-value	F1-Score	P-value
ABMIL	0.9557±0.0261	0.9596 (ns↓)	0.9133±0.0439	0.4586 (ns↓)	0.6388±0.1521	0.8188 (ns↓)	0.6613±0.1421	0.6876 (ns↑)	0.9668±0.0156	0.8758 (ns↓)	0.6104±0.1097	0.4598 (ns↓)
CLAM-SB	0.9359±0.0211	0.9072 (ns↓)	0.8836±0.0581	0.0013 (**↓)	0.5412±0.0604	0.0928 (ns↓)	0.5426±0.0436	0.0443 (*↓)	0.9564±0.0142	0.1108 (ns↓)	0.5326±0.0422	0.0450 (*↓)
CLAM-MB	0.9220±0.0230	0.2018 (ns↓)	0.8274±0.0892	<0.0001 (***↓)	0.5597±0.0679	0.0002 (***↓)	0.5893±0.1183	<0.0001 (***↓)	0.9436±0.0281	0.0004 (***↓)	0.5593±0.0794	<0.0001 (***↓)
DSMIL	0.9371±0.0179	0.6008 (ns↓)	0.8854±0.0162	<0.0001 (***↓)	0.6272±0.1865	0.0663 (ns↓)	0.6231±0.1614	0.0364 (*↓)	0.9580±0.0011	0.0942 (ns↓)	0.5950±0.1335	0.0196 (*↓)
TransMIL	0.9341±0.0244	0.3737 (ns↓)	0.8491±0.0769	<0.0001 (***↓)	0.5850±0.2152	0.0056 (**↓)	0.5560±0.2055	0.0023 (**↓)	0.9459±0.0217	0.0095 (**↓)	0.5427±0.1678	0.0009 (***↓)

MambaMIL	0.9461±0.0235	0.4821 (ns↓)	0.8860±0.0211	<0.0001 (***↓)	0.6179±0.1552	0.0869 (ns↓)	0.6231±0.1449	0.0397 (*↓)	0.9570±0.0099	0.0897 (ns↓)	0.5858±0.1026	0.0324 (*↓)
MambaMIL+HiLA-MIL (Ours)	0.9627±0.0039	N/A	0.9198±0.0327	N/A	0.6720±0.1792	N/A	0.6585±0.1662	N/A	0.9682±0.0093	N/A	0.6615±0.1691	N/A

Methods	Breast invasive ductal carcinoma											
	AUC	P-value	Accuracy	P-value	Precision	P-value	Recall	P-value	Specificity	P-value	F1-Score	P-value
ABMIL	0.9574±0.0150	0.7966 (ns↓)	0.8223±0.0557	<0.0001 (***↓)	0.4394±0.0314	<0.0001 (***↓)	0.3835±0.1013	<0.0001 (***↓)	0.8949±0.0390	0.1899 (ns↓)	0.3831±0.0720	0.2528 (ns↓)
CLAM-SB	0.9512±0.0200	0.6423 (ns↓)	0.8223±0.0557	<0.0001 (***↓)	0.4561±0.0452	0.3489 (ns↓)	0.3445±0.0556	0.2547 (ns↓)	0.8939±0.0381	0.1145 (ns↓)	0.3683±0.0583	0.3314 (ns↓)
CLAM-MB	0.9624±0.0150	0.8516 (ns↓)	0.8414±0.0884	1.0000 (ns↓)	0.5007±0.1073	0.9343 (ns↑)	0.4279±0.1718	0.4998 (ns↑)	0.9102±0.0241	0.7222 (ns↓)	0.4393±0.1601	0.6876 (ns↑)
DSMIL	0.9609±0.0127	0.7857 (ns↓)	0.8318±0.0720	<0.0001 (***↓)	0.4145±0.0733	0.1328 (ns↓)	0.3862±0.1053	1.0000 (ns↓)	0.8956±0.0415	0.0672 (ns↓)	0.3931±0.0997	0.3172 (ns↓)
TransMIL	0.9647±0.0111	0.7145 (ns↓)	0.8318±0.0720	<0.0001 (***↓)	0.4044±0.0772	0.0791 (ns↓)	0.3758±0.1070	<0.0001 (***↓)	0.9032±0.0485	0.5374 (ns↓)	0.3829±0.1010	0.1252 (ns↓)
MambaMIL	0.9665±0.0120	0.6804 (ns↓)	0.8423±0.0627	1.0000 (ns↓)	0.4184±0.0729	0.3772 (ns↓)	0.4001±0.1079	0.5021 (ns↑)	0.8986±0.0439	0.2870 (ns↓)	0.4017±0.1011	0.8819 (ns↑)
MambaMIL+HiLA-MIL (Ours)	0.9689±0.0106	N/A	0.8423±0.0627	N/A	0.4592±0.0441	N/A	0.3897±0.1055	N/A	0.9116±0.0246	N/A	0.3940±0.0762	N/A

Methods	Breast invasive lobular Carcinoma											
	AUC	P-value	Accuracy	P-value	Precision	P-value	Recall	P-value	Specificity	P-value	F1-Score	P-value
ABMIL	0.8166±0.0946	0.0076 (**↓)	0.5194±0.1617	<0.0001 (***↓)	0.3194±0.1577	0.0294 (*↓)	0.2083±0.1102	<0.0001 (***↓)	0.7792±0.1711	0.0294 (*↓)	0.2396±0.1213	0.0294 (*↓)
CLAM-SB	0.8743±0.0332	0.7435 (ns↓)	0.7463±0.1445	0.2478 (ns↑)	0.4028±0.1339	0.2544 (ns↓)	0.2824±0.0561	0.4963 (ns↑)	0.8359±0.0987	0.8087 (ns↓)	0.3181±0.0721	0.4963 (ns↓)
CLAM-MB	0.8975±0.0279	0.1242 (ns↑)	0.6676±0.0962	<0.0001 (***↓)	0.3095±0.1688	1.0000 (ns↓)	0.2454±0.0488	<0.0001 (***↓)	0.7988±0.1028	<0.0001 (***↓)	0.2646±0.0886	0.5085 (ns↓)
DSMIL	0.8839±0.0120	0.6543 (ns↓)	0.6676±0.0962	<0.0001 (***↓)	0.3333±0.1443	0.7516 (ns↓)	0.2454±0.0488	<0.0001 (***↓)	0.8336±0.1205	0.7516 (ns↓)	0.2785±0.0780	0.7516 (ns↓)
TransMIL	0.8813±0.0229	0.6602 (ns↓)	0.6676±0.0962	0.2483 (ns↓)	0.3333±0.1443	0.3761 (ns↓)	0.2454±0.0488	0.2483 (ns↓)	0.8336±0.1205	0.8727 (ns↓)	0.2785±0.0780	0.3761 (ns↓)
MambaMIL	0.8257±0.0603	0.7246 (ns↓)	0.6009±0.1900	<0.0001 (***↓)	0.2500±0.0000	0.2463 (ns↓)	0.2037±0.0424	<0.0001 (***↓)	0.8169±0.1097	0.2463 (ns↓)	0.2229±0.0253	0.2463 (ns↓)
MambaMIL+HiLA-MIL (Ours)	0.8905±0.0141	N/A	0.7093±0.0956	N/A	0.4167±0.1443	N/A	0.2731±0.0713	N/A	0.8440±0.1309	N/A	0.3201±0.0879	N/A

Supplementary Table 5. Continued

YDSY

Methods	Multi-cancer											
	AUC	P-value	Accuracy	P-value	Precision	P-value	Recall	P-value	Specificity	P-value	F1-Score	P-value
ABMIL	0.8881±0.0342	0.1761 (ns↓)	0.6926±0.0864	<0.0001 (***↓)	0.5142±0.0578	0.0067 (**↓)	0.4908±0.0580	0.0062 (**↓)	0.8892±0.0313	0.0181 (*↓)	0.4776±0.0636	0.0039 (**↓)
CLAM-SB	0.9042±0.0128	0.7280 (ns↓)	0.7169±0.0589	0.0002 (***↓)	0.5359±0.0438	0.0997 (ns↓)	0.5055±0.0241	0.0809 (ns↓)	0.8945±0.0126	0.0764 (ns↓)	0.5053±0.0253	0.0741 (ns↓)
CLAM-MB	0.8710±0.0259	0.0274 (*↓)	0.6651±0.0948	<0.0001 (***↓)	0.4598±0.0660	0.0332 (*↓)	0.4505±0.0558	0.0029 (**↓)	0.8833±0.0122	0.0036 (**↓)	0.4361±0.0599	0.0033 (**↓)
DSMIL	0.8978±0.0102	0.3931 (ns↓)	0.7256±0.0386	<0.0001 (***↓)	0.5126±0.0149	0.0025 (**↓)	0.4985±0.0401	0.0707 (ns↓)	0.9001±0.0161	0.1052 (ns↓)	0.4848±0.0198	0.0053 (**↓)

TransMIL	0.8993±0.0213	0.6593 (ns↓)	0.7334±0.0216	0.0085 (**↓)	0.5096±0.0388	0.1965 (ns↓)	0.4879±0.0509	0.0313 (*↓)	0.8767±0.0113	0.0525 (ns↓)	0.4809±0.0372	0.0568 (ns↓)
MambaMIL	0.8998±0.0272	0.8638 (ns↓)	0.7183±0.0537	0.0031 (**↓)	0.5060±0.0382	0.0746 (ns↓)	0.4780±0.0227	0.0179 (*↓)	0.8808±0.0096	0.0996 (ns↓)	0.4770±0.0243	0.0296 (*↓)
MambaMIL+HiLA-MIL (Ours)	0.9163±0.0325	N/A	0.7869±0.0493	N/A	0.5753±0.0738	N/A	0.5606±0.0580	N/A	0.9227±0.0106	N/A	0.5527±0.0652	N/A

Methods	Gastric adenocarcinoma											
	AUC	P-value	Accuracy	P-value	Precision	P-value	Recall	P-value	Specificity	P-value	F1-Score	P-value
ABMIL	0.8039±0.1936	0.2653 (ns↓)	0.5833±0.3329	<0.0001 (***↓)	0.3611±0.2406	<0.0001 (***↓)	0.3056±0.1577	<0.0001 (***↓)	0.8486±0.1649	<0.0001 (***↓)	0.3222±0.1932	<0.0001 (***↓)
CLAM-SB	0.8167±0.1225	0.2049 (ns↓)	0.5000±0.1000	<0.0001 (***↓)	0.3056±0.0962	0.2513 (ns↓)	0.2500±0.0417	<0.0001 (***↓)	0.8139±0.0674	<0.0001 (***↓)	0.2694±0.0567	<0.0001 (***↓)
CLAM-MB	0.7425±0.1490	<0.0001 (***↓)	0.4333±0.0577	<0.0001 (***↓)	0.2083±0.0417	<0.0001 (***↓)	0.2083±0.0417	<0.0001 (***↓)	0.7903±0.0734	<0.0001 (***↓)	0.2000±0.0000	<0.0001 (***↓)
DSMIL	0.8103±0.1534	<0.0001 (***↓)	0.6500±0.2179	<0.0001 (***↓)	0.4167±0.1443	1.0000 (ns↓)	0.3333±0.1102	<0.0001 (***↓)	0.8764±0.1168	<0.0001 (***↓)	0.3639±0.1214	<0.0001 (***↓)
TransMIL	0.7869±0.1274	<0.0001 (***↓)	0.5667±0.0577	<0.0001 (***↓)	0.2917±0.1250	0.5006 (ns↓)	0.2778±0.0241	<0.0001 (***↓)	0.8097±0.0168	<0.0001 (***↓)	0.2750±0.0682	<0.0001 (***↓)
MambaMIL	0.7514±0.1441	<0.0001 (***↓)	0.5667±0.0577	<0.0001 (***↓)	0.3194±0.0867	0.5006 (ns↓)	0.2778±0.0241	<0.0001 (***↓)	0.8306±0.0419	<0.0001 (***↓)	0.2917±0.0417	<0.0001 (***↓)
MambaMIL+HiLA-MIL (Ours)	0.8567±0.0584	N/A	0.7167±0.1041	N/A	0.4306±0.1203	N/A	0.3611±0.0636	N/A	0.8931±0.0880	N/A	0.3861±0.0835	N/A

Methods	Colorectal adenocarcinoma											
	AUC	P-value	Accuracy	P-value	Precision	P-value	Recall	P-value	Specificity	P-value	F1-Score	P-value
ABMIL	0.9522±0.0300	0.4786 (ns↓)	0.8009±0.0607	0.0011 (**↓)	0.5839±0.0193	0.2035 (ns↓)	0.5490±0.0321	0.1427 (ns↓)	0.9206±0.0230	0.4722 (ns↓)	0.5539±0.0266	0.1578 (ns↓)
CLAM-SB	0.9734±0.0176	0.9114 (ns↑)	0.8780±0.0368	0.5434 (ns↑)	0.7450±0.1676	0.6857 (ns↑)	0.6808±0.1288	0.6287 (ns↑)	0.9423±0.0328	0.7444 (ns↑)	0.6985±0.1285	0.7649 (ns↑)
CLAM-MB	0.9469±0.0183	0.8778 (ns↓)	0.7673±0.0390	<0.0001 (***↓)	0.5569±0.0965	0.0135 (*↓)	0.5206±0.0231	0.0333 (*↓)	0.9177±0.0127	0.1502 (ns↓)	0.5012±0.0429	0.0176 (*↓)
DSMIL	0.9651±0.0257	0.9699 (ns↓)	0.8399±0.0546	0.0694 (ns↓)	0.6690±0.0673	0.8950 (ns↑)	0.6212±0.1260	0.9544 (ns↓)	0.9370±0.0159	0.8542 (ns↓)	0.6096±0.0524	0.9862 (ns↓)
TransMIL	0.9572±0.0231	0.8632 (ns↓)	0.8523±0.0337	0.5134 (ns↓)	0.6359±0.0683	0.8143 (ns↓)	0.5564±0.0137	0.2643 (ns↓)	0.9344±0.0277	0.8951 (ns↓)	0.5741±0.0155	0.3540 (ns↓)
MambaMIL	0.9815±0.0082	0.7864 (ns↑)	0.8523±0.0337	0.5514 (ns↓)	0.6210±0.0396	0.8593 (ns↓)	0.5820±0.0284	0.5273 (ns↓)	0.9424±0.0228	0.7183 (ns↑)	0.5953±0.0305	0.6854 (ns↓)
MambaMIL+HiLA-MIL (Ours)	0.9678±0.0198	N/A	0.8639±0.1068	N/A	0.6462±0.0864	N/A	0.6238±0.1037	N/A	0.9413±0.0437	N/A	0.6231±0.0916	N/A

Methods	Lung adenocarcinoma											
	AUC	P-value	Accuracy	P-value	Precision	P-value	Recall	P-value	Specificity	P-value	F1-Score	P-value
ABMIL	0.8666±0.0100	0.5742 (ns↑)	0.6284±0.0802	0.3734 (ns↓)	0.5026±0.0485	0.5616 (ns↓)	0.4861±0.0347	0.6257 (ns↓)	0.8627±0.0354	0.6922 (ns↓)	0.4798±0.0356	0.5005 (ns↓)
CLAM-SB	0.8886±0.0367	0.4921 (ns↑)	0.6549±0.1087	0.5070 (ns↑)	0.5222±0.0647	0.6716 (ns↓)	0.4819±0.0806	0.7033 (ns↓)	0.8654±0.0317	0.5259 (ns↓)	0.4810±0.0830	0.7954 (ns↓)
CLAM-MB	0.7834±0.0873	0.4291 (ns↓)	0.5191±0.2583	<0.0001 (***↓)	0.3319±0.2217	0.2974 (ns↓)	0.3875±0.1205	0.1423 (ns↓)	0.8310±0.0704	0.0948 (ns↓)	0.3309±0.1671	0.1101 (ns↓)
DSMIL	0.8610±0.0251	0.9495 (ns↑)	0.5940±0.0854	0.0126 (*↓)	0.4665±0.0563	0.2040 (ns↓)	0.4444±0.0883	0.1096 (ns↓)	0.8502±0.0203	0.2066 (ns↓)	0.4464±0.0781	0.2211 (ns↓)

TransMIL	0.8795±0.0300	0.4979 (ns↑)	0.7061±0.1167	0.4610 (ns↑)	0.5514±0.1204	0.9677 (ns↑)	0.5125±0.0976	0.6788 (ns↑)	0.8825±0.0325	1.0000 (ns↑)	0.5128±0.0949	0.6183 (ns↑)
MambaMIL	0.8913±0.0267	0.5154 (ns↑)	0.6911±0.0238	0.4522 (ns↑)	0.5417±0.0551	0.7917 (ns↑)	0.5167±0.0144	0.6769 (ns↑)	0.8894±0.0083	0.7971 (ns↑)	0.5097±0.0195	0.7813 (ns↑)
MambaMIL+HiLA-MIL (Ours)	0.8411±0.1084	N/A	0.6427±0.1738	N/A	0.5292±0.1003	N/A	0.5042±0.0711	N/A	0.8665±0.0622	N/A	0.4934±0.0905	N/A

Methods	Breast invasive ductal carcinoma											
	AUC	P-value	Accuracy	P-value	Precision	P-value	Recall	P-value	Specificity	P-value	F1-Score	P-value
ABMIL	0.9201±0.0438	0.4558 (ns↓)	0.7896±0.0883	<0.0001 (***↓)	0.6871±0.0190	0.5301 (ns↓)	0.6356±0.0842	0.0291 (*↓)	0.9249±0.0234	0.0480 (*↓)	0.6311±0.0614	0.1034 (ns↓)
CLAM-SB	0.9254±0.0389	0.3517 (ns↓)	0.7659±0.1204	<0.0001 (***↓)	0.6276±0.0928	0.1902 (ns↓)	0.6233±0.0800	0.0653 (ns↓)	0.9168±0.0479	0.0366 (*↓)	0.6133±0.0937	0.1295 (ns↓)
CLAM-MB	0.9365±0.0235	0.9857 (ns↓)	0.7844±0.0582	0.0150 (*↓)	0.6603±0.0475	0.3541 (ns↓)	0.6279±0.0668	0.2176 (ns↓)	0.9258±0.0218	0.1740 (ns↓)	0.6255±0.0533	0.2238 (ns↓)
DSMIL	0.9349±0.0420	0.9066 (ns↓)	0.8059±0.0648	0.0307 (*↓)	0.6442±0.0600	0.3589 (ns↓)	0.6600±0.0204	0.3506 (ns↓)	0.9343±0.0244	0.2559 (ns↓)	0.6323±0.0529	0.4118 (ns↓)
TransMIL	0.9366±0.0450	0.9847 (ns↓)	0.7978±0.1433	0.0164 (*↓)	0.6538±0.0777	0.5171 (ns↓)	0.6427±0.0960	0.1291 (ns↓)	0.9235±0.0572	0.0681 (ns↓)	0.6334±0.1059	0.3213 (ns↓)
MambaMIL	0.9507±0.0112	0.5323 (ns↑)	0.7793±0.0600	0.0064 (**↓)	0.6451±0.0299	0.2475 (ns↓)	0.6054±0.0847	0.1087 (ns↓)	0.9184±0.0253	0.0779 (ns↓)	0.5990±0.0956	0.1447 (ns↓)
MambaMIL+HiLA-MIL (Ours)	0.9423±0.0375	N/A	0.8481±0.0925	N/A	0.7704±0.1562	N/A	0.7642±0.1862	N/A	0.9543±0.0269	N/A	0.7546±0.1781	N/A

Methods	Breast invasive lobular Carcinoma											
	AUC	P-value	Accuracy	P-value	Precision	P-value	Recall	P-value	Specificity	P-value	F1-Score	P-value
ABMIL	0.8979±0.0726	0.0154 (*↓)	0.6607±0.1812	<0.0001 (***↓)	0.4361±0.1435	0.0610 (ns↓)	0.4778±0.1347	0.0610 (ns↓)	0.8889±0.0636	0.0610 (ns↓)	0.4008±0.1435	0.0610 (ns↓)
CLAM-SB	0.9170±0.1104	0.0593 (ns↓)	0.7857±0.2575	<0.0001 (***↓)	0.4792±0.0955	0.1222 (ns↓)	0.4917±0.0144	0.1222 (ns↓)	0.9340±0.0809	0.2473 (ns↓)	0.4643±0.0429	0.1222 (ns↓)
CLAM-MB	0.9455±0.0477	0.5220 (ns↓)	0.8214±0.1557	<0.0001 (***↓)	0.5417±0.0722	0.2530 (ns↑)	0.5083±0.0629	<0.0001 (***↓)	0.9519±0.0417	0.7479 (ns↓)	0.5231±0.0656	1.0000 (ns↑)
DSMIL	0.9176±0.0993	0.0728 (ns↓)	0.7381±0.2508	<0.0001 (***↓)	0.3667±0.1202	0.2439 (ns↓)	0.4333±0.0764	<0.0001 (***↓)	0.9028±0.1049	<0.0001 (***↓)	0.3717±0.1180	<0.0001 (***↓)
TransMIL	0.9364±0.0804	0.2106 (ns↓)	0.7440±0.1162	<0.0001 (***↓)	0.4153±0.2096	0.6235 (ns↓)	0.4500±0.1803	0.1230 (ns↓)	0.8333±0.0722	0.0605 (ns↓)	0.4091±0.1712	0.3138 (ns↓)
MambaMIL	0.9239±0.0791	0.0241 (*↓)	0.7024±0.1833	<0.0001 (***↓)	0.4028±0.1936	0.3420 (ns↓)	0.4083±0.1377	0.0616 (ns↓)	0.8229±0.0651	0.0605 (ns↓)	0.3892±0.1515	0.1573 (ns↓)
MambaMIL+HiLA-MIL (Ours)	0.9737±0.0242	N/A	0.8631±0.1432	N/A	0.5000±0.1667	N/A	0.5500±0.1323	N/A	0.9583±0.0417	N/A	0.5065±0.1626	N/A

*：P<0.05；**：P<0.01；***：P<0.001；ns：no statistical significance.

↑: an increase in metrics of the baseline models compared with the MambaMIL+HiLA-MIL model.

↓: a decrease in metrics of the baseline models compared with the MambaMIL+HiLA-MIL model.

N/A: Not applicable. Statistical comparison was not performed.

A two-sided paired permutation test with 10,000 permutations was applied.
