# Supplementary material for: Weakly supervised artificial intelligence for multi-cancer detection of lymph node metastasis on whole slide images
Source: Nat Commun. 2026 Aug 14;17:9770. doi: 10.1038/s41467-026-76410-w (PMC13572452; doi:10.1038/s41467-026-76410-w)
Supplement: Supplementary file 2 [file 41467_2026_76410_MOESM2_ESM.pdf]

nature portfolio

Corresponding author(s): Qi Jia, Yanmei Zhu

Last updated by author(s): Jul 28, 2026

Reporting Summary

Nature Portfolio wishes to improve the reproducibility of the work that we publish. This form provides structure for consistency and transparency in reporting. For further information on Nature Portfolio policies, see our Editorial Policies and the Editorial Policy Checklist.

Statistics

For all statistical analyses, confirm that the following items are present in the figure legend, table legend, main text, or Methods section.

n/a	Confirmed	
☐	☒	The exact sample size (n) for each experimental group/condition, given as a discrete number and unit of measurement
☐	☒	A statement on whether measurements were taken from distinct samples or whether the same sample was measured repeatedly
☐	☒	The statistical test(s) used AND whether they are one- or two-sided<br>*Only common tests should be described solely by name; describe more complex techniques in the Methods section.*
☐	☒	A description of all covariates tested
☐	☒	A description of any assumptions or corrections, such as tests of normality and adjustment for multiple comparisons
☐	☒	A full description of the statistical parameters including central tendency (e.g. means) or other basic estimates (e.g. regression coefficient) AND variation (e.g. standard deviation) or associated estimates of uncertainty (e.g. confidence intervals)
☐	☒	For null hypothesis testing, the test statistic (e.g. F, t, r) with confidence intervals, effect sizes, degrees of freedom and P value noted<br>*Give P values as exact values whenever suitable.*
☒	☐	For Bayesian analysis, information on the choice of priors and Markov chain Monte Carlo settings
☐	☒	For hierarchical and complex designs, identification of the appropriate level for tests and full reporting of outcomes
☒	☐	Estimates of effect sizes (e.g. Cohen's d, Pearson's r), indicating how they were calculated

Our web collection on statistics for biologists contains articles on many of the points above.

Software and code

Policy information about availability of computer code

Data collection	In this study, whole-slide digital images are acquired using a Hamamatsu digital slide scanner. Hamamatsu NDP.view2 (v2.9.25) is used for image viewing and screenshot export. Adobe Illustrator 2024 (v28.0) and BioRender are adopted for figure preparation.
Data analysis	The full source code for implementing the HiLA-MIL framework is publicly available in the GitHub repository (https://github.com/YAOSL98/HiLA-MIL) under the MIT License and archived on Zenodo (DOI: 10.5281/zenodo.21028097). The slide preprocessing modules in our codebase adapt from the open-source CLAM framework (https://github.com/mahmoodlab/CLAM), and the original license and copyright statements are preserved in compliance with its MIT License. All computational and statistical analyses are performed using Python (v3.10). Stratified k-fold cross-validation and all evaluation metrics are calculated via the StratifiedKFold and Metrics functions implemented in scikit-learn (v1.3.2). A two-sided paired permutation test with 10,000 permutations serves to assess statistical significance of performance differences across competing models.

For manuscripts utilizing custom algorithms or software that are central to the research but not yet described in published literature, software must be made available to editors and reviewers. We strongly encourage code deposition in a community repository (e.g. GitHub). See the Nature Portfolio guidelines for submitting code & software for further information.

Data

Policy information about availability of data

All manuscripts must include a data availability statement. This statement should provide the following information, where applicable:

- Accession codes, unique identifiers, or web links for publicly available datasets
- A description of any restrictions on data availability
- For clinical datasets or third party data, please ensure that the statement adheres to our policy

To protect patient privacy, raw whole-slide images (WSIs) data are not publicly available. Researchers requiring access to the raw data may contact the corresponding author Yanmei Zhu. Remote access to WSIs data via Virtual Private Network will only be granted upon approval from the Institutional Review Boards of Cancer Hospital of Dalian University of Technology, Dalian Central Hospital, and the Fourth Affiliated Hospital of China Medical University. All data access requests will be processed within 20 working days. Source Data are provided with this paper.

Research involving human participants, their data, or biological material

Policy information about studies with human participants or human data. See also policy information about sex, gender (identity/presentation), and sexual orientation and race, ethnicity and racism.

Reporting on sex and gender	No sex- and gender-related hypotheses were formulated at the study design stage. The primary scientific objective of this work is artificial intelligence-based identification of lymph node metastasis. The study endpoints do not aim to investigate sex and gender differences; therefore, we did not pre-specify any sex- and gender-stratified analysis plan, and no corresponding analysis were performed. Participant sex was extracted from official hospital medical records rather than participant self-reporting. Sex and gender information is only utilized to describe baseline population characteristics and is not employed as a variable for stratification or interaction analysis. The conclusions of this study are generalizable to all sex and gender groups and are not restricted to a single group.
Reporting on race, ethnicity, or other socially relevant groupings	This study did not involve any analyses or data collection related to race, ethnicity, or other socially relevant groupings. No such variables were defined, collected, or considered in the study design or analyses.
Population characteristics	The only covariate-relevant population characteristic of the human research participants was the lymph node pathological diagnosis classification labels (i.e., different metastatic types of lymph nodes). No other covariates (e.g., age, gender, or other patient-level information) were collected or analyzed in this study.
Recruitment	Lymph node tissue samples were retrospectively collected from the pathological specimen archives of Liaoning Cancer Hospital and Institute, Dalian Central Hospital and the Fourth Affiliated Hospital of China Medical University. No self-selection bias was present as all eligible samples were included. Potential selection bias was acknowledged but is not expected to affect the study results.
Ethics oversight	This retrospective, non-interventional study was approved by the Ethics Committees of Cancer Hospital of Dalian University of Technology (Approval No. KY20231115), Dalian Central Hospital (Approval No. YN2025-118-01), and the Fourth Affiliated Hospital of China Medical University (Approval No. EC-2024-KS-119). The ethics committees granted a waiver of informed consent. The full justifications for this waiver are presented below:<br>1.The application for the waiver of informed consent was submitted in accordance with Article 39 of the Measures for the Ethical Review of Biomedical Research Involving Human Subjects issued by the National Health Commission of the People' s Republic of China, which stipulates the circumstances under which informed consent may be waived for biomedical research involving human participants.<br>2.This retrospective, non-interventional study only performs retrospective analysis on archived whole-slide pathological images during routine clinical diagnosis and treatment. No additional invasive procedures, supplementary examinations or prospective data collection were conducted on participants. No additional tissue specimens were collected specifically for this study.<br>3.All direct personal identifiers (including names, identity card numbers, medical record numbers and other traceable information) were permanently removed prior to data analysis. The processed data cannot be used to re-identify individual participants, which strictly protects patient privacy and information confidentiality.<br>4.This study meets the minimal risk standard. There is no contact with participants, and no additional physical or psychological burden will be imposed on individuals. Risks such as privacy leakage, commercial exploitation of clinical data and infringement of participants' legitimate rights and interests do not exist. The risks associated with this research do not exceed those encountered in daily life.<br>5.The study would be practically infeasible without a waiver of informed consent. The dataset consists of archived whole-slide pathological images accumulated from years of routine clinical practice, and some participants cannot be reliably traced. Attempting to contact all former participants one by one to obtain informed consent would introduce substantial selection bias and impair the representativeness of the study cohort.<br>6.This study has important clinical value. The artificial intelligence algorithm developed herein enables automated evaluation of lymph node metastasis based on pathological whole-slide images. It can reduce inter-pathologist discordance, optimize diagnostic workflows and facilitate clinical management of tumour patients. The anticipated clinical benefits outweigh the minimal risks borne by participants.<br><br>No financial compensation was offered to participants in this study. Only de-identified pathological image data archived from routine clinical care were utilized. No participants were actively recruited, and no additional research-related interventions were administered to patients.

Note that full information on the approval of the study protocol must also be provided in the manuscript.

Field-specific reporting

Please select the one below that is the best fit for your research. If you are not sure, read the appropriate sections before making your selection.

- [x] Life sciences
- [ ] Behavioural & social sciences
- [ ] Ecological, evolutionary & environmental sciences

For a reference copy of the document with all sections, see nature.com/documents/nr-reporting-summary-flat.pdf

Life sciences study design

All studies must disclose on these points even when the disclosure is negative.

Sample size

This is a multicenter retrospective study. All digital whole-slide images were consecutively enrolled from single-cancer and pan-cancer patient cohorts following unified inclusion and exclusion criteria. Three independent datasets were established in total: the NIMM dataset consisting of 629 hematoxylin and eosin (H&E)-stained whole-slide images of lymph nodes; the DLZX dataset consisting of 402 H&E-stained whole-slide lymph node images; and the YDSY dataset consisting of 301 H&E-stained whole-slide lymph node images. No prospective statistical power analysis for sample size calculation was performed prior to data collection, which represents a standard practice for retrospective pathological artificial intelligence studies based on archived pathological slides.

Although the sample size of each individual dataset is limited, this study has multiple strengths verifying that the current sample size can robustly support all experimental conclusions:
1. Specimens were collected from three distinct medical institutions, covering various primary tumors of single cancer type and pan-cancer types. There are inherent variations across hospitals in slide scanning equipment, tissue section preparation standards, and pathological diagnostic interpretation. The cohort of this study exhibits greater clinical heterogeneity and better reflects real-world clinical practice, which effectively compensates for the limited sample size of each single dataset.
2. Datasets in this study suffer from scarcity of minority samples corresponding to isolated tumor cells and micrometastases. Stratified K-fold cross-validation was adopted throughout all experiments to maintain consistent distribution of the four metastatic subtypes across training and test subsets in every fold. Meanwhile, class-weighted cross-entropy loss was applied during model training to fully extract information from scarce minority samples and prevent prediction failure of minority classes caused by insufficient total samples.
3. Stratified 10-fold cross-validation was implemented for the four-class lymph node metastasis classification task, while stratified 3-fold cross-validation was used for subgroup analyses across multiple cancer types. All evaluation metrics showed minor mean fluctuations and low standard deviations, with stable performance across repeated experiments without drastic result oscillations. This indicates that the current sample scale can offset random partitioning bias, and the conclusions derived from the model possess favorable reproducibility.

Data exclusions

Exclusion criteria were:
1. Slides whose tissue was severely compressed or distorted, compromising diagnostic accuracy;
2. Faded slides that could not be re-stained because the corresponding paraffin blocks contained insufficient residual tissue;
3. Whole-slide images with poor image clarity.

Replication

The average values and standard deviations of each evaluation metric were reported over 10-fold cross-validation for overall four-class and individual classification of lymph node metastasis, and over 3-fold cross-validation for overall four-class classification across multiple and single cancer types. All repeated cross-validation experiments yielded stable and consistent model performance, and no irreproducible or unrepeatable experimental results were observed in this study.

Randomization

This study is a retrospective computational pathology study, and no allocation of participants to experimental groups was conducted. A standard straight 10-fold cross-validation method was adopted: all unique patient samples were shuffled with a fixed random seed and divided into 10 non-overlapping folds, with 9 folds used as the training set and 1 fold as the validation set in each round. This random partitioning is used for the unbiased evaluation of the model, and no additional random allocation of participants to experimental groups is required for this study.

Blinding

This study evaluated the performance of an AI algorithm in detecting lymph-node metastases across predefined research groups under known group allocation; therefore, blinding of group assignment was not feasible during data collection or analysis.

Reporting for specific materials, systems and methods

We require information from authors about some types of materials, experimental systems and methods used in many studies. Here, indicate whether each material, system or method listed is relevant to your study. If you are not sure if a list item applies to your research, read the appropriate section before selecting a response.

Materials & experimental systems

n/a	Involved in the study
☐	☐ Antibodies
☐	☐ Eukaryotic cell lines
☐	☐ Palaeontology and archaeology
☐	☐ Animals and other organisms
☐	☒ Clinical data
☐	☐ Dual use research of concern
☐	☐ Plants

Methods

n/a	Involved in the study
☐	☐ ChIP-seq
☐	☐ Flow cytometry
☐	☐ MRI-based neuroimaging

Antibodies

Antibodies used

Describe all antibodies used in the study; as applicable, provide supplier name, catalog number, clone name, and lot number.

Validation

Describe the validation of each primary antibody for the species and application, noting any validation statements on the manufacturer's website, relevant citations, antibody profiles in online databases, or data provided in the manuscript.

Eukaryotic cell lines

Policy information about cell lines and Sex and Gender in Research

Cell line source(s)

State the source of each cell line used and the sex of all primary cell lines and cells derived from human participants or vertebrate models.

Authentication

Describe the authentication procedures for each cell line used OR declare that none of the cell lines used were authenticated.

Mycoplasma contamination

Confirm that all cell lines tested negative for mycoplasma contamination OR describe the results of the testing for mycoplasma contamination OR declare that the cell lines were not tested for mycoplasma contamination.

Commonly misidentified lines
(See ICLAC register)

Name any commonly misidentified cell lines used in the study and provide a rationale for their use.

Palaeontology and Archaeology

Specimen provenance

Provide provenance information for specimens and describe permits that were obtained for the work (including the name of the issuing authority, the date of issue, and any identifying information). Permits should encompass collection and, where applicable, export.

Specimen deposition

Indicate where the specimens have been deposited to permit free access by other researchers.

Dating methods

If new dates are provided, describe how they were obtained (e.g. collection, storage, sample pretreatment and measurement), where they were obtained (i.e. lab name), the calibration program and the protocol for quality assurance OR state that no new dates are provided.

☐ Tick this box to confirm that the raw and calibrated dates are available in the paper or in Supplementary Information.

Ethics oversight

Identify the organization(s) that approved or provided guidance on the study protocol, OR state that no ethical approval or guidance was required and explain why not.

Note that full information on the approval of the study protocol must also be provided in the manuscript.

Animals and other research organisms

Policy information about studies involving animals; ARRIVE guidelines recommended for reporting animal research, and Sex and Gender in Research

Laboratory animals

For laboratory animals, report species, strain and age OR state that the study did not involve laboratory animals.

Wild animals

Provide details on animals observed in or captured in the field; report species and age where possible. Describe how animals were caught and transported and what happened to captive animals after the study (if killed, explain why and describe method; if released, say where and when) OR state that the study did not involve wild animals.

Reporting on sex

Indicate if findings apply to only one sex; describe whether sex was considered in study design, methods used for assigning sex. Provide data disaggregated for sex where this information has been collected in the source data as appropriate; provide overall numbers in this Reporting Summary. Please state if this information has not been collected. Report sex-based analyses where performed, justify reasons for lack of sex-based analysis.

Field-collected samples | *For laboratory work with field-collected samples, describe all relevant parameters such as housing, maintenance, temperature, photoperiod and end-of-experiment protocol OR state that the study did not involve samples collected from the field.*

Ethics oversight | *Identify the organization(s) that approved or provided guidance on the study protocol, OR state that no ethical approval or guidance was required and explain why not.*

Note that full information on the approval of the study protocol must also be provided in the manuscript.

Clinical data

Policy information about clinical studies

All manuscripts should comply with the ICMJE guidelines for publication of clinical research and a completed CONSORT checklist must be included with all submissions.

Clinical trial registration | *Provide the trial registration number from ClinicalTrials.gov or an equivalent agency.*

Study protocol | This is a retrospective study, and no prospective trial protocol was established.

Data collection | The NIMM dataset (662 lymph node histopathological sections) was collected from Cancer Hospital of Dalian University of Technology (Shenyang, China) from January 2018 to January 2024 and included 384 patients (135 males and 249 females; age range 26–86 years; median age 58 years [interquartile range 52–64]), including 141 gastric adenocarcinoma, 168 colorectal adenocarcinoma, 116 lung invasive adenocarcinoma, 156 breast invasive ductal carcinoma, 33 breast invasive lobular carcinoma, 36 endometrioid adenocarcinoma, and 12 thyroid papillary carcinoma sections.

The DLZX dataset (402 sections) was collected from Dalian Central Hospital (Dalian, China) from January 2018 to January 2026 and included 241 patients (70 males and 171 females; age range 23–93 years; median age 60 years [interquartile range 48–68]), including 45 gastric adenocarcinoma, 150 colorectal adenocarcinoma, 2 lung invasive adenocarcinoma, 97 breast invasive ductal carcinoma, 20 breast invasive lobular carcinoma, 85 thyroid papillary carcinoma, 2 cervical adenocarcinoma, and 1 submandibular duct adenocarcinoma sections. The

The YDSY dataset (301 sections) was collected from the Fourth Affiliated Hospital of China Medical University (Shenyang, China) from January 2018 to January 2026 and included 283 patients (103 males and 180 females; age range 20–89 years; median age 60 years [interquartile range 49–68]), including 14 gastric adenocarcinoma, 81 colorectal adenocarcinoma, 36 lung invasive adenocarcinoma, 68 breast invasive ductal carcinoma, 16 breast invasive lobular carcinoma, 73 thyroid papillary carcinoma, 5 cervical adenocarcinoma, 3 high-grade serous carcinoma of female genital tract, and 5 biliary tract adenocarcinoma sections.

Outcomes | The model performance evaluation metrics in this study include: AUC (Area Under the Curve), Accuracy, Precision, Recall, Specificity, and F1-score.

Dual use research of concern

Policy information about dual use research of concern

Hazards

Could the accidental, deliberate or reckless misuse of agents or technologies generated in the work, or the application of information presented in the manuscript, pose a threat to:

No	Yes	
☐	☐	Public health
☐	☐	National security
☐	☐	Crops and/or livestock
☐	☐	Ecosystems
☐	☐	Any other significant area

Experiments of concern

Does the work involve any of these experiments of concern:

No	Yes	
☐	☐	Demonstrate how to render a vaccine ineffective
☐	☐	Confer resistance to therapeutically useful antibiotics or antiviral agents
☐	☐	Enhance the virulence of a pathogen or render a nonpathogen virulent
☐	☐	Increase transmissibility of a pathogen
☐	☐	Alter the host range of a pathogen
☐	☐	Enable evasion of diagnostic/detection modalities
☐	☐	Enable the weaponization of a biological agent or toxin
☐	☐	Any other potentially harmful combination of experiments and agents

Plants

Seed stocks

Report on the source of all seed stocks or other plant material used. If applicable, state the seed stock centre and catalogue number. If plant specimens were collected from the field, describe the collection location, date and sampling procedures.

Novel plant genotypes

Describe the methods by which all novel plant genotypes were produced. This includes those generated by transgenic approaches, gene editing, chemical/radiation-based mutagenesis and hybridization. For transgenic lines, describe the transformation method, the number of independent lines analyzed and the generation upon which experiments were performed. For gene-edited lines, describe the editor used, the endogenous sequence targeted for editing, the targeting guide RNA sequence (if applicable) and how the editor was applied.

Authentication

Describe any authentication procedures for each seed stock used or novel genotype generated. Describe any experiments used to assess the effect of a mutation and, where applicable, how potential secondary effects (e.g. second site T-DNA insertions, mosiacism, off-target gene editing) were examined.

ChIP-seq

Data deposition

☐ Confirm that both raw and final processed data have been deposited in a public database such as GEO.

☐ Confirm that you have deposited or provided access to graph files (e.g. BED files) for the called peaks.

Data access links
May remain private before publication.

For "Initial submission" or "Revised version" documents, provide reviewer access links. For your "Final submission" document, provide a link to the deposited data.

Files in database submission

Provide a list of all files available in the database submission.

Genome browser session
(e.g. UCSC)

Provide a link to an anonymized genome browser session for "Initial submission" and "Revised version" documents only, to enable peer review. Write "no longer applicable" for "Final submission" documents.

Methodology

Replicates

Describe the experimental replicates, specifying number, type and replicate agreement.

Sequencing depth

Describe the sequencing depth for each experiment, providing the total number of reads, uniquely mapped reads, length of reads and whether they were paired- or single-end.

Antibodies

Describe the antibodies used for the ChIP-seq experiments; as applicable, provide supplier name, catalog number, clone name, and lot number.

Peak calling parameters

Specify the command line program and parameters used for read mapping and peak calling, including the ChIP, control and index files used.

Data quality

Describe the methods used to ensure data quality in full detail, including how many peaks are at FDR 5% and above 5-fold enrichment.

Software

Describe the software used to collect and analyze the ChIP-seq data. For custom code that has been deposited into a community repository, provide accession details.

Flow Cytometry

Plots

Confirm that:

☐ The axis labels state the marker and fluorochrome used (e.g. CD4-FITC).

☐ The axis scales are clearly visible. Include numbers along axes only for bottom left plot of group (a 'group' is an analysis of identical markers).

☐ All plots are contour plots with outliers or pseudocolor plots.

☐ A numerical value for number of cells or percentage (with statistics) is provided.

Methodology

Sample preparation

Describe the sample preparation, detailing the biological source of the cells and any tissue processing steps used.

Instrument

Identify the instrument used for data collection, specifying make and model number.

Software

Describe the software used to collect and analyze the flow cytometry data. For custom code that has been deposited into a community repository, provide accession details.

Cell population abundance | *Describe the abundance of the relevant cell populations within post-sort fractions, providing details on the purity of the samples and how it was determined.*

Gating strategy | *Describe the gating strategy used for all relevant experiments, specifying the preliminary FSC/SSC gates of the starting cell population, indicating where boundaries between "positive" and "negative" staining cell populations are defined.*

☐ Tick this box to confirm that a figure exemplifying the gating strategy is provided in the Supplementary Information.

Magnetic resonance imaging

Experimental design

Design type | *Indicate task or resting state; event-related or block design.*

Design specifications | *Specify the number of blocks, trials or experimental units per session and/or subject, and specify the length of each trial or block (if trials are blocked) and interval between trials.*

Behavioral performance measures | *State number and/or type of variables recorded (e.g. correct button press, response time) and what statistics were used to establish that the subjects were performing the task as expected (e.g. mean, range, and/or standard deviation across subjects).*

Acquisition

Imaging type(s) | *Specify: functional, structural, diffusion, perfusion.*

Field strength | *Specify in Tesla*

Sequence & imaging parameters | *Specify the pulse sequence type (gradient echo, spin echo, etc.), imaging type (EPI, spiral, etc.), field of view, matrix size, slice thickness, orientation and TE/TR/flip angle.*

Area of acquisition | *State whether a whole brain scan was used OR define the area of acquisition, describing how the region was determined.*

Diffusion MRI ☐ Used ☐ Not used

Preprocessing

Preprocessing software | Data preprocessing was performed using the public WSI preprocessing repository (https://github.com/mahmoodlab/CLAM). WSIs were processed with Otsu thresholding to remove background regions, and the tissue regions were segmented into non-overlapping 256×256 pixel image patches.

Normalization | Linear normalization was performed on each 256×256 pixel RGB image patch.

Normalization template | The image patches were uniformly transformed into a standard feature space based on the statistical properties of the ImageNet dataset. The specific parameters are mean = [0.485, 0.456, 0.406], std = [0.229, 0.224, 0.225]. This step was implemented via torchvision.transforms.Normalize.

Noise and artifact removal | Non-tissue background regions and common artifacts (such as marker pen marks and air bubbles) were excluded during the patching process. Tissue segmentation masks were automatically generated using the Otsu thresholding method, and only image patches containing valid pathological tissue were retained for subsequent analysis.

Volume censoring | *Define your software and/or method and criteria for volume censoring, and state the extent of such censoring.*

Statistical modeling & inference

Model type and settings | *Specify type (mass univariate, multivariate, RSA, predictive, etc.) and describe essential details of the model at the first and second levels (e.g. fixed, random or mixed effects; drift or auto-correlation).*

Effect(s) tested | *Define precise effect in terms of the task or stimulus conditions instead of psychological concepts and indicate whether ANOVA or factorial designs were used.*

Specify type of analysis: ☐ Whole brain ☐ ROI-based ☐ Both

Statistic type for inference | *Specify voxel-wise or cluster-wise and report all relevant parameters for cluster-wise methods.*

(See Eklund et al. 2016)

Correction | *Describe the type of correction and how it is obtained for multiple comparisons (e.g. FWE, FDR, permutation or Monte Carlo).*

Models & analysis

n/a	Involved in the study
☐	☐ Functional and/or effective connectivity
☐	☐ Graph analysis
☐	☐ Multivariate modeling or predictive analysis

Functional and/or effective connectivity	*Report the measures of dependence used and the model details (e.g. Pearson correlation, partial correlation, mutual information).*
Graph analysis	*Report the dependent variable and connectivity measure, specifying weighted graph or binarized graph, subject- or group-level, and the global and/or node summaries used (e.g. clustering coefficient, efficiency, etc.).*
Multivariate modeling and predictive analysis	*Specify independent variables, features extraction and dimension reduction, model, training and evaluation metrics.*
