# Supplementary material for: Weakly supervised artificial intelligence for multi-cancer detection of lymph node metastasis on whole slide images
Source: Nat Commun. 2026 Aug 14;17:9770. doi: 10.1038/s41467-026-76410-w (PMC13572452; doi:10.1038/s41467-026-76410-w)
Supplement: Supplementary file 3 [file 41467_2026_76410_MOESM3_ESM.pdf]

nature portfolio

Peer Review File

Weakly Supervised Artificial Intelligence for Multi-cancer Detection of Lymph Node Metastasis on Whole Slide Images

Corresponding Author: Professor Yanmei Zhu

This file contains all reviewer reports in order by version, followed by all author rebuttals in order by version.

Version 0:

Reviewer comments:

Reviewer #1

(Remarks to the Author)
This manuscript presents a weakly supervised deep learning approach, MambaMIL+HiLA-MIL, for the classification of lymph node metastasis (LNM) from whole slide images (WSIs). The study reports impressive results on both internal and external datasets and addresses an important problem in computational pathology. However, several key issues need to be addressed before the paper can be considered for publication.
1. Although the authors employed three-fold cross-validation, this level of evaluation is insufficient to demonstrate statistical robustness. A ten-fold cross-validation would provide a more reliable estimate of model variance. Moreover, statistical significance tests (e.g., DeLong test, p-values) should be included to support claims of superiority over baseline methods.
2. Recent pathology foundation models such as UNI, Virchow, and Gigapath have demonstrated superior performance and generalization compared to ResNet-based feature extractors for LNM classification. The omission of these models in the comparison is a significant limitation and should be addressed or at least discussed.
3. The proposed method is built upon MambaMIL, yet the manuscript lacks sufficient technical detail regarding how the attention mechanism in MambaMIL operates. A clearer explanation or visualization of the attention computation is necessary for reproducibility and understanding.
4. The NIMM dataset exhibits strong class imbalance, particularly in the ITC and micro-metastasis categories. The manuscript should clarify how the dataset was partitioned to ensure balanced representation across folds and what measures (e.g., oversampling, reweighting) were taken to mitigate potential bias.
While the study addresses an important clinical task and achieves competitive performance, the methodological novelty is limited. The proposed attention separation loss appears to be a relatively minor modification to MambaMIL, and without robust statistical validation or comparison to modern pathology foundation models, the claimed improvements are not yet convincing. Addressing the above concerns will substantially strengthen the paper.

(Remarks on code availability)
The provided code enables reproducibility by giving the model training process. However, they are recommended to provide a clearer description of the inference procedure. Additionally, releasing the pretrained model weights would further enhance transparency and reproducibility.

Reviewer #2

(Remarks to the Author)
The work is significant in that the authors have developed an improved classification model to detect metastatic carcinoma in lymph nodes using whole slide images (MambaMIL+HiLA-MIL). They trained the model on an internal set of pathology images of four cancers from their institution and then evaluated the model on two external WSI sets of breast cancer lymph nodes (CAMELYON16 and CAMELYON17). The results and statistical analyses show improvement in detection of even isolated tumor cells (ITCs) as well as overall improved sensitivity and specificity compared to six comparable weak supervised learning models.

The authors summarize appropriately the prior work in the specific focus of AI detection of metastases in lymph nodes.

Methods are sound for an initial foray using their innovative model. Further work will require a multi-institutional set of WSIs

to contend with the issues of variation in fixation, processing and staining of tissue sections.

The authors describe the model "for detection of pan-cancer LNM". This is overstating the breadth of cancers evaluated in their model which comprised four carcinomas only: gastric adenocarcinoma, colorectal adenocarcinoma, lung adenocarcinoma and breast ductal carcinoma. Therefore, the assertion that the model is a "pan-cancer" detector of lymph node metastases should be changed to "multi-cancer" detector. This is because carcinomas have a broad range of histologic cytologic characteristics that could significantly diminish the capabilities of the model to accurately detect metastatic deposits. Stating that their model is "pan-cancer" is an over-reach. Notably, for the largest dataset, breast carcinoma, only ductal carcinomas were evaluated which excludes at least 10% of other tumors such as lobular carcinoma the cells of which can closely mimic lymphocytes of a lymph node.

It would be helpful to examine the incorrectly classified slides in each tumor category to determine whether repetitive factors resulted in misclassification. This would require pathologist participation.

In summary, the issue of identifying lymph node metastases is clinically important and in fact crucial for determining treatment as well as prognosis. The method developed is a further advance in optimizing machine learning tools in this effort. Further work is necessary including diversification of cancer case types in each category (such as including lobular carcinoma of the breast) and in testing (and potentially re-training/validating) the model in WSI datasets generated from multiple institutions.

(Remarks on code availability)

Version 1:

Reviewer comments:

Reviewer #1

(Remarks to the Author)
The authors have resolved my comments.

(Remarks on code availability)
The authors have provided a thorough instruction on reproducing their code.

Reviewer #2

(Remarks to the Author)
The authors have addressed my comments in appropriate revisions. I defer to Reviewer #1 for assessing author responses and revisions to Reviewer 1's detailed methodological review.

From my perspective, the manuscript is acceptable for publication.

(Remarks on code availability)

Open Access This Peer Review File is licensed under a Creative Commons Attribution 4.0 International License, which permits use, sharing, adaptation, distribution and reproduction in any medium or format, as long as you give appropriate credit to the original author(s) and the source, provide a link to the Creative Commons license, and indicate if changes were made.
In cases where reviewers are anonymous, credit should be given to 'Anonymous Referee' and the source.
The images or other third party material in this Peer Review File are included in the article's Creative Commons license, unless indicated otherwise in a credit line to the material. If material is not included in the article's Creative Commons license and your intended use is not permitted by statutory regulation or exceeds the permitted use, you will need to obtain permission directly from the copyright holder.
To view a copy of this license, visit https://creativecommons.org/licenses/by/4.0/

REVIEWER COMMENTS

Reviewer #1 (Remarks to the Author): Expert in machine learning and deep learning for digital pathology

This manuscript presents a weakly supervised deep learning approach, MambaMIL+HiLA-MIL, for the classification of lymph node metastasis (LNM) from whole slide images (WSIs). The study reports impressive results on both internal and external datasets and addresses an important problem in computational pathology. However, several key issues need to be addressed before the paper can be considered for publication.

1. Although the authors employed three-fold cross-validation, this level of evaluation is insufficient to demonstrate statistical robustness. A ten-fold cross-validation would provide a more reliable estimate of model variance. Moreover, statistical significance tests (e.g., DeLong test, p-values) should be included to support claims of superiority over baseline methods.

Response：

Thank you for your advice.

We recognize that 10-fold cross-validation can yield more robust estimation when the sample size is sufficient. Therefore, we strictly adopted 10-fold cross-validation to evaluate model performance in the overall four-class classification of lymph node metastasis (LNM), individual LNM categories, and comparative experiments of the four feature extractors. A two-tailed paired permutation test with 10,000 permutations was further conducted to analyze the statistical significance of performance differences among models and feature extractors.

Nevertheless, when evaluating the LNM diagnostic performance of the model across multi-cancer and individual cancer types, some cancer subtypes had limited sample sizes. For instance, the NIMM dataset contained only 1 case of ITCs and 7 cases of micrometastasis for gastric adenocarcinoma. Mandatory adoption of 10-fold cross-validation would result in an excessively small number of training/validation samples per fold, easily induce distribution shifts across folds, and fail to preserve the stratified structure of the original dataset as well as the robustness of validation results.

Accordingly, 3-fold cross-validation was employed in this study to ensure that the proportion of each category in every fold remained largely consistent with that in the original dataset. Meanwhile, paired permutation tests with 1,000 permutations were also performed on the 3-fold results to quantify the statistical significance of performance discrepancies between models and strengthen the reliability of the conclusions. The modified results are presented in Table 2, and Supplementary Tables 3 and 4.

2. Recent pathology foundation models such as UNI, Virchow, and Gigapath have demonstrated superior performance and generalization compared to ResNet-based feature extractors for LNM classification. The omission of these models in the comparison is a significant limitation and should be addressed or at least discussed.

Response：

Thank you for your suggestions.

We have incorporated pathological foundation models including UNI, Virchow, and Gigapath, and supplemented corresponding comparative experiments. Please refer to the "Comparison with Feature Extractor Models" section in the Results part of the revised manuscript (Page 12) .

3. The proposed method is built upon MambaMIL, yet the manuscript lacks sufficient technical detail regarding how the attention mechanism in MambaMIL operates. A clearer explanation or visualization of the attention computation is necessary for reproducibility and understanding.

Response：

We sincerely appreciate the reviewer's valuable feedback regarding the technical details of the attention mechanism in MambaMIL. We provide a more explicit and reproducible description of the attention computation and supplement it with an architecture diagram (Supplementary Fig. 8 in the revised manuscript) to illustrate the full pipeline. Please refer to the "Attention Mechanism" section in the Methods part of the revised manuscript (Page 26) .

Specifically, we introduce the MambaMIL framework to model long-range dependencies and complex tissue interactions in whole slide images (WSIs). Compared with Transformer-based methods such as TransMIL and CNN-based MIL approaches such as AB-MIL, the proposed framework integrates convolutional feature extraction with the Vision Mamba architecture to capture both local morphological patterns and global contextual relationships among instances. For each WSI, patch-level instance features are first extracted using a pre-trained feature encoder, including ResNet50, UNI, Gigapath, or Virchow. The dimensionality of the extracted features depends on the backbone encoder: for ResNet50 and UNI, the feature dimension is 1024; for Gigapath it is 1536; and for Virchow it is 2560. These features are subsequently normalized and fed into a Vision Mamba block, which models long-range spatial dependencies across all patches using bidirectional state space models (SSMs). The forward and backward SSM branches capture sequential context in both directions, while an activation branch learns adaptive gating weights to dynamically modulate the information flow. The output of the Vision Mamba block is then fused with the original CNN features through a residual connection, producing enhanced instance representations z_i that integrate both local convolutional features and global contextual information.

To obtain slide-level predictions, we adopt an attention-based multiple instance learning (MIL) aggregation mechanism. The enhanced instance features z_i are first passed through a two-layer multilayer perceptron (MLP) to generate raw attention logits $\tilde{a}_i$. These logits are normalized using a softmax function to obtain attention scores $a_i \in [0,1]$, ensuring that the contributions of all N instances are properly normalized:

$$a_i = \frac{exp(\tilde{a}_i)}{\sum_{j=1}^{N} exp(\tilde{a}_j)}.$$

The attention scores are then used to weight the corresponding instance features, and a weighted aggregation is performed to obtain a slide-level representation:

$$\mathrm{h} = \sum_{i=1}^{N} a_i z_i \,.$$

Finally, the aggregated representation is fed into a fully connected classification layer followed by a softmax activation to produce the final slide-level prediction:

$$\hat{Y}=Softmax(FC(\mathrm{h}))$$

4. The NIMM dataset exhibits strong class imbalance, particularly in the ITCs and micro-metastasis categories. The manuscript should clarify how the dataset was partitioned to ensure balanced representation across folds and what measures (e.g., oversampling, reweighting) were taken to mitigate potential bias.

Response：

We thank the reviewer for pointing out the class imbalance issue in the NIMM dataset. We note that the numbers of samples in the isolated tumor cells (ITCs) and micrometastasis categories are relatively small in this dataset. Therefore, in our experimental design we adopted measures at both the data splitting stage and the model training stage to mitigate the potential bias caused by class imbalance. Please refer to the "WSI processing" section (Page 24) and "Student Optimization" section (Page 27-28) in the Methods part of the revised manuscript.

First, at the data splitting stage, we employed **Stratified K-Fold cross-validation (StratifiedKFold)** to perform stratified partitioning of the dataset. Unlike ordinary K-Fold random splitting, StratifiedKFold performs stratified sampling based on the class labels of the samples, so that the class proportions in each fold remain consistent with those of the overall dataset. Specifically, we used the label of each WSI as the stratification criterion and applied the **StratifiedKFold function in the scikit-learn library** to divide the dataset into K folds. In each round of experiments, one fold is selected as the test set while the remaining folds are used as the training set. This strategy ensures that both the training and test sets in each fold contain all classes and maintain consistent class distributions, thereby avoiding class bias caused by random partitioning.

Second, at the model training stage, we adopted a **class-weighted cross-entropy loss**

to further alleviate the class imbalance problem. Specifically, the class weights are computed according to the number of samples in each class within the training set. Let n_c denote the number of samples in class c, C the total number of classes, and N the total number of samples in the training set. The class weight w_c is computed using an inverse-frequency strategy:

$$w_c = \frac{N}{C \square n_c}.$$

These weights are incorporated into the cross-entropy loss function as:

$$L = -\sum_{c=1}^{C} w_c y_c \log(p_c),$$

Where y_c is the ground-truth label indicator and p_c is the predicted probability for class c. In practice, the weights are calculated for each training fold and passed to the weighted cross-entropy implementation in the training framework. Through this mechanism, classes with fewer samples (such as ITCs and micrometastasis) contribute more to the training loss, encouraging the model to pay greater attention to minority classes during optimization and thereby reducing bias toward majority classes.

Through the use of the class-weighted loss function, we effectively mitigate the potential impact of class imbalance in the NIMM dataset within our experimental setup. The relevant description has been added to the experimental settings section of the revised manuscript to improve the transparency and reproducibility of the experimental design.

While the study addresses an important clinical task and achieves competitive performance, the methodological novelty is limited. The proposed attention separation loss appears to be a relatively minor modification to MambaMIL, and without robust statistical validation or comparison to modern pathology foundation models, the claimed improvements are not yet convincing. Addressing the above concerns will substantially strengthen the paper.

Reviewer #1 (Remarks on code availability):

The provided code enables reproducibility by giving the model training process. However, they are recommended to provide a clearer description of the inference procedure. Additionally, releasing the pretrained model weights would further enhance transparency and reproducibility.

Response：

Thank you for your advice.

We have provided a clearer description of the inference procedure in the Methods–Inference Pipeline section of the revised manuscript (Page 28-29) , and have released the pretrained model weights in a GitHub repository at https://github.com/YAOSL98/HiLA-MIL/tree/main/Demo/checkpoints. Furthermore, we have updated the GPU model used in this experiment and supplemented the computation time as specified in the machine learning checklist in the Methods–Implementation Details section (Page 29-30) .

Reviewer #2 (Remarks to the Author): Expert in cancer pathology and lymph node metastasis

The work is significant in that the authors have developed an improved classification model to detect metastatic carcinoma in lymph nodes using whole slide images (MambaMIL+HiLA-MIL). They trained the model on an internal set of pathology images of four cancers from their institution and then evaluated the model on two external WSI sets of breast cancer lymph nodes (CAMELYON16 and CAMELYON17). The results and statistical analyses show improvement in detection of even isolated tumor cells (ITCs) as well as overall improved sensitivity and specificity compared to six comparable weak supervised learning models.

The authors summarize appropriately the prior work in the specific focus of AI detection of metastases in lymph nodes.

1. Methods are sound for an initial foray using their innovative model. Further work will require a multi-institutional set of WSIs to contend with the issues of variation in fixation, processing and staining of tissue sections.

Response：

Thank you for your suggestions.

We have incorporated lymph node WSIs from two external datasets to address the issues of variation in fixation, processing, and staining of tissue sections. Please refer to the Methods-Dataset Description section (Page 23) and Results section (Page 5-15) in the revised manuscript.

2. The authors describe the model "for detection of pan-cancer LNM". This is overstating the breadth of cancers evaluated in their model which comprised four carcinomas only: gastric adenocarcinoma, colorectal adenocarcinoma, lung adenocarcinoma and breast ductal carcinoma. Therefore, the assertion that the model is a "pan-cancer" detector of lymph node metastases should be changed to "multi-cancer" detector. This is because carcinomas have a broad range of histologic cytologic characteristics that could significantly diminish the capabilities of the model to accurately detect metastatic deposits. Stating that their model is "pan-cancer" is an over-

reach. Notably, for the largest dataset, breast carcinoma, only ductal carcinomas were evaluated which excludes at least 10% of other tumors such as lobular carcinoma the cells of which can closely mimic lymphocytes of a lymph node.

Response：

Thank you for your proposals.

We have changed the “pan-cancer” detector of lymph node metastases to a “multi-cancer” detector. Furthermore, we have included lymph node WSIs from breast invasive lobular carcinoma to evaluate the performance of our multi-cancer detector for lymph node metastasis. Please refer to the "Comparison of Multi-Cancer and Single-Cancer" section in the Results part of the revised manuscript (Page 8-12).

3. It would be helpful to examine the incorrectly classified slides in each tumor category to determine whether repetitive factors resulted in misclassification. This would require pathologist participation.

Response：

Thank you for your suggestions.

We have examined the incorrectly classified slides in each tumor category to determine whether repetitive factors resulted in misclassification. Please refer to the "Qualitative Comparison of Visualization" section in the Results part of the revised manuscript (Page 15).

4. In summary, the issue of identifying lymph node metastases is clinically important and in fact crucial for determining treatment as well as prognosis. The method developed is a further advance in optimizing machine learning tools in this effort. Further work is necessary including diversification of cancer case types in each category (such as including lobular carcinoma of the breast) and in testing (and potentially re-training/validating) the model in WSI datasets generated from multiple institutions.

Response：

Thank you for your advice.

We have re-evaluated the model using WSI datasets generated by multiple institutions, including cases of breast invasive lobular carcinoma. Please refer to the responses to the previous Questions 1 and 2.

See Nature Portfolio’s author and referees' website at www.nature.com/authors for information about policies, services and author benefits.

This email has been sent through the Springer Nature Tracking System NY-610A-NPG&MTS

Confidentiality Statement:

This e-mail is confidential and subject to copyright. Any unauthorised use or disclosure of its contents is prohibited. If you have received this email in error please notify our Manuscript Tracking System Helpdesk team at http://platformsupport.nature.com .

Details of the confidentiality and pre-publicity policy may be found here http://www.nature.com/authors/policies/confidentiality.html

Privacy Policy | Update Profile
